# A photo-SAR study of photoswitchable azobenzene tubulin-inhibiting antimitotics identifying a general method for near-quantitative photocontrol†

**DOI:** 10.1039/d4sc03072a

**Published:** 2024-07-02

**Authors:** Martin Reynders, Małgorzata Garścia, Adrian Müller-Deku, Maximilian Wranik, Kristina Krauskopf, Luis de la Osa de la Rosa, Konstantin Schaffer, Anna Jötten, Alexander Rode, Valentin Stierle, Yvonne Kraus, Benedikt Baumgartner, Ahmed Ali, Andrei Bubeneck, Trina Seal, Michel O. Steinmetz, Philipp Paulitschke, Oliver Thorn-Seshold

**Affiliations:** a Faculty of Chemistry and Pharmacy, Ludwig-Maximilians-University Munich Munich 81377 Germany oliver.thorn-seshold@cup.lmu.de; b Laboratory of Biomolecular Research, Division of Biology and Chemistry, Paul Scherrer Institut Villigen 5232 Switzerland; c Faculty of Physics and Center for NanoScience (CeNS), Ludwig-Maximilians-University Munich Munich 80539 Germany; d PHIO Scientific GmbH Munich 81371 Germany; e Biozentrum, University of Basel Basel 4056 Switzerland

## Abstract

Azobenzene analogues of the tubulin polymerisation inhibitor combretastatin A4 (PSTs) were previously developed to optically control microtubule dynamics in living systems, with subsecond response time and single-cell spatial precision, by reversible *in situ* photoswitching of their bioactivity with near-UV/visible light. First-generation PSTs were sufficiently potent and photoswitchable for use in live cells and embryos. However, the link between their seconds-scale and hours-scale bioactivity remained untested. Furthermore, the scope for modifications to tune their photo-structure–activity-relationship or expand their function was unknown. Here, we used large-field-of-view, long-term tandem photoswitching/microscopy to reveal the temporal onset of cytostatic effects. We then synthesised a panel of novel PSTs exploring structural variations that tune photoresponse wavelengths and lipophilicity, identifying promising blue-shifted analogues that are better-compatible with GFP/YFP imaging. Taken together, these results can guide new design and applications for photoswitchable microtubule inhibitors. We also identified tolerated sites for linkers to attach functional cargos; and we tested fluorophores, aiming at RET isomerisation or reporter probes. Instead we found that these antennas greatly enhance long-wavelength single-photon photoisomerisation, by an as-yet un-explored mechanism, that can now drive general progress towards near-quantitative long-wavelength photoswitching of photopharmaceuticals in living systems, with minimal molecular redesign and broad scope.

## Abstract 2

We develop reagents to optically control microtubule dynamics, with (1) lensless microscopy to track their bioactivity; (2) photo-SAR to find GFP-compatible reagents; & (3) fluorophore antennas to drive single-photon isomerisation even ≥600 nm.

## Graphical abstract

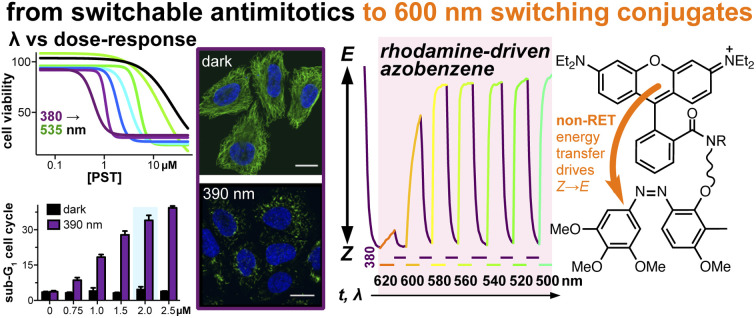

## Introduction

### MT biology needs spatiotemporally precise reagents

**Fig. 1:**
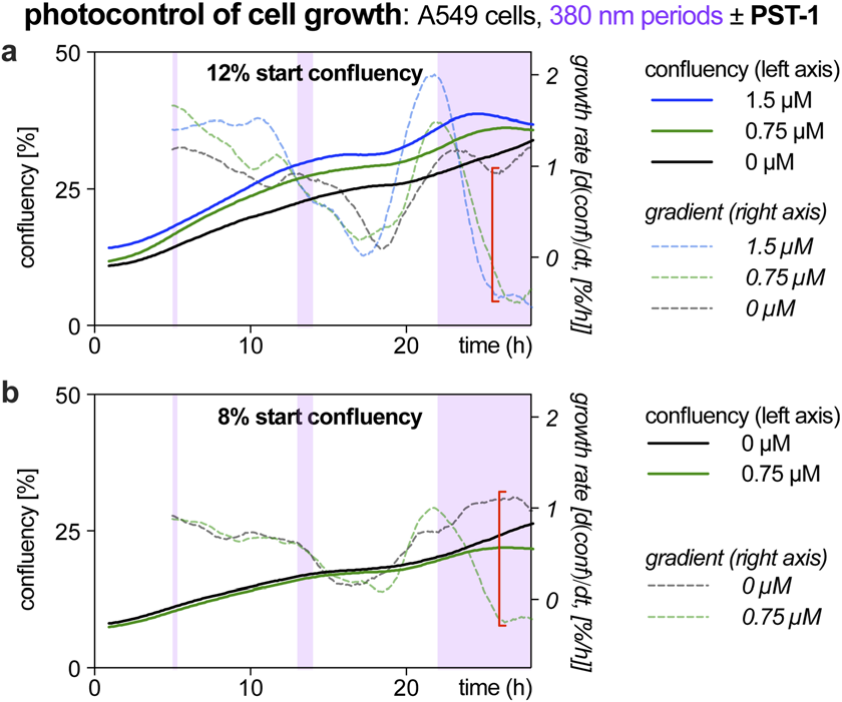
Exposure time-dependent onset of antimitotic effects with *Z*-PST-1. A549 lung cancer cells seeded at (a) 12% or (b) 8% confluency were treated with PST-1, and pulsed irradiations at 380 nm were applied during the purple-boxed timespans (durations 20 min, 1 h, or >6 h respectively; monitoring by RFLM; details at Fig. S10†).

The protein tubulin is dynamically polymerised to form microtubules (MTs), major cellular scaffolds whose structure and remodelling underlie diverse processes from intracellular transport and cellular motility to the function of the mitotic spindle during proliferation.^1^ Small molecule drugs that interfere with MT structure and dynamics are indispensable as tools for research in and around cytoskeletal biology.^2^ These include taxanes, vinca alkaloids,^3,4^ and inhibitors binding at the colchicine site, *e.g.*, colchicine, combretastatin A-4 (CA4, Fig. 1a), and their closely isosteric analogues^5^ (for more detail, see ESI Note 1†). However, it is near-impossible to use these drugs to study particular roles of MTs in specific cell regions, cell populations, or tissues, at precisely defined times, since their activity cannot be spatially or temporally directed.^6^ Developing spatiotemporally-targetable MT modulators has thus been an important goal, and optical control strategies have been prioritised since light can be applied with high spatiotemporal precision even *in vivo*.^7,8^ Although a few optogenetic MT-modulating tools were recently created,^9–11^ most research has focused on chemical tools. Irreversibly photobleachable or photouncageable inhibitors were the first developed; these were followed by tools using C=C double bond photoisomerisation that is irreversible in practice.^12–17^ However, irreversible approaches cannot overcome the diffusion of the active drug, so their spatiotemporal resolution is limited.

### Photoswitchable MT reagents

**Fig. 2:**
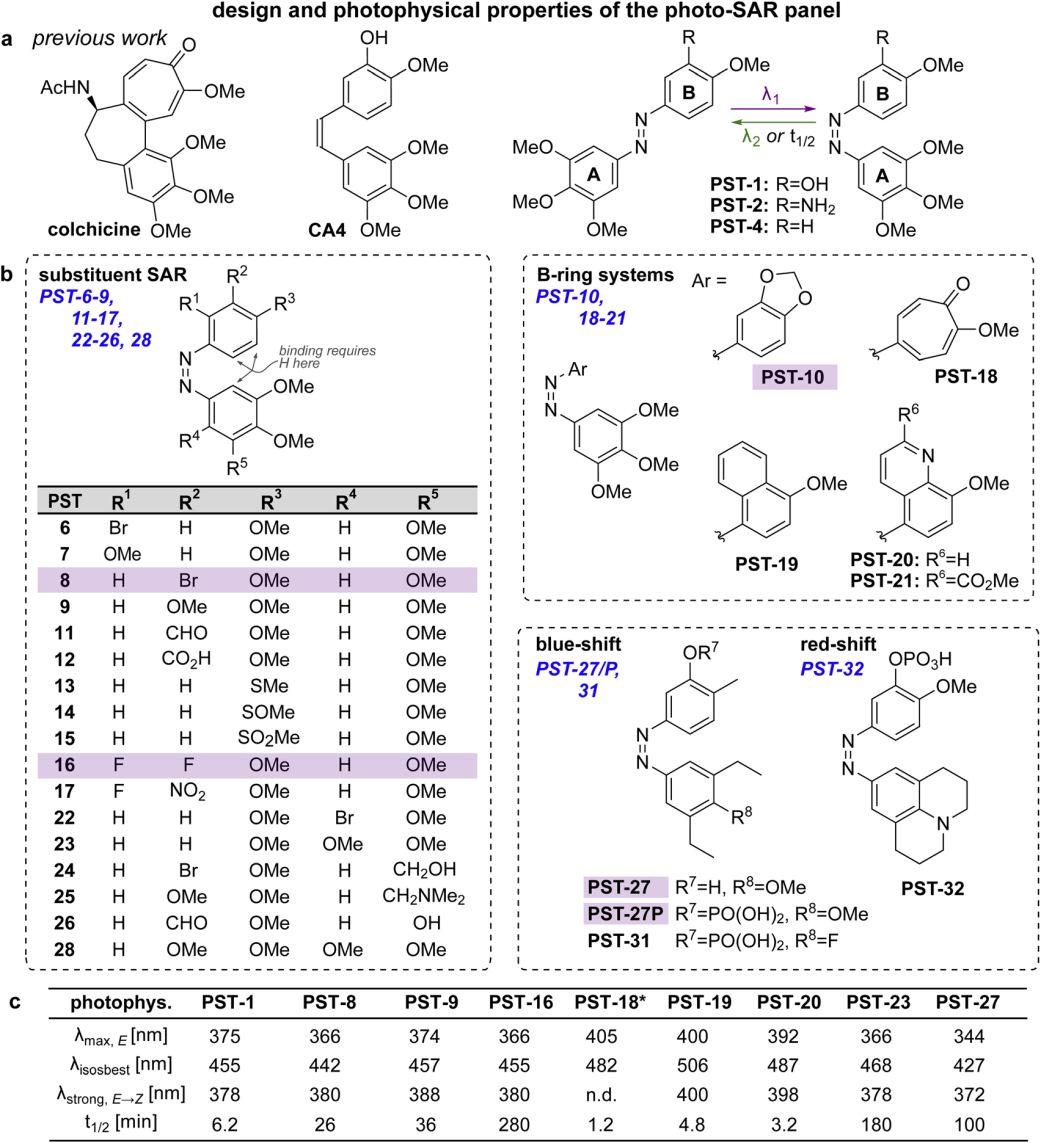
Design, synthesis and photoswitching. (a) Parent structures: colchicine, CA4, and prior PST-1/2/4. (b) Initial PSAR panel. (c) Photoswitching of key compounds: peak *E*-absorbance wavelength *λ*_max,*E*_; isosbestic point *λ*_iso_; wavelength with max PSS% *Z λ*_strong,*E* → *Z*_; thermal relaxation half-life *t*_1/2_. PSTs are drawn as *Z*-isomers for easier comparison to CA4. See also Fig. S1, S2 and Table S1.†

Spatially and temporally patterning bioactivity instead demands reversible switching *in situ* in live cells, over many off ↔ on cycles. Azobenzene-based “PSTs” that are structural analogues of CA4 (Fig. 2a) are the most widely-applied photopharmaceutical MT tools. PSTs can be reversibly photoswitched between bio-inactive *trans* and >100-fold more potent *cis* isomers,^18^ with full photostability, by low intensity near-UV and visible light: a combination of features that out-performs almost ^19^ all other photoresponsive MT inhibitors.^20^ In cell biology, PSTs can photocontrol MT architecture, dynamics, and many MT-dependent processes with excellent spatiotemporal precision.^21,22^ Streu,^23^ Hartman,^24^ Rastogi and Brittain^25^ reported complementary aspects of PST biochemistry; and we applied soluble *in vivo*-compatible prodrugs PST-1P and PST-2S (Fig. 2a) to study complex phenomena in cultured cells^26,27^ and in developing embryos.^28,29^

### Needs for improving MT reagents

Despite these applications,^21^ there is much room to improve the utility of PSTs as tool compounds. For this, the chemical space for PSTs must first be characterised and understood. Though structure–activity-relationships (SAR) for combretastatin-like stilbenes are well known,^30^*cis*-PST potency is affected by unique factors that do not apply to the isosteric stilbenes (*e.g.*, PSTs have >20-fold lower potency than expected, for reasons that are still unclear), and PST*cis* ⇄ *trans* switching adds new dimensions of structure–property relationships that must be balanced for performance. Therefore, we aimed to explore SAR and photophysical tuning of bioactive PST derivatives in a “photo-SAR” or PSAR study, in three areas: (i) classical SAR, to find the restraints on *cis*-PST structure needed to keep their potency useful within their solubility range; (ii) chemical biology, to find what modifications for developing multi-functional tool compounds are tolerated; (iii) photochemistry, to modify their practical performance (*e.g.*, incremental absorption band shifting through substitutions, or larger modifications that test the limits of what photostationary states can be accessed in bioactive derivatives). Rastogi and Brittain^25^ have made the only other systematic study of PST derivatives, though mostly this was limited to varying *meta*-substituents on the B ring: a separate scope of structural explorations that complements the PSAR developed here.

Finally, even the link between rapid-onset MT-inhibiting effects seen in live cell imaging assays (<seconds), and the long-term antimitotic effects that are the only cellular readout assessed in most photopharmacology studies (since easily and cheaply done in parallel throughput), has not been convincingly explored for this compound class (or indeed for most other photopharmaceuticals). However, it is necessary to confirm that the transition from short-term to long-term effects is indeed mechanistically linked, before one can be confident that cheap long-term assays can guide reagent development for the painstaking short-term imaging and photocontrol studies where reagents are ultimately supposed to perform. We therefore resolved to begin by monitoring this transitional link in time.

## Results and discussion

### Time-dependency of photopharmaceutical effects

Like many photopharmaceuticals, PSTs undergo *Z* → *E* relaxation over time. In short-term assays where MT dynamics are monitored directly, this is usually irrelevant since high-intensity bidirectional isomerisations are used to jump between photostationary states (PSSs). However, in long-term assays (>24 hours) where indirect, downstream effects such as cell death are monitored, illuminations are often applied by low intensity light pulses every 1–5 minutes, aiming to build up and maintain a PSS throughout the experiment. As far as we know, no studies have explored the time-dependency of these readouts, so we were motivated to find out (1) how much *E* → *Z* illumination time is needed for cellular responses to be detectable *via* the usual long-term readouts (combines progressive *E* → *Z* isomerisation with cell inhibition); and (2) how much time is needed during dark phases for cells to resume normal behaviour (combines *Z* → *E* relaxation with biological recovery).

Such experiments would be highly time-consuming in endpoint assays (needs a great many endpoints to trace evolution over time), or in typical microscopy (small fields of view mean only few cells can be tracked in parallel). However, reconstruction-free lensless microscopy (RFLM) is ideally suited to track large numbers of cells non-invasively in parallel in such longitudinal assays. RFLM setups can be very compact (10 × 10 × 15 cm) and can simply be placed in an ordinary cell culture incubator to image and track thousands of cells per condition,^31^ over many conditions in parallel (here, eight). We modified an RFLM setup to co-apply 380 nm light at typical long-term low-intensity settings, during defined phases, for *in situ E* → *Z* photoswitching.

With short photoactivation periods (20 min, 1 h) no effects on proliferation were detected. However, after *ca.* 3 h of photoactivation, antimitotic effects were notable, since growth rates (rate of change of confluency over time) became strongly dependent on presence or absence of PST (Fig. 1, red brackets). This reassured us that the PSAR of PSTs would be reliably assessed in typical long-term assays (24–48 h), even though we expected them to have differences in their efficiencies of bulk photoisomerisation and rates of *Z* → *E* relaxation.

### Photo-structure–activity-relationship (PSAR) study

#### PSAR panel design

The SAR of stilbene-type combretastatin analogues, and the X-ray structure of the combretastatin–tubulin complex, suggest that one A-ring *ortho* position, and a neighbouring *ortho* and *meta* position on the B-ring, should be the most tolerant of substitutions:^30,32^ but this PSAR has not been tested for azobenzene analogues. We synthesised a panel of 29 novel azobenzenes and azoheteroarenes (PST-6–32, Fig. 2) to test these structural variations, primarily by diazonium couplings (see ESI†). Their photophysical properties, including *E* and *Z* isomer absorption spectra, PSS compositions, wavelengths giving the highest proportion of *Z* isomer, reversibility of photoswitching, and *Z* → *E* relaxation half-lives *t*_1/2_ in aqueous media, were determined *in vitro*^21,33^ (key compounds in Fig. 2c, others in Table S1† and Fig. S1–S3†).

#### PSAR panel cell screening

**Fig. 3:**
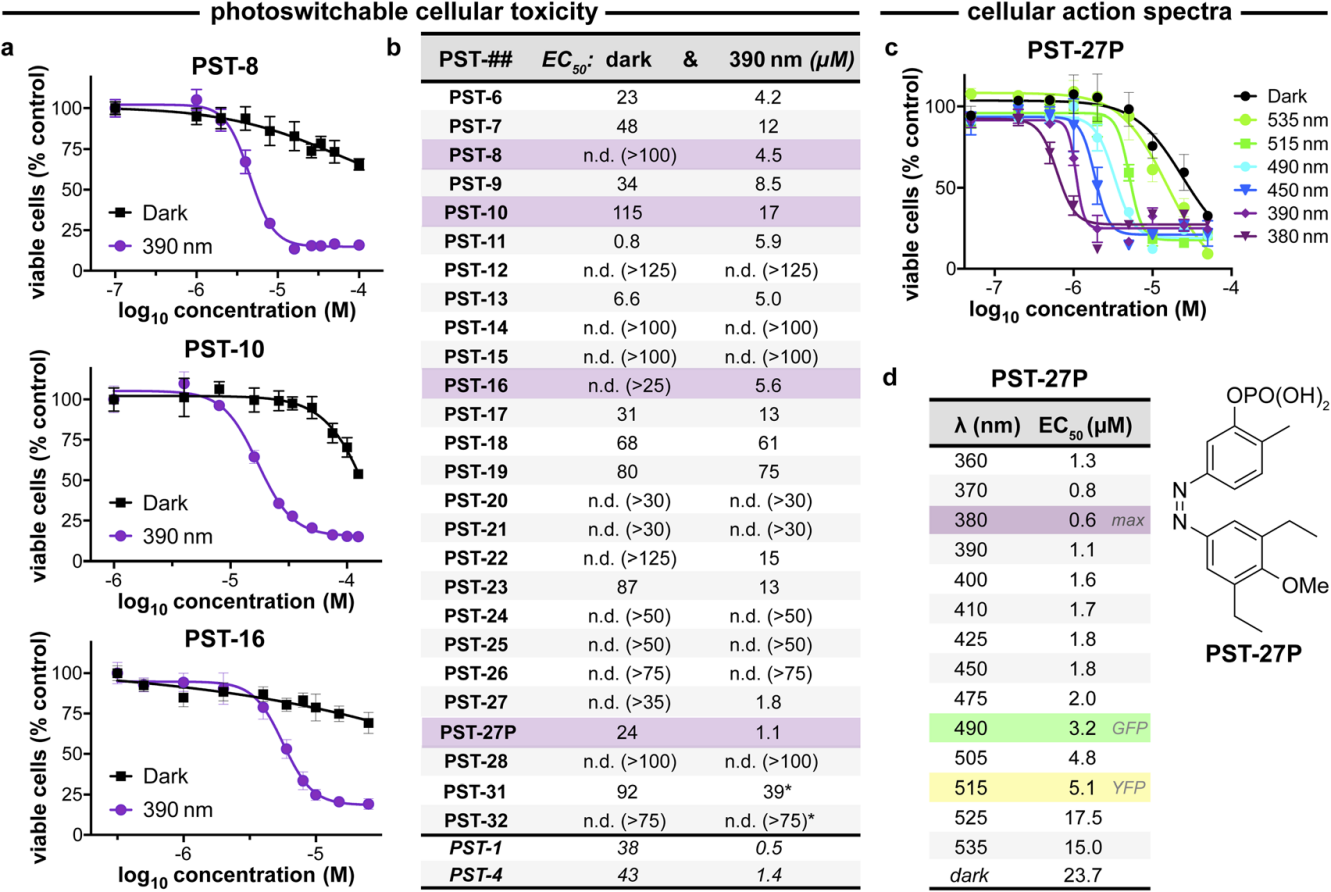
PST cytotoxicity can be optically controlled, with up to 20-fold enhancement from dark to lit conditions (MTT viability assay; HeLa cervical cancer cells; 46 h incubation). (a) Cell viability for selected PSTs. Data as mean ± SD. (b) EC_50_ values for all compounds (full data in Fig. S9;† *PST-31 irradiated at 370 nm and PST-32 at 425 nm). (c and d) Wavelength-dependent cell viability with PST-27P.

The compounds were screened for cytotoxicity in the HeLa cervical cancer cell line under dark *vs.* 390 nm-illuminated conditions (Fig. 3 and S9†).^21^ Our priority was not to maximise *Z*-isomer potency but rather to identify the structural tolerance for photoswitchably bioactive PSTs, that are potent as the *Z* isomer (illuminated) yet have low toxicity as the *E* isomer (dark): which is needed for photoswitchable tools. ESI Note 1† gives a full PSAR discussion. In brief however, for photoswitchability of bioactivity, the B ring tolerates small low-polarity groups at the *meta*-position (active bromo 8 but lower-potency alkoxy 9/10, inactive carboxylate 12); *ortho*-substituents are disfavoured although still have activity (6*vs.*7); and if small, double substitution is allowed (difluoro 16). Matching this, larger rings replacing the phenyl system that were reported for stilbenes^34–37^ were not tolerated in the azobenzenes (19–21); so their alluring red-shifted photoresponse (Fig. 2c) could not be harnessed. The A ring also tolerates small low-polarity *ortho*-substituents (22, 23); but neither isosteric nor smaller *meta* groups are tolerated if they are polar (24–26; Fig. 3b).

Within this SAR range, multiple tolerated modifications can be made simultaneously. For example, PST-27 was designed for blue-shifted photoresponse (less electron-rich), by replacing three methoxy groups with methyl/ethyls; this extra hydrophobicity required a B ring *meta* hydroxyl for solubility but became our most potently photoswitchably bioactive compound. This led us to prepare its highly water-soluble phosphate ester prodrug PST-27P for more reliable use in biology. We confirmed its strong wavelength-dependency of cytotoxicity over the entire range 360–535 nm (Fig. 3c and d), supporting our premise that PST analogues' toxicities are determined by their *in situ* photoconversion to the bioactive *cis* isomer at PSS under the wavelength applied. Being blue-shifted, PST-27P had twice as good orthogonality to GFP/YFP excitation wavelengths (6-to-8 fold lower activity than max, Fig. 3d) as compared to known PST-1 (3-to-4 fold), which may make it more useful in many practical settings.^21^ Unfortunately, the yet more blue-shifted PST-31 lost too much potency to be a useful reagent; but we expect other strategies for further blue-shifting could be successful (*e.g.* a 3,4,5-triethyl A ring) in future work.

### Confirmation of cellular mechanism of action

**Fig. 4:**
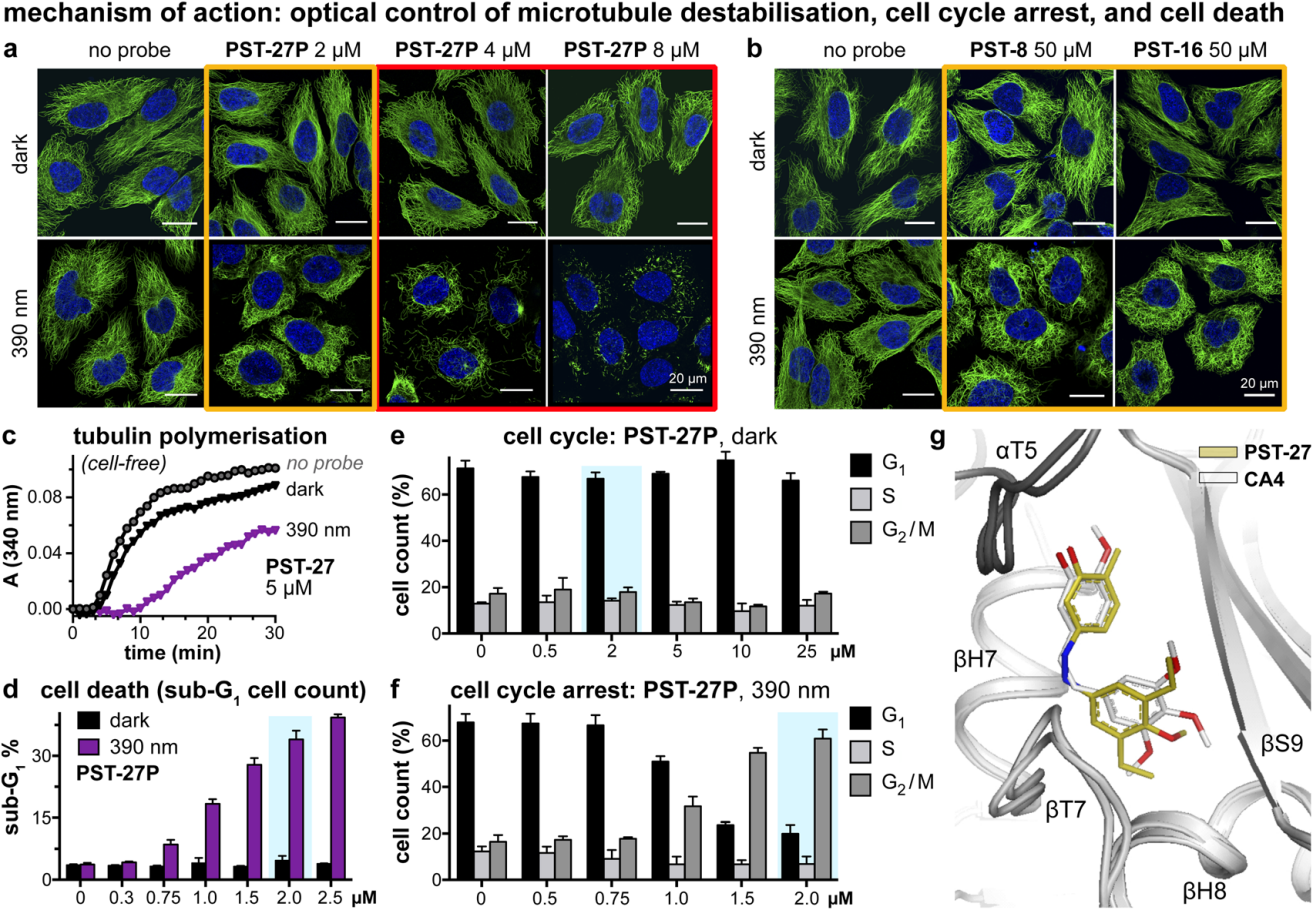
Mechanism of action assays show that *Z*-PSTs, but not *E*-PSTs, (a and b) induce microtubule network disorganisation [yellow frames] and depolymerisation [red frames], (c) inhibit polymerisation of purified tubulin protein, and (d–f) cause G_2_/M phase cell cycle arrest and cell death [sub-G_1_]. (g) X-ray crystal structure (PDB: 9F8G) of αβ-tubulin:DARPin D1 ^38^:*Z*-PST27 (olive carbons) superimposed on the previously reported tubulin:CA4 complex structure (white carbons; PDB 5LYJ;^32^ rmsd = 0.584 Å). ((a, b, d–f): in HeLa cells. a and b: 20 h treatment, α-tubulin immunostained with Alexa488 (green), DNA stained with DAPI (blue), scale bars 20 μm. (d–f): data as mean ± SD).

We next tested if the new PSTs'*cis* isomers retain tubulin inhibition as their primary mechanism of action (as for *Z*-PST-1–5).^21^ Indeed, PST-27P gave light-specific disruption of MT network organisation at its cell viability EC_50_, and gross MT depolymerisation above it (confocal imaging, Fig. 4a), as did other well-performing compounds (PST-8 and PST-16, Fig. 4b). PST-27 was progressed to further mechanistic tests, where it strongly inhibited microtubule assembly *in vitro* under 390 nm illumination, almost without perturbing polymerisation in the dark (Fig. 4c). Cellular readouts downstream from MT inhibition were consistent with this mechanism of action: PST-27P gave light-specific accumulation of a sub-G_1_ (dying) population (Fig. 4d) consistent with the viability assay, accompanying a distinctive light-specific G_2_/M phase arrest expected for cellular inhibition of tubulin dynamics (Fig. 4e and f). Finally, we obtained an X-ray crystal structure of the metastable *Z*-PST-27 bound to tubulin, confirming the same binding site and pose as the reference stilbene CA4 (Fig. 4g; PDB: 9F8G). Thus, we concluded that the *Z*-PST binding pose, SAR, and mechanism of action, are validated as matching known colchicine-site inhibitors.^30^

### Discovery of long-wavelength assisted photoswitching

#### Fluorophore conjugate design

**Fig. 5:**
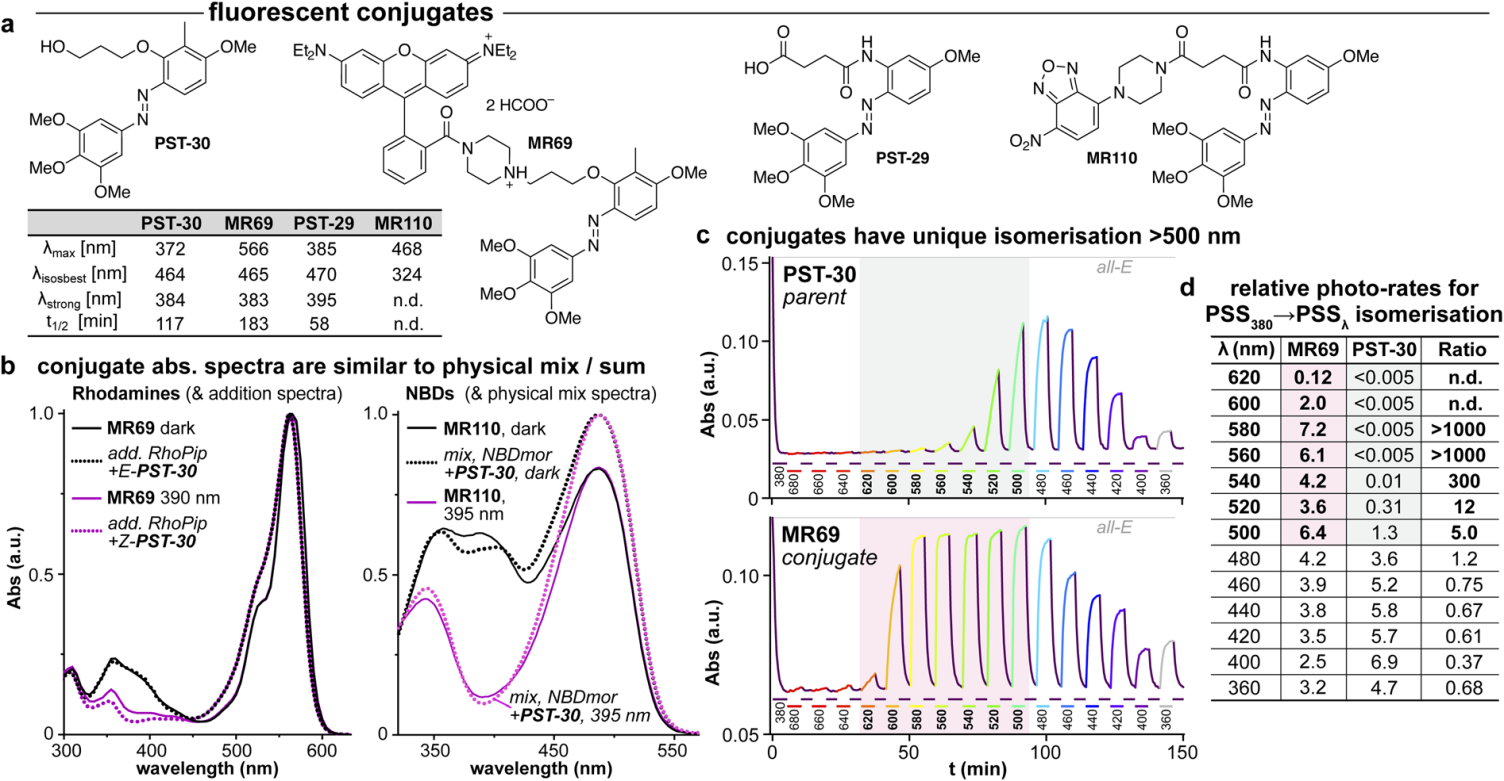
(a) Conjugates MR69 and MR110 have (b) similar absorption as the spectral sum of their components, but (c and d) undergo dramatically efficient and near-complete isomerisation when irradiated in the absorption region of their fluorophore motif (here, 520–600 nm). ((b): “add.” is the sum of isolated spectra, “mix” is the spectrum of a mixed solution. c: time-course of absorption at 370 nm (high: mostly-*E*; low: mostly-*Z*) during orthogonal illuminations alternating between 380 nm (deep purple, *E* → *Z*) and the indicated wavelengths (coloured as appropriate); grey/red boxes indicate the unique spectral region for assisted photoswitching with RhB. (c and d): monochromator light source with 5 nm FWHM used for switching. (d): “photo-rates” are normalised photoisomerisation conversion per unit light intensity (details at Fig. S4†).).

We had seen so far that larger groups are best tolerated in *ortho* at the A or B rings, and we aimed to exploit this to create photoswitchably tubulin-binding PST conjugates with functional cargos. Here, we tested fluorophore conjugates that might use resonant energy transfer (RET) to drive *E* ⇆ *Z* isomerisation (ESI Notes 2–3†), or else might be light-dependent MT imaging agents (*e.g.*, *E*: distributed, *Z*: MT-bound). In brief, we attached fluorophores *via* linkers to low steric demand ether and anilide groups at the *ortho* position of the A ring. We aimed for fluorophores with minimal absorption at 390 nm (Fig. S5a†), so that the conjugates might reach similarly *Z*-rich PSSs under UV illumination as the parent azobenzenes (later confirmed: Fig. 5b) and so might be bioactive against tubulin.

We chose a nitrobenzoxadiazole fluorophore (NBD, *ca.* 450/550 nm ex/em) whose <510 nm emission tail overlaps with the absorption of *E* & *Z*-PSTs (Fig. S1 and S2†), for conjugate MR110, expecting to drive some isomerisation by RET. For conjugate MR69,^18^ we instead chose an environment-independent rhodamine (RhB, *ca.* 530/560 nm ex/em; as a secondary amide to prevent forming a nonfluorescent spirolactam; Fig. 5a) where we expected that under excitation at *e.g.* 550 nm, its tiny or ∼zero emission overlap with azobenzene *Z* or *E* isomer absorption (<540 nm) ought to make RET isomerisation either *Z* → *E* selective but slow, or else impossible (Fig. S5b†).

#### Conjugates give assisted photoswitching

Our expectations were dramatically overturned, in that MR69 in particular was very efficiently isomerised to high-*E*-PSSs by exciting the RhB fluorophore (540–590 nm; Fig. S4†), although the azobenzene alone does not respond to these wavelengths (*cf.*Fig. 5d: PST-30 at ≥560 nm). In brief, key features of the conjugates include: (1) they have fast, efficient, *E* → *Z* photoswitching under UV light, as usual for azobenzenes (385 nm; Fig. 5b–d). (2) Exciting the fluorophore motif gives exceptionally efficient switching (Fig. 5c). MR69*Z* → *E* photoswitching above 550 nm approaches its PSS even more photon-efficiently than does its parent azobenzene under UV light (rough switching rates for >550 nm switching of MR69 and 400 nm switching of PST-30 are the same; Fig. 5d). (3) Conjugate switching by exciting the fluorophore can be exceptionally complete: MR69 reaches 94 ± 3% *E* isomer at PSS 554 nm (typical azobenzenes reach *ca.* 80% *E* with best-chosen cyan/green light wavelengths).

#### Assisted switching has promise to solve general problems in photopharmacology

The conjugates' performance is exciting. Their switching at long wavelengths can be more complete, and (more importantly) orders of magnitude more photon-efficient, than direct *Z* → *E* photoswitching of the parent azobenzene at its typical wavelengths (Fig. 5d: 20 to 400-fold faster switching of MR69 at 540–560 nm, than green light switching of PST-30 at 520–540 nm): yet, direct *E* → *Z* photoswitching is not much impaired. RhB conjugate *Z* → *E* switching operated efficiently even to ∼600 nm, which has much better penetration into tissue than wavelengths azobenzenes typically harness.

As the fluorophore motifs were capturing the energy used for this switching, we named this effect “assisted” isomerisation. Excitingly, this can address general challenges that have troubled photopharmacology: *e.g.*, how to achieve efficient isomerisation at long wavelengths, with a simple design for predictable photoresponse (here, at whatever wavelengths the fluorophore motif absorbs), without requiring drastic photoswitch redesign that blocks many substituent positions. For a brief discussion of the assisted switching design, rationale, and outcomes, see ESI Notes 2–5†; but since the mechanisms behind this effect turned out to be more complex than simple RET, we report their elucidation in two separate papers.^39,40^ Because azobenzenes are popular quenchers for fluorescent probes, we think it likely that such assisted switching will have been ongoing before: but it seems that it was not necessarily measured and reported as such; and as far as we are aware, such conjugates have never before been trialled for photoswitching in biology under single photon excitation at wavelengths longer than those at which azobenzenes usually absorb. Thus, we briefly examined their biological performance.

**Fig. 6:**
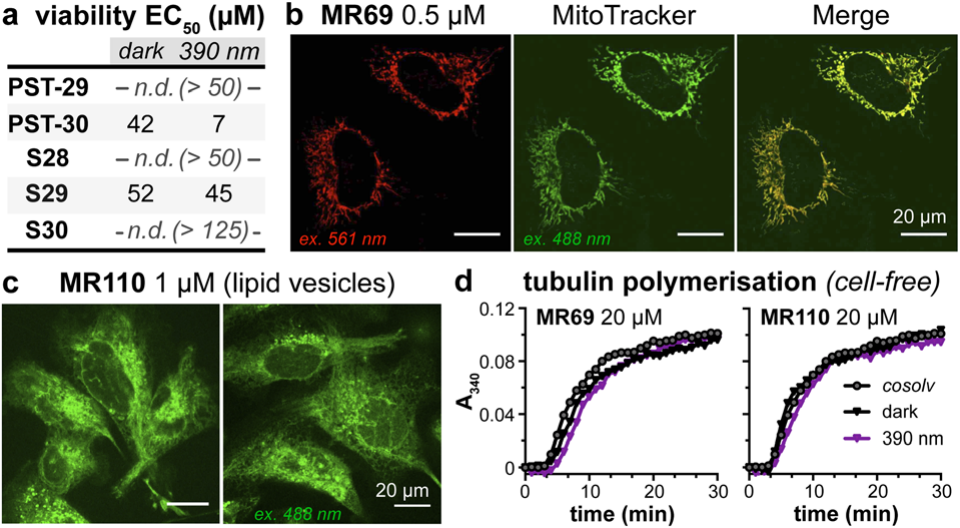
(a) Cytotoxicity of linker-PSTs (ESI Note 1†). (b and c) In cells, MR69 localises to mitochondria (*cf.* MitoTracker Green), and MR110 to lipid vesicles in the cytoplasm. ((a–c): HeLa cells). (d) Neither MR69 nor MR110 inhibit tubulin polymerisation.

As expected, installing linkers to give PST-29/30 lowered potency (ether PST-30 was still somewhat photoswitchably bioactive; Fig. 6a). Unfortunately, the cellular localisation of conjugates MR69 and MR110 was dominated by their fluorophores' intrinsic distribution: delocalised lipophilic cation MR69 to mitochondria (Fig. 6b),^41^ and hydrophobic NBD MR110 in lipid vesicles (Fig. 6c):^41^ thus, unsurprisingly, they did not stain MTs even after UV illumination. To test their target-binding, cell-free tubulin polymerisation assays were run at high^21^ concentrations, but did not show inhibition (Fig. 6d and e). We thus halted investigations, realising that substantial tuning would be needed for cellularly-useful assisted-switching tubulin photopharmaceuticals, regarding both the assisting chromophore and the photoswitchable ligand (discussion at Conclusions and in ESI Note 4†).

## Conclusions

### Motivation

PSTs were previously used in biology studies to disrupt MT polymerisation dynamics with spatiotemporal patterning, and so to apply spatiotemporally-defined gradients of antimitotic, anti-migratory, and cytotoxic effects in diverse cellular and *in vivo* models. Still, to unlock higher performance in cytoskeleton photopharmacology, designs with drastically improved photoswitching completeness and efficiency, higher potency, and/or that feature cargos allowing more elaborate chemical biology applications, are needed.

### Photo-SAR

This photo-SAR study identified tolerances for modifications to the PST scaffold which can now be used to adapt them for these needs: *e.g.*, installing response to specific wavelengths, or avoiding it (wavelength orthogonality, *e.g.*, blue-shifted PST-27); or tuning spontaneous relaxation speeds and polarity (Fig. 2); which can be performed while keeping the *Z*-specific tubulin-binding cellular mechanism of action (Fig. 3 and 4). This will support developing higher-performance PST reagents for microtubule studies; and we believe that even more blue-shifted (more GFP-orthogonal) reagents will prove to be the most valuable of those reagents.

### Assisted switching with colchicinoids

We also found positions that may be suitable for attaching payloads, and used these to create fluorescent conjugates. We found these offered powerful solutions to the longstanding practical challenges of redshifting and high-completion isomerisation, which have hindered photopharmacology from *in vivo* application. The elucidation of the assisted switching mechanism is being reported elsewhere.^39,40^ We think it likely that assisted switching will not actually prove impactful within the PST series: because PSTs are stoichiometric binders for a high-expression target (tubulin: *ca.* 10 μM in the cytosol^42^), so tubulin-inhibitory activity requires rather high concentrations of conjugates in the cytosol. Since most fluorophores are larger than the (moderate-potency) PST pharmacophore, we suspect that inevitable losses of potency upon payload attachment will be complicated by fluorophore biodistribution effects such that cellular applications as photoswitchably-binding colchicinoids are blocked (ESI Note 4†).

### Assisted switching for other reagents and targets

However, we see strong scope for applying assisted switching elsewhere, *e.g.* with (a) inherently high-potency scaffolds, since these may better tolerate fluorophore attachment; (b) reagents where incomplete *Z* → *E* switch-off has limited performance so far; (c) reagents being developed for use in deeper tissues, or in other optically dense or scattering systems including in materials, where long-wavelength response can be a great advantage. Although we will report such applications from our work in due course,^39,40^ we believe that the discovery of this assisted switching effect will have significantly broader impact than our photopharmacology-oriented investigations: and we look forward to its adoption by the chemical community.

## Abbreviations

MT: Microtubule
PSAR: Photoresponse-structure–activity relationship
PSS: Photostationary state [*E*:*Z* photoequilibrium]
PST: Photostatin [azobenzene analogues of the tubulin polymerisation inhibitor combretastatin A-4]
SAR: Structure–activity relationship
RET: Resonant energy transfer
RFLM: Reconstruction-free lensless microscopy

## Data availability

All data supporting this article have been included as part of the ESI (ESI Notes 1–5, methods, procedures, characterisations). † Crystallographic data for the αβ-tubulin:DARPin D1:*Z*-PST27 complex has been deposited at the PDB under accession number 9F8G and can be obtained from https://doi.org/10.2210/pdb9F8G/pdb.

## Author contributions

M. R., A. M.-D., K. K., A. R., and A. A. designed compounds, performed synthesis and analysis; M. G. performed and supervised cell-free and cellular assays; M. W. performed protein production, crystallization, compound soaking, X-ray data collection, processing, and structural refinement, as supervised by M. O. S.; L. O. R. and Y. K. performed cellular assays; K. S., A. J., and V. S. built the illuminating RFLM and performed online monitoring assays, as designed and supervised by P. P.; B. B. performed analysis; A. B. and T. S. performed synthesis; O. T.-S. designed the study and the targets, performed and supervised experiments, and wrote the manuscript with contributions from all authors.

## Conflicts of interest

K. S., A. J., and P. P. are employees of PHIO Scientific GmbH. There are no conflicts of interest.

## Supplementary Material

SC-015-D4SC03072A-s001
