# Supplementary material for: A photo-SAR study of photoswitchable azobenzene tubulin-inhibiting antimitotics identifying a general method for near-quantitative photocontrol
Source: Chem Sci. 2024 Jul 2;15(31):12301–9. doi: 10.1039/d4sc03072a (PMC11304547; doi:10.1039/d4sc03072a)
Supplement: SC-015-D4SC03072A-s001 [file SC-015-D4SC03072A-s001.pdf]

Electronic Supplementary Material (ESI) for Chemical Science.
This journal is © The Royal Society of Chemistry 2024

Supporting Information to

A photo-SAR study of photoswitchable azobenzene tubulin-inhibiting antimitotics identifying a general method for near-quantitative photocontrol

Martin Reynders,[a] Małgorzata Garścia,[a] Adrian Müller-Deku,[a] Maximilian Wranik,[b] Kristina Krauskopf,[a] Luis de la Osa de la Rosa,[a] Konstantin Schaffer,[c,d] Anna Jötten,[c,d] Alexander Rode,[a] Valentin Stierle,[c] Yvonne Kraus,[a] Benedikt Baumgartner,[a] Ahmed Ali,[a] Andrei Bubeneck,[a] Trina Seal,[a] Michel O. Steinmetz,[b,e] Philipp Paulitschke [c,d] and Oliver Thorn-Seshold *[a]

[a] Faculty of Chemistry and Pharmacy, Ludwig-Maximilians-University Munich, Munich 81377, Germany. [b] Laboratory of Biomolecular Research, Division of Biology and Chemistry, Paul Scherrer Institut, Villigen 5232, Switzerland. [c] Faculty of Physics and Center for NanoScience (CeNS), Ludwig-Maximilians-University Munich, Munich 80539, Germany. [d] PHIO scientific GmbH, Munich 81371, Germany. [e] Biozentrum, University of Basel, Basel 4056, Switzerland.

Correspondence and requests for material: oliver.thorn-seshold@cup.lmu.de

Table of Contents

Supporting Notes ... **2**
- Supporting Note 1: Biological Background and PSAR Discussion (Fig 2-3) ... 2
- Supporting Note 2: Rationale for developing assisted photoswitching ... 4
- Supporting Note 3: Design of our assisted photoswitching conjugates ... 6
- Supporting Note 4: Assisted Photoswitching: Experimental Features ... 8
- Supporting Note 5: Assisted Photoswitching Bioactives: future directions ... 8

Part A: Chemical Synthesis and Characterisation ... **9**
- Conventions ... 9
- Standard Procedures ... 11
- Syntheses ... 12

Part B: Photocharacterisation in vitro ... **45**
- Spectrophotometry equipment ... 45
- 1: trans- and cis- absorption spectra, and PSS φ(λ) ... 45
- 2: Thermal cis→trans reversion halflives ... 47
- 3: Reversible trans↔cis photoisomerisation of non-conjugated PSTs ... 48
- 4: Conjugates MR69 / MR110: absorption and E↔Z photoisomerisation ... 49
- 5: Conjugates MR69 / MR110: fluorescence ... 49

Part C: Cell Biology ... **55**
1. In vitro tubulin polymerisation assay (Fig 4cd) ... 55
2. Cell culture under controlled light conditions (Fig 3-4) ... 55
3. MTT viability assay (Fig 3) ... 55
4. Confocal microscopy (fixed samples) (Fig 4ab) ... 58
5. Nuclear fragmentation and cell cycle analysis (Fig 4ef) ... 58
6. Live cell imaging (Fig 6bc) ... 58
7. Growth rate monitoring (RFLM) (Fig 1) ... 59

Part D: Structure of *Z*-PST-27 bound to tubulin-DARPin D1 complex ... **60**
1. Protein Production, Crystallisation, and Soaking ... 60
2. X-ray Diffraction Data Collection, Processing, and Refinement ... 61

Appendix: NMR Spectra ... **62**

Supplemental References ... **96**

Supporting Notes

Supporting Note 1: Biological Background and PSAR Discussion (Fig 2-3)

Background *(expanded from the Introduction)*: Microtubules (MTs) are major cytoskeletal components whose structure and dynamic remodelling are vital for diverse processes, from intracellular transport and cellular motility to the form and function of the mitotic spindle during proliferation[1].The protein tubulin, which forms MTs, is highly conserved across all eukaryotes, and attempts to genetically modify the tubulin protein for direct stimulus-responsive control over its function have not been successful. Although genetic tools are recently being developed,[2–4] small molecule inhibitors that interfere with MT structure and dynamics remain indispensable as research tools for manipulating cytoskeleton biology.[5] In medicine too, MT inhibitors are successful antimitotic chemotherapeutics, particularly the taxane and vinca site binders;[6,7] while inhibitors binding at the colchicine site, including colchicine, combretastatin A-4 (**CA4**, **Fig 1a**), and their closely isosteric analogues, have also entered late-stage clinical trials.[8] However, it is challenging or impossible to use these small molecule drugs study particular roles of MTs – e.g., in specific cell regions, cell populations or tissues, and at precisely defined times – since they cannot be either spatially or temporally directed.[9] Indeed, many important aspects of cytoskeleton biology remain poorly understood. Developing spatiotemporally-targetable small molecule MT inhibitors has thus been an important goal, and optical control strategies have been prioritised since light can be applied with high spatiotemporal precision even *in vivo*.[10,11] Irreversibly photo-triggered strategies were initially developed, including photobleachable and photouncageable inhibitors, or using practically irreversible C=C double bond photoisomerisation.[12–17] However, irreversibly triggered approaches cannot overcome the diffusion of the active drug over time, so they suffer conceptually from limited spatiotemporal resolution.

PSAR of PST derivatives: We found that the **B ring** tolerates some steric presence at the *ortho*-position, with bromo **PST-6** having better photoswitchable bioactivity than methoxy **PST-7**. The *meta* position also supports small substitutions, with *meta*-bromo **PST-8** having more potently photoswitchable bioactivity than the larger *meta*-methoxy **PST-9** or benzodioxolo **PST-10**. Increasing polarity to the *meta*-aldehyde **PST-11** was inconclusive since its toxicity in the *trans* form, which we attribute to reactivity with cellular components, prevents analysis in this series, but a larger polar carboxylate as in **PST-12** abrogated all potency. Taken together, these results confirm the established requirement for small uncharged groups in north ring *ortho*- and *meta*-positions, but shows there is only a moderate loss of potency with medium-sized replacements. We also examined three compounds with larger and more polar substituents in the *para* position, **PST-13** with *para*-SMe, **PST-14** with *para*-S(O)Me, and **PST-15** with *para*-S(O_2)Me. Of these only the smallest and least polar, **PST-13**, showed significant *apparent* bioactivity but this should be seen cautiously as its switchability of bioactivity was poor, which we interpret as reflecting weak solubility that causes significant aggregation toxicity (i.e. a tubulin-independent mechanism). Double substitutions on the north ring were also possible, as in the highly photoswitchably bioactive difluoro **PST-16**. The larger *ortho*-fluoro-*meta*-nitro **PST-17** was as expected less active, but this also suggested that the nitro group was photostable and tolerated in our cell assays, which recommends further exploration of this powerful auxochrome for wavelength-shifted designs.

Satisfactory ring systems to replace the phenyl north ring were not found. Tropolone **PST-18** was first designed as a relatively isosteric replacement, which can adopt a *cis*-geometry only marginally different from that of colchicine. However, its absorption spectrum had similar

features to typical *para*-phenolic azobenzenes, and while it was photoswitchable in DMSO, photoswitching was not observed in aqueous solution, presumably due to fast relaxation to the *E* isomer; it showed no significant or photoswitchable bioactivity. Bicyclic north rings have been reported for moderate to potent colchicine site inhibitors[18–21], so we also examined the poorly soluble naphthalene **PST-19**, previously reported by Friedman[22]. To study whether improving its cytosolic availability could restore its bioactivity, we also synthesised the more polar quinoline **PST-20** and quinoline ester **PST-21** where the nitrogen can act as a hydrogen bond acceptor (similarly to **PST-1** and **PST-2**). While these derivatives had red-shifted absorption spectra compared to the panel of **PSTs** (**Fig 2c, Fig S1-2, Table S1**), they showed no satisfactory photoswitchable bioactivity (as Brittain[23] has observed for **PST-19**), so we concluded that the steric demand of these bicycles attached at the 1-position cannot easily be accommodated at the binding site.

At the south ring, we also observed tolerance for steric presence at the *ortho*-position, as *ortho*-bromo **PST-22** and *ortho*-methoxy **PST-23** had potently photoswitchable bioactivity. Increasing *meta* steric presence and polarity in a single group, as in **PST-24/25**, was however not tolerated, nor was decreasing its size to the small polar hydroxyl group as in **PST-26**.

Simultaneous modifications to both rings within the scope of this SAR are **also** possible without losing bioactivity. **PST-27** has photoswitchable bioactivity almost identical to that seen for **PST-1** or **PST-4**, despite replacing the A-ring *para-* and the two B-ring *meta*-methoxy groups - which primarily play low-polarity space-filling roles - with still less polar alkyl groups, and simultaneously reducing the steric presence of the north ring *para* group by replacing it with methyl (we introduced a north ring *meta* hydroxyl to maintain satisfactory solubility for this otherwise apolar compound). As our most potent and photoswitchably bioactive compound in this set, we prepared its highly water-soluble phosphate ester prodrug **PST-27P** to use in further studies, which allowed us to avoid the effects of cosolvents as well as of solubility-related bioactive effects which could both complicate studies of its mechanism of action (see below). We confirmed its strong wavelength-dependency of cytotoxicity over the entire range 360-535 nm (**Fig 3c-3d**), supporting our understanding that such **PST** analogues' toxicities are still primarily determined by their photoconversion to the *cis* isomer, which is substantially more bioactive than the *trans*. **PST-27P** displays improved orthogonality to GFP/YFP excitation wavelengths compared to **PST-1** (**Fig 3d**). Finally, simultaneously installing two moderately potency-reducing modifications, as in **PST-28** (south *ortho*-OMe and north *meta*-OMe), was confirmed to abolish bioactivity within the series. In an attempt to strongly shift the λ_{max} of the *E* isomer, we prepared **PST-31** as the most blue-shifted compound in this series and **PST-32** with a strong bathochromic shift. While **PST-31** retained some photoswitchable bioactivity, **PST-32** was inactive, possibly due to the high steric remand or the absence of significant photoswitching in aqueous solution. This screening suggests that the SAR features have additive roles in the bioactivity of these azobenzene-based photoswitchable cytotoxins, which promotes the rational design of bioactive analogues bearing a range of desirable substituents.

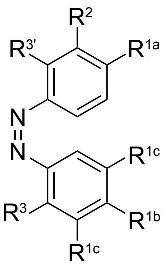

<u>SAR Summary</u>: Data for compound cytotoxicity is summarised in **Fig 3b**; in brief, as shown at right, groups R^1 should be small apolar (OMe, Et > Me, Pr, OEt; greater penalty for R^{1c} than R^{1b} than R^{1a}); R^2 should be small polar (OH, F, NH_2 > H >> Me, OMe, Et); R^3 and $R^{3'}$ ideally H, but larger substituents tolerated (greater penalty for R^3 than $R^{3'}$). We considered that the good match of the SAR arising from this study with SAR previously published for stilbenoid combretastatins[24] supports their sharing tubulin as the primary biological target of the **PSTs'** *cis* forms, and we performed selected evaluations of this mechanism of action,

primarily focusing on **PST-27** but also assaying lower-potency compounds with reasonable photoswitchability of bioactivity such as **PST-8** and **PST-16**. While their light-specific disruption of microtubule network structure was evident (**Fig 4b**), higher concentrations were not accessible due to their poor solubility, so we focussed on **PST-27** for further experiments.

Lack of activity for **PST** precursors **S28**, **S29** and **S30** (**Fig 6**)

We had expected that **S28**, **S29** and **S30** would be largely biologically inactive: since presumably their *ortho*-OH and –NH_2 groups would drive azo-hydrazone tautomerism allowing fast *cis* to *trans* relaxation[25] (typically < 1 ms), and indeed, we could observe no photoisomerisation of these compounds in aqueous media; and because only the *cis* isomer of colchicinoids can bind to tubulin,[24] we believe that the absence of *cis* in the aqueous environment of the cytosol where tubulin resides ought to result in non-toxicity *unless either (a) the illumination is being performed in a way that generates phototoxicity (potentially mediated by the non-tubulin-binding compound), or, (b) the trans-isomer in question has other biological targets than tubulin*. We note that this contrasts to Brittain's interpretation of similarly-structured azobenzenes with a *para*-OH group, whose photoisomerisation was likewise not observable, but which were reported to show up to two orders of magnitude greater toxicity *in cellulo* when UV-illuminated for 10 s every 30 min[23] (under illumination conditions that are not comparable to ours) which was then interpreted as light-dependent tubulin-binding-induced cytotoxicity. In this study, we would not have interpreted such a result as indicating tubulin-based activity: rather, we consider that confirming "no photoswitchable toxicity" for these essentially not-bulk-photoswitchable compounds is a useful control result, as it reveals the absence of e.g. nonspecific photo- or aggregation toxicity in our photo-assay.

Supporting Note 2: Rationale for developing assisted photoswitching

Generating bioactive *cis*-**PSTs** by direct UV *trans* to *cis* photoisomerisation *in cellulo* is relatively photon-efficient and is close to complete (~96% for **PST-1** under 385 nm, with ~90% under 405 nm), but the *cis* to *trans* back-photoswitching is typically 30-fold less photon-efficient as well substantially less complete (~19% **PST-1** remains under 514 nm, and ~12% under 532 nm).[26] Improving the completeness and/or efficiency of photoswitching, particularly back-switching, offers several conceptual benefits:

Spatiotemporal modulation of biological target activity depends on establishing a high ratio between the concentrations of the bioactive *cis* form in "UV-illuminated" versus "back-switched" spatial/temporal regions. The greater is this ratio, the more defined will be the difference in biological activity between the two zones, with the maximum possible ratio being set by the maximum fold change in e.g. [*cis*-**PST**] that can be applied in practice by photoswitching. With **PST-1**, this maximum fold change is typically around ~4-fold (under 405/514 nm photocontrol on the microscope stage). Although that value is superior to values for most reported photopharmaceuticals, and despite several successful applications of **PSTs** in biology[27–29], it nonetheless severely limits the magnitude of the difference in biological effect that can be patterned onto living systems. In practice, the user must also ensure that the total applied concentration of **PST** is such that this 4-fold window covers *cis*-**PST** concentrations ranging from non/weakly-inhibiting through to strongly-inhibiting, otherwise the bioactivity difference applied is substantially smaller than theoretically possible.

Since the *trans* isomer is biologically inactive against tubulin, linearly increasing the completeness of *cis* to *trans* back-photoswitching would *non*-linearly (1) improve the bioactivity fold change that can be applied by photoswitching, and (2) make it substantially easier to apply **PSTs** in practice since with a wider switching window, the tolerance for different total

concentrations is higher. For example: a **PST** with backswitching completeness improved from 81% to 99% would allow spatiotemporal regions to be optically patterned with a fold difference of the bioactive *cis*-**PST** concentration improved from 4-fold up to >90-fold. That could open up a new scope of applications for precision photopharmacology by totally blocking/totally allowing biological function in neighbouring regions, rather than the partial block/allowance that is possible with a 4-fold change. Since MT dynamics show low sensitivity to inhibitor concentration up to a threshold concentration, then typically slow down and are abolished within a ~ten-fold increase in inhibitor concentration [strong non-linearity of response], such a 90-fold-change reagent would be able to be used at any total concentration between 10-100× the threshold concentration while still ensuring full photoswitching between entirely non-inhibited and entirely inhibited states; whereas at its optimum working concentration, the 4-fold-change switch can only ensure a 50% change of inhibitory activity, and any deviation from the optimum will reduce the change of inhibition still further from this.

Additional benefits could also be obtained from a linear improvement in back-switching photon efficiency that would give a linear decrease in the illumination time and energy delivery needed for back-switching, and so (3) a linear increase in the time precision of patterning zones where MT polymerisation is allowed, as well as (4) a linear reduction of photobleaching/phototoxicity during patterning. This initial research into assisted photoswitches aimed to tackle all four of these points, by addressing both completeness and efficiency of back-switching.

(It should also be noted that another long-term potential benefit for assisted photoswitching is that it might be possible to apply it in deep tissue, towards *in vivo* applications of photopharmacology other than in the optically transparent setting of the eye. A long-wavelength-photocontrolled inhibitor might be useful for preclinical studies into the therapeutic benefit of avoiding side-effects by localising drug activity; or it might even potentially be developed as a drug candidate for special settings.)

Differentiation from known assisted intramolecular (triplet photoredox-induced) switching via Ir(III) complexes: Here, we required a non-toxic and non-phototoxic antenna. Cyclometallated Ir(III) complexes are highly phototoxic *in cellulo* (Hecht has noted the strong quenching of photoredox isomerisation by dissolved O_2[30]), acting as potent photodynamic therapy agents[31]; they can also cause uptake-dependent cell toxicity without illumination even at low µM concentrations[32]. Secondly, we aimed to install near-complete backswitching. The Ir(III) complexes require UV/blue illumination, which overlaps strongly with azobenzene absorbance spectra, and competitive direct azobenzene excitation causes its substantial reduction in isomerisation completeness; which can be compared to the non-overlapping RhB band here. Lastly, we desired an antenna that would be synthetically accessible, with easy purification and handling by basic synthetic methods, ideally an all-organic moiety that is known to be biologically tolerated. We consider that the synthetic challenges of conjugating metal complexes as well as general toxic effects from their [photo]catalytic activity or metabolic breakdown do not easily suit them to this role. However, these considerations seem to align with organic fluorophores used in biology, that are not employed as photosensitisers.

Regarding other strategies for red-shifting azobenzene photoresponse beyond the UV/blue: Push-pull or electron donating substituent patterns deliver azobenzenes where *cis* to *trans* isomerisation is possible at longer wavelengths than *trans* to *cis* (typically, up to 560 nm), but these compounds have thermal halflives of seconds or less. The other main strategies for red-shifting azobenzenes (tetra-*ortho*-substitutions that use steric clash to twist the *trans* geometry, tetra-*ortho*-fluoro substitutions modifying the *n* orbital energy, and stabilised azonium switches[33–35]) invert the order of wavelengths for photocontrol, so requiring UV/blue light for

cis to trans isomerisation. Bridged azobenzenes[36] are an exception, in that the cis isomer is the thermodynamic ground state: but the requirement for UV/blue light to generate the *trans* form, and their short halflifes (minutes), are other obstacles to their *in vivo* use; and particularly for our case, their steric blocking of key *ortho* positions can prevent binding.

Supporting Note 3: Design of our assisted photoswitching conjugates

Our SAR analysis informed our design for photoswitchable conjugates one day intended to bear functional cargo (eg. employing bioorthogonal ligation groups or tight binders, such as azide, alkyne, biotin or benzylguanine units), aiming towards high-spatiotemporal precision chemical biology studies. Here, valuable conjugates should show *cis*-selective binding to tubulin when applied at concentrations well below the threshold for toxicity.

We here report initial work on fluorophore-bearing "assisted photoswitching" conjugates, that both allow sensitive fluorescent readout of their biolocalisation (as model systems for future conjugates), and also display an under-studied mechanism by which excitation of the fluorophore “antenna” can assist the photoswitch’s isomerisation to address two challenges that face photopharmaceuticals in general: ***Aim 1****:* allowing more *in vivo* compatible patterning of bioactivity, ie. with more photon-efficient and substantially red-shifted photocontrol, though with minimal modification to the photoswitch-pharmacophore nucleus. ***Aim 2****:* allowing higher spatiotemporal precision *in cellulo* work, by improving the isomerisation completeness especially for *cis* to *trans* isomerisation, while retaining photon efficiency.

Aim 1, often summarised as “redshifting”, is widely pursued in the hope of enabling photopharmacology applications in deep tissue *in vivo*[37] and should also reduce phototoxicity. Here our design was to use a fluorophore with a high absorption coefficient to capture extra photons in a wavelength region where the azobenzene absorbs moderately at most, hoping that the combination of energy transfer plus direct azobenzene absorption would increase the overall speed of generating a *threshold level* of the bioactive *cis* isomer. Intra- and intermolecular energy transfer from antennae to photoswitch chromophores which perform fast energy dissipation have been extensively demonstrated and reviewed,[38] and have long been used for RET fluorescence quenching in probes where a single- or two-photon antenna donor fluorophore is conditionally linked to or separated from a photochromic quencher (often push-pull classical or heteroaryl azobenzenes, e.g. DABCYL, Black Hole Quenchers, etc)[39]. However, that body of research has almost exclusively dealt with the modulation of the donor's fluorescence properties, while the consequences of those interactions on the photochromic moiety are barely reported. Branda’s reports of a porphyrin-diarylethene conjugate designed to allow photoswitching to modulate luminescence[40,41] in which prolonged porphyrin excitation led to ring-opening of the diarylethene are, to our knowledge, one of the earliest relevant reports on the effects of intramolecular energy/electron transfer on the photoswitch. Studies aiming to use indirect excitation mechanisms to accumulate bistable photoswitches to useful PSSs are more recent. Two-photon antenna excitation and RET to a covalently attached azobenzene has been used by Gorostiza to drive 58% *trans* to *cis* isomerisation of a ligand for an engineered glutamate receptor channel[42], and Hecht used an antenna-azobenzene conjugate to drive *cis* to *trans* reversion with single photon (76%) as well as two-photon excitation (65%; both in organic solvent);[43] other strategies to modify photoswitching performance, such as sensitised access to the triplet manifold, have been addressed elsewhere[44] and are comprehensively reviewed in our two accompanying papers[45,46].

With little data to guide a choice of antenna for single-photon biological work, we thus set the requirement that for one conjugate, its emission would have to overlap substantially with the

absorption of the *trans* for good energy transfer, though unavoidably for **PSTs** this also overlaps with the *cis* and therefore may reduce the PSS compared to that obtainable by direct azobenzene excitation only. We selected excitation at 460 nm as our target: a compromise between excitation at biologically compatible and well-transmitted longer wavelengths, and shorter wavelengths where absorption of the typical **PSTs** is stronger (increases steadily from ε_{trans}~1500 $M^{-1}cm^{-1}$ and $\varepsilon_{trans/}\varepsilon_{cis}$~0.5 as wavelength decreases). We therefore designed nitrobenzoxadiazole (NBD) conjugate **MR110**, whose NBD fluorophore can be stimulated in the blue/cyan (440-490 nm) and emits broadly at >505 nm, in a range overlapping moderately with both isomers' absorption (**Fig 5b, Fig S5**). We expected that 460 nm excitation of **MR110** would more quickly reach a mixed PSS than direct azobenzene excitation alone (**PST-29** has ~40% *cis* at PSS under 460 nm).

<u>Aim 2</u> is less widely addressed in current photopharmacology, but we consider it to be a more pressing need for *in vitro* uses of photopharmacology tools by the biological research community; we discuss this at the start of **Supporting Note 2**.

We originally expected that one possibility for near-quantitative backswitching would be to employ an energy transfer donor excited at a wavelength where the *trans*-azobenzene has vanishing absorbance but above which the *cis* isomer still has small but measurable absorbance (favouring near-unidirectional isomerisation by both direct and RET excitation), and which features only a small Stokes shift so that the overlap integral is maximised (favouring greater RET efficiency).

A second possibility would be to employ photoredox-induced isomerisation, which offers an alternative mechanism for antenna conjugates destined for biological use to undergo unidirectional and potentially high-yielding backswitching. The first photoswitch-focussed studies of photoredox-induced unidirectional reversion were published during the preparation of this research[47] and counterpointed[48] the results we obtained here. A full discussion is given in our parallel paper[45], but in brief, Ir(III) complexes mixed with photoswitches were first shown to undergo intermolecular photoredox-based unidirectional isomerisation by Nam (diarylethene ring-opening, quantitative)[49] and by Hecht (azobenzene *cis* to *trans* isomerisation, 76%[48]); Hecht subsequently delineated the relative rate enhancement when an intramolecular conjugate was designed (azobenzene, 76% when degassed[30]). While this research was not directed towards photopharmacology, those approaches may not succeed in biology due to several constraints which in our parallel paper[45] we aimed to address by considering organic fluorophores used in biology, that are not employed as photosensitisers but which are related to photooxidant metal complexes.

Taken together, primarily for reasons of spectral overlap, here we selected a Rhodamine B fluorophore with excitation and emission maxima λ_{ex} ~550 nm /λ_{em} ~570 nm, hoping that the excited state could perform selective RET to the *cis*, giving near-quantitative backswitching of the conjugate **MR69**. We reasoned that if unidirectional photoredox-back-switching were also present, it would also improve the speed and completeness of back-switching. In the course of this work, it was found that the energy transfer from the antenna to the azobenzene seen here was likely occurring by a more complicated mechanism involving triplet states; the separate paper exploring this mechanism is being preprinted in parallel to this manuscript.[46]

<u>Bioactivity:</u> To try to design conjugates that could retain bioactivity in *cis*, we selected low steric demand ether and anilide groups at the *ortho* position of the B ring for attaching the linkers to the azobenzene, guided by the combretastatin-tubulin crystal structure[50] and by our SAR studies showing that the B ring better tolerates sterically demanding groups.[51]

We had also selected piperazine as the proximal connection to the fluorophores hoping to reduce the chance of intramolecular fluorophore-photoswitch steric interactions that might alter their photophysical properties in an environment-dependent manner, although in retrospect this assumption turned out to have been naive. Synthesis of the NBD-azobenzene conjugate **MR110** was achieved through acylation of aniline **S29** to reach linker anilide **PST-29**, and coupling to an amine-terminated NBD giving **MR110**. Although **PST-29** showed very weak *Z*-cytotoxicity suggesting, in the best case, only very poor binding to tubulin, this was not considered predictive for **MR110** since **PST-29**'s free carboxylate could disfavour binding at this typically hydrophobic pocket. We noted in passing that **PST** precursors **S28**, **S29** and **S30**, which fit the steric SAR requirements outlined above, were nonetheless biologically inactive even under illumination (discussion in **Supporting Information Part C**). Rhodamine-azobenzene conjugate **MR69** was tackled by alkylating phenol **S30** to deliver **PST-30**, which was halogenated and alkylated onto rhodamine B-piperazine[52] to give **MR69** in poor but isolable yield. The steric demand of the ether linkage in **MR69** seemed well tolerated since MTT toxicity evaluation of **PST-30** had photoswitchable bioactivity (**Fig 6a**).

Supporting Note 4: Assisted Photoswitching: Experimental Features

The conjugates **MR69** and **MR110** both retain fast, near-complete, single-photon *trans* to *cis* photoswitching driven by direct UV absorption by the azobenzene (as normal azobenzenes). However, they feature more complete and more rapid *cis* to *trans* isomerisation which can be driven by photoexcitation of the fluorophore moiety (**Fig 5**, **Fig S6**, **Tables S2**-**S3**).

MR69 in particular shows up to >100× faster single-photon *cis* to *trans* backswitching compared to its switch moiety **PST-30**, and this back-switching is nearly complete: which is outstanding performance compared to typical azobenzenes; the backswitching even proceeds acceptably quickly at wavelengths up to 600 nm which have excellent penetration deep into living tissue. This fits the requirements of **<u>Aim 2</u>**: with its ~40% *trans* to *cis* isomerisation under 405 nm, but 94% complete (as well as *more* photon-efficient) back-isomerisation under 561 nm, it can be used under standard confocal microscopy conditions to perform single-photon optical patterning with >10-fold difference in concentration – an unprecedented ratio for azobenzene photopharmacology.

MR110, with greater overlap between the excitation bands of photoswitch and fluorophore, showed a smaller change of PSS from its azobenzene moiety (**Fig S6**, **Tables S2-S3**). The importance of covalent attachment of the antenna and switch for modification of PSS, isomerisation speed and wavelength response was confirmed for **MR110** since the physical mix behaves almost identically regarding photoswitching as does a superposition of the isolated azobenzene and fluorophore spectra: i.e. no intermolecular assisted switching (**Fig S6**). The fluorescence properties (as well as thermal relaxation speeds) of the conjugates were closely similar those of the physical mixes and free moieties (**Fig 5a, Fig S5**). The absence of strong fluorescence quenching in the conjugates indicates that the efficiency of the indirect isomerisation process could probably be increased (by modification of antenna, linker and/or photoswitch); and this is a topic of our ongoing research.

Supporting Note 5: Assisted Photoswitching Bioactives: future directions

Although conjugates are promising for their fast, optionally quantitative, long-wavelength single-photon photoswitching, further modifications to the "antenna" strategy are needed to retain bioactivity, and so enable cellular applications to protein control. Increased solubility, preferential retention within the cytosol, and improvement of the tolerance for the linker-

attaching group are three ongoing topics of our research towards this aim within the MT space, as well as investigating the roles of linker length and geometry in indirect photoisomerisation. More broadly, we consider that single-photon antenna conjugates may prove a powerful strategy with applications to many other biological targets and photoswitches: especially for inhibitors with more steric tolerance in their binding sites, or which address targets at the organelles in which the antenna itself tends to accumulate.[46]

Conceptually however, the antenna strategy's greatest value may be in addressing the problem of background activity of photopharmaceuticals which are bioactive as their thermodynamic ground state isomer. Azobenzene photopharmaceuticals (which are the overwhelming majority of photopharmaceuticals developed to date) in particular are often *trans*-bioactive,[10,53] which is problematic for *in vivo* biological applications where it is desirable to avoid a systemic background of bioactivity. Substantial *trans* to *cis* photoisomerisation *in situ* (typically reaching only 80% completion and needing UV light) is not compatible with long-term *in vivo* applications. Thus *in vivo* applications of *trans*-active compounds may require them to be administered as the less active *cis* isomer, to be activated by *in situ cis* to *trans* photoisomerisation where activity is desired, and that it be possible to maintain or reapply this spatial patterning of bioactivity over the long term. This probably translates to requiring ≥600 nm single-photon *cis* to *trans* photoisomerisation as well as relatively slow thermal relaxation (> hours in water solution). None of the main strategies for red-shifting azobenzene absorbances beyond the UV/blue region results in bistable switches where the *cis* to *trans* isomerisation can be conducted at long wavelengths in aqueous media. The single-photon antenna strategy that we have explored can however enable highly photon-efficient and quantitative ≥600 nm *cis* to *trans* isomerisation, while necessitating only minimal redesign of the azobenzene photoswitch-pharmacophore (so that bioactivity is more likely to be retained).

In other words, such conjugates can deliver photochemically unusual solutions to the practical challenges of redshifting and completeness of isomerisation, which hinder the precise application of photopharmaceuticals *in vivo* respectively. Particularly, this offers a potential solution to the challenge of applying azobenzene-based, as well as other, photopharmaceuticals which are bioactive as their thermodynamic groundstate isomer without background activity, in a manner compatible with long-term *in vivo* applications. The key strategic advance that we demonstrate is the ability to perform highly efficient, single-photon *cis* to *trans* photoisomerisation with ≥600 nm light in aqueous solution, while necessitating only minimal redesign of the azobenzene photoswitch-pharmacophore (so that bioactivity is more likely to be retained). We believe that this method holds great promise in chemical biology, not only for the unprecedentedly precise and high-magnitude *in cellulo* spatiotemporal patterning of *cis*-active molecules such as **PST-**like photopharmaceuticals for the microtubule cytoskeleton; but more widely as a promising and minimally disruptive method to adapt the groundstate-active photoswitchable bioactives that constitute the majority of photopharmaceuticals hitherto developed for *in vivo*-compatible use.

Part A: Chemical Synthesis and Characterisation

Conventions

<u>Diazene geometry and nomenclature</u>: Azobenzenes are drawn by default in their *Z*-isomeric form to enable easier comparison with the SAR literature of their isosteric tubulin-binding stilbenes or stilbenoids such as the combretastatins. However, this should be understood to

imply either or both of the *E* & *Z* isomers constituting a given sample depending on light exposure, therefore by default they are also named without *E/Z*-designations.

Abbreviations: The following abbreviations are used: Boc – *tert*-butoxycarbonyl, brsm – based on recovered starting material, Cy – cyclohexane, DBU – 1,8-diazabicycloundec-7-ene, DCC – dicyclohexylcarbodiimide, DCM – dichloromethane, DMAP – 4-dimethylaminopyridine, DMF – dimethylformamide, EA – ethyl acetate, *i*Pen – *iso*-pentyl, *i*PenONO – isopentyl nitrite, HOBt – 1-hydroxybenzotriazole, Hx – distilled *iso*-hexanes, PBS – phosphate buffered saline, TBS – *tert*-butyldimethylsilyl, TFA – 2,2,2-trifluoroacetic acid, Ts or tosyl – *para*-toluenesulfonyl, wt% - percentage by weight.

Reagents and Conditions: Unless stated otherwise, (1) all reactions and characterisations were performed with unpurified, undried, non-degassed solvents and reagents, used as obtained, under closed air atmosphere without special precautions; (2) "hexane" used for chromatography was distilled from commercial crude *iso*-hexane fraction on rotavap; (3) "column" and "chromatography" refer to flash column chromatography, performed on Merck silica gel Si-60 (40-63 µm); (4) procedures and yields are unoptimised; (5) yields refer to isolated chromatographically and spectroscopically pure materials, corrected for residual solvent content; (6) all eluent and solvent mixtures are given as volume ratios unless otherwise specified, thus "1:1 Hx:EA" indicates a 1:1 mixture (by volume) of hexane and ethyl acetate.

Thin-layer chromatography (TLC) was run on 0.25 mm Merck silica gel plates (60, F-254). UV light (254 nm) was used as a visualising agent, and standard TLC dips based on Hanessian's cerium ammonium molybdate formulation (Han), 0.6% methanolic $FeCl_3$ ($FeCl_3$), basic $KMnO_4$ ($KMnO_4$), phosphomolybdic acid (PMA), - followed by heating where necessary - were used as developing agents. A particularly sensitive stain for azobenzenes was obtained by wafting TFA vapour over the TLC plate; the azobenzenes stain with intense orange/red/purple/blue colours according to the substitution patterns. R_f values were usually determined in hexane:ethyl acetate (Hx:EA) eluents. TLC characterisations are thus abbreviated as per (R_f = 0.19 on 6:1 Hx:EA, TFA).

NMR: Standard NMR characterisation was by ^{1}H- and ^{13}C-NMR spectra. Known compounds were checked against literature data and their spectral analysis is not detailed unless necessary. Unless otherwise stated, the spectrometer used by default was a Bruker Ascend 400 (400 MHz & 100 MHz for ^{1}H and ^{13}C respectively); the alternative spectrometer was a Bruker AVANCE 500 (500 MHz & 125 MHz for ^{1}H and ^{13}C respectively); both used at 300K. Where not indicated otherwise, the NMR solvent was $CDCl_3$. Chemical shifts (δ) are reported in ppm calibrated to residual non-perdeuterated solvent as an internal reference.[54] The following peak descriptions are used: singlet (s), doublet (d), triplet (t), quartet (q), multiplet (m), broad (br); apparent multiplicities (resolved by 2D experiments or determined by complete spectral assignment) are denoted by a tilde, eg. "doublet of doublets, appears as a triplet with apparent coupling constant *J* = 3 Hz" is denoted (~t, 3 Hz).

Mass Spectra: Unit mass measurements were performed on an AGILENT 1200 SL coupled LC-MS system with ESI mode ionisation, with binary eluent mixtures of water:acetonitrile, with the water containing formic acid. For LCMS, standard run conditions used an Eclipse Plus 3.5 µm / 4.6×100 mm C18 column, maintained at 25 °C, with a 2 mL/min flow rate, whereby the solvent front eluted at t_{ret} = 0.76 min. A linear gradient of eluent composition from 90:10→10:90 water:acetonitrile was applied over the first 4.5 min, then 10:90 maintained until all peaks of interest had been observed (typically a further 3 min). Ion peaks from (positive/negative mode) are reported as (+/-) with units Th (m/z). Thus "LCMS(+): t_{ret} = 5.60 & 5.82 min, each 419 Th = $[MH]^+$" indicates LCMS under the standard run conditions with ESI

ionisation giving two positive ion peaks eluting at 5.60 and 5.82 min retention times, each at m/z = 419 Th, attributed as the protonated molecular ion. Unless stated otherwise, all reported peaks in the positive mode were $[MH]^+$ peaks, and all observed peaks in the negative mode were $[M\text{-}H]^-$ peaks. HRMS was carried out by the Zentrale Analytik of the LMU, Munich using ESI or EI ionisation as specified.

Standard Procedures

Where Standard Procedures were used in synthesis, unless stated otherwise, the amounts of reactants/reagents employed were implicitly adjusted to maintain the same molar ratios as in the given Procedure, and no other alterations from the Standard Procedure (e.g. reaction time, extraction solvent, temperature) were made, unless stated otherwise.

Standard Procedure A: Diazonium-Phenol Coupling in MeOH

To the aniline (1 mmol) were added MeOH (5 mL) and conc. HCl (0.25 mL), and the mixture cooled in an icebath. A solution of *iso*-pentyl nitrite (1.02 mmol) in methanol (0.6 mL) was added dropwise and the reaction stirred for 30 min in the cold. A cold solution of the phenol (1.05 mmol) in methanol (2 mL) and NaOH (2.0 M, 1.8 mL) was prepared, with additional MeOH/H_2O added for solubility if needed, and to it at 0ºC was added the solution of the diazonium, dropwise over 1 minute. After typically 30 minutes stirring in the cold, the pH was adjusted to 7 with phosphate buffer, chloroform (10 mL) was added, and the aqueous phase was extracted with $CHCl_3$ (2×10 mL). The combined organic layers were washed with water (15 mL) and brine (10 mL), dried on Na_2SO_4, filtered and concentrated. Chromatography with a Hx:EA gradient was used to separate the *para*-phenolic azobenzene product which typically ran as a single isomer during chromatography.

Standard Procedure B: Phenol methylation in acetone

To the phenol (1 mmol) were added K_2CO_3 (3 mmol), technical grade acetone (10 mL), and MeI (2 mmol), and the mixture stirred at RT for 2-12 h, until TLC showed satisfactory conversion. TLC often separated the E/Z azobenzene isomers, with the major spot being the faster-running E isomer; the Z isomer typically appeared at near-identical R_f to that of the phenol starting material. The volatiles were evaporated on the rotavap, then the crude mixture was separated by chromatography with a Hx:EA gradient.

Standard Procedure C: Phenol methylation using MeI and Ag_2CO_3 in toluene

To the phenol (1 mmol) in a screw-cap pressure tube were added toluene (6 mL), Ag_2CO_3 (1 mmol, supported on Celite or not), and MeI (1.5 mmol). The tube was sealed, protected from light, and the reaction heated to 110 ºC overnight with stirring. After cooling, the crude reaction mixture was filtered, the residue washed with chloroform (2 mL), and the combined filtrates concentrated and separated on column as for Standard Procedure B.

Standard Procedure D: Diazonium-Phenol Coupling in Water

The aniline (1 mmol) was dissolved in aqueous HCl (1.2 M, 0.95 mL), diluted with water (3 mL) and cooled in an icebath while a cold aqueous solution of $NaNO_2$ (0.85 M, 1 mL, 1.2 eq) was added dropwise with stirring. Stirring in the icebath was continued for 6 min, then a cold aqueous solution of NaOH (1.0 M, 0.65 mL) was added dropwise followed by a cold solution of the phenol (1.05 mmol) in THF (4 mL). After typically four minutes stirring, the pH was adjusted to 8 with phosphate buffer, and stirring in the cold continued for 5 min before chloroform (4 mL) was added. The aqueous phase was extracted with $CHCl_3$ (2×8 mL), then the combined organic layers were washed with water (12 mL) and brine (10 mL), dried on

Na_2SO_4, filtered and concentrated. Chromatography with a Hx:EA gradient was used to separate the azobenzene product; significant quantities of byproducts were usually obtained.

Syntheses

PST-6

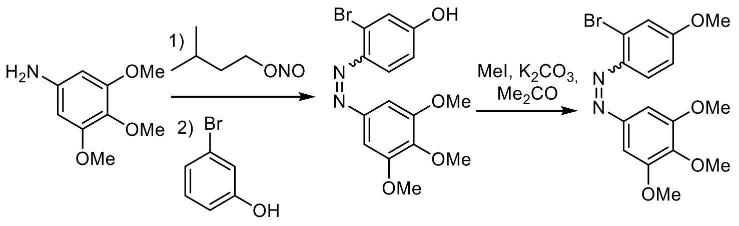

S1 7% **PST-6** 95%

3-bromo-4-((3,4,5-trimethoxyphenyl)diazenyl)phenol (**S1**)

3,4,5-Trimethoxyaniline (2.75 g, 15 mmol) was diazotized and coupled to 3-bromophenol (2.60 g, 15 mmol) following Standard Procedure A. Chromatography on 5:1→1:1 Hx:EA gradient returned **S1** (390 mg, 1.06 mmol, 7.1%; R_f = 0.56 on 1:1 Hx:EA) as an orange solid.

^{1}H NMR (400 MHz, DMSO-d_6) δ = 7.65 (d, *J* = 8.9 Hz, 1H), 7.23 (s, 1H), 7.22 (s, 2H), 6.90 (dd, *J* = 8.9 Hz, 2.5 Hz, 1H), 3.87 (s, 6H), 3.75 (s, 3H) ppm. ^{13}C NMR (100 MHz, DMSO-d_6) δ = 166.65, 158.53, 153.23, 146.61, 145.23, 132.54, 123.86, 123.83, 105.39, 65.48, 61.12 ppm. HRMS (ESI+): Calculated for $[C_{15}H_{16}BrN_2O_4]^+$ = $[MH]^+$: m/z 367.02880, found 367.02882.

1-(2-bromo-4-methoxyphenyl)-2-(3,4,5-trimethoxyphenyl)diazene (**PST-6**)

S1 (375 mg, 1 mmol) was methylated by Standard Procedure B. Chromatography on 5:1 Hx:EA returned **PST-6** (364 mg, 0.95 mmol, 95%; R_f = 0.83 on 1:1 Hx:EA) as an orange solid.

^{1}H NMR (400 MHz, $CDCl_3$) δ = 7.72 (d, *J* = 9.0 Hz, 1H), 7.28 – 7.26 (m, 3H), 7.25 (s, 2H), 6.92 (dd, *J* = 9.0 Hz, 2.7 Hz, 1H), 3.96 (s, 6H), 3.93 (s, 3H), 3.88 (s, 3H) ppm. ^{13}C NMR (100 MHz, $CDCl_3$) δ = 162.07, 153.51, 148.70, 143.47, 140.60, 127.77, 118.48, 117.87, 114.58, 100.59, 61.07, 56.16, 55.89 ppm. LCMS(+): t_{ret}=4.32 (Z) + 5.30 (E) min, 381 Th = $[MH]^+$. HRMS (ESI+): Calculated for $[C_{16}H_{18}BrN_2O_4]^+$ = $[MH]^+$: m/z 381.04445, found 381.04452.

PST-7

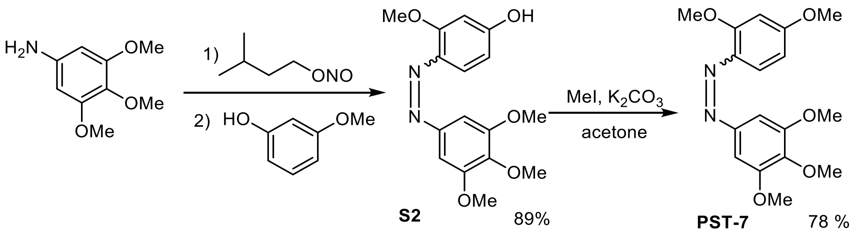

3-methoxy-4-((3,4,5-trimethoxyphenyl)diazenyl)phenol (**S2**):

By Standard Procedure A, 3,4,5-trimethoxyaniline (366 mg, 2.00 mmol) was reacted with commercial 3-methoxyphenol (240 mg, 2.05 mmol). Chromatography of the red crude oil on 5:1→1:1 Hx:EA gradient returned **S2** (564 mg, 1.77 mmol, 89%; R_f = 0.11 on 2.4:1 Hx:EA, $FeCl_3$) as a deep red powder.

^{1}H NMR (400 MHz, DMSO-d$_6$): δ = 10.32 (s br, 1H), 7.55 (d, *J* = 8.8 Hz, 1H), 7.12 (s, 2H), 6.60 (d, *J* = 2.4 Hz, 1H), 6.45 (dd, *J* = 8.8 Hz, 2.4 Hz, 1H), 3.91 (s, 3H), 3.87 (s, 6H), 3.74 (s, 3H) ppm. ^{13}C NMR (100 MHz, DMSO-d$_6$): δ = 163.0, 159.3, 153.7 (×2), 149.0, 139.6, 135.2, 118.0, 108.4, 100.4, 100.1 (×2), 60.7, 56.4 (×2), 56.3 ppm. LCMS(+): t_{ret} = 3.57 min, 319 Th = [MH]$^+$. HRMS (ESI+) calcd for $[C_{16}H_{19}N_2O_5]^+$ = [MH]$^+$: m/z 319.12885, found 319.12856.

1-(2,4-dimethoxyphenyl)–2-(3,4,5-trimethoxyphenyl)diazene (**PST-7**)

By Standard Procedure B, **S2** (550 mg, 1.73 mmol) was methylated for 6 hours. Chromatography on 5:1→1:1 Hx:EA gradient returned **PST-7** (460 mg, 1.38 mmol, 78%; R_f = 0.19 on 2.4:1 Hx:EA, $FeCl_3$) as an orange powder.

^{1}H NMR (400 MHz, DMSO-*d*$_6$) δ = 7.60 (d, *J* = 8.9 Hz, 1H), 7.14 (s, 2H), 6.77 (d, *J* = 2.5 Hz, 1H), 6.62 (dd, *J* = 9.0, 2.5 Hz, 1H), 3.96 (s, 3H), 3.87 (m, 9H), 3.74 (s, 3H) ppm. ^{13}C NMR (100 MHz, DMSO) δ = 163.52, 158.48, 153.27, 148.44, 139.45, 135.73, 117.44, 106.19, 99.87, 99.20, 60.22, 56.21, 55.95, 55.71 ppm. LCMS(+): t_{ret} = 3.72 and 4.42 min, 333 Th = [MH]$^+$: these peaks were assigned to the *cis* & *trans* isomers respectively since the UV absorption profile of the first peak (*cis*) featured a secondary band at around 440 nm which was absent in the second peak. HRMS (ESI+) calcd for $[C_{17}H_{21}N_2O_5]^+$ = [MH]$^+$: m/z 333.14450, found 333.14430.

PST-8

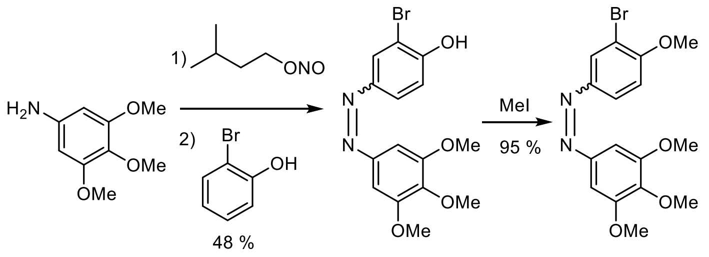

S3 **PST-8**

2-bromo-4-((3,4,5-trimethoxyphenyl)diazenyl)phenol (**S3**)

By Standard Procedure A, 3,4,5-trimethoxyaniline (3.67 g, 20.0 mmol) was reacted with commercial 2-bromophenol (3.46 g, 20.0 mmol). Chromatography of the orange crude oil on 5:1→1:1 Hx:EA gradient returned **S3** (3.52 g, 9.62 mmol, 48%; R_f = 0.66 on 1:1 Hx:EA, Han) as a yellow solid.

^{1}H NMR (400 MHz, $CDCl_3$): δ = 8.03 (d, *J* = 2.3 Hz, 1H), 7.80 (dd, *J* = 8.7 Hz 2.3 Hz, 1H), 7.15 (s, 2H), 7.09 (d, *J* = 8.7 Hz, 1H), 3.89 (s, 6H), 3.87 (s, 3H) ppm. ^{13}C NMR (100 MHz, $CDCl_3$): δ = 154.4, 153.5 (×2), 148.2, 147.1, 140.6, 125.5, 125.5, 116.1, 111.1, 100.3 (×2), 61.1, 56.2 (×2) ppm. HRMS (ESI+) calcd for $[C_{15}H_{16}N_2O_4Br]^+$ = $[MH]^+$: m/z 367.02880, found 367.02857. LCMS(+): t_{ret} = 4.38 min, 367 and 369 Th = $[MH]^+$.

1-(3-bromo-4-methoxyphenyl)-2-(3,4,5-trimethoxyphenyl)diazene (**PST-8**)

By Standard Procedure B, **S3** (2.40 g, 6.54 mmol) was methylated overnight. Chromatography on 5:1→1:1 Hx:EA gradient returned **PST-8** (2.37 g, 6.21 mmol, 95%; R_f = 0.69 and 0.51 on 1:1 Hx:EA, *trans* and *cis* isomers, $FeCl_3$) as orange crystals.

^{1}H NMR (400 MHz, $CDCl_3$): δ = 8.19 (d, *J* = 2.3 Hz, 1H), 7.94 (dd, *J* = 8.7 Hz, 2.4 Hz, 1H), 7.24 (s, 2H), 7.05 (d, *J* = 8.8 Hz, 1H), 4.01 (s, 3H), 3.99 (s, 6H), 3.96 (s, 3H ppm. ^{13}C NMR (100 MHz, $CDCl_3$): δ = 157.94, 153.63, 148.39, 147.03, 140.61, 126.11, 125.74, 112.76, 111.47, 100.36, 61.18, 56.69, 56.31 ppm. LCMS(+): t_{ret} = 4.20 & 5.28 min, each 381 and 383 Th = $[MH]^+$: these peaks were assigned to the *cis* & *trans* isomers respectively since the UV absorption profile of the first peak (*cis*) featured a secondary band centred around 445 nm which was absent in the second peak. HRMS (ESI+) calcd for $[C_{16}H_{18}N_2O_4Br]^+$ = $[MH]^+$: m/z 381.04445, found 381.04419.

PST-9

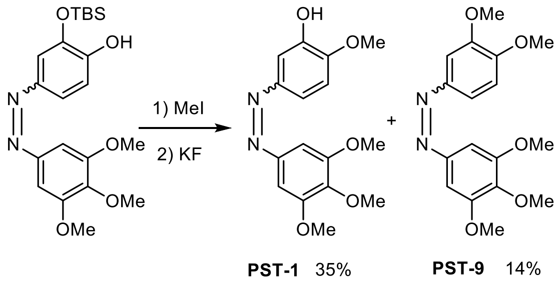

1-(3,4-dimethoxyphenyl)–2-(3,4,5-trimethoxyphenyl)diazene (**PST-9**):

Sufficient **PST-9** for biological testing could be recovered as a byproduct during synthesis of **PST-1** according to the published procedure[26]. In brief, to 2-((*tert*-butyldimethylsilyl)oxy)–4-((3,4,5-trimethoxyphenyl)diazenyl)phenol[26] (73 mg, 0.175 mmol) were added K_2CO_3 (44 mg, 0.32 mmol), dry DMF (2 mL), and MeI solution (1.37 g of a 4.3 wt% solution in DMF, 0.43 mmol), and the mixture stirred at RT for 2 h until TLC (5:1 Hx:EA) showed complete conversion of the starting material to a faster-running product. The volatiles were evaporated at 60 °C and 5 mbar, then THF (8 mL) and an aqueous solution of KF (1 M, 5 mL) were added to the residue and the mixture stirred at RT for 3.5 h. The bulk of the THF was removed on the rotavap, then water (15 mL), brine (2 mL), and KH_2PO_4/K_2HPO_4 buffer (2 M, pH = 6.8, 4 mL) were added and the aqueous phase extracted with dichloromethane (3×15 mL). The combined organic layers were washed with water (15 mL) and brine (10 mL) and dried on Na_2SO_4, filtered and concentrated to a crude oil. Flash chromatography on 5:1→2.4:1 Hx:EA gradient returned **PST-9** (8.0 mg, 0.024 mmol, 14% over 2 steps) before **PST-1**[26]. **PST-9** displayed R_f (*trans*/*cis*) = 0.34 and 0.14 on 2.4:1 Hx:EA; R_f (*trans*/*cis*) = 0.53 and 0.28 on 1.7:1 Hx:EA (Anis); yellow solid.

^{1}H NMR (400 MHz, CD_3CN): δ = 7.60 (dd, *J* = 8.5 Hz, 2.2 Hz, 1H), 7.52 (d, *J* = 2.2 Hz, 1H), 7.26 (s, 2H), 7.12 (d, *J* = 8.5 Hz, 1H), 3.93 (s, 6H), 3.93 (s, 3H), 3.91 (s, 3H), 3.83 (s, 3H) ppm. ^{13}C NMR (100 MHz, CD_3CN): δ = 154.72, 153.17, 150.82, 149.44, 147.51, 141.15, 121.26, 111.97, 102.82, 100.92, 60.98, 56.71, 56.58, 56.27 ppm. LCMS(+): t_{ret} = 3.45 & 4.35 min, each 333.1 Th = $[MH]^+$: these peaks were assigned to the *cis* & *trans* isomers respectively since the first peak had a secondary absorption band at 440 nm. HRMS (ESI+) calcd for $[C_{17}H_{21}N_2O_5]^+$ = $[MH^+]$: m/z 333.1445, found 333.1443.

PST-10

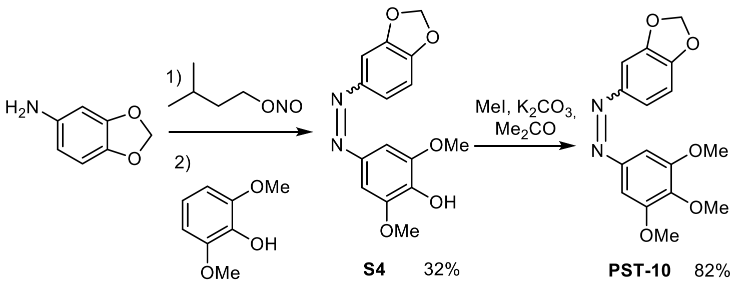

4-(benzo[d][1,3]dioxol-5-yldiazenyl)-2,6-dimethoxyphenol (**S4**)

5-Aminobenzo[1,3]dioxole (411 mg, 3 mmol) was diazotized and coupled to 2,6-dimethoxyphenol (426 mg, 3 mmol) by Standard Procedure A. Chromatography on 5:1→1:1 Hx:EA gradient returned **S4** (290 mg, 0.96 mmol, 32%; R_f = 0.54 on 1:1 Hx:EA) as a red solid.

^{1}H NMR (400 MHz, $CDCl_3$) δ = 7.54 (dd, *J* = 8.2 Hz, 1.9 Hz, 1H), 7.41 (d, *J* = 1.9 Hz, 1H), 7.25 (s, 2H), 6.94 (d, *J* = 8.2 Hz, 1H), 6.06 (s, 2H), 5.81 (s, 1H), 4.00 (s, 6H) ppm. ^{13}C NMR (100 MHz, $CDCl_3$) δ = 149.86, 148.77, 148.46, 147.20, 145.43, 137.37, 122.92, 107.99, 101.85, 100.18, 98.97, 56.40 ppm. LCMS(+): t_{ret}= 4.00 (E) min, 303 Th = $[MH]^+$. HRMS (ESI+): Calculated for $[C_{15}H_{15}N_2O_5]^+$ = $[MH]^+$: m/z 303.09755 found 303.09722.

1-(benzo[d][1,3]dioxol-5-yl)-2-(3,4,5-trimethoxyphenyl)diazene (**PST-10**)

S4 (270 mg, 0.89 mmol) was methylated following Standard Procedure B. Chromatography on 5:1 Hx:EA returned **PST-10** (231 mg, 0.73 mmol, 82%; R_f = 0.80 on 1:1 Hx:EA) as an orange solid.

^{1}H NMR (400 MHz, $CDCl_3$) δ = 7.50 (dd, *J* = 8.2 Hz, 1.9 Hz, 1H), 7.35 (d, *J* = 1.9 Hz, 1H), 7.14 (s, 2H), 6.88 (d, *J* = 8.2 Hz, 1H), 6.00 (s, 2H), 3.89 (s, 6H), 3.86 (s, 3H) ppm. ^{13}C NMR (100 MHz, $CDCl_3$) δ = 153.64, 150.32, 148.94, 148.54, 148.52, 140.33, 123.60, 108.13, 102.05, 100.24, 99.08, 61.19, 56.33 ppm. LCMS(+): t_{ret}=3.10 (Z) + 4.67 (E) min, 317 Th = $[MH]^+$. HRMS (ESI+): Calculated for $[C_{16}H_{17}N_2O_5]^+$ = $[MH]^+$: m/z 317.11320, found 317.11286.

PST-11 & PST-12

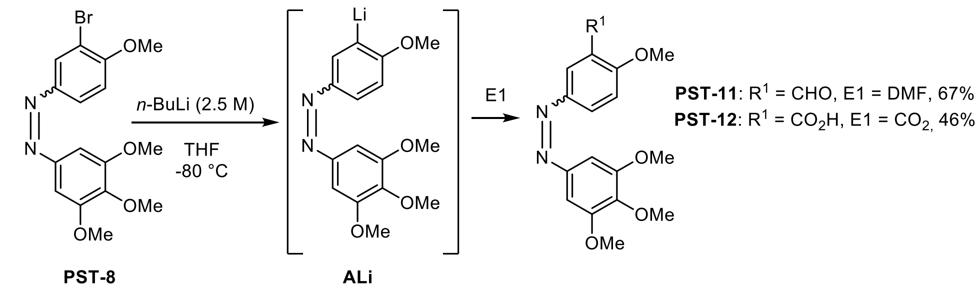

(2-methoxy-5-((3,4,5-trimethoxyphenyl)diazenyl)phenyl)lithium stock solution "**ALi**"

To **PST-8** (1.20 g, 3.15 mmol) under nitrogen at –80 °C were added dry THF (9 mL) and, dropwise, *n*-butyllithium (2.5 M in hexanes, 1.32 mL, 3.30 mmol). The solution darkened significantly over the course of the addition. This stock solution "**ALi**" of the azoaryllithium intermediate (approximately 0.30 M) was aged at –80 °C for 1 hour, then used without delay.

2-methoxy-5-((3,4,5-trimethoxyphenyl)diazenyl)benzaldehyde (**PST-11**)

To a solution of DMF (0.2 mL) in dry THF (3 mL) at –80 °C under nitrogen was added dropwise the stock solution "**ALi**" (1.0 mL, approx. 0.30 mmol). The solution was warmed to room temperature and stirred for 30 min, then quenched by its dropwise addition into a rapidly-stirred mixture of aqueous KH_2PO_4 solution (10%, 15 mL) and Et_2O (10 mL). The aqueous layer was extracted with Et_2O (2×10 mL), then the combined organic layers were washed with water (10 mL), brine (10 mL), dried on Na_2SO_4, filtered and concentrated. The brown crude oil was separated by chromatography on 5:1→1:1 Hx:EA gradient yielding **PST-11** (67 mg, 0.21 mmol, 67%; R_f = 0.50 and 0.29 on 1:1 Hx:EA, *trans* and *cis* isomers, Han) as a yellow oil.

^{1}H NMR (400 MHz, $CDCl_3$): δ = 10.45 (s, 1H), 8.34 (d, *J* = 2.6 Hz, 1H), 8.08 (dd, *J* = 8.9 Hz, 2.6 Hz, 1H), 7.17 (s, 2H), 7.06 (d, *J* = 8.9 Hz, 1H), 3.96 (s, 3H), 3.90 (s, 6H), 3.86 (s, 3H) ppm. ^{13}C NMR (100 MHz, $CDCl_3$): δ = 189.49, 163.44, 153.63, 148.40, 146.41, 140.68, 130.09, 125.15, 123.51, 112.23, 100.42, 61.17, 56.31 ppm. HRMS (ESI+) calcd for $[C_{17}H_{19}N_2O_5]^+$ = $[MH]^+$: m/z 331.12885, found 331.12855. LCMS(+): t_{ret} = 3.61 & 4.59 min, each 331 Th = $[MH]^+$: these peaks were assigned to the *cis* & *trans* isomers respectively since the UV absorption profile of the first peak (*cis*) featured a secondary band centred around 440 nm which was absent in the second peak.

2-methoxy-5-((3,4,5-trimethoxyphenyl)diazenyl)benzoic acid (**PST-12**)

To a mixture of solid CO_2 (1 g) in dry THF (5 mL) at –80 °C under nitrogen was added dropwise the stock solution "**ALi**" (1.0 mL, approx. 0.30 mmol). The drops of "**ALi**" lightened in colour immediately upon leaving the needle. The resultant yellow solution was warmed to room temperature and stirred for 5 min, then poured into a rapidly-stirred mixture of aqueous KH_2PO_4 solution (10%, 15 mL) and Et_2O (10 mL), rinsing the flask once with Et_2O (5 mL). The aqueous layer was extracted with Et_2O (2×10 mL), then the combined organic layers were washed with water (10 mL), brine (10 mL), dried on Na_2SO_4, filtered and concentrated. The brown crude oil was separated by chromatography on 5:1:0→1:1:0→1:1:0.5 Hx:EA:MeOH gradient yielding **PST-12** (48 mg, 0.14 mmol, 46%; R_f = 0.13 on 1:1:0.5 Hx:EA:MeOH, *trans* and *cis* isomers overlap, Han) as a brown solid.

^{1}H NMR (400 MHz, CD_3CN): δ = 8.46 (d, *J* = 2.6 Hz, 1H), 8.13 (dd, *J* = 8.9 Hz, 2.6 Hz, 1H), 7.35 (d, *J* = 8.9 Hz, 1H), 7.30 (s, 2H), 4.09 (s, 3H), 3.93 (s, 6H), 3.83 (s, 3H) ppm. ^{13}C NMR (100 MHz, CD_3CN): δ = 165.39, 160.71, 154.10, 148.63, 146.49, 141.02, 128.98, 126.75, 119.53, 113.52, 100.65, 60.40, 57.20, 56.15 ppm. HRMS (ESI+) calcd for $[C_{17}H_{19}N_2O_6]^+$ = $[MH]^+$: m/z 347.12376, found 347.12343. LCMS(+): t_{ret} = 2.95 & 3.69 min, each 347 Th = $[MH]^+$: these peaks were assigned to the *cis* & *trans* isomers respectively since the UV absorption profile of the first peak (*cis*) featured a secondary band centred around 440 nm which was absent in the second peak.

PST-13, PST-14 & PST-15

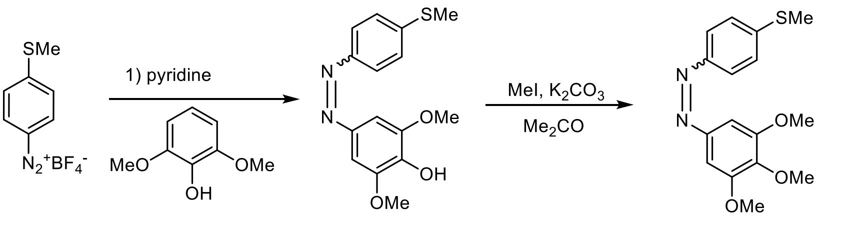

S5 58% **PST-13** 34%

2,6-dimethoxy-4-((4-(methylthio)phenyl)diazenyl)phenol (**S5**)

2,6-dimethoxyphenol (0.129 g, 0.84 mmol) was dissolved in 34 mL of acetonitrile and cooled to 0 °C by means of an ice bath. Pyridine (0.135 mL, 0.6 mmol) was added, followed by portion-wise addition of methylthiophenyl diazonium tetrafluoroborate[55] (0.2 g, 0.84 mmol). After the addition was completed, the reaction was allowed to stir for 2 h at 0 °C before it was quenched with water, extracted with CH_2Cl_2 and dried over $MgSO_4$. The column purification was performed using 7:3 Hx:EA to yield **S5** (150 mg, 0.493 mmol, 58%).

^{1}H NMR (400 MHz, $CDCl_3$) δ = 7.76 (d, *J* = 8Hz, 2H), 7.27 (d, *J* = 8 Hz, 2H),7.21 (s, 2H), 5.77 (s, 1H), 3.93 (s, 6H), 2.48 (s, 3H) ppm. ^{13}C NMR (100 MHz, $CDCl_3$) δ = 149.89, 147.22, 145.63, 142.15, 137.63, 126.03, 123.08, 100.32, 56.41, 15.36 ppm. HRMS (ESI+): Calculated for $[C_{15}H_{17}N_2O_3S]^+$ = $[MH]^+$: m/z 305.09599, found 305.09555.

1-(4-(methylthio)phenyl)-2-(3,4,5-trimethoxyphenyl)diazene (**PST-13**)

To **S5** (0.130 g, 0.427 mmol) were added K_2CO_3 (0.177 g, 1.281 mmol), technical grade acetone (5 mL), and MeI (53 µL, 0.854 mmol). The reaction was allowed to stir at rt for 16 hr. After evaporating solvent under vacuum, the residue was dissolved in CH_2Cl_2, washed with brine, water, and dried over anhydrous $MgSO_4$. Purification using 8:2 Hx:EA yielded the methylated azobenzene **PST-13** (46 mg, 0.144 mmol, 34%).

^{1}H NMR (400 MHz, $CDCl_3$) δ = 7.87 (d, *J* = 8 Hz, 2H), 7.36 (d, *J* = 8 Hz, 2H), 7.26 (s, 2H), 3.99 (s, 6H), 3.95 (s, 3H), 2.58 (s, 3H) ppm. ^{13}C NMR (100 MHz, $CDCl_3$) δ = 153.52, 149.80, 148.55, 142.75, 140.43, 125.95, 123.25, 100.24, 61.07, 56.21, 15.30 ppm. HRMS (ESI+): Calculated for $[C_{16}H_{19}N_2O_3S]^+$ = $[MH]^+$: m/z 319.11109, found 319.11107.

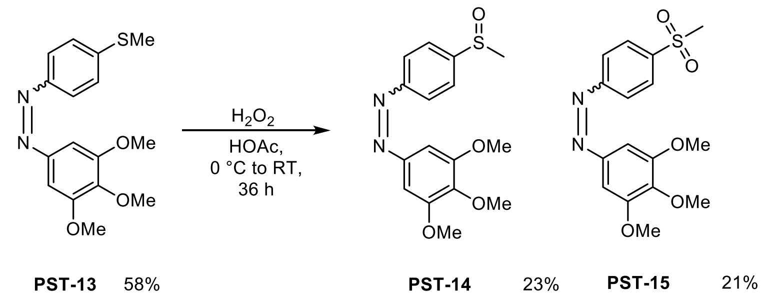

1-(4-(methylsulfinyl)phenyl)-2-(3,4,5-trimethoxyphenyl)diazene (**PST-14**) &

1-(4-(methylsulfonyl)phenyl)-2-(3,4,5-trimethoxyphenyl)diazene (**PST-15**)

A solution of **PST-13** (25 mg, 0.078 mmol) in 1 mL of HOAc was cooled to 0 °C by using an ice bath and then was added dropwise another 1 mL of HOAc containing 10 μL of 30% H_2O_2 (3 mg, 0.088 mmol). The reaction mixture was allowed to stir for 36 h. The solvent was removed under vacuum, reconstituted with CH_2Cl_2, washed with H_2O, dried over $MgSO_4$. Purification using 7:3 Hx:EA yielded sulfone derivative **PST-14** (6.3 mg, 0.018 mmol, 23%) and sulfoxide derivative **PST-15** (5.4 mg, 0.016 mmol, 21%).

PST-14: ^{1}H NMR (400 MHz, $CDCl_3$) δ = 8.04 (d, *J* = 8 Hz, 2H), 7.80 (d, *J* = 8 Hz, 2H), 7.26 (s, 2H), 3.98 (s, 6H), 3.95 (s, 3H), 2.79 (s, 3H) ppm. ^{13}C NMR (100 MHz, $CDCl_3$) δ = 152.16, 151.63, 146.33, 145.79, 139.37, 122.50, 121.60, 98.82, 59.17, 54.32, 42.07 ppm. HRMS (ESI+): Calculated for $[C_{16}H_{19}N_2O_4S]^+$ = $[MH]^+$: m/z 335.10600, found 335.10588.

PST-15: ^{1}H NMR (400 MHz, $CDCl_3$) δ = 8.08 (d, *J* = 8 Hz, 2H), 8.02 (d, *J* = 8 Hz, 2H), 7.24 (s, 2H), 3.96 (s, 6H), 3.94 (s, 3H), 3.10 (s, 3H) ppm. ^{13}C NMR (101 MHz, $CDCl_3$) δ = 155.38, 153.58, 148.19, 141.78, 141.48, 128.59, 123.38, 101.02, 61.14, 56.28, 44.58 ppm. HRMS (ESI+): Calculated for $[C_{16}H_{19}N_2O_5S]^+$ = $[MH]^+$: m/z 351.10092, found 351.10089.

PST-16

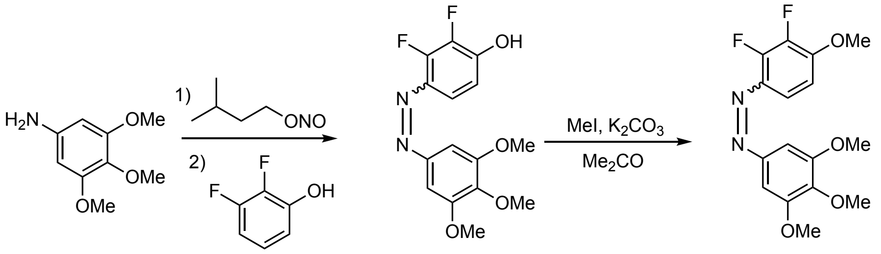

S6 28% **PST-16** 83%

2,3-difluoro-4-((3,4,5-trimethoxyphenyl)diazenyl)phenol (**S6**):

By Standard Procedure A, 3,4,5-trimethoxyaniline (186 mg, 1.02 mmol) was reacted with 2,3-difluorophenol (140 mg, 1.08 mmol). Chromatography on 5:1→2.5:1 Hx:EA returned **S6** (91 mg, 0.28 mmol, 28%; R_f = 0.26 on 1.7:1 Hx:EA, Van) as a yellow oil.

^{1}H NMR (400 MHz, $CDCl_3$): δ = 7.46 (ddd, *J* = 9.2 Hz, 7.5 Hz, 2.3 Hz, 1H), 7.17 (s, 2H), 6.78 (ddd, *J* = 9.3 Hz, 8.0 Hz, 2.1 Hz, 1H), 5.79 (s br, 1H), 3.89 (s, 6H), 3.87 (s, 3H) ppm. ^{13}C NMR (100 MHz, $CDCl_3$): δ = 153.5 (×2), 149.1 (dd, *J* = 260.6 Hz, 11.3 Hz), 148.6, 147.3 (d, *J* = 11.4 Hz), 140.4 (dd, *J* = 240.2 Hz, 13.4 Hz), 140.9, 135.4 (d, *J* = 4.7 Hz), 112.7 (d, *J* = 3.7 Hz), 111.9 (d, *J* = 3.6 Hz), 100.6 (×2), 61.1, 56.24 (×2) ppm. HRMS (ESI-) calcd for $[C_{15}H_{13}N_2O_4F_2]^-$ = $[M-H]^-$: m/z 323.08489, found 323.08495.

1-(2,3-difluoro-4-methoxyphenyl)-2-(3,4,5-trimethoxyphenyl)diazene (**PST-16**):

By Standard Procedure B, **S6** (89 mg, 0.27 mmol) was methylated overnight. Chromatography on 10:1→2:1 Hx:EA returned **PST-16** (79 mg, 0.23 mmol, 83%; R_f = 0.34 and 0.17 on 2.4:1 Hx:EA, $FeCl_3$: E and Z isomers) as fine orange crystals.

^{1}H NMR (400 MHz, DMSO-d_6): δ = 7.59 (ddd, *J* = 9.3 Hz, 8.0 Hz, 2.3 Hz, 1H), 7.23 (s, 2H), 7.19 (ddd, *J* = 9.7 Hz, 8.0 Hz, 1.9 Hz, 1H), 3.98 (s, 3H), 3.89 (s, 6H), 3.78 (s, 3H) ppm. ^{13}C NMR (100 MHz, DMSO-d_6): δ = 153.34, 150.90 (dd, *J* = 7.8, 3.0 Hz), 148.22 (dd, *J* = 256.2, 10.9 Hz), 147.88, 140.62, 140.47 (dd, *J* = 245.7, 12.8 Hz), 134.44 (d, *J* = 4.8 Hz), 112.33 (d, *J* = 3.7 Hz), 108.74 (d, *J* = 2.5 Hz), 100.34, 60.28, 56.94, 56.01 ppm. ^{19}F NMR (282 MHz, DMSO-d_6): δ = -148.54 (ddd, *J* = 20.0 Hz, 8.1 Hz, 2.1 Hz), -159.65 (ddd, *J* = 20.1 Hz, 8.1 Hz, 2.5 Hz) ppm. LCMS(+): t_{ret} = 4.09 & 4.84 min, each 338 Th = $[MH]^+$: these peaks were assigned to the Z & E isomers respectively since the UV absorption profile of the first peak (Z) had a secondary band centred at 440 nm which was absent in the first peak. HRMS (EI+) calcd for $[C_{16}H_{16}N_2O_4F_2]^+$ = $[M]^+$: m/z 338.1078, found 338.1074.

PST-17

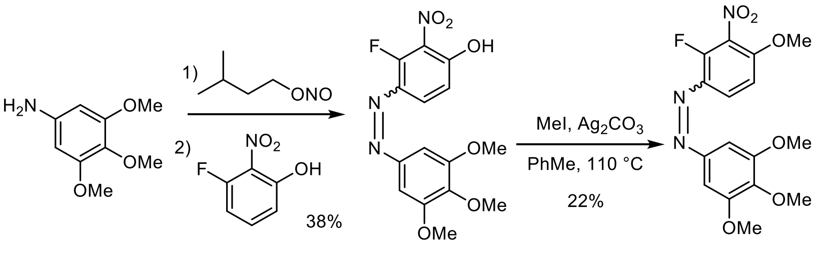

3-fluoro-2-nitro-4-((3,4,5-trimethoxyphenyl)diazenyl)phenol (**S7**):

By Standard Procedure A, 3,4,5-trimethoxyaniline (185 mg, 1.01 mmol) was reacted with 3-fluoro-2-nitrophenol (162 mg, 1.03 mmol), where the reaction between phenolate and diazonium was stirred for 5 h in the dark in the cold to allow for better conversion of the slow-reacting materials. Chromatography on 1:1:0→5:1:0→5:1:0.5 Hx:EA:MeOH returned **S7** (134 mg, 0.38 mmol, 38%; R_f = 0.16 on 1:5 Hx:EA, Han) as a red solid.

^{1}H NMR (400 MHz, $CDCl_3$): δ = 10.68 (s, 1H), 8.02 (dd, *J* = 9.4 Hz, 7.6 Hz, 1H), 7.26 (s, 2H), 6.98 (dd, *J* = 9.4 Hz, 1.9 Hz, 1H), 3.90 (s, 6H), 3.88 (s, 3H) ppm. ^{13}C NMR (100 MHz, $CDCl_3$): δ = 157.4 (~s), 155.8 (d, *J* = 279 Hz), 153.6 (×2), 148.3, 141.5, 134.4 (d, *J* = 6.3 Hz), 125.8 (d, *J* = 7.4 Hz), 125.0 (d, *J* = 3.3 Hz), 114.7 (d, *J* = 4.3 Hz), 100.9 (×2), 61.10, 56.3 (×2) ppm. LCMS(+): t_{ret} = 4.25 min, 352 Th = $[MH]^+$. HRMS (ESI-) calcd for $[C_{15}H_{13}N_3O_6F]^-$ = $[M-H]^-$: m/z 350.0794, found 350.0796.

1-(2-fluoro-4-methoxy-3-nitrophenyl)-2-(3,4,5-trimethoxyphenyl)diazene (**PST-17**):

Standard Procedure B gave poor yields of the desired product. Therefore, by Standard Procedure C, **S7** (130 mg, 0.37 mmol) was methylated using MeI (69 mg, 0.48 mmol) and Ag_2CO_3 (50% on Celite, 210 mg, 0.38 mmol) in toluene (6 mL). The crude was separated with 3:1→2:1 Hx:EA to afford **PST-17** (30 mg, 0.08 mmol, 22%; R_f = 0.19 on 2.4:1 Hx:EA, $FeCl_3$) as an orange solid.

^{1}H NMR (400 MHz, $CDCl_3$): δ = 7.91 (dd, *J* = 9.3, 8.1 Hz, 1H), 7.24 (s, 2H), 6.90 (dd, *J* = 9.4, 1.7 Hz, 1H), 4.00 (s, 3H), 3.95 (s, 6H), 3.94 (s, 3H) ppm. ^{13}C NMR (100 MHz, $CDCl_3$): δ = 153.90 (d, *J* = 2.5 Hz), 153.68, 152.35 (d, *J* = 267.3 Hz), 148.50, 141.56, 134.65 (d, *J* = 6.1 Hz), 131.52 (d, *J* = 14.6 Hz), 120.16 (d, *J* = 2.0 Hz), 107.80 (d, *J* = 3.6 Hz), 100.94, 61.21, 57.29, 56.37 ppm. ^{19}F-NMR (282 MHz): δ = -132.69 (dd, *J* = 8.1, 1.9 Hz) ppm. LCMS(+): t_{ret} = 4.18 & 4.83 min, each 366 Th = $[MH]^+$: these peaks were assigned to the Z & E isomers respectively since the first peak showed a secondary band at 445 nm whereas the second peak had a single-band structure. HRMS (ESI+) calcd for $[C_{16}H_{17}N_3O_6F]^+$ = $[MH^+]$: m/z 366.10959, found 366.10944.

PST-18

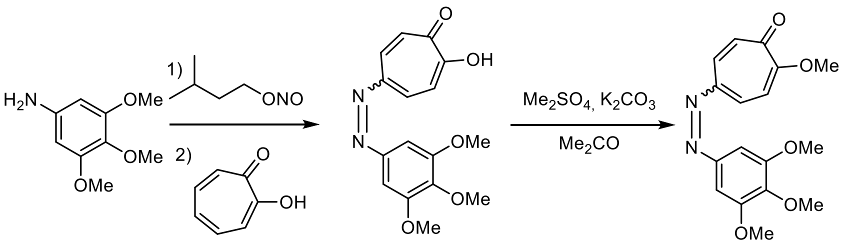

S8 52% **PST-18** 49%

2-hydroxy-5-((3,4,5-trimethoxyphenyl)diazenyl)cyclohepta-2,4,6-trien-1-one (**S8**)

3,4,5-Trimethoxyaniline (366 mg, 2 mmol) was diazotized and coupled to tropolone (244 mg, 2 mmol) following Standard Procedure A. Chromatography on 2.4:1:0→1:1:0.25 Hx:EA:MeOH returned **S8** (330 mg, 1.04 mmol, 52%; R_f = 0.51 on 1:1:0.5 Hx:EA:MeOH) as a red solid.

^{1}H NMR (400 MHz, DMSO-d_6) δ = 8.10 – 7.91 (m, 2H), 7.15 (s, 2H), 6.93 – 6.76 (m, 2H), 3.87 (s, 6H), 3.73 (s, 3H). ^{13}C NMR (100 MHz, DMSO-d_6) δ = 183.6, 153.8, 152.7, 148.6, 139.1, 132.4, 120.9, 99.8, 60.7, 56.3 ppm. LCMS(+): t_{ret} = 4.07 min, 317 Th = [MH]$^+$. HRMS (ESI+): Calculated for $[C_{16}H_{17}N_2O_5]^+$ = [MH]$^+$: m/z 317.11320, found 317.11288.

2-methoxy-5-((3,4,5-trimethoxyphenyl)diazenyl)cyclohepta-2,4,6-trien-1-one (**PST-18**)

S8 (340 mg, 1.08 mmol) was methylated using dimethyl sulfate (417 mg, 3.3 mmol) and potassium carbonate (152 mg, 1.1 mmol) in 25 mL dry acetone for 2 h. After removing the volatiles in vacuo the residue was purified by chromatography on 5:1→1:5 Hx:EA. **PST-18** was obtained as orange solid (174 mg, 0.53 mmol, 49%; R_f = 0.24 on 1:5 Hx:EA).

^{1}H NMR (400 MHz, $CDCl_3$) δ = 8.12 (dd, *J* = 12.9, 2.2 Hz, 1H), 7.87 (dd, *J* = 10.6, 2.2 Hz, 1H), 7.33 (d, *J* = 12.9 Hz, 1H), 7.22 (s, 2H), 6.94 (d, *J* = 10.7 Hz, 1H), 4.05 (s, 3H), 3.96 (s, 6H), 3.94 (s, 3H) ppm. ^{13}C NMR (100 MHz, $CDCl_3$) δ = 180.36, 164.69, 153.71, 150.89, 148.07, 141.27, 136.46, 134.83, 127.95, 111.10, 100.73, 61.24, 56.88, 56.36 ppm. LCMS(+): t_{ret}=4.88 (Z) + 5.33 (E) min, 381 Th = [MH]$^+$. HRMS (ESI+): Calculated for $[C_{17}H_{19}N_2O_5]^+$ = [MH]$^+$: m/z 331.12885, found 331.12849.

PST-19

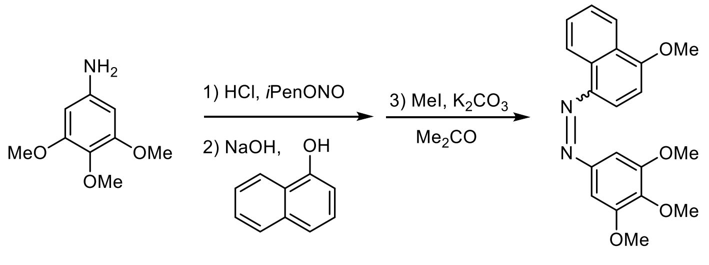

PST-19 9%

1-(4-methoxynaphthalen-1-yl)-2-(3,4,5-trimethoxyphenyl)diazene (**PST-19**)[22]

By Standard Procedure A, 3,4,5-trimethoxyaniline (384 mg, 2.1 mmol) was reacted with 1-naphthol (360 mg, 2.5 mmol) following standard procedure A. The crude compound, before column purification, was methylated directly using MeI (600 mg, 4.2 mmol) as in Standard Procedure B. Very slow gradient flash chromatography on 10:1 → 5:1 Hx:EA returned known **PST-19**[22] (68 mg, 0.19 mmol, 9%; R_f = 0.81 on 1:1 Hx:EA; LCMS(+): t_{ret}=4.57 & 5.54 min, each 353 Th = $[MH]^+$) as a yellow solid that was separated from the presumed non-methylated *ortho*-coupling product (10 mg, 0.03 mmol, 1%; R_f = 0.86 on 1:1 Hx:EA; LCMS(+): t_{ret}=5.45 min, 339 Th = $[MH]^+$) and from non-methylated *para*-coupling product (57 mg, 0.17 mmol, 8%; R_f = 0.56 on 1:1 Hx:EA; LCMS(+): t_{ret}=4.46 min, 339 Th = $[MH]^+$).

NMR of this poorly water-soluble compound matched literature data from Brittain[23].

Note: Brittain has also revived a discussion of the differential solubility[56] and LogP of azobenzene *trans* and *cis* isomers, which may generally be important in the roughly hundred-fold cellular bioconcentration and subsequent cellular biolocalisation patterning which can be expected to affect these compounds[57]. Such factors may be key to explaining the still unpredictable relationships between the apparent potencies of combretastatin analogues measured *in cellulo*, compared to their binding potencies on purified tubulin *in vitro*, or their observed potencies *in vivo*; and we recommend the paper[56] to the interested reader.

PST-20

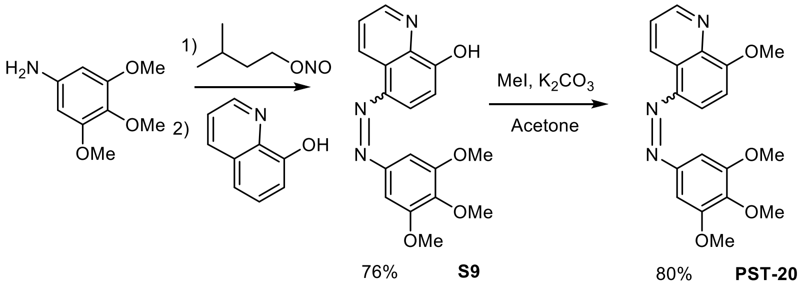

5-((3,4,5-trimethoxyphenyl)diazenyl)quinolin-8-ol (**S9**)

By Standard Procedure A, 3,4,5-trimethoxyaniline (366 mg, 2.00 mmol) was reacted with commercial 8-hydroxyquinoline (300 mg, 2.07 mmol). Chromatography of the red crude solid on 1:1:0→1:5:0→1:5:0.3 Hx:EA:MeOH returned **S9** (514 mg, 1.52 mmol, 76%; R_f = 0.07 on 1:5 Hx:EA, $FeCl_3$) as a deep orange solid.

^{1}H NMR (400 MHz, $CDCl_3$): δ = 9.20 (dd, *J* = 8.5 Hz, 1.4 Hz, 1H), 8.81 (dd, *J* = 4.2 Hz, 1.6 Hz, 1H), 7.95 (d, *J* = 8.3 Hz, 1H), 7.56 (dd, *J* = 8.6 Hz, 4.1 Hz, 1H), 7.26 (s, 2H), 7.20 (~d overlapped, *J* = 7 Hz, 1H), 3.93 (s, 6H), 3.88 (s, 3H) ppm. ^{13}C NMR (100 MHz, $CDCl_3$) showed 2 isomers in >4:1 ratio, only the major isomer's peaks are reported: δ = 155.2, 153.6 (×2), 149.0, 148.5, 140.5, 139.9, 137.7, 132.9, 127.0, 122.8, 115.6, 110.1, 100.3 (×2), 61.1, 56.3 (×2) ppm. HRMS (ESI+) calcd for $[C_{18}H_{18}N_3O_4]^+$ = $[MH]^+$: m/z 340.12918, found 340.12898. LCMS(+): t_{ret} = 4.02 min, 340 Th = $[MH]^+$.

8-methoxy-5-((3,4,5-trimethoxyphenyl)diazenyl)quinoline (**PST-20**)

By Standard Procedure B, **S9** (505 mg, 1.49 mmol) was methylated overnight. Chromatography of the red crude on 1:5:0→1:5:1 Hx:EA:MeOH returned **PST-20** (422 mg, 1.20 mmol, 80%; R_f = 0.32 on 1:5:0.1 Hx:EA:MeOH, $FeCl_3$) as a brown solid.

^{1}H NMR (400 MHz, $CDCl_3$): δ = 9.32 (d, *J* = 8.4 Hz, 1H), 9.10 – 9.05 (m, 1H), 7.99 (d, *J* = 8.6 Hz, 1H), 7.70 – 7.62 (m, 1H), 7.32 (s, 2H), 7.18 (d, *J* = 8.6 Hz, 1H), 4.20 (s, 3H), 4.00 (s, 6H), 3.96 (s, 3H) ppm. ^{13}C NMR (100 MHz, $CDCl_3$): δ = 157.64, 153.75, 149.54, 149.14, 140.88, 140.87, 133.16, 127.98, 122.61, 114.13, 107.80, 100.63, 61.25, 56.64, 56.40 ppm. LCMS(+): t_{ret} = 2.69 & 3.55 min, each 354 Th = $[MH]^+$: these peaks were assigned to the *cis* & *trans* isomers respectively since the UV absorption profile of the first peak (*cis*) featured a very broad secondary band centred around 400 nm and extending to 480 nm at half-maximum, which was absent in the second peak. HRMS (ESI+) calcd for $[C_{19}H_{20}N_3O_4]^+$ = $[MH]^+$: m/z 354.14483, found 354.14462.

PST-21

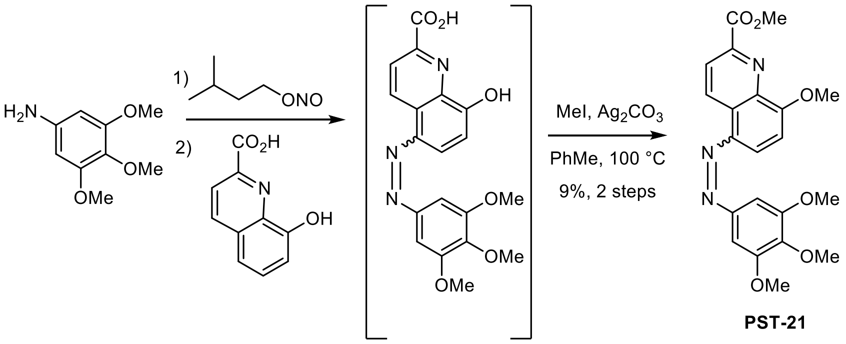

methyl 8-methoxy-5-((3,4,5-trimethoxyphenyl)diazenyl)quinoline-2-carboxylate (**PST-21**)

By Standard Procedure A, ,4,5-trimethoxyaniline (187 mg, 1.02 mmol) was reacted with commercial 8-hydroxyquinoline-2-carboxylic acid (195 mg, 1.03 mmol), using EtOAc as the extraction solvent while maintaining the aqueous phase at pH = 3. Pad filtration of the black crude on 1:0 → 1:1 $CHCl_3$:MeOH removed both fast-running and immobile crude components, and the resultant black crude containing residual starting material and other contaminants was carried over to the next step directly (247 mg; LCMS(+): t_{ret} = 4.02 min, 384 Th = $[MH]^+$). The crude (120 mg) was methylated by Standard Procedure C using MeI (88 mg, > 2 equivalents). Chromatography on 5:1:0 → 1:1:0 → 1:1:1 Hx:EA:MeOH returned **PST-21** (19 mg, 0.046 mmol; 9% over 2 steps; R_f = 0.27 on 1:5 Hx:EA, UV) as an orange oil.

^1H NMR (400 MHz, $CDCl_3$) without precautions to block ambient light revealed a 3:1 proportion of [presumably *trans*:*cis*] isomers: δ = 9.37 (d, *J* = 8.9 Hz, $1H_Z$), 9.34 (d, *J* = 8.8 Hz, $1H_E$), 9.04 (d, 8.7 Hz, $1H_Z$), 8.33 (d, *J* = 8.8 Hz, $1H_E$), 8.29 (d, *J* = 8.8 Hz, $1H_Z$), 7.99 (d, *J* = 8.6 Hz, $1H_E$), 7.58 (s, $2H_Z$), 7.25 (s, $2H_E$), 7.13 (d, *J* = 8.6 Hz, $1H_E$), 7.10 (d, *J* = 8.9 Hz, $1H_Z$), 4.12 (s, $3H_E$), 4.11 (s, $3H_Z$), 4.02 (s, $3H_E$), 4.01 (s, $3H_Z$), 3.94 (s, $6H_E$), 3.94 (s, $6H_Z$), 3.89 (s, $3H_E$), 3.88 (s, $3H_Z$) ppm. ^{13}C NMR (100 MHz, $CDCl_3$; only major isomer peaks are reported): δ = 165.98, 158.50, 153.74, 149.04, 147.30, 140.96, 140.59, 139.20, 133.75, 129.03, 122.43, 116.08, 107.96, 100.66, 61.23, 56.72, 56.40, 53.33 ppm. HRMS (ESI+) calcd for $[C_{21}H_{22}N_3O_6]^+$ = $[MH]^+$: m/z 412.15031, found 412.15036. LCMS(+): t_{ret} = 3.61 & 4.47 min, 412 Th = $[MH]^+$: these peaks were assigned to the *cis* & *trans* isomers respectively since the UV absorption profile of the first peak (*cis*) featured a secondary band centred around 455 nm.

PST-22

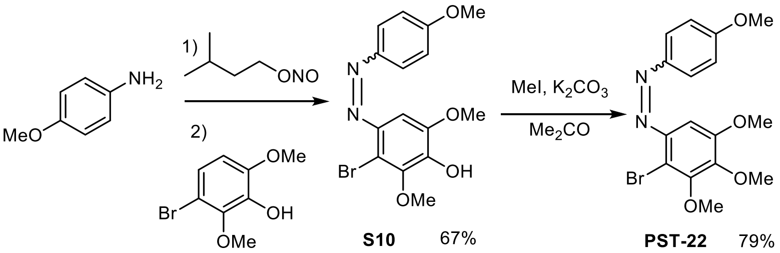

3-bromo-2,6-dimethoxy-4-((4-methoxyphenyl)diazenyl)phenol (**S10**)

4-Methoxyaniline (246 mg, 2 mmol) was diazotized and coupled to 3-Bromo-2,6-dimethoxyphenol (466 mg, 2 mmol) following Standard Procedure A. Chromatography on 5:1→2.4:1 Hx:EA. returned **S10** (480 mg, 1.31 mmol, 67%; R_f = 0.54 on 1:1 Hx:EA) as an red solid.

^{1}H NMR (400 MHz, $CDCl_3$) δ = 8.00 – 7.95 (m, 2H), 7.27 (s, 1H), 7.06 – 7.01 (m, 2H), 4.03 (s, 3H), 3.99 (s, 3H), 3.92 (s, 3H) ppm. ^{13}C NMR (100 MHz, $CDCl_3$) δ = 162.20, 147.33, 147.18, 144.69, 142.54, 142.03, 125.20, 114.94, 114.40, 95.69, 61.07, 56.51, 55.74 ppm. LCMS(+): t_{ret}= 4.75 (E), 367 Th = $[MH]^+$. HRMS (ESI+): Calculated for $[C_{15}H_{15}N_2O_5]^+$ = $[MH]^+$: m/z 367.02880, found 367.02843.

1-(2-bromo-3,4,5-trimethoxyphenyl)-2-(4-methoxyphenyl)diazene (**PST-22**)

S10 (470 mg, 1.3 mmol) was methylated following Standard Procedure B. Chromatography on 5:1 Hx:EA returned **PST-22** (392 mg, 1.03 mmol, 79%; R_f = 0.39 on 5:1 Hx:EA) as an orange oil.

^{1}H NMR (400 MHz, $CDCl_3$) δ = 8.08 – 8.02 (m, 2H), 7.35 (s, 1H), 7.13 – 7.07 (m, 2H), 4.06 (s, 3H), 4.05 (s, 3H), 4.01 (s, 3H), 3.99 (s, 3H) ppm. ^{13}C NMR (100 MHz, $CDCl_3$) δ = 162.49, 153.13, 151.42, 147.16, 145.64, 145.29, 125.44, 114.43, 113.97, 96.38, 61.48, 61.34, 56.29, 55.77 ppm. LCMS(+): t_{ret}=4.88 (Z) + 5.33 (E), 381 Th = $[MH]^+$. HRMS (ESI+): Calculated for $[C_{16}H_{18}BrN_2O_4]^+$ = $[MH]^+$: m/z 381.04445, found 381.04415.

PST-23

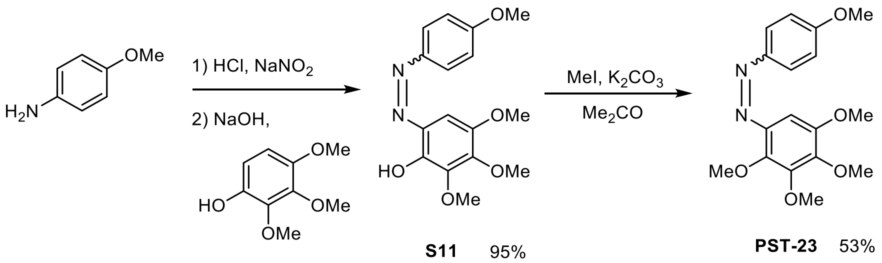

2,3,4-trimethoxy-6-((4-methoxyphenyl)diazenyl)phenol (**S11**)

Commercial 4-methoxyaniline (100 mg, 0.813 mmol) was dissolved in aqueous HCl (2 M, 1.22 mL), diluted with water (7 mL) and cooled in an icebath while a cold aqueous solution of $NaNO_2$ (2.3 M, 0.4 mL, 1.1 eq) was added dropwise with stirring. Stirring in the icebath was continued for 6 minutes, then a cold solution of 2,3,4-trimethoxyphenol (224 mg, 1.22 mmol, synthesised as per literature[58]) in methanol (6 mL) was added followed by a cold aqueous solution of NaOH until pH 10 was reached. The aqueous phase was extracted with ethyl acetate (3×20 mL), then the combined organic layers were washed with brine (60 mL), dried on Na_2SO_4, filtered and concentrated to yield a deep red crude oil. Chromatography on on 15:1→1:1 Hx:EA returned **S11** as a yellow oil (248 mg, 0.780 mmol, 95%; R_f = 0.55 on 2.5:1 Hx:EA).

^{1}H NMR (400 MHz, $CDCl_3$): δ = 13.33 (s, 1H), 7.80 (d, *J* = 9.0 Hz, 2H), 7.17 (s, 1H), 7.01 (d, *J* = 9.0 Hz, 2H), 4.05 (s, 3H), 3.98 (s, 3H), 3.92 (s, 3H), 3.89 (s, 3H) ppm. ^{13}C NMR (100 MHz, $CDCl_3$): δ = 161.8, 146.5, 146.0, 144.3, 143.4, 141.3, 133.0, 123.6, 114.6, 109.0, 61.4, 61.1, 56.4, 55.6 ppm. LCMS(+): t_{ret} = 8.64 min, 319 Th = $[MH]^+$. HRMS (ESI+) calculated for $[C_{16}H_{19}N_2O_5]^+$ = $[M{\cdot}H^+]$: m/z 319.12885, found 319.12872.

1-(4-methoxyphenyl)-2-(2,3,4,5-tetramethoxyphenyl)diazene (**PST-23)**

S11 (127.1 mg, 0.400 mmol) was added to a mixture of MeI (554 mg, 3.92 mmol) and MeCN (10mL). K_2CO_3 (216 mg, 1.56 mmol) was added and the mixture heated to 60°C and stirred for 12 hours. The solid was removed and the aqueous phase was extracted with ethyl acetate (3×20 mL), then the combined organic layers were washed with brine (60 mL), dried on Na_2SO_4, filtered and concentrated. Chromatography on on 5:1→1:1 Hx:EA returned **PST-23** as a yellow oil (69.6 mg, 0.210 mmol, 53%; R_f = 0.48 and 0.29 on 2.5:1 Hx:EA).

^{1}H NMR (400 MHz, $CDCl_3$): δ = 7.85 (d, *J* = 9.0 Hz, 2H), 7.03 (s, 1H), 6.93 (d, *J* = 9.0 Hz, 2H), 3.99 (s, 3H), 3.93 (s, 3H), 3.90 (s, 3H), 3.82 (s, 3H), 3.81 (s, 3H) ppm. ^{13}C-NMR (100 MHz, $CDCl_3$): δ = 161.9, 149.6, 147.3, 147.2, 145.6, 141.3, 124.8, 114.2, 106.4, 94.0, 63.9, 61.7, 61.4, 56.1, 55.6 ppm. LCMS(+): t_{ret} = 7.12 & 8.53 min, each 333 Th = $[MH]^+$: these peaks were assigned to the Z & E isomers respectively. HRMS (EI+) calculated for $[C_{17}H_{20}N_2O_5]^+$ = $[M]^+$: m/z 332.1372, found 332.1354.

PST-24

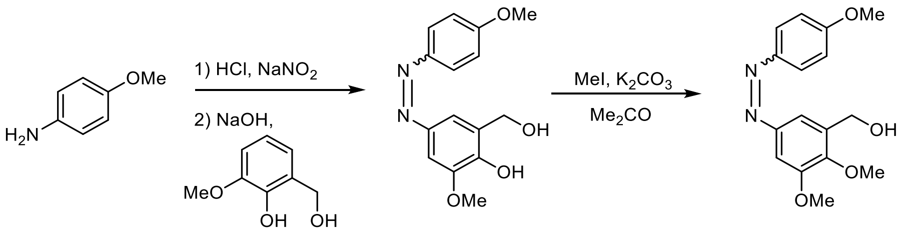

S12 51% **PST-24** 78%

o-vanillyl alcohol

$NaBH_4$ (1 g, 27 mmol) was added portionwise over 20 min to a solution of *o*-vanillin (7.6 g, 50 mmol) in MeOH (100 mL) at 0 °C. After 20 min, water (3 mL) was added, and the solution filtered and evaporated and filtered over silica pad (EA) to give pale yellow product (5.0 g, 32 mmol, 64%). The NMR matched literature data.[59]

2-(hydroxymethyl)-6-methoxy-4-((4-methoxyphenyl)diazenyl)phenol (**S12**)

p-Anisidine (2.6 g, 16.9 mmol) was dissolved in MeOH (20 mL) and conc HCl (5 mL) was added. The solution was cooled in an ice-bath and $NaNO_2$ solution (2.3 M, 9 mL) was added. After 15 min stirring, the diazonium solution was added dropwise to a cold suspension of *o*-vanillyl alcohol (3.05 g) in MeOH (60 mL) and NaOAc solution (2 M, 60 mL). After 2 h stirring, the solution was extracted with $CHCl_3$ (3 x 20 mL), washed with water (2 x 20 mL) and brine (25 mL), dried over Na_2SO_4, filtered, and chromatographed on 5:1→1:1 Hx:EA gradient to yield the product (2.47 g, 8.6 mmol, 51%) as a yellow solid (R_f = 0.33 on 1:1 Hx:EA; LCMS(+): t_{ret} = 6.39 min, 289 Th = $[MH]^+$.

^{1}H NMR (400 MHz, $CDCl_3$) δ = 7.94 – 7.86 (m, 2H), 7.59 (d, *J* = 2.2 Hz, 1H), 7.47 (d, *J* = 2.2 Hz, 1H), 7.07 – 6.98 (m, 2H), 6.38 (s, 1H), 4.87 (s, 2H), 4.02 (s, 3H), 3.91 (s, 3H), 2.30 (s, 1H) ppm. ^{13}C NMR (100 MHz, $CDCl_3$) δ = 161.63, 147.05, 146.90, 146.16, 146.11, 126.29, 124.37, 119.05, 114.21, 101.96, 61.60, 56.25, 55.58 ppm. HRMS (ESI+) calculated for $[C_{15}H_{17}N_2O_4]^+$ = $[MH]^+$: m/z 289.11828, found 289.11828.

(2,3-dimethoxy-5-((4-methoxyphenyl)diazenyl)phenyl)methanol (**PST-24**)

To **S13** (2.00g, 6.9 mmol) were added K_2CO_3 (2.87 g, 20.8 mmol), acetone (40 mL), MeI (1.28 g, 9.0 mmol, 1.3 eq) and the mixture was refluxed for 1 h. The solution was evaporated and chromatographed on 6:1→2:1 Hx:EA yielding **PST-24** (1.65 g, 5.5 mmol, 78%) as a yellow solid (Rf = 0.43 on 2:1 Hx:EA).

^{1}H NMR (400 MHz, $CDCl_3$) δ = 7.95 – 7.88 (m, 2H), 7.59 (d, *J* = 2.2 Hz, 1H), 7.48 (d, *J* = 2.2 Hz, 1H), 7.07 – 7.01 (m, 2H), 4.81 (d, *J* = 6.3 Hz, 2H), 3.99 (s, 3H), 3.98 (s, 3H), 3.91 (s, 3H) ppm. ^{13}C NMR (100 MHz, $CDCl_3$) δ = 161.9, 152.9, 149.0, 148.9, 146.9, 134.7, 124.6, 117.3, 114.2, 104.2, 61.4, 61.1, 55.9, 55.6 ppm. LCMS(+): t_{ret} = 5.75 & 7.22 min, each 303 Th = $[MH]^+$. HRMS (ESI+) calculated for $[C_{16}H_{19}N_2O_4]^+$ = $[MH]^+$: m/z 303.13393, found 303.13383.

PST-25

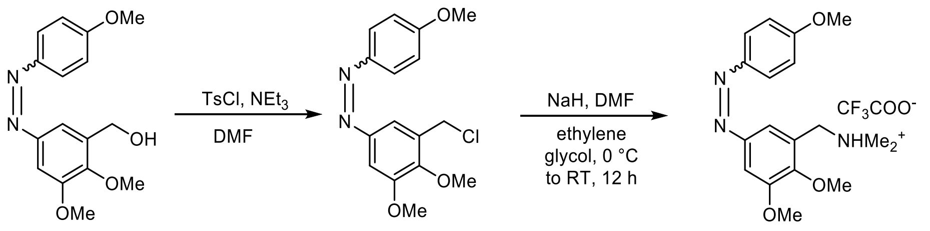

PST-24 **S13** 65% **PST-25** 79%

1-(3-(chloromethyl)-4,5-dimethoxyphenyl)-2-(4-methoxyphenyl)diazene (**S13**)

PST-24 (605 mg, 2.0 mmol) was dissolved in DMF (15 mL) and at 0 °C NEt3 (0.7 mL) and TsCl (0.85 g) were added. After 12 h stirring at room temperature, the solution was diluted with water (150 mL), extracted with CHCl3 (2 x 20 mL), washed with water (20 mL) and brine (15 mL), dried over Na2SO4, filtered, and chromatographed on 20:1→5:1 Hx:EA gradient to yield **S13** (417 mg, 1.3 mmol, 65%) as an orange oil (Rf = 0.54 on 5:1 Hx:EA, t_{ret} = 5.86 & 8.12 min ($[MH]^+$= 321/323 Th) on 40/100).

^{1}H NMR (400 MHz, $CDCl_3$) δ = 7.93 (d, *J* = 9.0 Hz, 2H), 7.64 (d, *J* = 2.2 Hz, 1H), 7.50 (d, *J* = 2.2 Hz, 1H), 7.04 (d, *J* = 9.0 Hz, 2H), 4.74 (s, 2H), 4.02 (s, 3H), 3.99 (s, 3H), 3.92 (s, 3H) ppm. ^{13}C NMR (100 MHz, $CDCl_3$) δ = 162.0, 153.2, 149.3, 148.9, 146.8, 131.6, 124.7, 119.1, 114.3, 104.8, 61.4, 56.0, 55.6, 41.1 ppm. HRMS (EI) calculated for $[C_{16}H_{17}ClN_2O_3]^+$ = $[M]^+$: m/z 320.0928, found 320.0919.

1-(2,3-dimethoxy-5-((4-methoxyphenyl)diazenyl)phenyl)-N,N-dimethylmethanammonium 2,2,2-trifluoroacetate (**PST-25**)

S13 (40 mg, 0.12 mmol) was dissolved in DMF (3 mL) and ethylene glycol (6 mL) and added to a mixture of sodium hydride (140 mg, 3.5 mmol, 60% in mineral oil) in ethylene glycol (5 mL) at 0 °C. After warming to RT the solution was stirred at room temperature for 12 h. The solution was diluted with aqueous NaOH (2 M, 50 mL), extracted with EA (3 × 20 mL), washed with water (20 mL). The organic layer was washed with aqueous HCl (2 M, 3 × 20 mL). The combined aqueous phase was basified with aqueous NaOH (3 M, 60 mL), extracted with EA (3 × 20 mL) washed with water (50 mL) and brine (50 mL), dried over Na_2SO_4, filtered, and concentrated to yield a crude oil which was dissolved in hexanes and triturated with TFA to precipitate a red solid, which was washed with hexanes and dried to yield **PST-25** (42 mg, 0.094 mmol, 79%) as the TFA salt.

^{1}H NMR (400 MHz, $CDCl_3$) δ = 11.68 (s, 1H), 7.92 (d, *J* = 8.8 Hz, 2H), 7.60 (s, 2H), 7.03 (d, *J* = 8.9 Hz, 2H), 4.37 (s, 2H), 4.00 (s, 3H), 3.99 (s, 3H), 3.91 (s, 3H), 2.85 (s, 6H) ppm. ^{13}C NMR (100 MHz, $CDCl_3$) δ = 162.46, 161.45 (q, *J* = 37.8 Hz), 153.16, 150.12, 148.90, 146.76, 125.01, 121.56, 120.46 123.66 – 113.45 (m), 114.42, 106.80, 61.44, 56.13, 55.72, 42.38 ppm. LCMS(+): t_{ret} = 1.69 & 3.31 min ($[MH]^+$ = 330 Th) on 30/100, HRMS (ESI+) calculated for $[C_{18}H_{24}N_3O_3]^+$ = $[MH]^+$: m/z 330.18122, found 330.18154.

PST-26

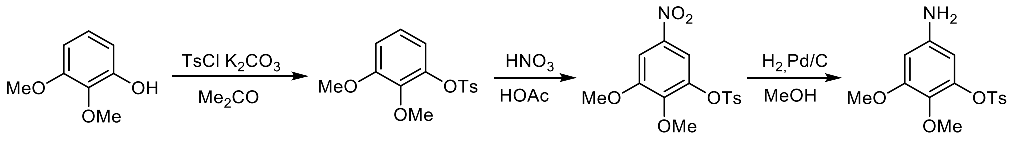

2,3-dimethoxyphenyl 4-methylbenzenesulfonate (**S14**)

2,3-dimethoxyphenol (530 mg, 3.44 mmol) and 4-toluenesulfonyl chloride (787 mg, 4.12 mmol) and potassium carbonate (2.37 g, 17.2 mmol) were stirred in acetone (50 mL) for 12 h at 50 °C. The volatiles were evaporated, the residue partitioned between pH 7 phosphate buffer and EtOAc, washed with brine, dried over Na_2SO_4 and concentrated. The crude was purified on 5:1→1:1 Hx:EA gradient. **S14** (0.96 g, 3.1 mmol, 90%) was obtained as a solid (R_f = 0.36 on 2.4:1 Hx:EA).

NMR matched literature data.[60] LCMS(+): t_{ret} = 5.31 min, 309 Th = $[MH]^+$.

2,3-dimethoxy-5-nitrophenyl 4-methylbenzenesulfonate (**S15**)

S14 (502 mg, 1.63 mmol) was dissolved in glacial acetic acid (5 mL) and fuming nitric acid (1 mL) was added at 0 °C. The reaction mixture was warmed up to room temperature and stirred for 14 h. Saturated sodium bicarbonate solution was added until the acid was neutralized and the mixture was extracted using ethyl acetate. The crude product was purified by chromatography on 5:1→1:1 Hx:EA gradient to yield **S15** (186 mg, 0.43 mmol, 70%) as a yellow solid (R_f = 0.43 on 1:1 Hx:EA).

^{1}H NMR (400 MHz, $CDCl_3$) δ = 7.82 (d, *J* = 8.3 Hz, 2H), 7.72 (d, J = 2.5 Hz, 1H), 7.61 (d, *J* = 2.5 Hz, 1H), 7.39 (d, *J* = 8.0 Hz, 2H), 3.95 (s, 3H), 3.88 (d, J = 1.6 Hz, 3H), 2.49 (s, 3H) ppm. ^{13}C NMR (100 MHz, $CDCl_3$) δ = 153.3, 148.0, 146.0, 142.3, 141.7, 132.5, 130.0, 128.4, 112.2, 106.3, 61.5, 56.6, 21.8 ppm.

5-amino-2,3-dimethoxyphenyl 4-methylbenzenesulfonate (**S16**)

S15 (981 mg, 2.78 mmol) and palladium on charcoal (10% Pd/C, 294 mg, 0.28 mmol), was dissolved in methanol (25 mL) and degassed. The flask was evacuated, backfilled with hydrogen and stirred under a hydrogen atmosphere for 14 h. The reaction mixture was filtered over a celite and washed with ethyl acetate. The crude product was purified by chromatography on 2.4:1→1:1 Hx:EA gradient to yield **S16** (513 mg, 1.59 mmol, 57%) as a yellow solid (R_f = 0.37 on 1:1 Hx:EA).

^{1}H NMR (400 MHz, $CDCl_3$) δ = 7.84 (d, *J* = 8.0 Hz, 2H), 7.33 (d, *J* = 8.0 Hz, 2H), 6.22 (d, *J* = 2.4 Hz, 1H), 6.16 (d, *J* = 2.5 Hz, 1H), 3.79 (s, 3H), 3.60 (m, 5H), 2.46 (s, 3H) ppm. ^{13}C NMR (100 MHz, $CDCl_3$) δ = 154.2, 145.1, 143.4, 142.5, 134.0, 133.2, 129.6, 128.4, 101.8, 98.6, 61.2, 55.9, 21.8 ppm. LCMS(+): t_{ret} = 4.30 min, 324 Th = $[MH]^+$.

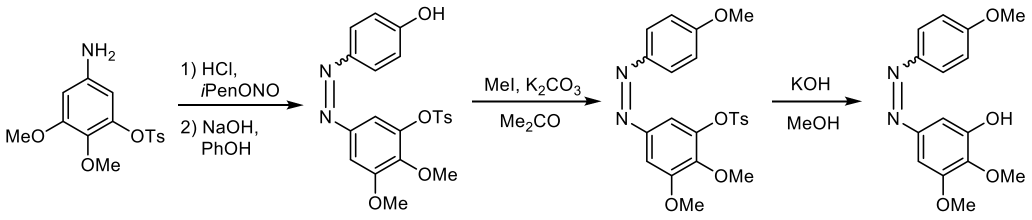

S17 70% **S18** 64% **PST-26** 43%

5-((4-hydroxyphenyl)diazenyl)-2,3-dimethoxyphenyl 4-methylbenzenesulfonate (**S17**)

S16 (200 mg, 0.62 mmol) was diazotized and coupled to phenol (61 mg, 0.65 mmol) following standard procedure A. The crude product was purified by column chromatography using 5:1→1:1 Hx:EA gradient to yield **S17** (186 mg, 0.43 mmol, 70%) as a yellow solid (R_f = 0.15 on 2.4:1 Hx:EA).

^{1}H NMR (400 MHz, $CDCl_3$) δ = 7.86 (dd, *J* = 8.2 Hz, 5.3 Hz, 4H), 7.43 (dd, *J* = 2.9 Hz, 1.7 Hz, 2H), 7.36 (d, *J* = 8.0 Hz, 2H), 6.97 (d, *J* = 8.5 Hz, 2H), 3.95 (s, 3H), 3.81 (s, 3H), 2.48 (s, 3H). ^{13}C NMR (100 MHz, $CDCl_3$) δ = 158.6, 153.8, 147.8, 146.7, 145.4, 143.7, 142.8, 133.0, 129.8, 128.5, 125.1, 115.9, 112.0, 103.7, 61.3, 56.2, 21.8 ppm. HRMS (ESI+): Calculated for $[C_{21}H_{21}N_2O_6S]^+$ = $[MH]^+$: m/z 429.11148, found 429.11167.

2,3-dimethoxy-5-((4-methoxyphenyl)diazenyl)phenyl 4-methylbenzenesulfonate (**S18**)

S17 (180 mg, 0.42 mmol) was methylated as described in standard procedure B. The crude product was purified by column chromatography using 5:1→2.4:1 Hx:EA gradient to yield **S18** (120 mg, 0.27 mmol, 64%) as a yellow solid (R_f = 0.33 (*E*), 0.11 (*Z*) on 2.4:1 Hx:EA).

^{1}H NMR (400 MHz, $CDCl_3$) δ 7.88 (dd, *J* = 14.7 Hz, 8.3 Hz, 4H), 7.43 (s, 2H), 7.36 (d, *J* = 8.0 Hz, 2H), 7.04 (d, *J* = 8.7 Hz, 2H), 3.95 (s, 3H), 3.93 (s, 3H), 3.81 (s, 3H), 2.48 (s, 3H). ^{13}C NMR (100 MHz, $CDCl_3$) δ = 162.2, 153.8, 147.9, 146.6, 145.3, 143.7, 142.9, 133.1, 129.7, 128.5, 124.9, 114.3, 112.0, 103.7, 61.3, 56.2, 55.6, 21.8. HRMS (ESI+): Calculated for $[C_{22}H_{23}N_2O_6S]^+$ = $[MH]^+$: m/z 443.12713, found 443.12713.

2,3-dimethoxy-5-((4-methoxyphenyl)diazenyl)phenol (**PST-26**)

S18 (320 mg, 0.72 mmol) and KOH (410 mg, 7.3 mmol) were heated to 50 °C in 20 mL of methanol. After satisfactory conversion of the starting material, methanol was removed in vacuo and the crude product was purified by column chromatography using 5:1→2.4:1 Hx:EA gradient to yield **PST-26** (89 mg, 0.31 mmol, 43%) as a yellow solid (R_f = 0.35 on 2.4:1 Hx:EA).

^{1}H-NMR (400 MHz, $CDCl_3$) δ = 7.92 (d, *J* = 8.7 Hz, 2H), 7.24 (d, *J* = 2.1 Hz, 1H), 7.13 (d, *J* = 2.1 Hz, 1H), 7.04 (d, *J* = 8.7 Hz, 2H), 5.91 (s, 1H), 4.00 (s, 3H), 3.99 (s, 3H), 3.92 (s, 3H) ppm. ^{13}C NMR (100 MHz, $CDCl_3$) δ = 162.0, 152.5, 149.4, 149.0, 146.8, 137.5, 124.7, 114.2, 103.6, 98.3, 61.2, 56.0, 55.6 ppm. HRMS (ESI+): Calculated for $[C_{15}H_{17}N_2O_4]^+$ = $[MH]^+$: m/z 289.11828, found 289.11817.

PST-27 & PST-27P

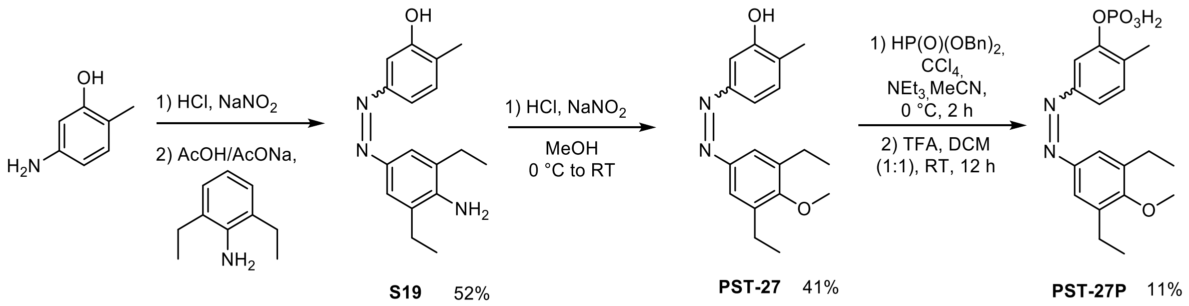

5-((4-amino-3,5-diethylphenyl)diazenyl)-2-methylphenol (**S19**)

2-methyl-5-aminophenol (500 mg, 4.1 mmol, 1.0 eq) was dissolved in MeOH and diazotized with $HCl/NaNO_2$ at 0°C as in Standard Procedure D. Then, H_3NSO_3 (48 mg, 0.1 eq) was dissolved in the minimum volume of water and added to the reaction mixture to scavenge the remaining nitrous acid. Glacial acetic acid (5 mL) was then added to the mixture. A methanolic solution of 2,6-diethylaniline (1.22 g, 8.2 mmol, 2.0 eq) was then added to the diazonium solution. Solid sodium acetate (4.1 g) was dissolved in water (5 mL) and added dropwise to the reaction mixture, which was left stirring for 2 hours. Standard work-up followed, extracting the crude product from aqueous acid (pH ≈ 2) into EA to remove aniline starting material. Chromatography on silica using a gradient from 10:1 to 2.4:1 Hx:EA returned a product which was triturated with Hx to give **S20** as a brown solid (605 mg, 2.1 mmol, 52%, R_f = 0.44 on 2.4:1 Hx:EA).

^{1}H NMR (400 MHz, DMSO-d_6): δ = 9.62 (s, 1H), 7.45 (s, 2H), 7.23 (d, *J* = 1.4 Hz, 1H), 7.20–7.18 (m, 2H), 5.51 (s, 2H), 2.57 (q, *J* = 7.4 Hz, 4H), 2.18 (s, 3H), 1.21 (t, *J* = 7.4 Hz, 6H). ^{13}C NMR (100 MHz, DMSO-d_6): δ = 156.3, 152.4, 147.6, 143.2, 131.2, 126.8 (×2), 126.5, 121.6 (×2), 115.5, 105.8, 24.1 (×2), 16.5, 13.5 (×2). LCMS(+): t_{ret} = 7.06 min, 284 Th = $[MH]^+$. HRMS (ESI+) calcd for $[C_{17}H_{22}N_3O]^+ = [M+H^+]^+$: m/z 284.17574, found 284.17588.

5-((3,5-diethyl-4-methoxyphenyl)diazenyl)-2-methylphenol (**PST-27**)

S20 (100 mg, 0.35 mmol, 1.0 eq) was dissolved in MeOH (4 mL) and diazotized at 0 °C, adding conc. HCl (5 drops) and 0.18 mL (1.2 eq) of an aqueous 2.3 M $NaNO_2$ solution. The resulting solution was left stirring overnight at RT to allow the diazonium to decompose. After 12 h, the reaction progress was checked by LCMS. Then, 5 mL of a K_2HPO_4/K_3PO_4 buffer was added to neutralize HCl and MeOH was removed in vacuo. Extraction with EA (2×5 mL) and concentration gave a deep brown oil. Chromatography on silica using a 5:1 → 1:1 Hx:EA gradient gave the product as an orange oil (43 mg, 0.14 mmol, 41%, R_f = 0.46, E isomer, and 0.14, Z isomer, on 5:1 Hx:EA).

^{1}H NMR (400 MHz, $CDCl_3$): δ = 7.67 (s, 2H), 7.49 (dd, *J* = 7.9, 1.9 Hz, 1H), 7.35 (d, *J* = 1.9 Hz, 1H), 7.30 – 7.28 (m, 1H), 3.82 (s, 3H), 2.78 (q, *J* = 7.6 Hz, 4H), 2.35 (s, 3H), 1.33 (t, *J* = 7.6 Hz, 6H). ^{13}C NMR (100 MHz, $CDCl_3$): δ = 158.8, 154.4, 152.1, 148.8, 137.9, 131.3 (×2), 127.9, 121.7 (×2), 117.9, 106.5, 61.2, 22.9 (×2), 16.1, 14.7 (×2). LCMS(+): t_{ret} = 7.22 and 8.99 min, each 299 Th = $[MH]^+$. HRMS (ESI+): calcd for $[C_{18}H_{23}N_2O_2]^+ = [M+H^+]^+$: m/z 299.17540, found 299.17550.

5-((3,5-diethyl-4-methoxyphenyl)diazenyl)-2-methylphenyl phosphate (**PST-27P**)

PST-27 (43 mg, 0.14 mmol) was dissolved in MeCN (5 mL), and to it were added NEt_3 (0.1 mL) and CCl_4 (0.1 mL). An excess of dibenzyl phosphite (0.1 mL) dissolved in MeCN (3 mL) was added at 0 °C and the mixture stirred for 2 h. The mixture was evaporated and filtered through a silica plug with 10:1 Hx:EA to remove starting materials, then with 3:1 Hx:EA to yield the crude benzyl phosphate (24 mg at ≈60wt% purity, contaminated with **PST-27**). The crude was dissolved in 1:1 DCM:TFA (20 mL) and stirred capped under air for 12 h. The volatiles were evaporated and the residue partitioned between DCM (15 mL) and sat. aq. $NaHCO_3$ (20 mL). The organic layer was evaporated to recover **PST-27** (12 mg), and the aqueous layer was washed with EtOAc (10 mL), then acidified to pH ≈ 3 with aq. HCl, and extracted with EtOAc (2 × 10 mL). The combined organic layers were washed with brine, dried on Na_2SO_4, filtered, and concentrated to yield phosphoric acid **PST-27P** (6 mg, 0.016 mmol, 11%) as a yellow powder that required no further purification.

^{1}H NMR (400 MHz, D_2O) δ = 7.74 (m, 1H), 7.64 (s, 2H), 7.46 (dd, *J* = 8.0, 2.1 Hz, 1H), 7.38 (d, *J* = 8.1 Hz, 1H), 3.82 (s, 3H), 2.73 (q, *J* = 7.6 Hz, 4H), 2.32 (s, 3H), 1.23 (t, *J* = 7.5 Hz, 6H) ppm. LCMS(-): t_{ret} = 5.22 & 6.00 min, each 377 Th = $[M\text{-}H]^-$, attributed to the Z and E isomers respectively, since the absorption spectrum of the former featured a minimum at 380 nm and the latter a maximum at 347 nm. HRMS (ESI-): calcd for $[C_{18}H_{22}N_2O_5P]^-$ = $[M\text{-}H]^-$: m/z 377.12718, found 377.12737.

PST-28

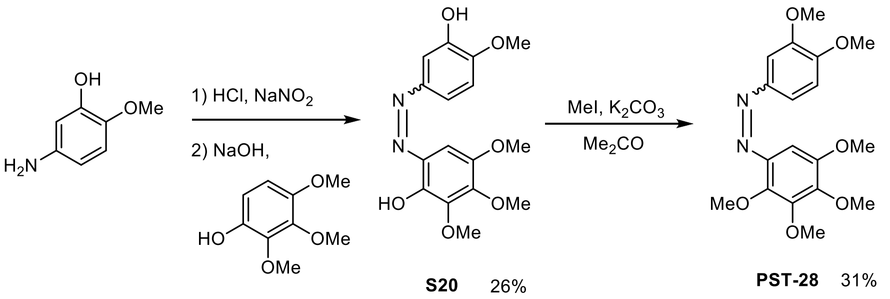

6-((3-hydroxy-4-methoxyphenyl)diazenyl)-2,3,4-trimethoxyphenol (**S20**)

5-amino-2-methoxyphenol (200 mg, 1.44 mmol) was dissolved in aqueous HCl (2 M, 0.88 mL), diluted with water (5 mL) and cooled in an ice bath while a cold aqueous solution of $NaNO_2$ (2.3 M, 0.7 mL, 1.1 eq) was added dropwise with stirring. Stirring in the ice bath was continued for 6 minutes, then a cold solution of 2,3,4-trimethoxyphenol (291 mg, 1.58 mmol) in methanol (6.2 mL) was added followed by a cold aqueous solution of NaOH (2 M, 0.51 mL). The aqueous phase was extracted with ethyl acetate (3 × 20 mL), then the combined organic layers were washed with brine (60 mL), dried on Na_2SO_4, filtered and concentrated to yield a deep red crude oil. Chromatography on 5:1→1:1 Hx:EA returned **S20** as a yellow oil (125 mg, 0.37 mmol, 26%; R_f = 0.50 on 1:1 Hx:EA).

^{1}H NMR (400 MHz, $CDCl_3$): δ = 13.31 (s, 1H), 7.47 (d, *J* = 2.3 Hz, 1H), 7.35-7.38 (dd, *J* = 8.5 Hz, 2.3 Hz, 1H), 7.15 (s, 1H), 6.94 (d, *J* = 8.6 Hz, 1H), 4.01 (s, 3H), 3.98 (s, 3H), 3.96 (s, 3H), 3.91 (s, 3H) ppm. ^{13}C NMR (100 MHz, $CDCl_3$): δ = 149.1, 146.6, 146.6, 146.0, 144.8, 143.7, 141.3, 132.9, 117.8, 110.3, 109.1, 105.2, 61.4, 61.1, 56.4, 56.2 ppm. LCMS(+): t_{ret} = 7.13 min, 335 Th = $[MH]^+$. HRMS (ESI+) calculated for $[C_{16}H_{19}N_2O_6]^+$ = $[M{\cdot}H^+]$: m/z 335.12376, found 335.12368.

1-(3,4-dimethoxyphenyl)-2-(2,3,4,5-tetramethoxyphenyl)diazene (**PST-28**)

To **S20 (**109 mg, 0.317 mmol) was added to a mixture of MeI (120 mg, 0.85 mmol) and MeCN (5 mL). K_2CO_3 (180 mg, 1.30 mmol) was added, and the mixture was stirred for 12 hours. The solid was removed and the aqueous phase was extracted with ethyl acetate (3×20 mL), then the combined organic layers were washed with brine (60 mL), dried on Na_2SO_4, filtered and concentrated. Chromatography on 5:1→1:1 Hx:EA returned **PST-28** as a yellow oil (36 mg, 0.1 mmol, 31%; R_f = 0.59 and 0.38 on 1:1 Hx:EA).

^{1}H NMR (400 MHz, $CDCl_3$): δ = 7.63 (dd, *J* = 8.5, 2.2 Hz, 1H), 7.53 (d, *J* = 2.2 Hz, 1H), 7.10 (s, 1H), 7.00 (d, *J* = 8.7 Hz, 1H), 4.07 (s, 3H), 4.00 (s, 3H), 3.98 – 3.97 (m, 9H), 3.90 (s, 3H) ppm. ^{13}C NMR (100 MHz, $CDCl_3$): δ = 151.88, 149.72, 149.66, 147.43, 147.36, 147.26, 145.76, 141.39, 120.37, 110.55, 102.67, 94.22, 64.01, 61.83, 61.53, 56.27, 56.19, 56.05 ppm. LCMS(+): t_{ret} = 6.61 & 7.94 min, each 363 Th = $[MH]^+$: these peaks were assigned to the Z & E isomers respectively. HRMS (EI+) calculated for $[C_{18}H_{22}N_2O_6]^+$ = $[M]^+$: m/z 362.1478, found 362.1471.

S29, PST-29 & S29-Ac

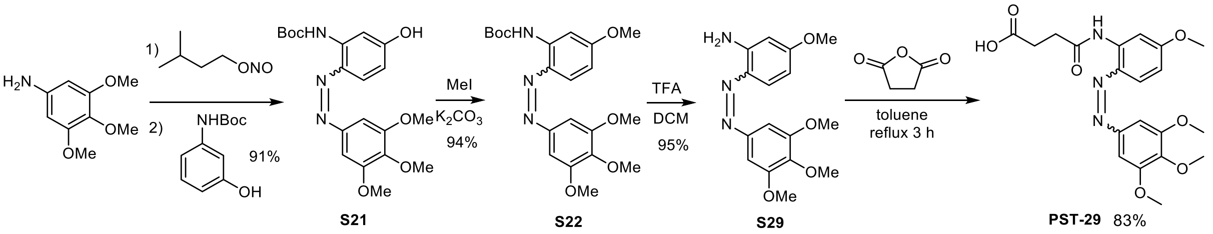

tert-butyl (5-hydroxy-2-((3,4,5-trimethoxyphenyl)diazenyl)phenyl)carbamate (**S21**)

3,4,5-Trimethoxyaniline (916 mg, 5 mmol) was diazotized and coupled to *tert*-butyl-(3-hydroxyphenyl)carbamate (1047 mg, 5 mmol) following Standard Procedure A. Chromatography on 5:1→4:1 Hx:EA returned **S21** (1.84 g, 4.56 mmol, 91%; R_f = 0.44 on 2.4:1 Hx:EA) as an orange solid.

^{1}H NMR (400 MHz, DMSO-d$_6$) δ = 10.44 (s, 1H), 9.84 (s, 1H), 7.81 – 7.55 (m, 2H), 7.17 (s, 2H), 6.57 (dd, *J* = 8.9 Hz, 2.6 Hz, 1H), 3.88 (s, 6H), 3.75 (s, 3H), 1.50 (s, 9H) ppm. ^{13}C NMR (100 MHz, DMSO-d$_6$) δ = 162.3, 153.8, 152.5, 148.4, 139.9, 138.6, 133.1, 123.1, 111.1, 105.6, 100.2, 80.5, 60.7, 56.3, 28.3 ppm. LCMS(+): t_{ret} = 5.16 min, 404 Th = [MH]$^+$. HRMS (ESI+): Calculated for $[C_{20}H_{26}N_3O_6]^+$ = [MH] $^+$: m/z 404.18161, found 404.18188.

tert-butyl (5-methoxy-2-((3,4,5-trimethoxyphenyl)diazenyl)phenyl)carbamate (**S22**)

S21 (1.83 g, 4.54 mmol) was methylated methylated for 6 hours with MeI (1.13 g, 8.0 mmol) and K_2CO_3 (2.2 g, 16 mmol) following Standard Procedure B. Chromatography on 5:1→2.4:1 Hx:EA returned **S22** (1.79 g, 4.29 mmol, 94%; R_f = 0.54 on 2.4:1 Hx:EA) as an orange solid.

^{1}H NMR (400 MHz, $CDCl_3$) δ = 9.84 (s, 1H), 7.98 (d, *J* = 2.7 Hz, 1H), 7.85 (d, *J* = 9.0 Hz, 1H), 7.16 (s, 2H), 6.65 (dd, *J* = 9.0 Hz, 2.7 Hz, 1H), 3.96 (s, 6H), 3.93 (s, 3H), 3.92 (s, 3H), 1.55 (s, 9H) ppm. ^{13}C NMR (100 MHz, $CDCl_3$) δ = 163.8, 153.6, 152.4, 147.9, 140.2, 138.6, 132.8, 124.2, 110.0, 101.8, 99.7, 80.7, 61.1, 56.1, 55.8, 28.3 ppm. LCMS(+): t_{ret} = 5.81 min, 418 Th = [MH]$^+$. HRMS (ESI+): Calculated for $[C_{21}H_{26}N_3O_6Na]^+$ = [MNa]$^+$: m/z 440.17920 found 440.17936.

5-methoxy-2-((3,4,5-trimethoxyphenyl)diazenyl)aniline (**S29**)

To **S22** (1.20 g, 2.87 mmol) were added CH_2Cl_2 (10 mL) and CF_3COOH (12 mL) and the purple solution stirred overnight at room temperature. The volatiles were removed under high vacuum, the purple residue was partitioned between CH_2Cl_2 (30 mL) and K_2HPO_4/KH_2PO_4 buffer (pH = 6.8, 30 mL), the aqueous layer extracted with DCM (15 mL), then the combined organic layers were washed with brine (20 mL), dried on Na_2SO_4, filtered and concentrated to give a red crude oil. Chromatography on 5:1→1:1 Hx:EA returned **S29** as a red oil (870 mg, 2.74 mmol, 95%; R_f = 0.55 on 1:1 Hx:EA ($FeCl_3$)).

^{1}H NMR (400 MHz, CD_3CN): δ = 7.67 (d, *J* = 8.9 Hz, 1H), 7.18 (s, 2H), 6.48 (s, 2H), 6.36 (dd, *J* = 8.9 Hz, 2.7 Hz, 1H), 6.32 (d, 2.6 Hz, 1H), 3.90 (s, 6H), 3.81 (s, 3H), 3.78 (s, 3H) ppm. ^{13}C NMR (100 MHz, CD_3CN): δ = 164.07, 154.73, 150.00, 146.77, 140.08, 132.52, 130.41, 106.15, 100.16, 99.95, 60.97, 56.72, 56.04 ppm. LCMS(+): t_{ret} = 4.35 min, 318 Th = [MH]+. HRMS (ESI+) calcd for $[C_{16}H_{20}N_3O_4]^+$ = [MH$^+$]: m/z 318.1448, found 318.14454.

5-((5-methoxy-2-((3,4,5-trimethoxyphenyl)diazenyl)phenyl)amino)-5-oxopentanoic acid (**PST-29**)

S29 (240 mg, 0.76 mmol) and succinic anhydride (80 mg, 0.8 mmol) were refluxed in 20 mL of toluene for 3 h. Upon cooling to RT a yellow precipitate formed and the solvent was removed in vacuo. The residue was dissolved in 100 mL CH_2Cl_2, washed with 50 mL sat. ammonium chloride solution and dried over Na_2SO_4. Concentration yielded **PST-29** as a green-yellow solid (265 mg, 0.63 mmol, 83%).

^{1}H NMR (400 MHz, DMSO-d_6) δ = 12.17 (s, 1H), 10.25 (s, 1H), 8.00 (d, *J* = 2.7 Hz, 1H), 7.75 (d, *J* = 9.1 Hz, 1H), 7.37 (s, 2H), 6.79 (dd, *J* = 9.1, 2.8 Hz, 1H), 3.90 (s, 6H), 3.84 (s, 3H), 3.75 (s, 3H), 2.77 (dd, *J* = 7.5, 5.6 Hz, 2H), 2.58 (dd, *J* = 7.3, 5.8 Hz, 2H) ppm. ^{13}C NMR (100 MHz, DMSO-d_6) δ = 173.88, 170.89, 162.59, 153.26, 148.16, 139.78, 138.81, 134.42, 118.07, 110.16, 105.66, 100.62, 60.28, 56.04, 55.63, 31.67, 28.96 ppm. LCMS(+): t_{ret} = 2.99 min (Z) + 3.98 min (E), each 418 Th = $[MH]^+$. HRMS (ESI+): Calculated for $[C_{20}H_{24}N_3O_7]^+$ = $[MH]^+$: m/z 418.16088, found 418.16084.

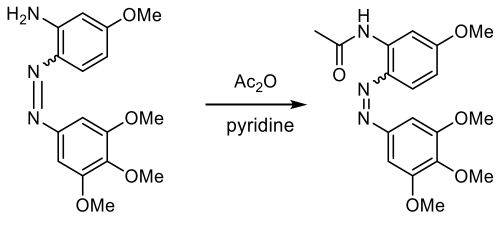

S29 **S29-Ac** 97%

N-(5-methoxy-2-((3,4,5-trimethoxyphenyl)diazenyl)phenyl)acetamide (**S29-Ac**)

To **S29** (51 mg, 0.16 mmol) were added pyridine (5 mL) and acetic anhydride (0.5 mL) and the mixture was stirred overnight. After evaporation of the volatiles at 2 mbar and 30 °C, the residue was partitioned between aqueous HCl (1 M, 5 mL) and EtOAc (5 mL), then the aqueous layer was extracted with EtOAc (2×10 mL); the combined organic layers were washed with aqueous HCl (1 M, 5 mL) and brine (5 mL), then dried on Na_2SO_4, filtered and concentrated to an olive powder which was spectroscopically pure **S29-Ac** (56 mg, 0.16 mmol, 97%; R_f = 0.15 on 2.4:1 Hx:EA (*trans* and *cis* isomers overlapped), $FeCl_3$).

^{1}H-NMR (400 MHz, $CDCl_3$): δ = 10.53 (s, 1H), 8.24 (d, 2.7 Hz, 1H), 7.76 (d, 9.0 Hz, 1H), 7.04 (s, 2H), 6.65 (dd, 9.0 & 2.8 Hz, 1H), 3.89 (s, 6H), 3.87 (s, 3H), 3.84 (s, 3H), 2.20 (s, 3H) ppm. ^{13}C-NMR (100 MHz, $CDCl_3$): δ = 168.8, 163.3, 153.7 (×2), 148.2, 140.4, 137.1, 133.1, 124.9, 110.8, 103.7, 99.7 (×2), 61.2, 56.2 (×2), 55.8, 25.6 ppm. HRMS (ESI-) calcd for $[C_{18}H_{20}N_3O_5]^-$ = $[M-H]^-$: m/z 358.14030, found 358.14059**.** LCMS(+): t_{ret} = 3.12 & 4.41 min, each 360 Th = $[MH]^+$: these peaks were assigned to the *cis* & *trans* isomers respectively since the UV absorption profile of the first peak (*cis*) featured a shoulder centred around 450 nm which was absent in the second peak.

S30 & PST-30

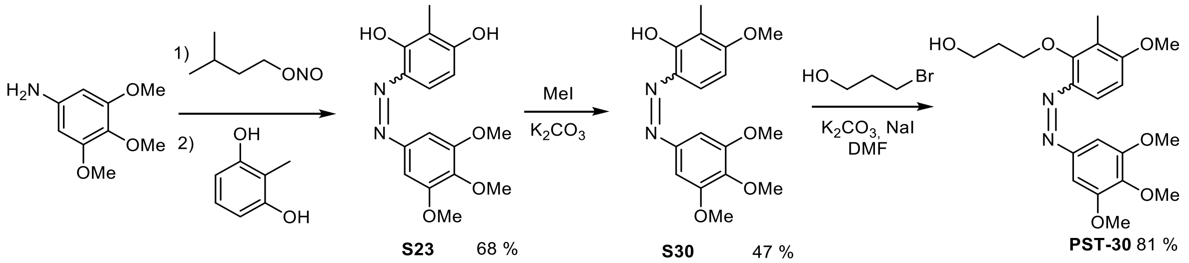

2-methyl-4-((3,4,5-trimethoxyphenyl)diazenyl)benzene-1,3-diol (**S23**)

By Standard Procedure A, 3,4,5-trimethoxyaniline (916 mg, 5.01 mmol) was reacted with commercial 2-methylresorcinol (618 mg, 4.98 mmol). Chromatography of the red crude oil on 5:1→1:1 Hx:EA returned **S23** (1079 mg, 3.39 mmol, 68%; R_f = 0.63 on 1:1 Hx:EA, $FeCl_3$) as a red solid. ^{1}H NMR (400 MHz, DMSO-d$_6$): δ = 12.98 (s, 1H), 10.48 (s br, 1H), 7.56 (d, *J* = 8.8 Hz, 1H), 7.26 (s, 2H), 6.60 (d, *J* = 8.9 Hz, 1H), 3.88 (s, 6H), 3.74 (s, 3H), 2.04 (s, 3H) ppm. ^{13}C NMR (100 MHz, DMSO-d$_6$): δ = 160.9, 154.3, 153.9 (×2), 146.7, 139.6, 132.0, 128.7, 111.2, 108.6, 99.7, 60.7, 56.5 (×2), 8.3 ppm. LCMS(+): t_{ret} = 4.26 min, 319 Th = $[MH]^+$. HRMS (ESI+) calcd for $[C_{16}H_{19}N_2O_5]^+$ = $[MH]^+$: m/z 319.12157, found 319.12859.

3-methoxy-2-methyl-6-((3,4,5-trimethoxyphenyl)diazenyl)phenol (**S30**)

By Standard Procedure B, **S23** (1.05 g, 3.30 mmol) was methylated for four hours with MeI (1.02 eq). Chromatography on silica, using a gradient of 5:1→1:1 Hx:EA returned **S30** (520 mg, 1.56 mmol, 47%; R_f = 0.51 on 2.4:1 Hx:EA, $FeCl_3$) as a red-orange solid crystallising as fine red needles from Hx/EtOAc. ^{1}H NMR (400 MHz, $CDCl_3$): δ = 13.65 (s, br, 1H), 7.72 (d, *J* = 8.9 Hz, 1H), 7.09 (s, 2H), 6.63 (d, *J* = 8.9 Hz, 1H), 3.94 (s, 6H), 3.92 (s, 3H), 3.91 (s, 3H), 2.14 (s, 3H) ppm. ^{13}C NMR (100 MHz, $CDCl_3$): δ = 161.39, 153.73, 152.94, 146.27, 139.89, 132.87, 131.96, 113.58, 102.79, 99.06, 61.08, 56.22, 55.84, 7.58 ppm. LCMS(+): t_{ret} = 5.24 min, 333 Th = $[MH]^+$. HRMS (ESI+) calcd for $[C_{17}H_{21}N_2O_5]^+$ = $[MH]^+$: m/z 333.14450, found 333.14421.

3-(3-methoxy-2-methyl-6-((3,4,5-trimethoxyphenyl)diazenyl)phenoxy)propan-1-ol (**PST-30**)

To **S30** (165 mg, 0.50 mmol) were added NaI (15 mg, 0.1 mmol), 3-bromopropanol (210 mg, 1.53 mmol), K_2CO_3 (207 mg, 1.46 mmol), and DMF (6 mL) and the mixture stirred overnight at room temperature. Water (10 mL), aqueous LiCl (10%, 10 mL) and aqueous KH_2PO_4 solution (10%, 10 mL) were added and the aqueous phase extracted with $CHCl_3$ (10 mL) then Et_2O (2 × 15 mL); the combined organic layers were washed with water (10 mL), aqueous LiCl (10%, 10 mL), and brine (10 mL), then dried on Na_2SO_4, filtered and concentrated. After column chromatography on 5:1→1:1 Hx:EA, **PST-30** (158 mg, 0.405 mmol, 81%; R_f = 0.35 and 0.12 on 1:1 Hx:EA (*trans* and *cis* isomers), Van) was returned as a yellow oil. ^{1}H NMR (400 MHz, MeOD): δ = 7.68 (d, *J* = 9.0 Hz, 1H), 7.29 (s, 2H), 6.72 (d, *J* = 9.1 Hz, 1H), 4.22 (t, *J* = 5.6 Hz, 2H), 3.98 (s, 6H), 3.97 – 3.94 (m, 2H), 3.93 (s, 3H), 3.91 (s, 3H), 2.24 (s, 3H), 2.10 – 2.05 (m, 2H) ppm. ^{13}C NMR (100 MHz, MeOD): δ = 161.62, 156.72, 153.71, 148.70, 140.40, 140.07, 120.72, 115.38, 106.53, 100.45, 75.11, 61.75, 61.20, 56.37, 56.06, 32.47, 9.10 ppm. HRMS (ESI+) calcd for $[C_{20}H_{27}N_2O_6]^+$ = $[MH]^+$: m/z 391.18636, found 391.18604. LCMS(+): t_{ret} = 3.52 & 4.48 min, each 391 Th = $[MH]^+$; the first peak was assigned as the *cis* isomer due to its secondary band centred at 450 nm.

S28

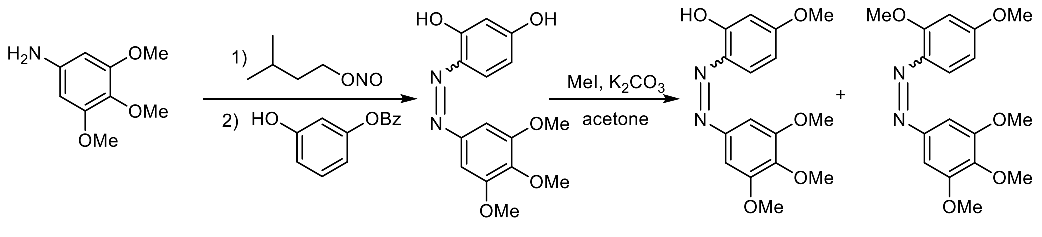

4-((3,4,5-trimethoxyphenyl)diazenyl)benzene–1,3-diol (**S24**):

By Standard Procedure A, 3,4,5-trimethoxyaniline (590 mg, 3.22 mmol) was reacted with resorcinol monobenzoate (724 mg, 3.38 mmol), where the phenol was dissolved in NaOH only one minute prior to diazonium addition to reduce ester hydrolysis prior to reaction, and where the coupling was run for only 15 minutes before neutralisation and extraction. Chromatography of the red crude oil on 5:1→1:1 Hx:EA returned the major product **S24** (693 mg, 2.28 mmol, 71%; R_f = 0.37 on 1.7:1 Hx:EA, $FeCl_3$) as a deep red powder. ^{1}H NMR (400 MHz, DMSO-d_6): δ = 12.15 (s, 1H), 10.50 (s, 1H), 7.68 (d, J = 8.8 Hz, 1H), 7.26 (s, 2H), 6.49 (dd, J = 8.8 Hz, 2.5 Hz, 1H), 6.36 (d, J = 2.5 Hz, 1H), 3.88 (s, 6H), 3.74 (s, 3H) ppm. ^{13}C NMR (100 MHz, DMSO-d_6): δ = 163.1, 156.6, 153.9 (×2), 147.2, 139.7, 132.5, 129.5, 109.4, 103.5, 99.9 (×2), 60.7, 56.5 (×2) ppm. HRMS (ESI+) calcd for $[C_{15}H_{17}N_2O_5]^+ = [MH]^+$: m/z 305.11320, found 305.11322.

5-methoxy-2-((3,4,5-trimethoxyphenyl)diazenyl)phenol (**S28**)

By Standard Procedure B, **S24** (670 mg, 2.20 mmol) was methylated overnight with MeI (618 mg, 4.35 mmol, 1.98 eq). Chromatography on a gentle gradient of 10:1→1:1 Hx:EA returned target *para*-monomethylated **S28** (255 mg, 0.80 mmol, 36%; R_f = 0.52 on 2.4:1 Hx:EA, $FeCl_3$) as a red solid, followed by undesired bismethylated byproduct **PST-7** (100 mg, 0.30 mmol, 14%; R_f = 0.19 on 2.4:1 Hx:EA, $FeCl_3$) as a viscous red oil.

S28: ^{1}H NMR (400 MHz, $CDCl_3$): δ = 13.69 (s, 1H), 7.77 (d, J = 8.9 Hz, 1H), 7.09 (s, 2H), 6.61 (dd, J = 8.9, 2.7 Hz, 1H), 6.48 (d, J = 2.7 Hz, 1H), 3.95 (s, 6H), 3.92 (s, 3H), 3.87 (s, 3H) ppm. ^{13}C NMR (100 MHz, $CDCl_3$): δ = 163.92, 156.29, 153.87, 146.13, 140.07, 134.60, 132.81, 108.37, 101.51, 99.13, 61.19, 56.37, 55.83 ppm. LCMS(+): t_{ret} = 4.83 min, 319 Th = $[MH]^+$: this peak was assigned to the *trans* isomer since the UV absorption profile did not feature any secondary band. HRMS (ESI-) calcd for $[C_{16}H_{17}N_2O_5]^- = [M-H]^-$: m/z 317.11430, found 317.11410.

MR69

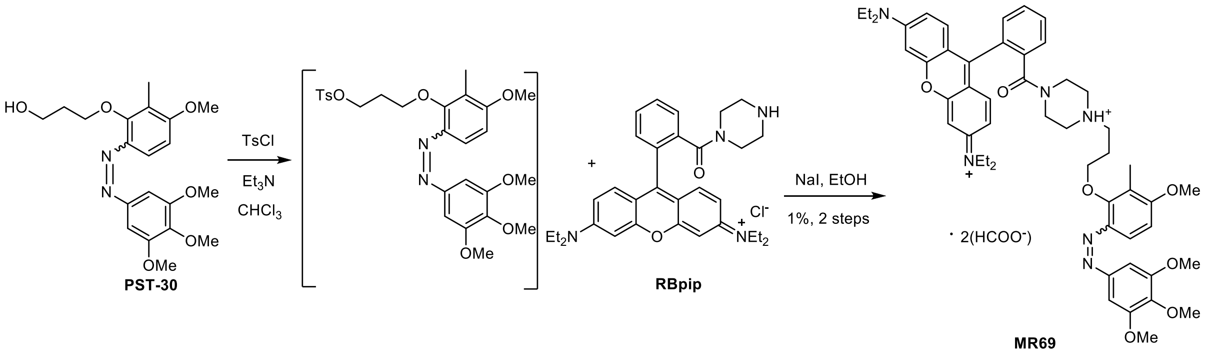

N-(6-(diethylamino)-9-(2-(4-(3-(3-methoxy-2-methyl-6-((3,4,5-trimethoxyphenyl)diazenyl)phenoxy)propyl)piperazine-1-carbonyl)phenyl)-3H-xanthen-3-ylidene)-*N*-ethylethanaminium bis(formate) salt (**MR69**)

Known compound *N*-(6-(diethylamino)-9-(2-(piperazine-1-carbonyl)phenyl)-3H-xanthen-3-ylidene)-*N*-ethylethanaminium chloride (abbreviated **RBpip** chloride) was made by the reported method and confirmed by NMR.[52]

To **PST-30** (140 mg, 0.36 mmol) were added CH_2Cl_2 (5 mL), Et_3N (89 mg, 0.88 mmol), 4-dimethylaminopyridine (10 mg), and TsCl (69 mg, 0.36 mmol), and the mixture stirred at room temperature for 4 hours until TLC indicated complete conversion of the starting material, presumably to the tosylate. After evaporation of the volatiles, to the residue were added EtOH (15 mL), NaI (20 mg), **RBpip** chloride (160 mg, 0.29 mmol) and NEt_3 (81 mg), and the mixture stirred at 80 °C for 2 days under closed air atmosphere. After cooling and evaporation of the volatiles, the crude residue was chromatographed on a small volume of silica gel to separate the bulk of the impurities, using a gradient of 1:1:0:0→1:1:1:0→1:1:0:0→1:1:0:1→0:0:0:1→0:0:1:1 Hx:EA:MeOH:CH_2Cl_2. The third red fraction to elute contained the pink-fluorescent product cation of **MR69** ($R_f = 0.25 < R_f < 0.4$ on 9:1 DCM:MeOH) as well as substantial impurities. This fraction was then concentrated (dry weight: 45 mg) then separated by semi-preparative HPLC on reverse-phase column with a 10:90 → 60:40 MeCN:water eluent gradient (water component contains 0.1% formic acid), and pure **MR69** as the bis(formate) salt (2.0 mg, 2.0 µmol, 1%) was recovered from the pure fractions as a dark purple solid. ^1H NMR (400 MHz, CD_3CN) showed overlapping peaks from two components at 5:2 ratio (the ^{13}C NMR spectrum suggests these may be conformers about the piperazine): δ = 8.35 (s, 2H), 7.72 – 7.68 (m, 2H), 7.59 – 7.54 (m, 2H), 7.43 – 7.40 (m, 1H), 7.20 (s, 2H), 7.17 (s, 1H), 6.94 (dd, *J* = 9.5, 2.5 Hz, 2H), 6.84 – 6.77 (m, 4H), 4.13 (t, *J* = 6.3 Hz, 2H), 3.88 (s, 3H), 3.88 (s, 6H), 3.79 (s, 3H), 3.59 (q, *J* = 7.2 Hz, 8H), 3.30 – 3.20 (m, 4H), 2.48 – 2.33 (m, 2H), 2.17 (s, 3H), 2.11 –2.07 (m, 4H), 1.91 – 1.88 (m, 2H), 1.23 (t, *J* = 7.0 Hz, 12H) ppm. ^{13}C NMR (100 MHz, CD_3CN): δ = 167.24, 164.98, 161.81, 158.29, 157.53, 156.70, 156.20, 154.30, 149.59, 140.67, 140.16, 136.56, 132.73, 131.21, 130.53, 130.37, 129.99, 127.97, 120.54, 114.82, 114.55, 114.17, 106.76, 100.56, 96.42, 74.63, 60.64, 56.37, 56.27, 55.15, 53.32, 47.73, 46.22, 27.90, 12.37, 8.86 ppm. LCMS(+): t_{ret} = 3.25 (*Z*) & 3.45 (*E*) min, each 883 Th = $[MH]^+$, assigned since the second peak has greater absorbance at 390 nm. HRMS (ESI+) calcd for $[C_{52}H_{63}N_6O_7]^+ = [M]^+$: m/z 883.47527, found 883.47453.

MR110

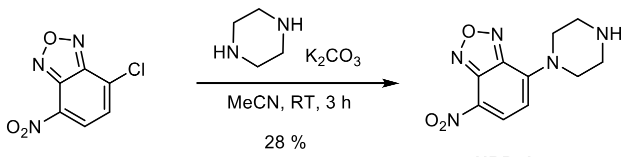

4-nitro-7-(piperazin-1-yl)benzo[c][1,2,5]oxadiazole (**NBDpip**)

To NBD-Cl (100 mg, 0.5 mmol) in MeCN (5 mL) was added piperazine (43 mg, 0.5 mmol) and potassium carbonate (70 mg, 0.5 mmol) and stirred for 3 h during which the reaction mixture turned red. The crude was concentrated and purified on silica gel starting the elution with 1:1 Hx:EA, then changing to 4:1 CH_2Cl_2:MeOH. **NBDpip** was obtained as orange solid (69 mg, 0.14 mmol, 28%; R_f = 0.62 on 4:1 CH_2Cl_2:MeOH).

^{1}H NMR (400 MHz, DMSO-d_6) δ = 8.45 (d, J = 9.2 Hz, 1H), 6.64 (d, J = 9.3 Hz, 1H), 4.08 (t, J = 4.8 Hz, 4H), 2.98 – 2.87 (m, 4H) ppm. ^{13}C NMR (100 MHz, DMSO-d$_6$) δ = 145.8, 145.4, 145.3, 136.8, 120.8, 103.6, 51.8, 46.3 ppm. LCMS(+): t_{ret} = 1.64 min, 250 Th = [MH]$^+$. HRMS (ESI+) calcd for $[C_{10}H_{12}N_5O_3^+]^+$ = [MH]$^+$: m/z 250.09347, found 250.09327.

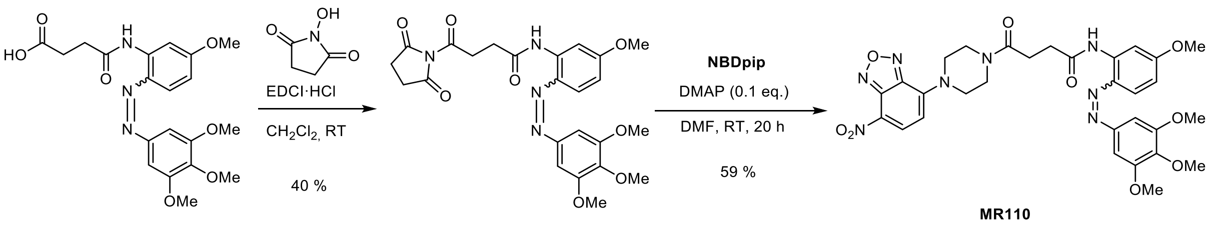

4-(2,5-dioxopyrrolidin-1-yl)-N-(5-methoxy-2-((3,4,5-trimethoxyphenyl)diazenyl)phenyl)-4-oxobutanamide (**S25**)

To **PST-29** (360 mg, 0.86 mmol) were added *N*-hydroxysuccinimide (110 mg, 0.95 mmol), EDCI·HCl (182 mg, 0.95 mmol) and CH_2Cl_2 (10 mL) stirring the mixture at room temperature for 12 h. The solvent was evaporated and the crude product was chromatographed on silica gel using a gradient of 1:1 → 1:5 Hx:EA. **S25** was obtained as orange solid (175 mg, 0.34 mmol, 40%; R_f = 0.40 on 1:5 Hx:EA). The product slowly decomposes upon standing or on silica and was directly used for the next step.

LCMS(+): t_{ret} = 3.45 & 4.32 min, each 515 Th = [MH]$^+$. ^{1}H NMR (400 MHz, $CDCl_3$) δ = 10.70 (s, 1H), 8.23 (d, J = 2.8 Hz, 1H), 7.75 (d, J = 9.0 Hz, 1H), 7.06 (s, 2H), 6.66 (dd, J = 9.0 Hz, 2.7 Hz, 1H), 3.89 (s, 6H), 3.87 (s, 3H), 3.84 (s, 3H), 3.05 (t, J = 7.0 Hz, 2H), 2.82 (t, J = 7.0 Hz, 2H), 2.72 (s, 4H) ppm. ^{13}C NMR (100 MHz, $CDCl_3$) δ = 168.8, 168.6, 168.3, 163.2, 153.7, 148.3, 140.3, 136.7, 133.3, 124.8, 111.2, 103.7, 99.8, 61.1, 56.3, 55.8, 32.4, 26.5, 25.5 ppm.

N-(5-methoxy-2-((3,4,5-trimethoxyphenyl)diazenyl)phenyl)-4-(4-(7-nitrobenzo[c][1,2,5]oxadiazol-4-yl)piperazin-1-yl)-4-oxobutanamide (**MR110**)

To **S25** (20 mg, 0.04 mmol) were added **NBDpip** (20 mg, 0.08 mmol, 2 eq.), DMAP (0.2 mg, 0.002 mmol) and DMF (1 mL) and the solution was stirred overnight. The reaction was partitioned between water (20 mL) and extracted with EtOAc (3 × 20 mL), then the combined organic layers were washed with aqueous NaOH (2 M, 20 mL), aqueous HCl (1 M, 20 mL) and

aqueous NaOH (2 M, 20 mL) again, then dried on Na_2SO_4, filtered and concentrated to an crude orange powder which was chromatographed on silica gel using a gradient of 1:1:0 → 1:1:0.1 Hx:EA:MeOH. **MR110** was obtained as orange solid (15 mg, 0.023 mmol, 59%; R_f = 0.21 on 1:1:0.1 Hx:EA:MeOH).

HRMS (ESI-) calcd for $[C_{30}H_{31}N_8O_9^-]^-$ = $[M-H]^-$: m/z 647.22195, found 647.22178**.**

LCMS(+): t_{ret} = 3.94 & 4.83 min, each 649 Th = $[MH]^+$. ^{1}H NMR (400 MHz, $CDCl_3$) δ = 10.66 (s, 1H), 8.38 (d, *J* = 8.8 Hz, 1H), 8.27 (d, *J* = 2.7 Hz, 1H), 7.78 (d, *J* = 9.0 Hz, 1H), 7.23 (s, 2H), 6.68 (dd, *J* = 9.0 Hz, 2.7 Hz, 1H), 6.23 (d, *J* = 8.8 Hz, 1H), 4.25 (t, *J* = 5.3 Hz, 2H), 3.98 (s, 6H), 3.97 – 3.94 (m, 2H), 3.94 (s, 3H), 3.92 – 3.88 (m, 4H), 3.87 (s, 3H), 2.91 – 2.81 (m, 4H) ppm. ^{13}C NMR (100 MHz, $CDCl_3$) δ = 171.04, 170.66, 163.28, 153.72, 148.57, 144.83, 144.67, 140.43, 137.59, 134.99, 133.64, 124.46, 122.96, 110.72, 103.97, 102.74, 100.27, 100.21, 61.23, 56.45, 55.83, 48.68, 48.61, 44.47, 40.77, 33.22, 28.59 ppm.

PST-31

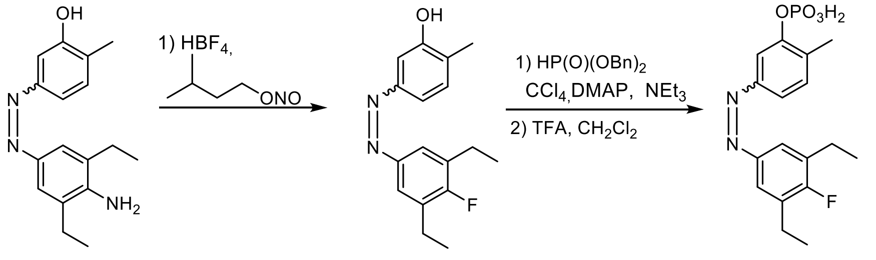

S19 **S31** 17% **PST-31** 43%

5-((3,5-diethyl-4-fluorophenyl)diazenyl)-2-methylphenol (**S31**)

To the aniline **S19** (296 mg, 1.05 mmol, 1.0 eq) were added acetonitrile (6 mL) and HBF_4 (4 mL) and the mixture was cooled to 0 °C with an ice bath. Isopentyl nitrite (161 mg, 1.38 mmol, 1.3 eq) was added dropwise and the reaction mixture was first stirred at 0 °C for 10 min and was then heated to 60 °C. After 30 min, the pH value was adjusted to neutral by adding NaOH (2 M) and $NaHCO_3$/Na_2CO_3-buffer. The aqueous mixture extracted with EA (2x20 mL) and the combined organic layers were washed with water (2x10 mL), brine (2x10 mL), dried over Na_2SO_4, filtered and concentrated to give the crude product. Column chromatography (silica gel, 25:1 → 10:1, Hx:EA) gave the product as a solid (50 mg, 0.17 mmol, 17%, R_f = 0.70 on 2.5:1, Hx:EA).

^{1}H-NMR (400 MHz, $CDCl_3$): δ = 7.53 (s, 1H), 7.51 (s, 1H), 7.37 (dd, *J* = 7.7, 1.9 Hz, 1H), 7.20 (d, *J* = 1.9 Hz, 1H), 7.16 (d, *J* = 7.6 Hz, 1H), 5.47 (s, OH), 2.64 (q, *J* = 7.7 Hz, 4H), 2.21 (s, 3H), 1.18 (t, *J* = 7.6 Hz, 6H) ppm. ^{13}C-NMR (101 MHz, $CDCl_3$): δ = 160.96 (d, *J* = 250.5 Hz, C-F), 154.35 (1xC_{quat}), 152.12 (1xC_{quat}), 148.55 (1xC_{quat}), 131.49 (2xC_{quat}), 131.34 (1xCH), 127.90 (1xC_{quat}), 121.95 (2xCH), 117.65 (1xCH), 106.75 (1xCH), 22.34 (2xCH_2), 16.10 (1xCH_3), 14.21 (2xCH_3) ppm. LCMS(70/100, +): t_{ret} = 3.51 & 6.62 min, 287 Th = $[MH]^+$. HRMS (EI): m/z: calc. for $[C_{17}H_{20}FN_2O]^+$: 287.15542, found: 287.15535.

dibenzyl (5-((3,5-diethyl-4-fluorophenyl)diazenyl)-2-methylphenyl) phosphate

To a cold mixture of the phenol (50 mg, 0.17 mmol, 1.0 eq), NEt_3 (136 mg, 1.18 mmol, 6.9 eq), CCl_4 (160 mg, 1.04 mmol, 6.1 eq) in acetonitrile (6 mL), was added dropwise a solution of $HPO(OBn)_2$ (76 mg, 0.28 mmol, 1.6 eq) in acetonitrile. The resulting mixture was stirred at rt until completion of phosphorylation (typically 1-2 h). The volatiles were evaporated to give the crude phenolic dibenzyl ester. Column chromatography (silica gel, 25:1 → 5:1, Hx:EA) yielded the product (57 mg, 0.10 mmol, 61%, R_f = 0.53 on 2.5:1, Hx:EA) which was directly used in the next step.

^{1}H-NMR (400 MHz, $CDCl_3$): δ = 7.88 (s, 1H), 7.71 (d, *J* = 8.0 Hz, 1H), 7.68 (s, 1H), 7.66 (s, 1H), 7.35 (m, 11H), 5.22 (s, 2H), 5.19 (s, 2H), 2.78 (q, *J* = 7.6 Hz, 4H), 2.34 (s, 3H), 1.34 (t, *J* = 7.6 Hz, 6H ppm. ^{13}C-NMR (101 MHz, $CDCl_3$): δ = 161.08 (d, *J* = 250.8 Hz, C-F), 151.85 (1xC_{quat}), 149.49 (1xC_{quat}), 148.50 (1xC_{quat}), 135.43–128.05 (16xC_{Aryl}), 122.16 (2xCH), 120.68 (1xCH), 113.14 (1xCH), 70.13 (2xCH_2), 22.35 (2xCH_2), 16.59 (1xCH_3), 14.27 (2xCH_3) ppm. LCMS(50/100, +): t_{ret} = 9.35 min, 547 Th = $[MH]^+$. HRMS (EI): m/z: calc. for $[C_{31}H_{32}FN_2O_4P]$: 546.2084; found: 546.2086.

5-((3,5-diethyl-4-fluorophenyl)diazenyl)-2-methylphenyl dihydrogen phosphate (**PST-31**)

The dibenzyl ester (87 mg, 0.16 mmol) was stirred in 1:1 TFA/DCM for 16 h. Then, the volatiles were removed under reduced pressure. To the crude residue was added EA (20 mL) and the resulting mixture was then quenched with phosphate buffer and adjusted to pH 7. The aqueous layer was first rinsed with pure Hx (10 mL) and then with a 1:1 Hx/EA mixture (10 mL). The pH of the aqueous layer was adjusted to 2 with 2 M HCl and the reaction mixture was extracted with EA (2x10 mL) and concentrated to yield the pure product **PST-31** (41 mg, 0.11 mmol, 70%).

^{1}H-NMR (400 MHz, MeOD): δ = 7.68 (t, *J* = 1.6 Hz, 1H), 7.49 – 7.40 (m, 3H), 7.19 (d, *J* = 8.1 Hz, 1H), 2.59 – 2.50 (m, 4H), 2.20 (s, 3H), 1.08 (t, *J* = 7.6 Hz, 6H) ppm. ^{13}C-NMR (101 MHz, MeOD): δ = 162.17 (d, *J* = 249.7 Hz), 153.06, 151.91 (d, *J* = 6.9 Hz), 149.96 (d, *J* = 3.4 Hz), 134.41 (d, *J* = 6.2 Hz), 132.88, 132.69, 132.42, 123.11 (d, *J* = 6.1 Hz), 121.07, 114.13 (d, *J* = 2.5 Hz), 23.22 (d, *J* = 3.4 Hz), 16.77, 14.73 (d, *J* = 1.3 Hz) ppm. LCMS(10/100, -): t_{ret} = 5.58 & 6.40 min, 365 Th = $[M]^-$; HRMS (EI+): m/z: calc. for $[C_{17}H_{21}FN_2O_4P]^+$: 367.12175 found: 367.12174.

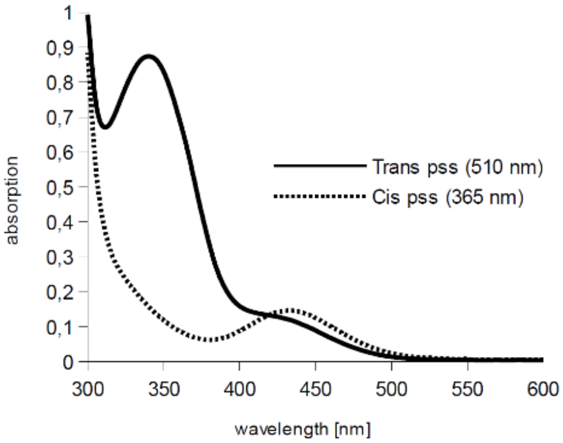

*UV-Vis absorption spectra of **PST-31** in PBS buffer (pH 7) at 365 nm and 561 nm PSSs.*

PST-32

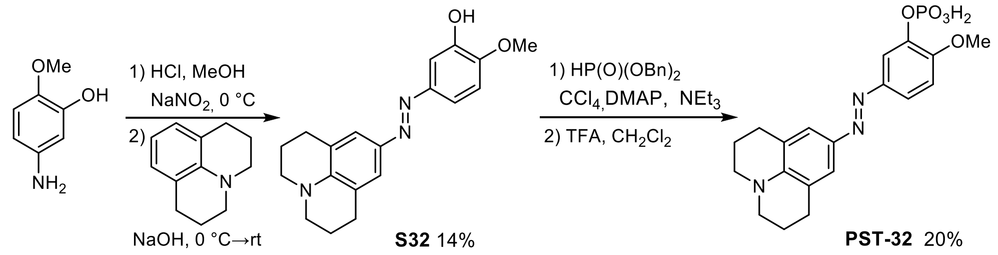

2-methoxy-5-((2,3,6,7-tetrahydro-1H,5H-pyrido[3,2,1-ij]quinolin-9-yl)diazenyl)phenol (**S32**)

To the aminophenol (163 mg, 1.06 mmol, 1.0 eq) were added 2.0 M aqueous H_2SO_4 (>3.0 eq) and a small amount of MeOH until dissolution, while cooling the mixture in an ice bath (under air). Then an aqueous solution of $NaNO_2$ (2.3 M, 0.55 mL, 1.27 mmol, 1.2 eq). was added dropwise, and the mixture was left stirring for 5 - 20 min in the cold. Sulfamic acid (>0.3 eq) was then added and the reaction stirred for 3 min to consume the excess of HNO_2. Julolidine (190 mg, 1.10 mmol, 1.1 eq), dissolved in MeOH, was then added to the cold diazonium solution, giving the crude product. Solid NaOAc (>10.0 eq) was then added, diluting with cold water and basifying where necessary (final pH typically about 5-6). The reaction was stirred until complete consumption. Methanol was removed under reduced pressure and the crude aqueous mixture was extracted with ethylacetate (EA) (2x20 mL). The combined organic layers were then washed with water (2x10 mL), brine (2x10 mL), dried over Na_2SO_4 and filtered. The crude was concentrated and purified by column chromatography (silica gel, 25:1 → 5:1, Hx:EA) to obtain the red product (47 mg, 0.15 mmol, 14%, R_f = 0.29 on 2.5:1, Hx:EA).

^{1}H-NMR (400 MHz, $CDCl_3$): δ = 7.48 (d, *J* = 2.3 Hz, 1H), 7.46 (s, 3H), 6.95 (d, *J* = 8.6 Hz, 1H), 5.70 (s, OH), 3.97 (s, 3H), 3.29 (t, *J* = 5.8 Hz, 4H), 2.84 (t, *J* = 6.4 Hz, 4H), 2.01 (m, 4H). ^{13}C-NMR (101 MHz, $CDCl_3$): δ = 148.17 (1xC_{quat}), 147.75 (1xC_{quat}), 146.03 (1xC_{quat}), 145.24 (1xC_{quat}), 142.56 (1xC_{quat}), 122.39 (2xC_{quat}), 120.89 (2xCH), 117.19 (1xCH), 110.14 (1xCH), 105.93 (1xCH), 56.09 (1xCH_3), 50.04 (2xCH_2), 27.80 (2xCH_2), 21.68 (2xCH_2). LCMS(+): t_{ret} = 3.89 min, 324 Th = $[MH]^+$ HRMS (EI+): m/z: calc. for $[C_{19}H_{22}N_3O_2]^+$: 324.17065, found: 324.17057.

Dibenzyl(2-methoxy-5-((2,3,6,7-tetrahydro-1H,5H-pyrido[3,2,1-ij]quinolin-9-yl)diazenyl)phenyl) phosphate

To a cold mixture of the corresponding phenol (37 mg, 0.11 mmol, 1.0 eq), NEt_3 (77 mg, 0.67 mmol, 6.1 eq), (DMAP one crystal), CCl_4 (2 mL) and acetonitrile (5 mL), was added dropwise a solution of $HPO(OBn)_2$ (51 mg, 0.19 mmol, 1.7 eq) in acetonitrile. The resulting mixture was stirred at rt until completion of phosphorylation (typically 1-2 h). The volatiles were evaporated to give the crude phenolic dibenzyl ester. Column chromatography (silica gel, 15:1 → 1:1, Hx:EA) yielded the dibenzyl phosphate (38 mg, 0.07 mmol, 60%, R_f = 0.45 on 1:1, Hx:EA) which was directly used in the next step.

LCMS(+): t_{ret} = 5.79 min, 584 Th = $[C_{33}H_{35}N_3O_5P]^+$

2-methoxy-5-((2,3,6,7-tetrahydro-1H,5H-pyrido[3,2,1-ij]quinolin-9-yl)diazenyl)phenyl dihydrogen phosphate (**PST-32**)

For the deprotection, the dibenzyl ester was stirred in 1:1 TFA/DCM o.n., under nitrogen atmophere. The reaction was followed by LCMS. Once complete, the volatiles were removed

under reduced pressure. To the crude residue was added EA (20 mL) and the resulting mixture was then quenched with phosphate buffer to get pH 7. The aqueous layer was first rinsed with pure Hx (10 mL) and then with a 1:1 Hx/EA mixture (10 mL). The pH of the aqueous layer was adjusted to 2 with 2 M HCl and the reaction mixture was extracted with EA (2x10 mL) and concentrated to yield the crude product. Reverse phase column chromatography (non-polar, functionalized silica gel, 10% → 100%, MeCN in H_2O) gave **PST-32** as a bright red solid (9.5 mg, 0.02 mmol, 34%).

^{1}H-NMR (400 MHz, D_2O): δ = 7.69 (s, 1H), 7.33 (d, *J* = 8.9 Hz, 1H), 7.26 (s, 2H), 7.10 (d, *J* = 8.7 Hz, 1H), 3.89 (s, 3H), 3.18 (t, *J* = 5.7 Hz, 4H), 2.72 (t, *J* = 6.4 Hz, 4H), 1.88 (p, *J* = 6.2 Hz, 4H) ppm. ^{13}C-NMR (101 MHz, D_2O): δ = 151.58, 146.39, 146.10, 143.61, 141.46, 122.38, 121.92, 118.08, 112.28, 111.55, 55.96, 49.63, 26.94, 20.77. LCMS(+): t_{ret} = 4.45 min, 404 Th = $[MH]^+$ HRMS (EI-): m/z: calc. for $[C_{19}H_{21}N_3O_5P]^-$: 402.12243, found: 402.12247.

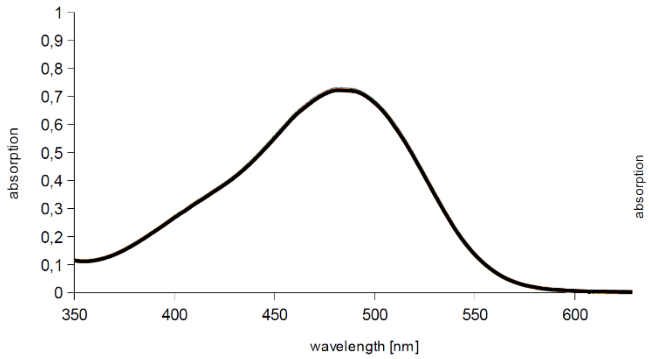

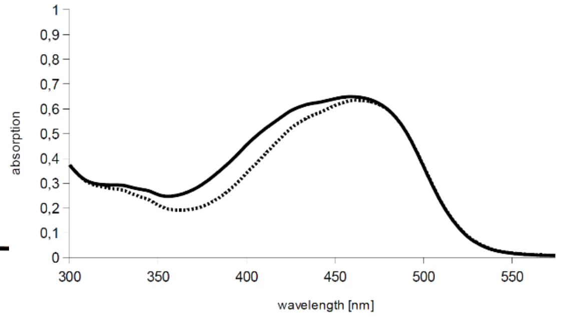

Absorption spectra of (left) **PST-32** in PBS buffer (pH 7); (right) its dephosphorylated free phenol in DMSO, in the dark (solid line) or under 475 nm illumination (dotted line).

Part B: Photocharacterisation in vitro

The rationale for the choice of isomerisation parameters to determine, and the methods for their measurement / modelling, were described in previous work[26].

Spectrophotometry equipment

Spectrophotometry was performed as described in previous work[26]. Separated spectra of *trans* and *cis* forms were acquired from the inline Diode Array Detector on the AGILENT 1260 SL coupled LC-MS system after HPLC separation as described in Part A with optional pre-illumination using a UV LED to generate a mix of *cis* and *trans*. Absorption spectra in cuvette ("UV-Vis") were acquired on a Varian CaryScan 50 (1 cm, 100 µL or 1 mL volume) with Peltier cell temperature control unit maintained at 37 °C, in PBS at pH~7.4 containing 10% MeCN by default. A TILL Photonics Polychrome V monochromator with a fibre optic cable output directed into the cuvette was used to deliver comparable intensity illumination at different wavelengths for comparing photoisomerisation speeds by UV-Vis spectrophotometry in the studies of fluorescent conjugates. While such monochromators are often used as a monochromatic source for measuring PSS, we caution that (a) the first harmonics of longer wavelengths (up to approx. 15% intensity at 345 nm relative to a selected band at 690 nm) are passed through the system by default; in our setup the fibre optic cut out all emission below ca. 320 nm (see **Fig S4**), i.e. below 620 nm there is no first harmonic light; but above it, the harmonic is strong and should be taken into account. (b) monochromators should be checked to confirm alignment so that broad spectrum stray light is not exiting the system (can be e.g. up to 1% intensity relative to a selected band if misaligned) - we checked ours with a spectrometer and confirmed that no detectable white stray light was passed through the fibre.

As a backup, also, single LEDs with lower monochromaticity but without potential broadband/UV contamination were used as light sources instead when appropriate (especially for fluorescent conjugates); in our opinion, their far greater delivered intensity and drastically lower cost recommend them as more appropriate, affordable, and versatile light sources than monochromators. LEDs CUN6AF1B (365 nm), CUN8AF1B (385 nm), CUN9AF1B (395 nm), and CUN0AF1B (405 nm) (all driven at 500 mA); H2A1-H420 (420 nm), H2A1-H435 (435 nm), H2A1-H450 (450 nm), H2A1-H470 (470 nm), H2A1-H480 (480 nm), H2A1-H490 (490 nm), H2A1-H505 (505 nm), H2A1-H515 (515 nm), H11A1-HR-30 (625 nm), H2A1-H650 (650 nm), H2A1-H660 (660 nm), H2A1-H670 (670 nm), H2A1-H680 (680 nm), H2A1-H690 (690 nm), and H2A1-H720 (720 nm) (all driven at 350 mA); H2A3-H530 (530 nm) and H2A3-H590 (590 nm) (both driven at 700 mA) were used together with constant current drivers at the appropriate current ratings, eg. drivers EAN 4016138919431 (350 mA), EAN 4040849306013 (500 mA), and EAN 4040849306020 (700 mA) supplied by Conrad Electronic. Lower-power LEDs in the green-yellow spectral range, eg. LED545-04 (545 nm), LED565-03U (565 nm), B5-433-20 (572 nm), and LED610-03 (610 nm) (all driven at 20 mA) were used to complement the set of higher-power models thus covering the full spectrum from 365-720 nm with LEDs.

1: trans- and cis- absorption spectra, and PSS φ(λ)

As outlined in previous work[26], the absorption spectra of **PSTs** were monitored by UV-Vis in cuvette (**Fig S2**) under illuminations to isomerise between *trans* and *cis* isomers, to determine parameters λ_{max} (maximum absorption wavelength of the *trans* form), λ_{strong} (wavelength giving the PSS containing the most *cis* isomer, slightly but significantly different from λ_{max}) and λ_{iso} (the isosbestic point). The *trans* and *cis* isomers of each compound were separated by HPLC and absorption spectra $\varepsilon^*_E(\lambda)$ and $\varepsilon^*_Z(\lambda)$ (in different arbitrary units) were measured on the

inline ultraviolet/visible Diode Array Detector; these were then scaled via either an isosbestic region (typically 210-240 nm) or else an isosbestic point to derive absorption spectra $A_E(\lambda)$ and $A_Z(\lambda)$ in comparable arbitrary units. Indicative PSSs φ(λ) calculated as per previous work[26] under the approximation that the quantum yields of isomerisation are similar for both isomers across all wavelengths, are presented in **Fig S1** for representative compounds of the set **PST-6-32**. We estimate this procedure typically delivers a discrepancy of no more than 15% from measured PSSs in pH-neutral aqueous media, which we consider accurate enough for meaningful comparisons (deviations are larger for pH-sensitive compounds).

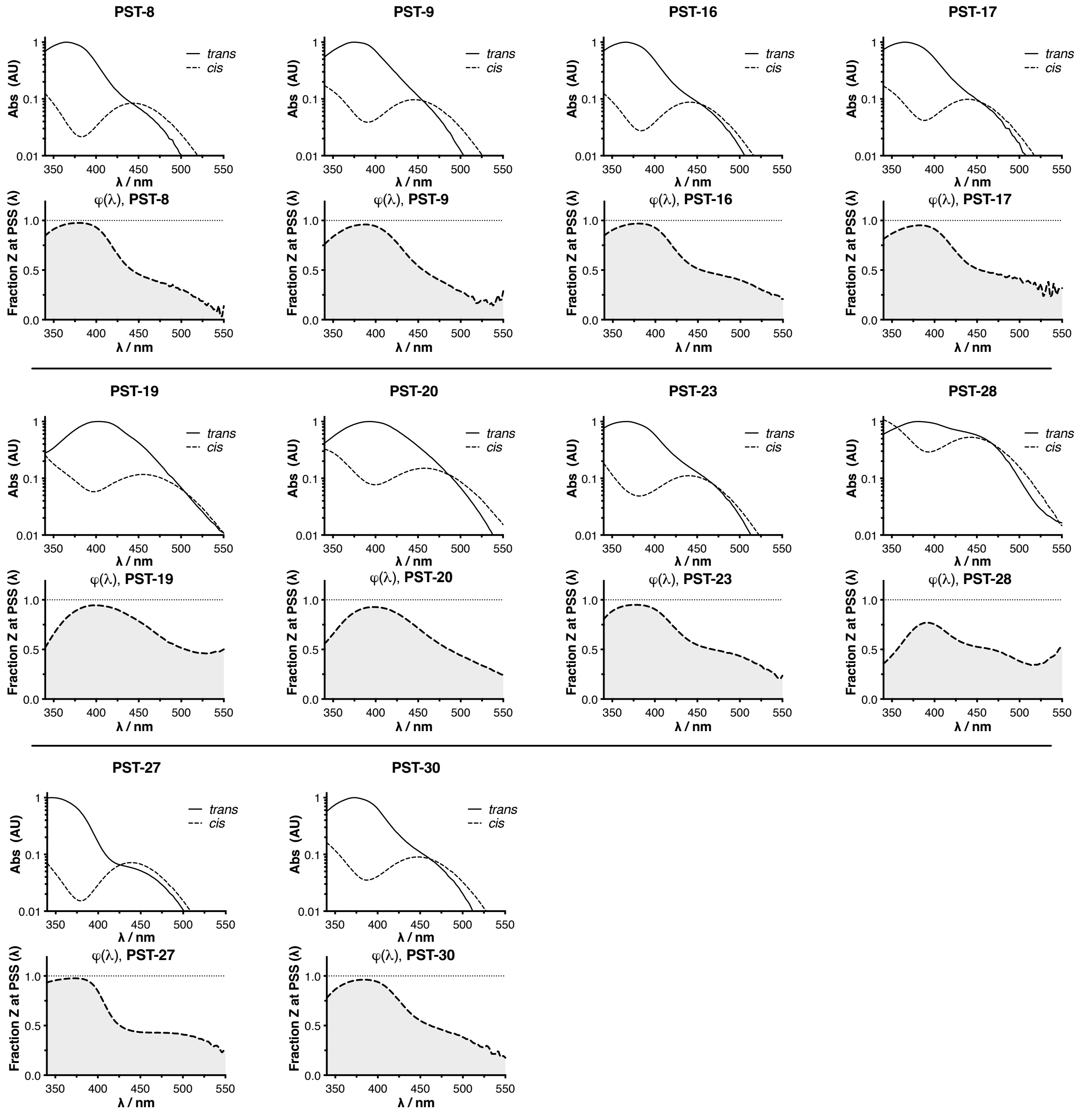

Figure S1 - Absorption spectra $A_E(\lambda)$ and $A_Z(\lambda)$ for *trans* and *cis* isomers in HPLC eluent, scaled onto the same arbitrary units scale, and fraction *Z* at PSS estimated from these spectra [φ(λ)], for selected **PSTs** (methods as detailed above).

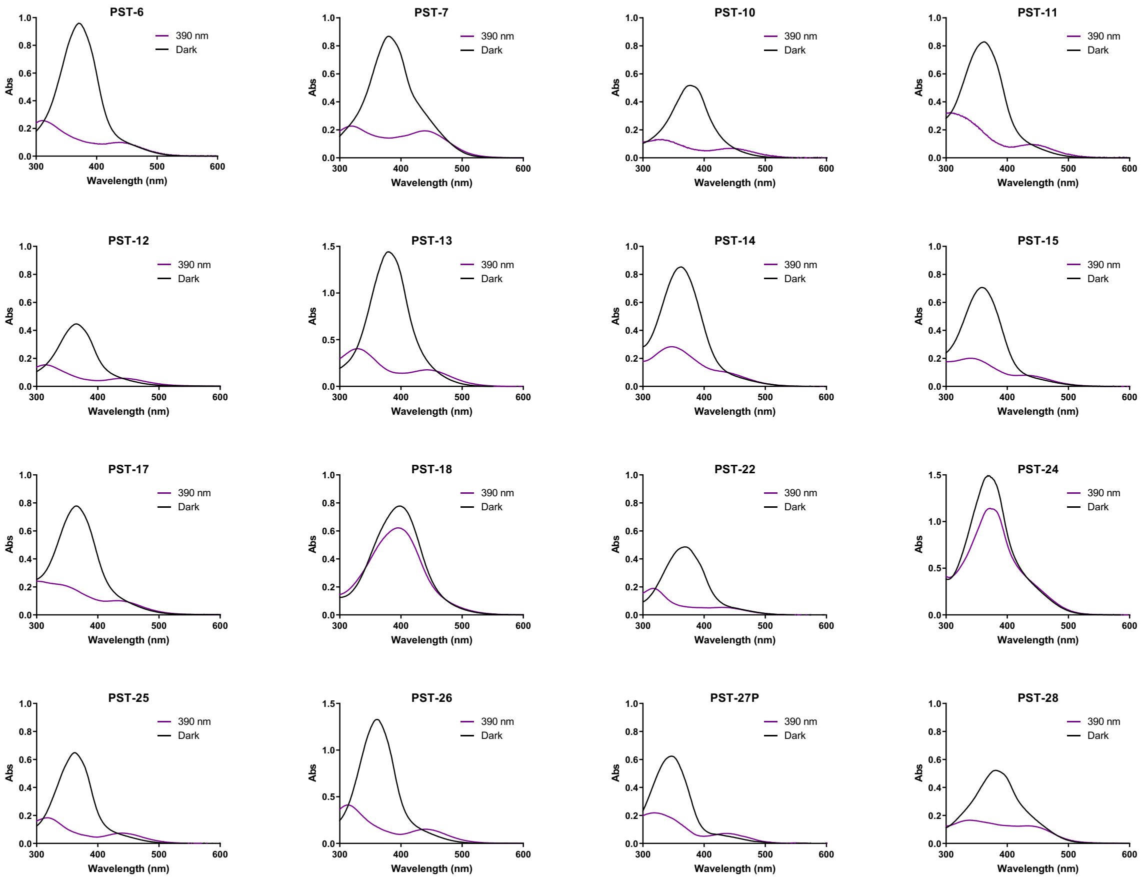

Figure S2 - Absorption spectra of selected PSTs in the dark or after irradiation with 390 nm (until PSS was reached) in PBS containing 50% MeCN.

2: Thermal cis→trans reversion halflives

Thermal relaxation halflives τ were measured as per previous work[26], in PBS at pH = 7.4 containing 50% MeCN to ensure solubility at room temperature unless indicated otherwise (**Table S1**). These halflives show that structural variations within the scope of photoswitchably bioactive **PST** designs can greatly alter the timescale of spontaneous *cis→trans* reversion.

Table S1. Key photoswitching properties of **PST** compounds, continued from Fig **1d**. Peak absorbance wavelength λ_{max} (*trans* form), isosbestic point λ_{iso} (only isosbestic points above 400 nm are reported), halflife for thermal relaxation $t_{1/2}$. *(10% MeCN in PBS was used and thermal relaxation was measured at 37 °C.) #(Measured in PBS)

	λ_{max} [nm]	$\lambda_{isosbest}$ [nm]	$t_{1/2}$ [min]
**PST-6**	371	458	> 48 h
**PST-7**	380	480	481
**PST-10**	377	451	344
**PST-11**	362	439	643
**PST-12**	365	440	505
**PST-13**	380	458	576
**PST-14**	362	438	> 48 h
**PST-15**	359	433	> 48 h
**PST-17**	364	450	1672
**PST-18**	398	474	-
**PST-22**	369	-	> 48 h
**PST-24**	370	432	-
**PST-25**	363	432	> 48 h
**PST-26**	361	434	> 48 h
**PST-27P**	346	421	435
**PST-28**	381	480	105*
**PST-29**	385	470	58
**PST-30**	372*	464*	177*
**PST-31#**	340	418	n.d.
**PST-32#**	484	-	n.d.

3: Reversible trans↔cis photoisomerisation of non-conjugated PSTs

A(λ_{strong}) was measured over time, while the monochromator applied λ_{irrad} alternating between two values λ_1 and λ_2, chosen to induce bulk *trans→cis* and bulk *cis→trans* isomerisation respectively. Typical results are presented for **PST-10** at 25 µM (50% MeCN in PBS) and **PST-27P** at 25 µM (in PBS) (**Fig S3**) measuring at λ_{strong}=375 nm, while the irradiating wavelength was held alternately at λ_1=380 nm (120 s; bulk *trans→cis*) then λ_2=480 nm (120 s, bulk *cis→trans*). Higher absorbance corresponds to a greater amount of *trans* isomer. Note that this bulk *trans→cis* isomerisation at 380 nm is significantly faster than the bulk *cis→trans* isomerisation at 480 nm, although the monochromator delivers light at 480 nm with approximately 1.5 times the intensity of the light delivered at 380 nm (**Fig S4**).

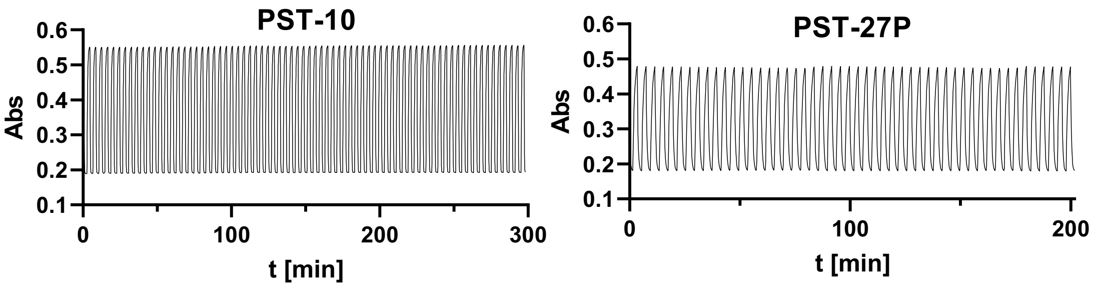

Figure S3 - Reversible trans↔cis photoisomerization of **PST-10** and **PST-27P** over many cycles of alternating 380 and 480 nm irradiation.

4: Conjugates MR69 / MR110: absorption and E↔Z photoisomerisation

The absorbance spectra of the *trans* and *cis* conjugate **MR69** are bimodal, with the region below 440 nm dominated by the azobenzene moiety and the region above dominated by the fluorophore moieties, while **MR110** has significant overlap of azobenzene and fluorophore spectral components across the UV/blue range (**Fig 5b**). The spectra of both conjugates are however approximately the sum of the spectra of their fluorophore (model compounds **RhodPip** and **NBDMor**[61]) and their azobenzene (model compounds **PST-28** and **PST-27**) (**Fig 5b, S7**). The fluorescence behaviour of the conjugates was also similar to that of their model fluorophores (**Fig S5**).

The photoisomerisation behaviours of **MR69** or **MR110** were however substantially different both from that of pure **PST-30** or **PST-29** in solution, as well as from 1:1 mixtures of **PST-30**+**RhodPip** or **PST-29**+**NBDMor** (called the “Mix” samples).

Irradiation of a dark-adapted (all-*trans*) **MR69** sample, especially around its absorption maximum at 564 nm, gave visible fluorescence, without the absorbance spectrum of the sample in the region 360-450 nm being greatly changed. This is consistent with the hypothesis that irradiation at this wavelength does not substantially decrease the population of the *trans* form (more accurate measurements indicate it drives a PSS around 95%*E*). Irradiation at 380 nm, intended to mainly excite the azobenzene, induced rapid bulk *trans→cis* photoisomerisation to approximately 45% *cis*. Subsequent selective excitation of the rhodamine moiety around 560 nm (with comparable intensity) then resulted in near-quantitative (ca. 95%) *cis→trans* photoisomerisation, with markedly faster time constant than for the isomerisation at 380 nm. Repeated switching by alternating between 384 nm and 554 nm illustrates this difference (**Fig S4**).

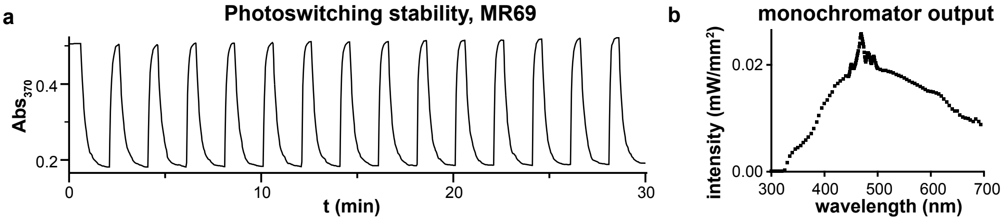

Figure S4. **(a)** Repeated switching of **MR69** by alternating between 384 nm and 554 nm while monitoring the absorbance at 370 nm. **(b)** Power spectrum of the monochromator light source used for this and other precise-wavelength switching experiments (e.g. switching in **Fig 5c** and **Fig S3**; and photo-rates in **Fig 5d**). The relative photo-rates in **Fig 5d** are calculated as $1/(\tau_\lambda I_\lambda)$ where τ_λ is the half-time for photoisomerisation from PSS_{380} to PSS_λ, and I_λ is the relative intensity (in mW/mm^2) of the monochromator at that wavelength: these usefully report on the *photon efficiency of isomerisation* over the spectral response range (in terms of fractional approach to PSS).

The unusual rapidity and quantitative nature of the *cis→trans* photoisomerisation should especially be compared with the slower and non-quantitative *cis→trans* photoisomerisations of non-conjugated **PST-30** under 500 nm to approximately 85% *trans* (**Fig 5d**). Excitation of **MR69** at 560-600 nm also enables fast *cis→trans* photoisomerisation of the azobenzene in a region where the non-conjugated **PST-30** does not detectably respond to irradiation at this intensity. This red-shifting and simultaneous boost of isomerization efficiency brings **MR69** closer to the biooptical transparency window, improving *in vivo* applications of azobenzenes.

5: Conjugates MR69 / MR110: fluorescence

All UV-Vis and fluorimetry photocharacterisations of **MR69** (and of its model fluorophore and switch, for comparisons) were collected in PBS with 20% MeCN cosolvent unless otherwise

described. Due to the poor aqueous solubility of **MR110**, all UV-Vis and fluorimetry photocharacterisations of it (and of its model fluorophore and switch, for comparisons) were collected in 20:80 MeCN: DMSO unless otherwise described.

Fluorescence excitation and emission spectra were acquired for the conjugate and compared to those of the model fluorophore (NBD-morpholine **NBDMor** or Rhodamine B (*N'*-acetamido)piperazinamide) **RhoPip** (**Fig S5**). The results show almost identical fluorescence spectral characteristics between conjugated and free fluorophores, meaning there is little spectral bias between them and therefore supporting the use of the model fluorophore's spectrum in deconvolution of the conjugate's spectrum (see PSS Characterisation).

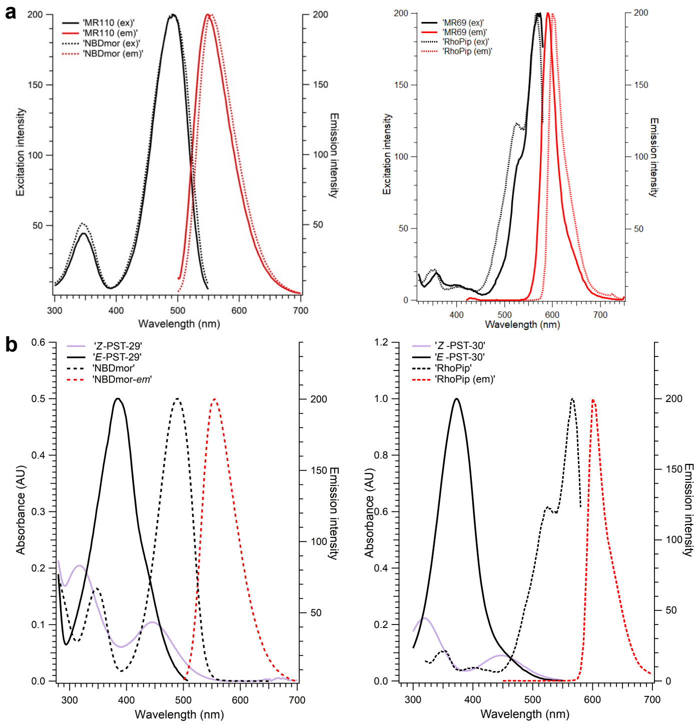

Figure S5 – **(a)** Fluorescence excitation and emission spectra of conjugate **MR110** and model fluorophore **NBDMor** [emission spectra collected with excitation at 495 nm; excitation spectra collected measuring emission at 500-700 nm]; and of conjugate **MR69** and model fluorophore RhodPip [emission spectra collected with excitation at 380 nm which was confirmed to match exactly the emission spectrum produced with excitation at 554 nm; excitation spectra collected measuring emission at 590 nm]. **(b)** Fluorescence properties (ex/em) of **NBDMor** (left panel), and RhodPip (right panel) compared to absorption spectra for their *E* and *Z* isomers of **PST-29** and **PST-30**.

PSS Characterisations

PSS Measurement Procedure

Having first obtained the fully relaxed sample (all-*trans*) by overnight relaxation (half-lives are ca. 2.5 h at 22 °C for **MR110** and ca. 3 h at 37 °C of **MR69** respectively), then measured its absorbance by UV-Vis, the sample was then subjected to LED illuminations to establish different PSS spectra (in between each spectrum, 390/395 nm light was used to revert the state to mostly-*cis* so that it could be confirmed when a wavelength caused cis to trans reversion, or not). For **MR110**, the procedure was separately applied to samples of the conjugate, to the "physical mix" (1:1 ratio of model fluorophore and model azobenzene switch), and to the isolated azobenzene "switch", to enable comparisons later, given the strong overlap of these two chromophores. Data are shown in **Fig 5** and **Fig S6**.

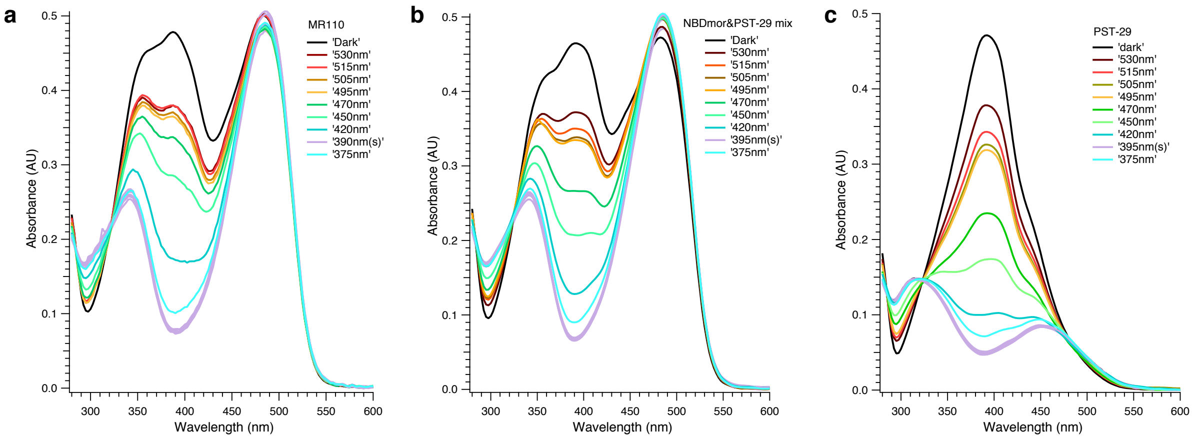

Figure S6. PSS absorption spectra of (a) **MR110** and its related (b) physical mix and (c) switch.

PSS Analysis – Assumption-free bounds

The *relative completeness* of photoreversion towards the all-*trans* state under different illuminations was examined first. The ratios [[P(λ)-MC(λ)]/[D(λ)-MC(λ)]] were calculated, where P(λ) is the PSS absorption spectrum being evaluated, MC(λ) is the PSS absorption spectrum under the wavelength giving the most-*cis*-containing PSS, and D(λ) is the all-*trans* absorption spectrum. This "relative completeness fraction" was calculated across the data range where the variation in absorbances with different PSSs is strongest (typically 370-420 nm), then the data were averaged to give the mean, and their standard deviation determined as a measure of the error in this fitting method. For **MR110**, the 395 nm PSS was the most-*cis* PSS measured. This procedure was performed for the conjugate, the free azobenzene switch, and the physical 1:1 mixture of azobenzene switch and model fluorophore. Results are shown in **Table S2** below. These completeness fraction values are (by definition) lower bounds for the PSS values of *E*-content, so calculating (1-fraction reconversion) determines **upper bounds** for the PSS values of *Z*-content.

Lower bounds for the PSS *Z*-content values may separately be obtained by assuming the absorption of the *cis* isomer is zero at λ_{strong} and then tabulating $A(\lambda_{strong,\ PSS})/A(\lambda_{strong,\ all\text{-}trans})$. This yields assumption-free bounded ranges for the true PSSs. This will be seen to be more than sufficient to give a PSS range with typically only 5% possible error at the wavelengths of most interest to this study (470 nm and above) - see **Table S3** in the following section.

Table S2. Fraction reconversion from PSS-395 towards the all-*trans* states under the given wavelengths for **MR110** and its related samples. Data ranges used for the fit calculation are specified as well as the standard deviations of the mean fit fractions so determined. These fractions are lower bounds for the PSS %*E* and as such are used to calculate the %*Z* upper bounds ("UB") in **Table S3**.

	**MR110**		**Mix**		**PST-29**	
**λ / nm**	**Fraction**	**StDev**	**Fraction**	**StDev**	**Fraction**	**StDev**
**530**	0.75	0.006	0.77	0.004	0.78	0.002
**515**	0.75	0.006	0.71	0.006	0.69	0.002
**505**	0.72	0.012	0.69	0.006	0.65	0.004
**495**	0.71	0.012	0.68	0.007	0.64	0.004
**470**	0.64	0.012	0.50	0.008	0.43	0.008
**450**	0.51	0.015	0.35	0.009	0.30	0.003
**420**	0.24	0.012	0.15	0.006	0.12	0.002
**375**	0.06	0.010	0.06	0.004	0.05	0.003
data range	*360 - 425 nm*		*360 - 420 nm*		*355 - 432 nm*	

PSS Analysis – Fitting with separated spectra from HPLC

The absolute PSS values cannot be determined from the preceding absorbance measurements alone, as they require a reference point to determine the “all-cis” spectrum. To this purpose, the spectra of the *E* and *Z* isomers of the azo switch could be obtained on an inline DAD detector of an HPLC (albeit in different solvent environment to that used in the UV-Vis measurements), and the assumption made that these spectra approximate the spectral contributions of the azo switch moiety in their respective conjugate (in the UV-Vis experiments). For PSS analysis by fitting of this HPLC data it is also necessary that the absorption spectra of the switch and fluorophore components in the conjugate act additively to produce the overall absorbance of the conjugate; this can be seen in **Fig 5c** and **Fig S7**.

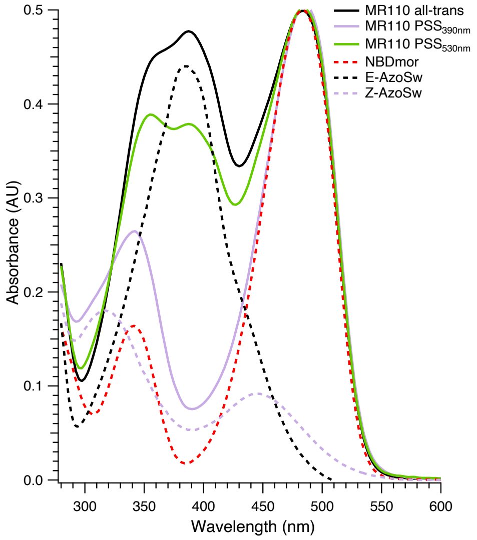

Figure S7. Absorption spectra of conjugates **MR110** at limiting PSSs (**MR110**: 395 and 530 nm) and in the all-*trans* state, compared to those of the *E* and *Z* states of the azobenzene switch (pure spectra, from HPLC with inline DAD) and to the model fluorophore **NBDmor**. This indicates the rough additivity of component spectra in deriving the PSS absorption spectra of the conjugates. (The spectrum of **NBDMor** was translated hypsochromically by 6 nm to match the absorption maximum of the conjugate; we attribute this to a small environment-sensitive translation of the absorbance profile, as known for NBD fluorophores). A similar additivity of component spectra applies for **MR69** in which, correspondingly, the rhodamine absorbance dominates the spectrum of the conjugate.

Under these assumptions, the pure-*Z* spectrum from the HPLC therefore can serve as a reference point to establish the absolute values of the PSS *Z*-contents measured on the UV-Vis previously. This determination proceeds as follows. The absorbance spectra of each sample (conjugate / mixture / isolated switch) at all PSS wavelengths, and of the model amine fluorophore, have already been measured on the UV-Vis. For each sample we now subtract the absorbance spectrum of each PSS state from that of its corresponding all-trans state defining a difference spectrum notated eg. $\Delta 450(\lambda)$ for the (dark - PSS450) spectrum. Noting that if component spectra are additive, then for any sample, at arbitrary PSS wavelength W, we have:

$$(1) \qquad \Delta W(\lambda) = Q_W \times [Z\text{-PST}(\lambda) - E\text{-PST}(\lambda)]$$

where Q_W is the PSS fraction of *cis* (note, $\Delta W(\lambda)$ are plotted for **MR110** in **Fig S8a**).

To bring in the HPLC E and Z spectra to the analysis, they are first interscaled relative to each other by matching their absorbances at the UV-Vis-determined isosbestic point (Fig S8b). Then, the interscaled HPLC traces can be used to determine a difference spectrum $E\Delta Z(\lambda)$ which under assumption of additivity of spectra results in:

$$(2) \qquad E\Delta Z(\lambda) = B \times [Z\text{-PST}(\lambda) - E\text{-PST}(\lambda)]$$

where B is a scaling factor (the red dotted curve in **Fig S8b** plots $E\Delta Z(\lambda)$ for **MR110**'s switch). We now vertically scale $E\Delta Z(\lambda)$ to match $\Delta W(\lambda)$, by subtracting the model fluorophore absorbance spectrum from the conjugate's all-trans spectrum and fitting the HPLC *E*-spectrum of the switch to the residual (**Fig S8c,e**), then applying the fitting factor as scaling factor B.

We can then use the now-scaled $E\Delta Z(\lambda)$ to solve for PSS values Q_W in the samples' difference spectra $\Delta W(\lambda)$, by best-fitting $q_w \times E\Delta Z(\lambda)$ [plotted in **Fig S8d** for several values of q_w] to the spectra $\Delta W(\lambda)$. **Fig S8f** plots several such fits for **MR110**.

This procedure relies on the additivity of spectra at relatively high dilution for the physical mixture, on the small variance of spectra with solvent nature between UVVis and HPLC measurements for the isolated switch, and on the additivity of component spectra for the fluorophore-azobenzene conjugate. This last could be seen as the most error-prone assumption although for the compounds tested, a strictly unbiased fitting procedure returned best-fit PSS values that were all entirely within the bounded range established through assumption-free measurements as outlined above, supporting the physical reasonableness of this PSS fitting procedure. Also, the additivity of the component model fluorophore and azobenzene switch spectra for deriving the overall spectrum of the conjugate is supported by eg. the match of form of the curves in **Fig S8e**. The sets of PSS *Z*-content estimations for each sample are tabulated in **Table S3**, alongside the upper and lower bound PSS values determined in the previous section.

Table S3. Assumption-free upper and lower bounds (UB and LB respectively) for experimentally determined *Z*-content PSS values together with HPLC fitted PSS estimation for **MR110**.

	MR110			PhysMix			PST-29		
**λ / nm**	**UB**	**Fit**	**LB**	**UB**	**Fit**	**LB**	**UB**	**Fit**	**LB**
**530**	0.25	**0.23**	0.21	0.23	**0.23**	0.20	0.22	**0.22**	0.20
**515**	0.25	**0.23**	0.21	0.29	**0.28**	0.25	0.31	**0.30**	0.27
**505**	0.28	**0.26**	0.23	0.31	**0.31**	0.27	0.35	**0.34**	0.31
**495**	0.29	**0.27**	0.24	0.32	**0.32**	0.28	0.36	**0.36**	0.32
**470**	0.36	**0.33**	0.30	0.50	**0.49**	0.43	0.57	**0.55**	0.50
**450**	0.49	**0.45**	0.40	0.65	**0.63**	0.55	0.70	**0.70**	0.63
**420**	0.76	**0.71**	0.64	0.85	**0.82**	0.72	0.88	**0.85**	0.79
**395**	(1)	**0.94**	0.84	(1)	**0.97**	0.86	(1)	**0.97**	0.89
**375**	0.94	**0.88**	0.79	0.94	**0.92**	0.81	0.95	**0.92**	0.85

A graphical summary of the HPLC-involving-PSS-fitting procedure is given below in **Fig S8** for interest, for **MR110**.

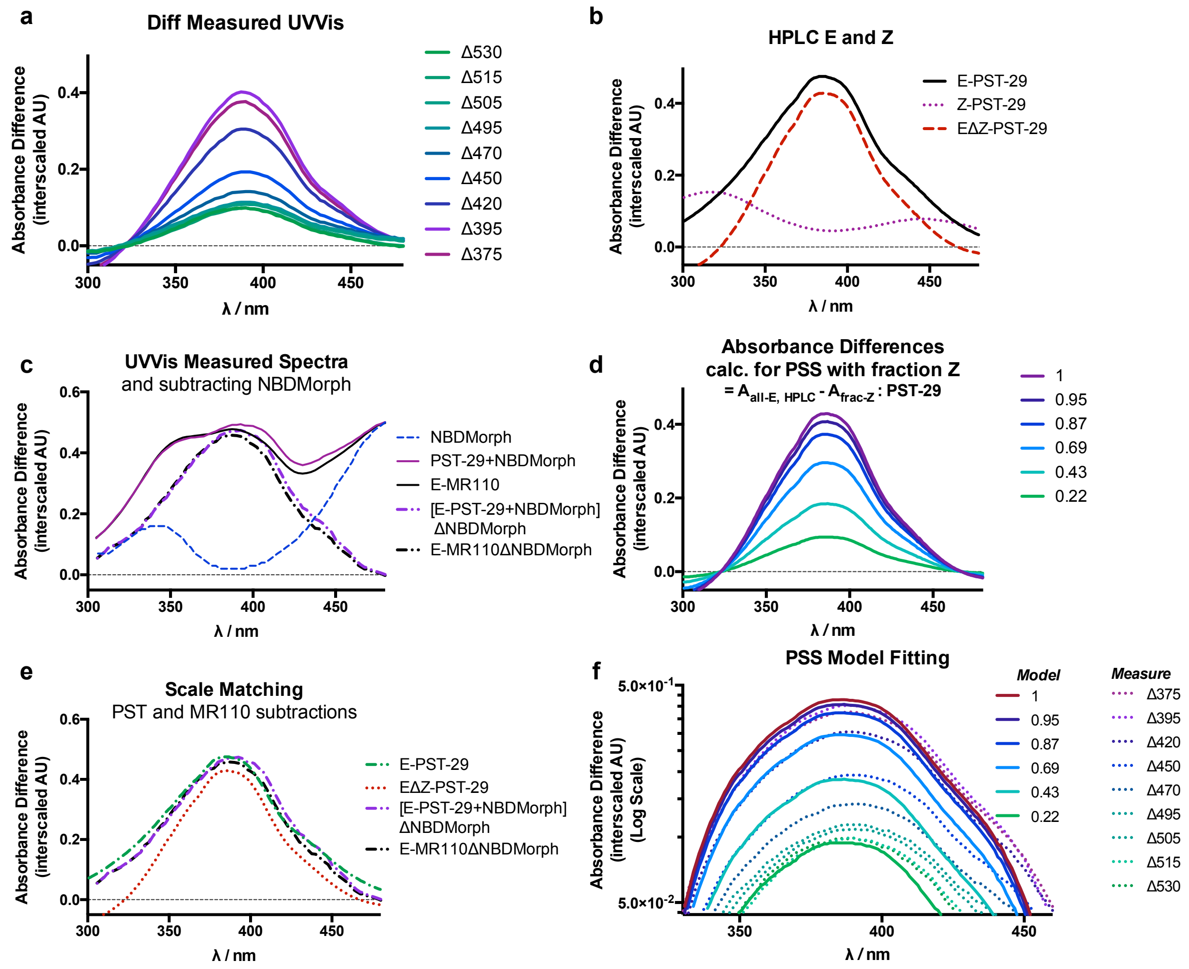

Figure S8. PSS model fitting procedures for **MR110**.

Part C: Cell Biology

1. In vitro tubulin polymerisation assay (Fig 4cd)

Tubulin from porcine brain with 30% MAPs (microtubule-associated proteins) was obtained from Cytoskeleton Inc. (Cat. # ML116, Denver, CO, USA). The polymerisation reaction was performed at 2 mg/mL tubulin, in polymerisation buffer (80 mM piperazine-N,N′-bis(2-ethanesulfonic acid) (PIPES) pH=6.9, 0.5 mM EGTA, 2 mM $MgCl_2$), in a cuvette (100 µL, 1 cm path length) in a Varian CaryScan 50 with Peltier cell temperature control unit maintained at 37 °C. Tubulin was first incubated for 10 min at 37 °C with an inhibitor of interest (in 1 % MeCN and 0.5 % DMSO to ensure solubility) in buffer without GTP. Then GTP was added to 1 mM final concentration, and the change in absorbance at 340 nm (Lin et al., 1988) was monitored for 30 min. Throughout the whole experiment samples were kept shielded from ambient light ("dark") or exposed to illumination at 390 nm using the TILL Photonics Polychrome V monochromator with the output of fibre optic cable directed into the cuvette.[26]

2. Cell culture under controlled light conditions (Fig 3-4)

HeLa (DSMZ; ACC57) cell line was purchased from the German Collection of Microorganisms and Cell Cultures. HeLa cells were grown in Dulbecco's modified Eagle's medium (DMEM, PAN-Biotech P04-03550) supplemented with 10% heat- inactivated fetal calf serum, 100 U/mL penicillin and 100 µg/mL streptomycin (Sigma Aldrich, P4333). Cells were grown at 37°C in a 10% CO_2 atmosphere. Prior to assays, cells were transferred to phenol red free medium (DMEM, PAN-Biotech P04-03591). In order to ensure precise light conditions, cells were incubated in light-proof boxes in the dark or upon pulsed illuminations, using a home-made "Disco" LED lighting system.[26] In brief, Disco uses an Arduino microcomputer to pulse arrays of LEDs placed under well plates, to illuminate them with regular flashes during a long-term incubation experiment. Typically, cells were illuminated with 75 ms pulses every 15 s. The following wavelengths of LEDs were used in this study (in nm): 360, 370, 380, 390, 400, 410, 425, 450, 475, 490, 505, 515, 525, and 535; all LEDs were bought from Roithner Lasertechnik as 4V / 20 mA / 5 mm units; the Roithner part numbers are XSL-360-5E, VL370-5-15, RLS-UV380, VL390-5-15, RLS-UV400, VL410-5-15, VL425-5-15, LED450-04, B56L5111P, LED490-03, B5-433-B505, LED515-10-30, B5-433-B525, and LED535-01 respectively.

3. MTT viability assay (Fig 3)

Mitochondrial dehydrogenase activity, as a proxy for viable cells, was quantified by spectrophotometrically measuring the reduction of 3-(4,5-dimethylthiazol-2-yl)-2,5-diphenyl tetrazolium bromide (MTT) to formazan, according to standard protocol[62]]. In brief, cells were seeded on 96-well microtitre plates. After 24 h inhibitors were applied using the same organic cosolvents for all samples (1% DMSO and 2% acetonitrile), in order to ensure full solubility of all compounds and intercomparability of the data. Cells were exposed to the appropriate light regimes or shielded from ambient light with light-proof boxes. Following 48 h of treatment, cells were incubated with 0.5 mg/mL MTT for 3 hours at 37 °C. The formazan crystals were dissolved in DMSO, and absorbance at 550 nm was measured using a FLUOstar Omega microplate reader (BMG Labtech). For statistical analysis, the absorbance readings for untreated controls (cosolvent only) were set as 100% of viable cells. Results are given as the mean percentage of viable cells relative to these controls, +/- standard deviation (SD) from a representative experiment performed in sextuplicates. Graphical representations and statistical analysis of the data were performed using GraphPad Prism (GraphPad Software, La

Jolla, CA, USA) (**Fig S9**). Four-parameter curve fitting for sigmoidal dose–response curves with variable slope was used, unless sigmoidal curve fitting was not appropriate in which case a linear regression fit was used instead.

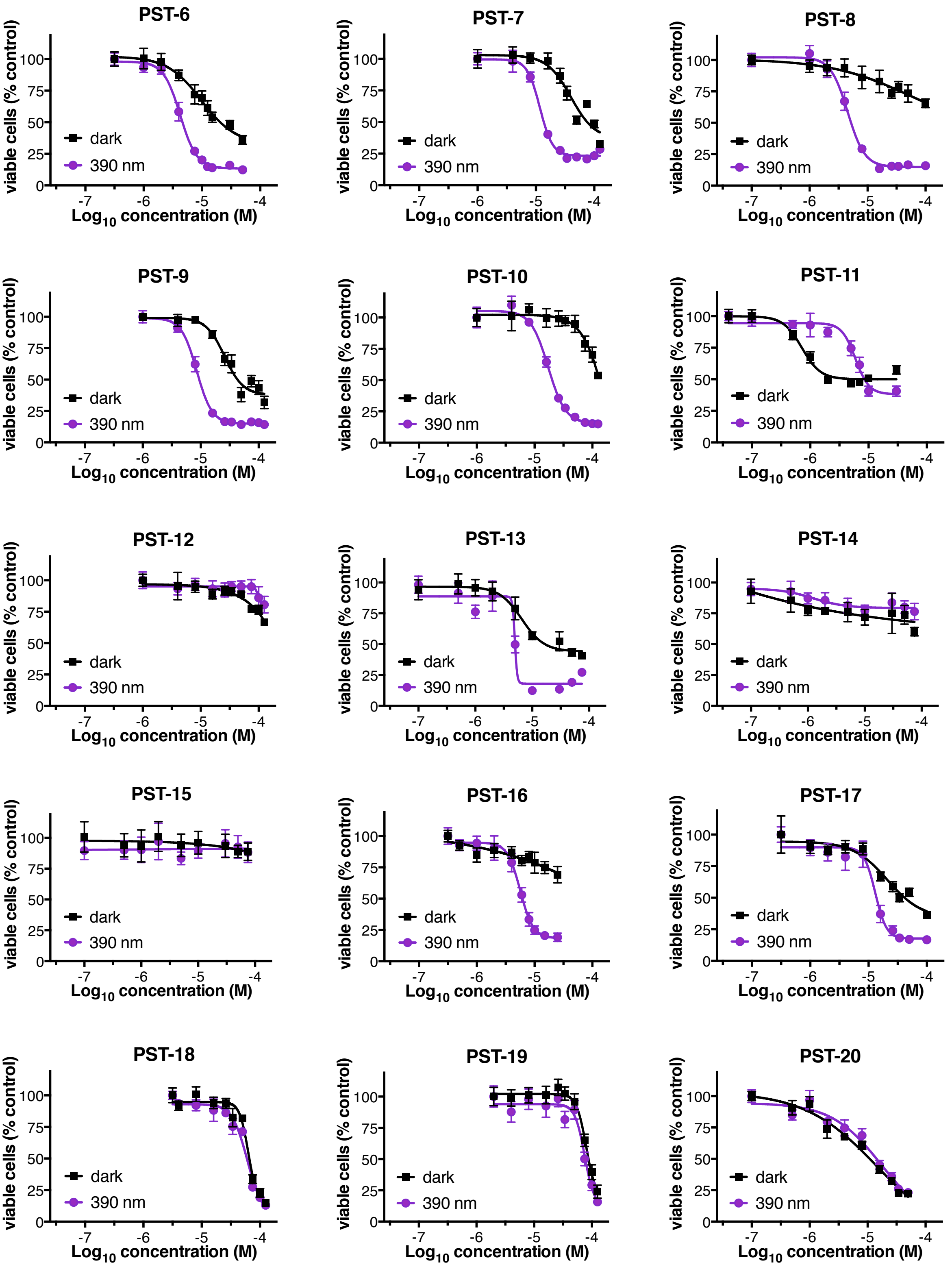

Figure S9, part 1/2. Cytotoxicity determined by MTT assay after 48 h incubation in HeLa cells, under dark and under 390 nm illumination, for the main compounds of this panel of **PSTs**.

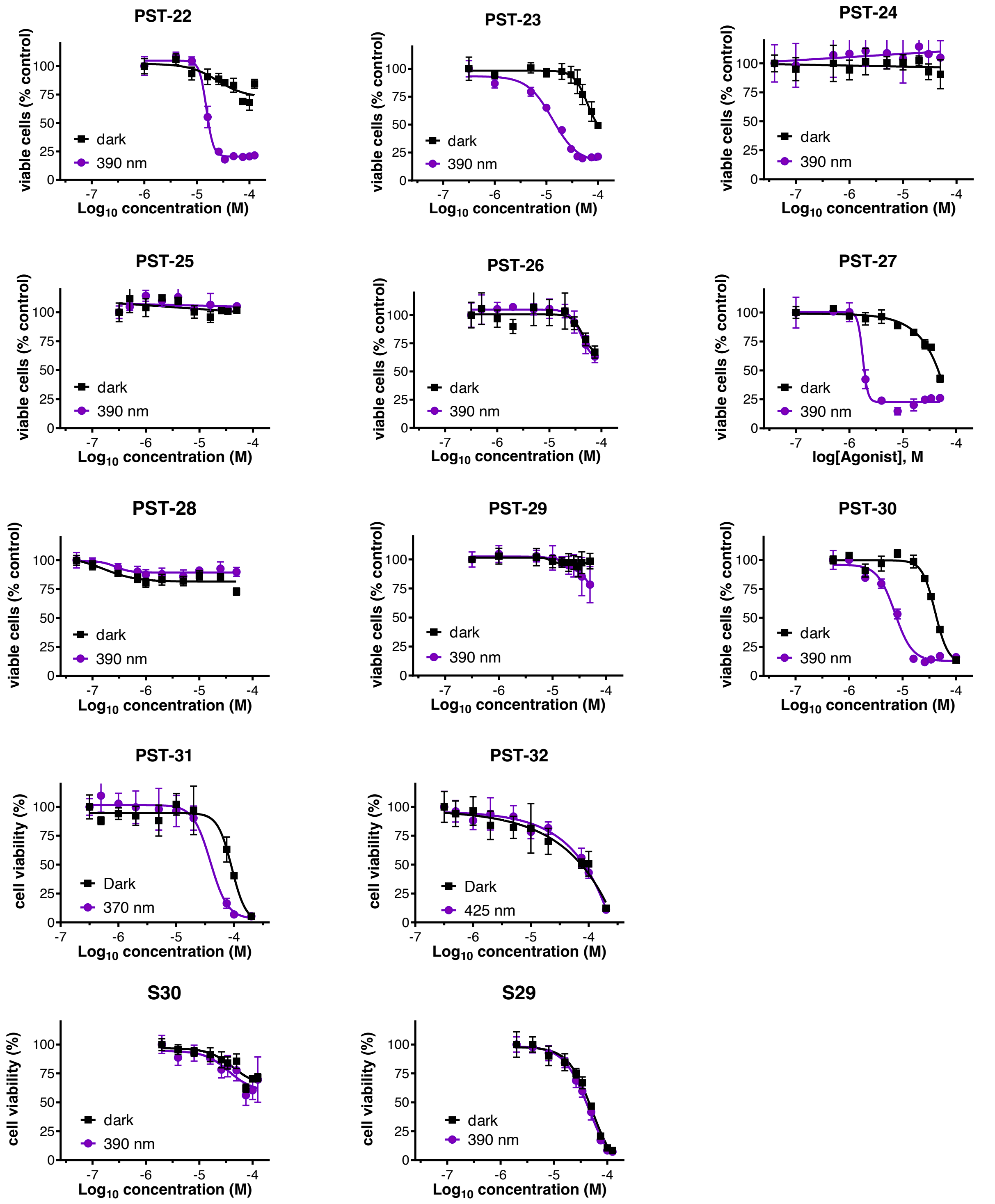

Figure S9, part 2/2. Cytotoxicity determined by MTT assay after 48 h incubation in HeLa cells, under dark and under 390 nm illumination, for the main compounds of this panel of **PSTs**.

The EC_{50} values of percentage cell viability were determined for sigmoidal fits to the 390 nm illumination condition, except for **PST-11** (determined for dark), or where no fit was possible (e.g. **PST-14**) in which case it was marked as “ND" or "n.d.” with the highest tested concentration that maintained satisfactory solubility indicated, e.g. “ND (> X μM)”. The EC_{50} values for dark, treated controls were then taken at the determined EC_{50} percentage of cell viability; when the viability percentage was not reached it was marked as “ND (> X μM)”.

4. Confocal microscopy (fixed samples) (Fig 4ab)

HeLa cells were seeded on coverslips and incubated for 20 h with test compounds under dark or toxic regime (390 nm pulses of 75 ms every 15 s) using the Disco system. Then, cells were exposed to cell extraction buffer (80 mM PIPES pH 6.8; 1 mM $MgCl_2$, 5 mM ethylene glycol tetraacetic acid (EGTA) dipotassium salt and 0.5% Triton X-100) in order to remove monomeric and dimeric tubulin subunits (thus, the lower green channel signal intensities in some cells reflect that they were exposed to tubulin-depolymerising compound action). After 30 s of extraction, cells were fixed with 0.5% glutaraldehyde, which was subsequently quenched with 0.1% $NaBH_4$. Coverslips were blocked with PBS containing 10% FCS, and subjected to immunostaining using anti-α-tubulin antibody (ab18251) and AlexaFluor 488 secondary antibody (A 11008), purchased from Abcam (Cambridge, UK) and Invitrogen (Darmstadt, Germany), respectively. Hoechst 33342 (bisbenzimide), purchased from Sigma Aldrich (Taufkirchen, Germany), was used at a concentration of 1 µg/mL for nuclear staining. Cells were mounted with FluorSave reagent (Calbiochem) and analysed with a Leica SP5 confocal microscope. Images were processed using ImageJ open-source software (National Institutes of Health). Representative images are shown. White scale bars correspond to 20 µm.

5. Nuclear fragmentation and cell cycle analysis (Fig 4ef)

Following compound treatment, cells were harvested on ice and incubated in a hypotonic buffer [0.1% sodium citrate, 0.1% Triton X-100 and 50 µg/mL propidium iodide (PI)] for 30 min at 4 °C. Following the PI staining cells were analysed by flow cytometry using a BD FACS CantoII flow cytometer (Becton Dickinson, Heidelberg, Germany) run by BD FACSDiva Software (Becton Dickinson). The cell cycle analysis was subsequently performed using FlowJo software (Tree Star Inc., Ashland, OR, USA) employing the Dean-Jett-Fox fitting model. Cells with subdiploid DNA content (below G_1 phase content) were considered as apoptotic in accordance with Nicoletti (Nicoletti et al., 1991). Results represent means +/- SD calculated for triplicates from one representative experiment out of three independent trials.

6. Live cell imaging (Fig 6bc)

The localization of NBD-conjugate **MR110** and rhodamine-conjugate **MR69** was assessed by live-cell imaging. HeLa cells were plated on 8-well ibiTreat µ ibidi slides (ibidi, Martinsried, Germany) and twenty-four hours later **MR69** and **MR110** were applied at respectively 0.5 µM and 1 µM (using 0.5 % MeCN as cosolvent to ensure solubility). For **MR69** MitoTracker Green FM (M7514, Thermo Fisher Scientific) was applied at the same time. Following 3 h of incubation, cells were imaged using an UltraVIEW VOX spinning disk confocal microscope (PerkinElmer) operated with the Volocity software. **MR110** was imaged using 488nm laser line and 527 (+/- 55 nm) emission filter. **MR69** was excited using 561 nm laser, and emitted light was collected using dual bandpass 525 (+/- 55 nm); 640 (+/- 120 nm) emission filter. During imaging cells were maintained at 37°C in a 5% CO_2 atmosphere. Representative images are shown in **Fig 6**, the scale bar corresponds to 20 µm. **MR110** was preferentially accumulated in lipid internal membranes around the nucleus (but was absent from the nucleus itself). **MR69** was localized to mitochondria as indicated by co-staining with the carbocyanine dye MitoTracker Green. The statistical significance of co-localization was determined using JACoP open-source ImageJ plugin.[63] The Pearson's correlation coefficient r=0.916, and Manders split coefficients M1=0.927 and M2=0.937 show nearly perfect correlation between the fluorescence intensity distributions in the two colour channels, supporting the localization of **MR69** to mitochondria.

7. Growth rate monitoring (RFLM) (Fig 1)

A549 (DSMZ; ACC 107) cell line was purchased from the German Collection of Microorganisms and Cell Cultures. The growth rate of A549 cells (cultured in DMEM containing 10% FBS at 37 °C, 95% humidity and 5% CO_2) was monitored with reconstruction-free lensless microscopy (RFLM) using a customised RFLM setup (PHIO scientific GmbH; Munich, Germany; **Figure S10**): a ca. 15×10×8 cm device that can be placed in a standard cell culture incubator, where it continuously images eight ca. 10 mm^2 fields of view (FOVs), in parallel, one per chamber with standard 8-chamber imaging slides (ibidi GmbH; Martinsried, Germany). Based on lensfree cell imaging with red (630 nm) light [to avoid photoswitching by imaging], up to ca. 10.000 cells per FOV can be tracked for movement, division, and shape; this allows reading out parameters such as the cell count over time, or the percentage surface area of the FOV that is covered by cells (proxy readout for cell confluency); or, equivalently, the first derivative of these readouts monitors the rate of cell growth at any given time. The RFLM setup was equipped with low-intensity near-UV LEDs for *in situ E→Z* photoswitching of **PSTs** (LEDs: RLS-UV380, 4V / 20 mA 5 mm package; Roithner Lasertechnik GmbH, Vienna, Austria; applying ca. 0.3 mW/cm^2 at the chamber during on-phases). The UV LEDs were controlled by an Arduino microcontroller to ensure that they were switched off during image acquisition. Cells were pulsed with 380 nm under relatively standard pulsing conditions used in long-term experiments (500 ms ON every 60 s), during phases of the indicated durations (0.3 h, 1 h or >6 h). To interpret this data, it must be kept in mind that (a) cells seeded at even slightly different confluencies will experience different growth dynamics; (b) the cells' natural doubling time is around 16-20 h, so a pause in cell confluency at around this time after the start of experiment is expected (cells were seeded at time zero, so that a large range of confluency could be explored during the 28 hour experiment, without reaching contact inhibition).

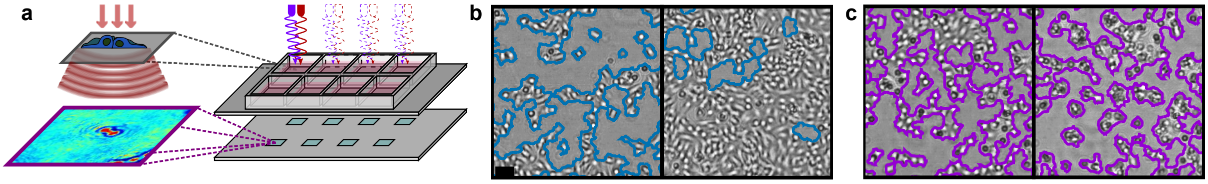

Figure S10. RFLM produces holographic near-field scatter images that look different to fluorescence or transmitted light imaging, but which report especially sensitively on which cells are adherent (healthy) or detached (indicative of cell death, except during mitosis). **(a)** Schematic of RFLM setup. **(b,c)** Representative RFLM images of cell confluency at start (left) and end (right) of two assays: **(b)** shows unimpeded cell growth to near-full-confluency, **(c)** shows impeded cell growth with substantial induction of cell detachment and death. Both panels show the auto-detected boundaries of cell coverage (= %confluency, solid lines in **Figure 1** which are plotted on the left-hand axes). The instantaneous derivative of %confluency over time is the growth rate (dotted lines in **Figure 1** which are plotted on the right-hand axes). Since no light is *absorbed* for RFLM image generation, it is a particularly noninvasive imaging technique that does not cause phototoxicity, and can therefore be performed continuously over days without impacting cell health (unlike e.g. fluorescence imaging). Panels (**b,c**) are zoom-ins on a small fraction of the field of view acquired by each camera; typically, up to 5000 cells (at full confluency) contribute to each trace. Since parameters such as growth rate at low confluency are essentially *per-cell* parameters, this gives high statistical confidence to each data trace (for details of RFLM statistical robustness and validation, see ref [64]).

Part D: Structure of *Z*-PST-27 bound to tubulin-DARPin D1 complex

Table S4. Crystallographic data and refinement statistics for *Z*-**PST-27** bound to tubulin-DARPin D1 (TD1) complex.

**Data Statistics**	**TD1-Z-PST-27**
Space group	P 2_1
Unit cell (a; b; c; β) (Å)	73.46 91.12 82.68 97.61
Wavelength (Å)	1.0
Resolution (Å)	44.618 – 1.905
R_{pim} (%)	5.9 (43.9)
I/σI	13.4 (1.8)
Spherical completeness (%)	78.2 (22.3)
Ellipsoidal completeness (%)	95.1 (85.2)
Ellipsoidal truncation resolution limits (Å)	1.82, 2.11, 2.17
Multiplicity	6.1 (3.7)
$CC_{1/2}$	0.990 (0.697)
**Refinement Statistics**	
Resolution (Å)	44.62 – 2.20
No. Reflections	54617
R_{work} / R_{free}	18.83% / 22.56%
Ramachandran favored	97.72 %
Ramachandran outliers	0.10%
R.m.s.d. Bond length (Å)	0.003
R.m.s.d. Bond angles (°)	0.610
PDB Deposition Code	**9F8G**

1. Protein Production, Crystallisation, and Soaking

The DARPin D1 was prepared as previously described by Pecqueur *et al.*,[65]. Tubulin from bovine brain was purchased from the Centro de Investigaciones Biológicas (Microtubule Stabilizing Agents Group, CSIC, Madrid, Spain). The tubulin-DARPin D1 protein complex (TD1) was prepared as previously described [65–67].

The TD1 complex was crystallised overnight by the hanging drop vapor diffusion method (EasyXTal plates (QIAGEN)), drop size 2 µL, drop ratio 1:1), protein complex concentration of 9.8 mg/mL, at 20 °C with a precipitant solution containing 21% PEG 3000, 0.2 M ammonium sulfate and 0.1 M bis-tris methane, pH 5.5. All drops were subsequently hair-seeded with crystalline material obtained in previous PEG screen. Grown crystals were washed from the plates with crystallization solution and transferred into a 0.6 mL Eppendorf tube. Within the tubes, the crystalline material induced a batch-crystallization (overnight, 20 °C) resulting in a sedimented pellet of TD1 crystals. Detailed standard operation procedures for the described protein production and crystal preparation are as reported elsewhere.[67]

Single crystals were transferred into fresh crystallization solution drops of 1.8 µL volume and 0.2 µL of **PST-27** compound dissolved in DMSO (150 mM) was added ([**PST-27**] = 15 mM within the prepared drops). Crystals were soaked and illuminated at 385 nm for 10 minutes to generate *Z*-**PST-27** in situ. In the dark, the crystals were transferred into a crystallization solution drop containing 15 mM *Z*-**PST-27** and 10% glycerol for cryo-protection before being frozen in liquid nitrogen for X-ray diffraction data collection.

2. X-ray Diffraction Data Collection, Processing, and Refinement

Data were collected at beamline X06SA (PXI) at the Swiss Light Source (Paul Scherrer Institute, Villigen, Switzerland). The X-ray beam spot was 20 x 20 μm^2, the flux was 5.5×10^{10} photons/s (40% beam intensity) and the data were collected with an exposure time of 0.02 s and an oscillation range of 0.1 per frame.

Indexing and integration was done with XDS[68]. The cryo-crystallographic rotation data (180°) of two single crystals were cut at 1.9 Å and 2.1 Å respectively and combined using the POINTLESS program in CCP4[69,70]. Due to anisotropy, the data were scaled, merged, and corrected (**Table S4**) using the Staraniso server[71] (http:// staraniso.globalphasing.org/). The structure was solved by molecular replacement using PDB ID **5NQU** as a search model[72]. The ligand and restraints were generated with the GRADE server (http://grade.globalphasing.org/)[73] using their SMILES annotation. The structure was then refined iteratively in PHENIX[74] with manual editing cycles in COOT[75] to a maximum resolution of 2.2 Å. Crystallographic data processing and structural refinement statistics are shown in **Table S4**.

Within the shown 2FoFc_no_fill map, the exact position of one of the ethyl groups (C13/C14) of the south ring is not well defined at 1.0 sigma (**Figure S11**). The modeled orientation became evident upon applying lower sigma levels and considering mFo-DFc Polder maps[55] at a threshold of 3.5 sigma (**Figure S11**).

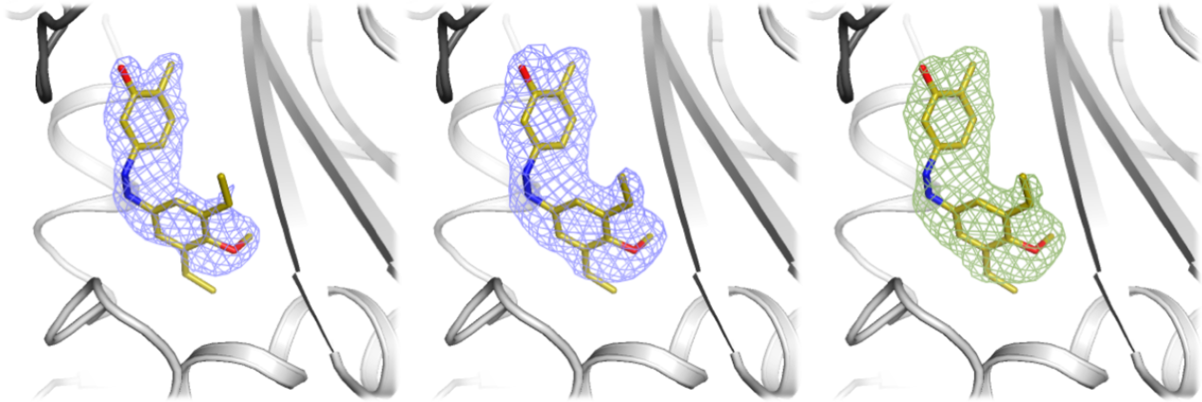

Figure S11. Determining the positions for both ethyl groups for the south ring. Left: 2Fo-Fc_no_fill map at a sigma level of 1.0; Middle: 2Fo-Fc_no_fill map at a sigma level of 0.5; Right: mFo-DFc Polder map at a sigma level of 3.5.

Appendix: NMR Spectra

1-(2-bromo-4-methoxyphenyl)-2-(3,4,5-trimethoxyphenyl)diazene (**PST-6**)

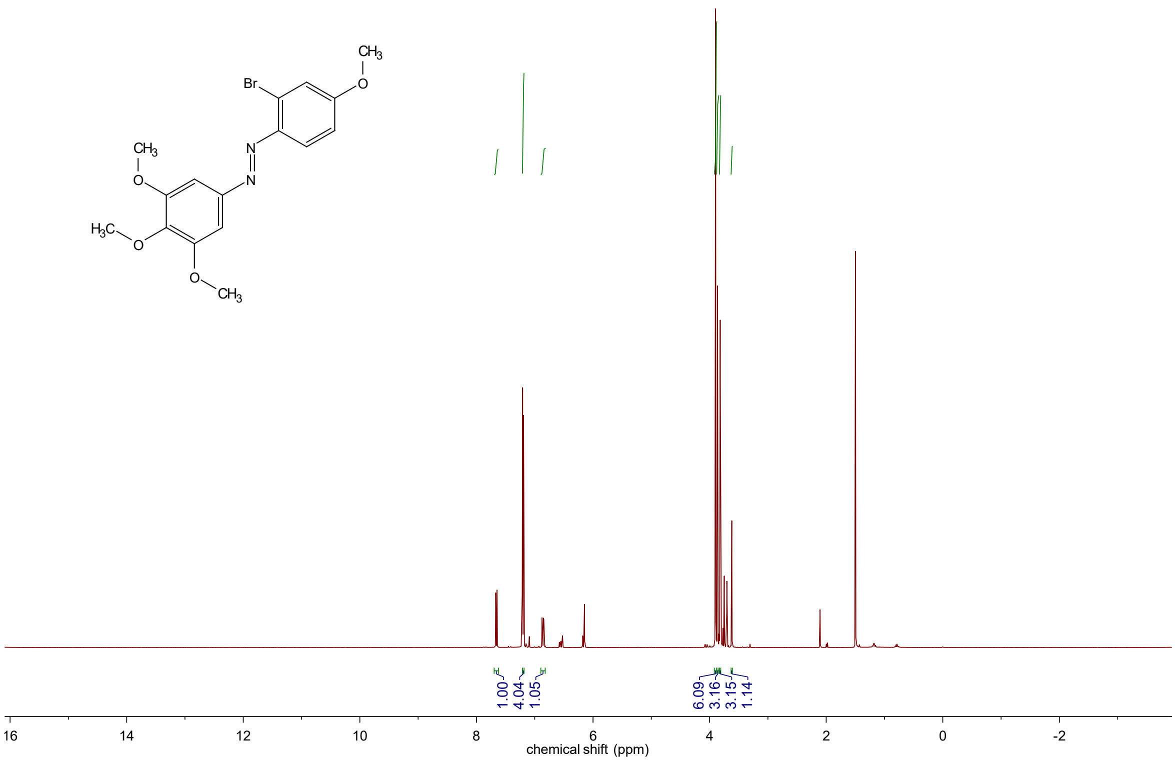

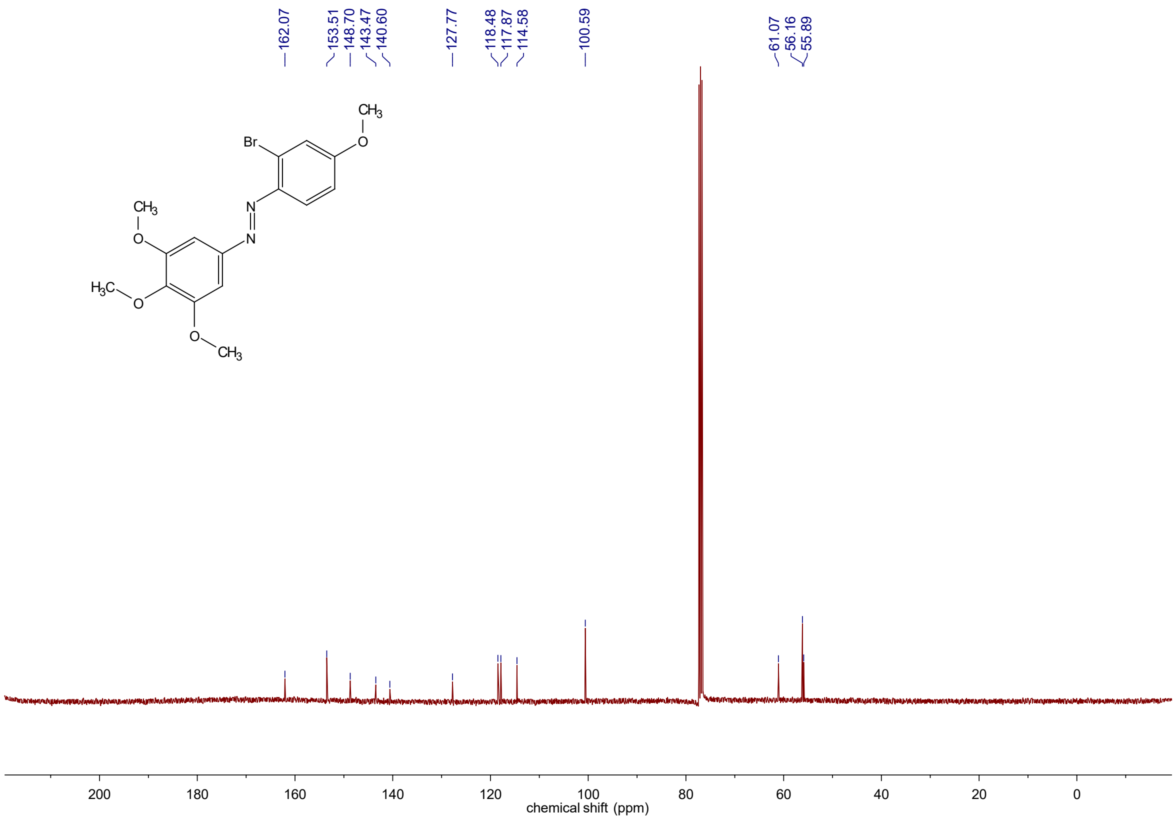

1-(2,4-dimethoxyphenyl)–2-(3,4,5-trimethoxyphenyl)diazene (**PST-7**)

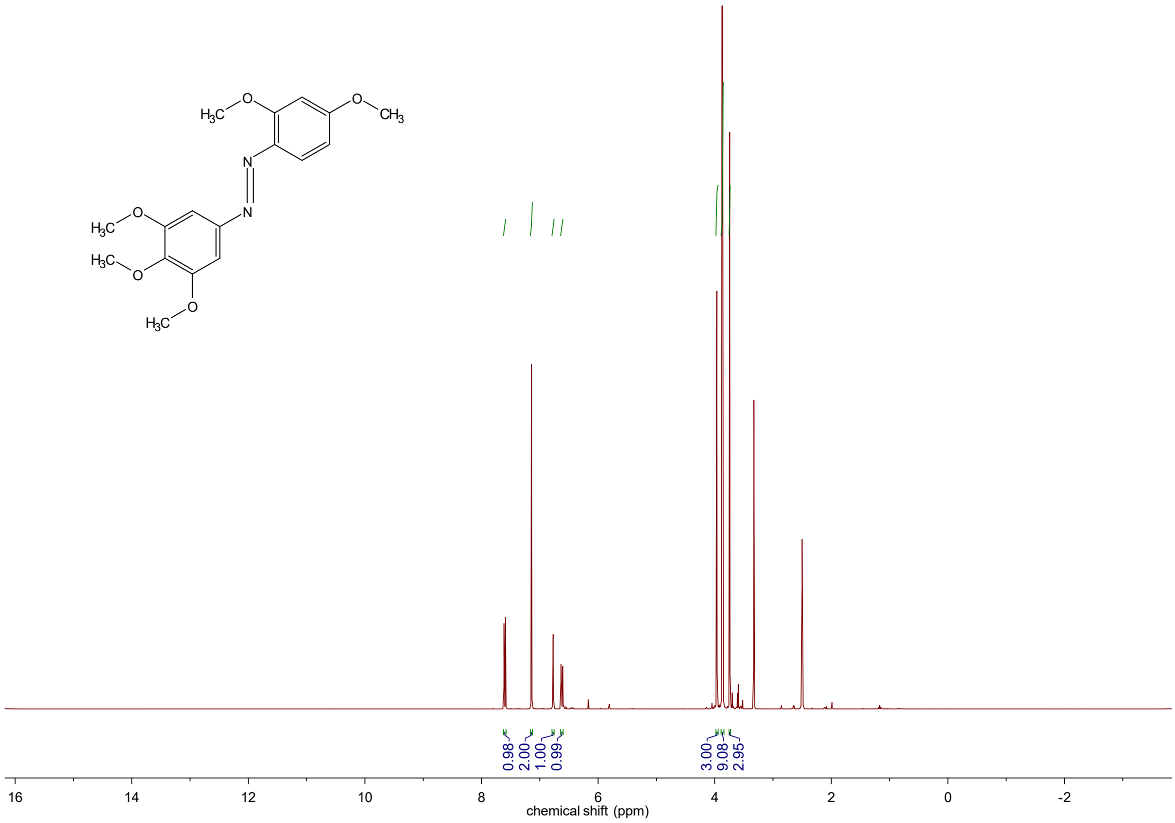

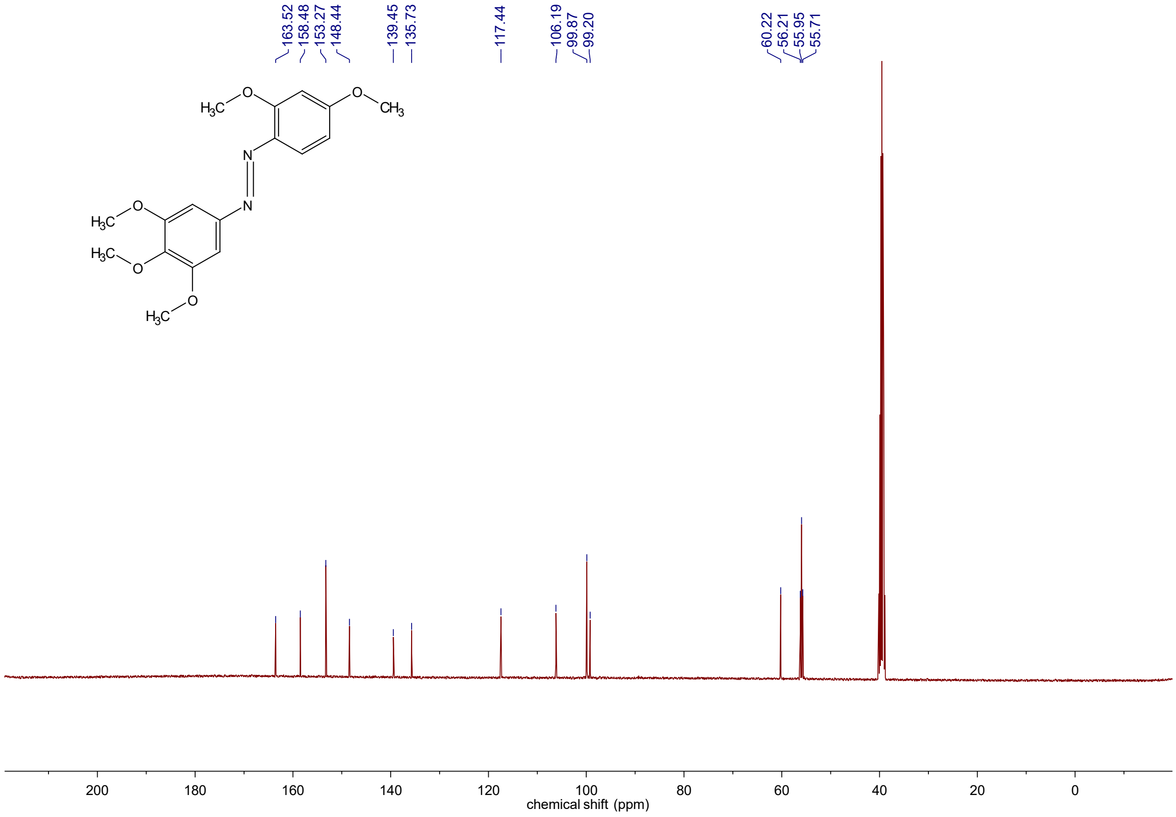

1-(3-bromo-4-methoxyphenyl)-2-(3,4,5-trimethoxyphenyl)diazene (**PST-8**)

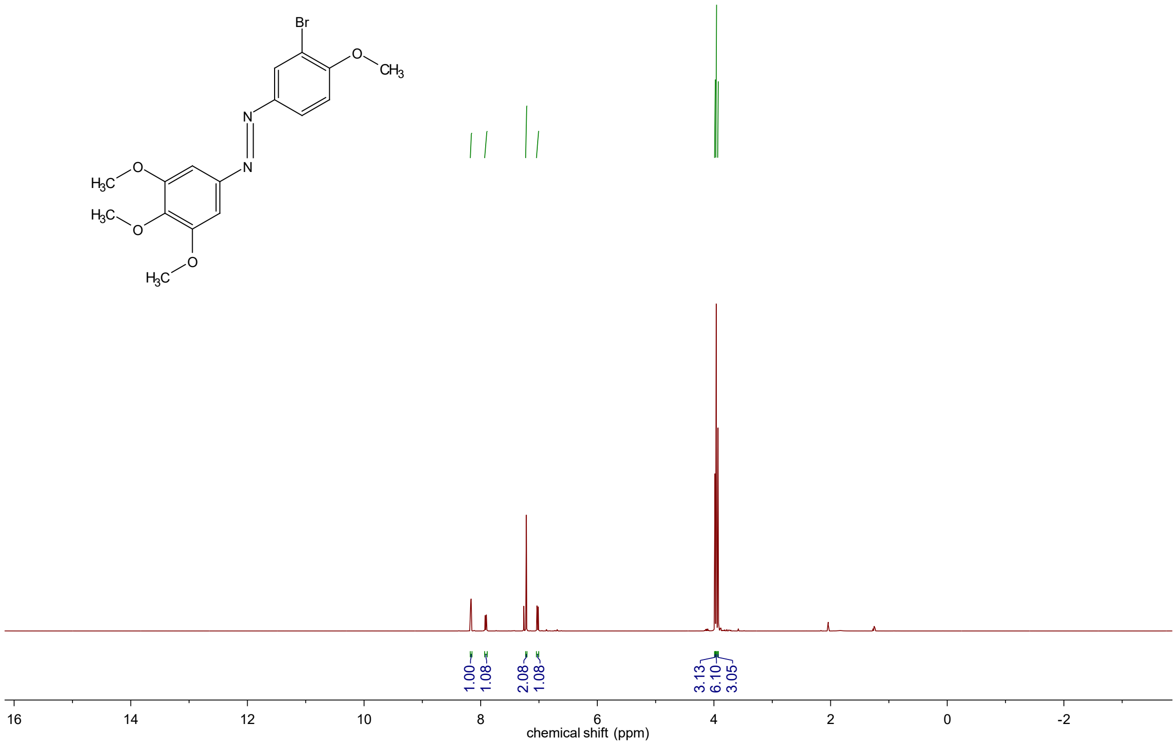

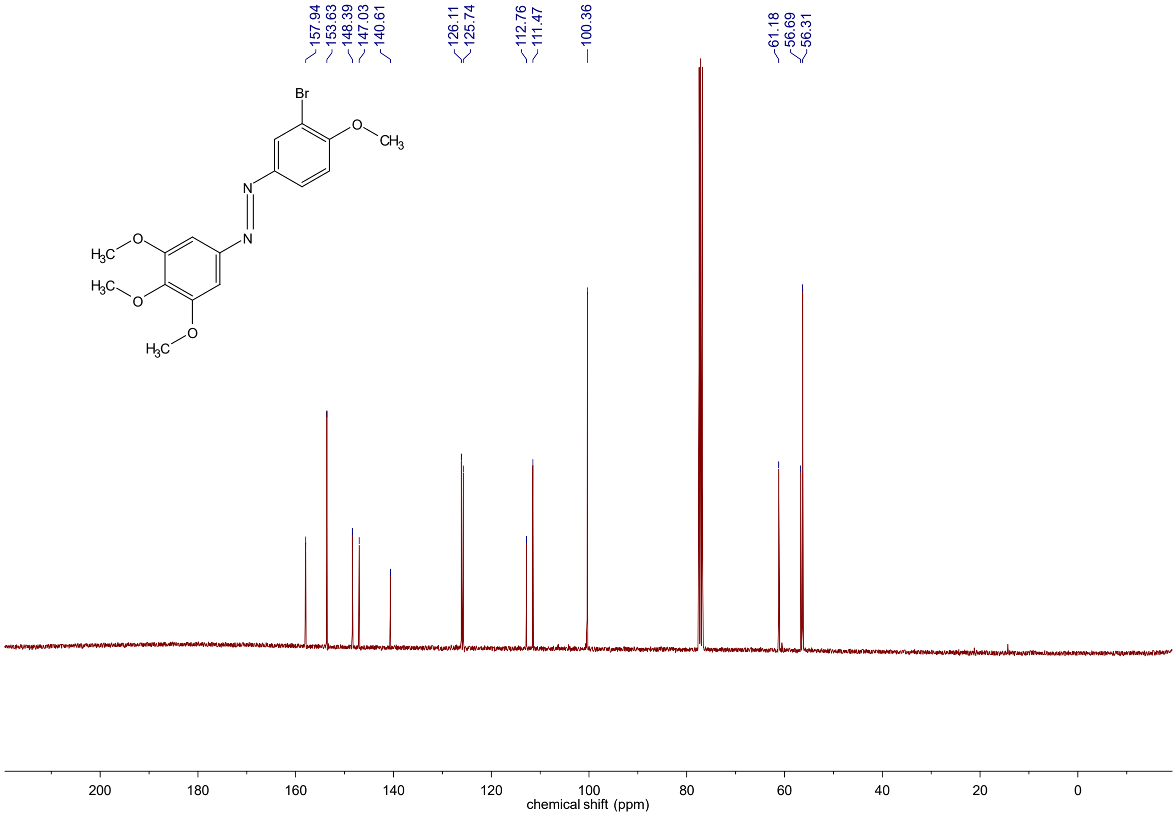

1-(3,4-dimethoxyphenyl)–2-(3,4,5-trimethoxyphenyl)diazene (**PST-9**)

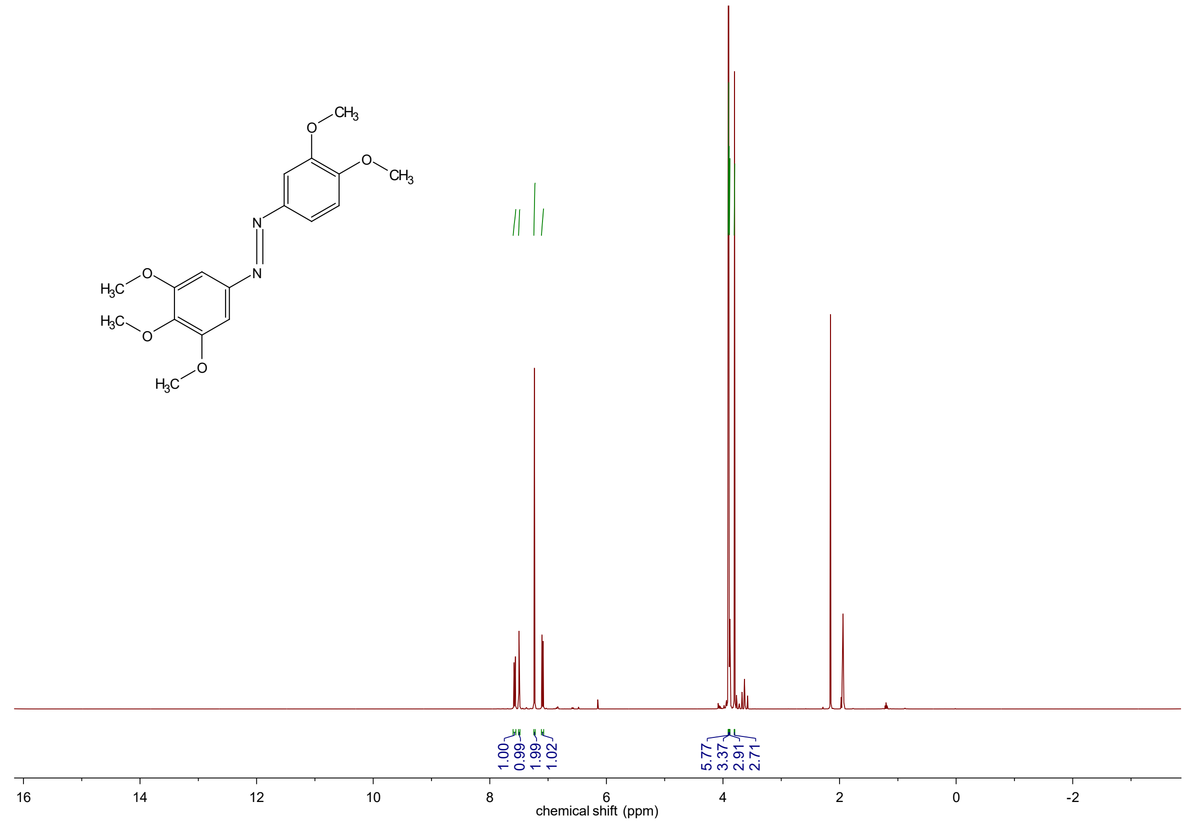

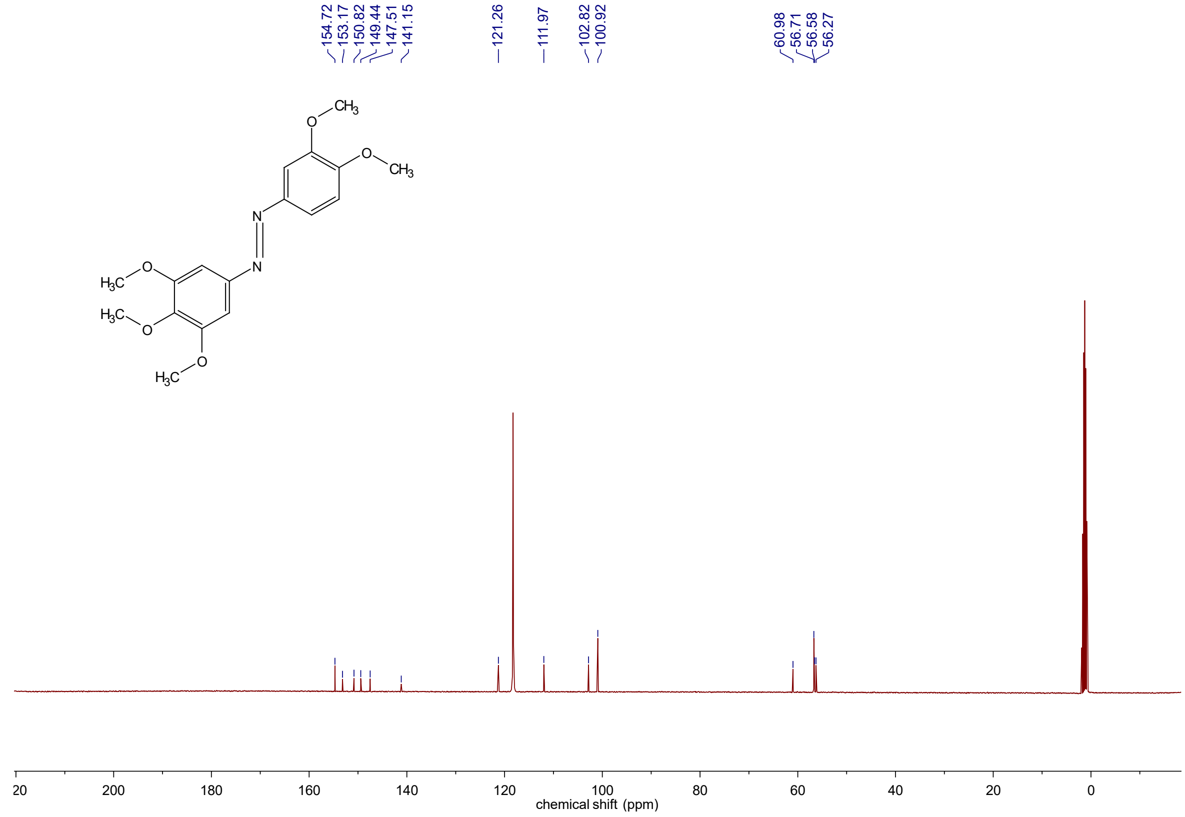

1-(benzo[d][1,3]dioxol-5-yl)-2-(3,4,5-trimethoxyphenyl)diazene (**PST-10**)

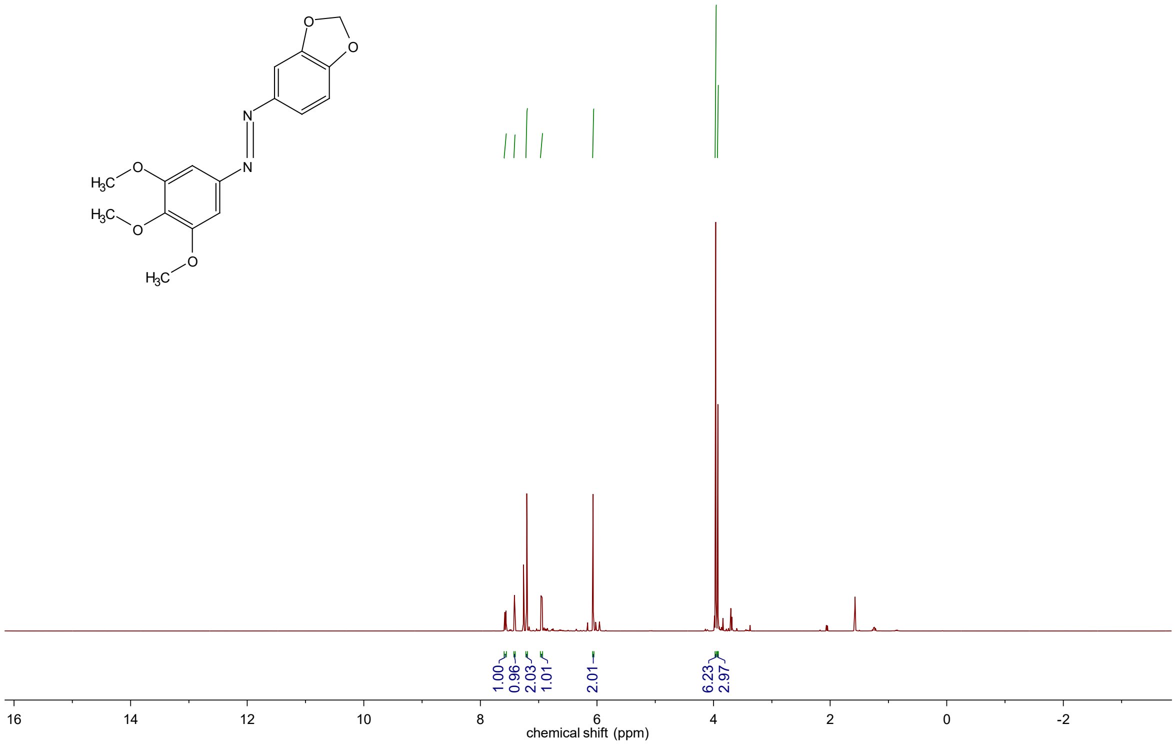

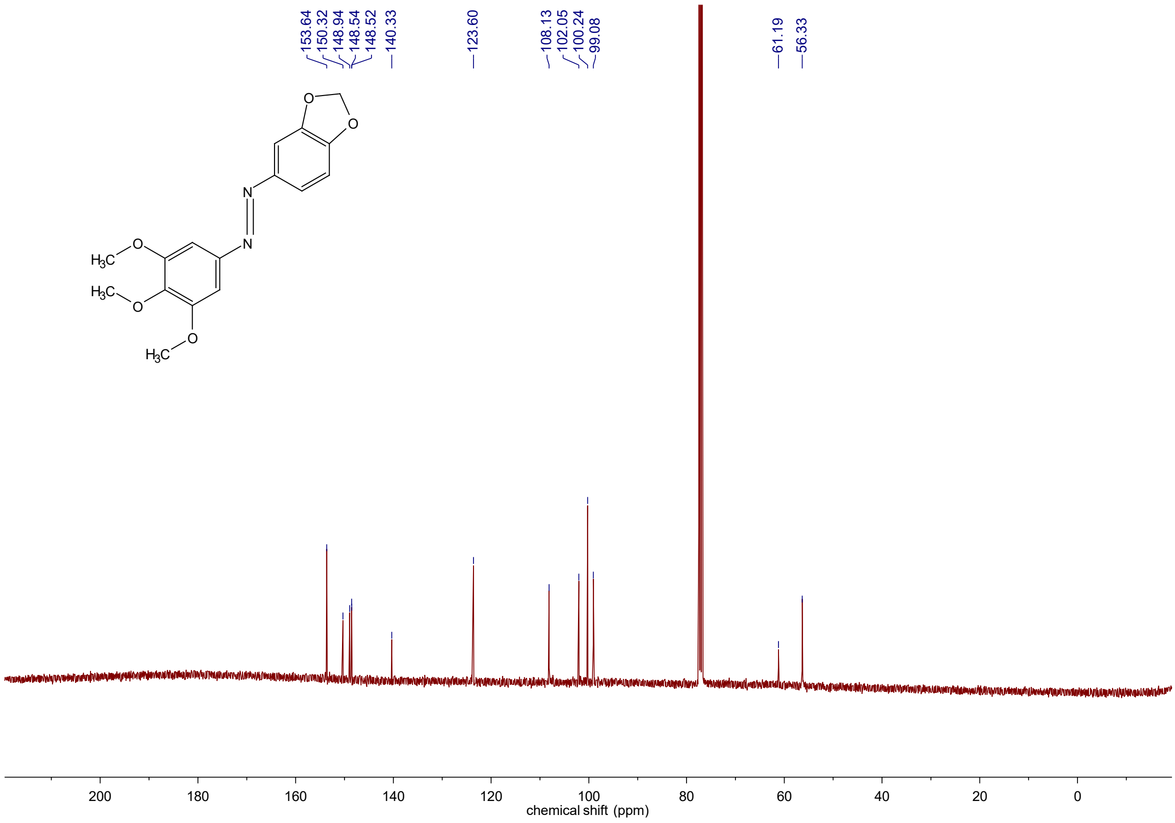

2-methoxy-5-((3,4,5-trimethoxyphenyl)diazenyl)benzaldehyde (**PST-11**)

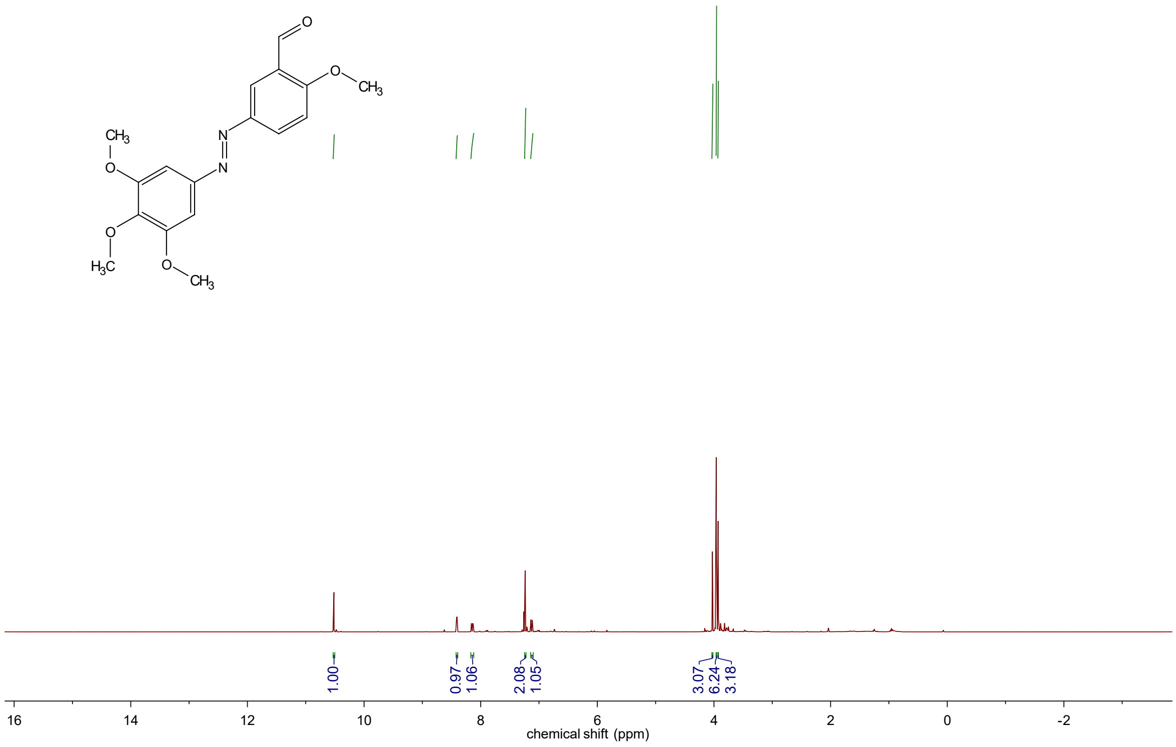

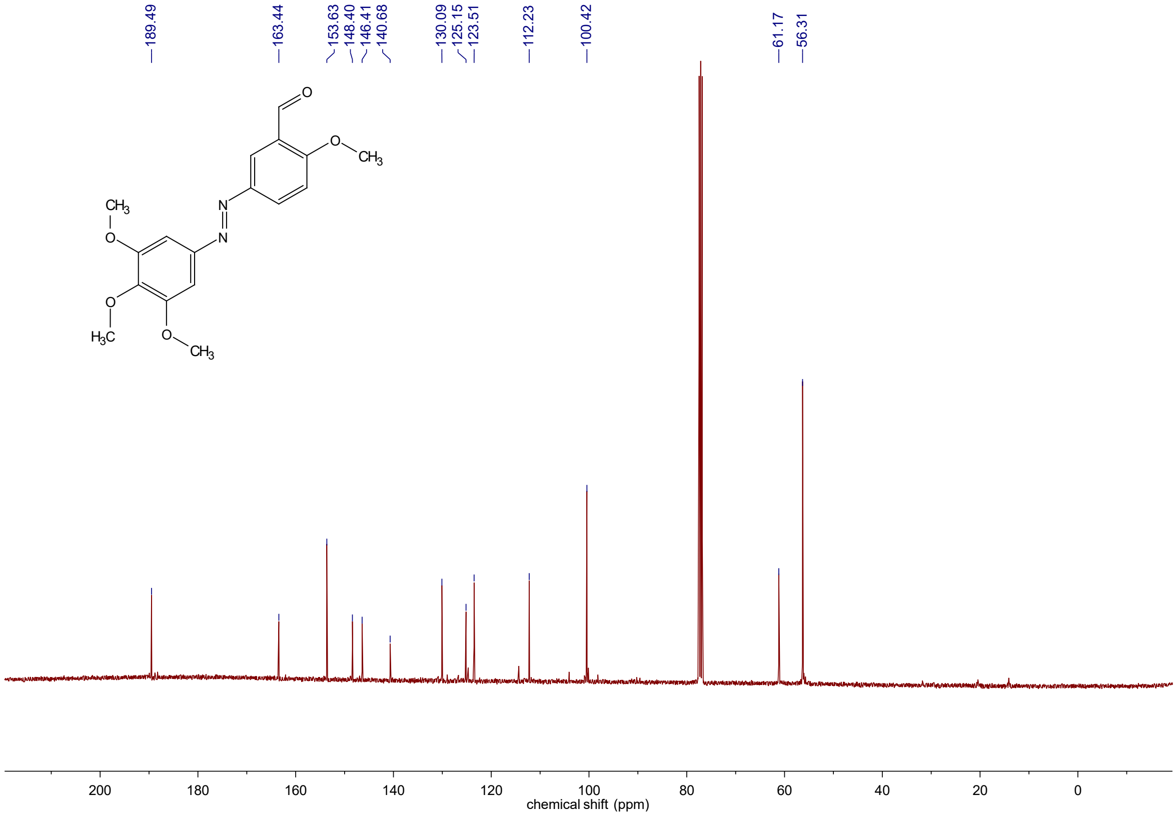

2-methoxy-5-((3,4,5-trimethoxyphenyl)diazenyl)benzoic acid (**PST-12**)

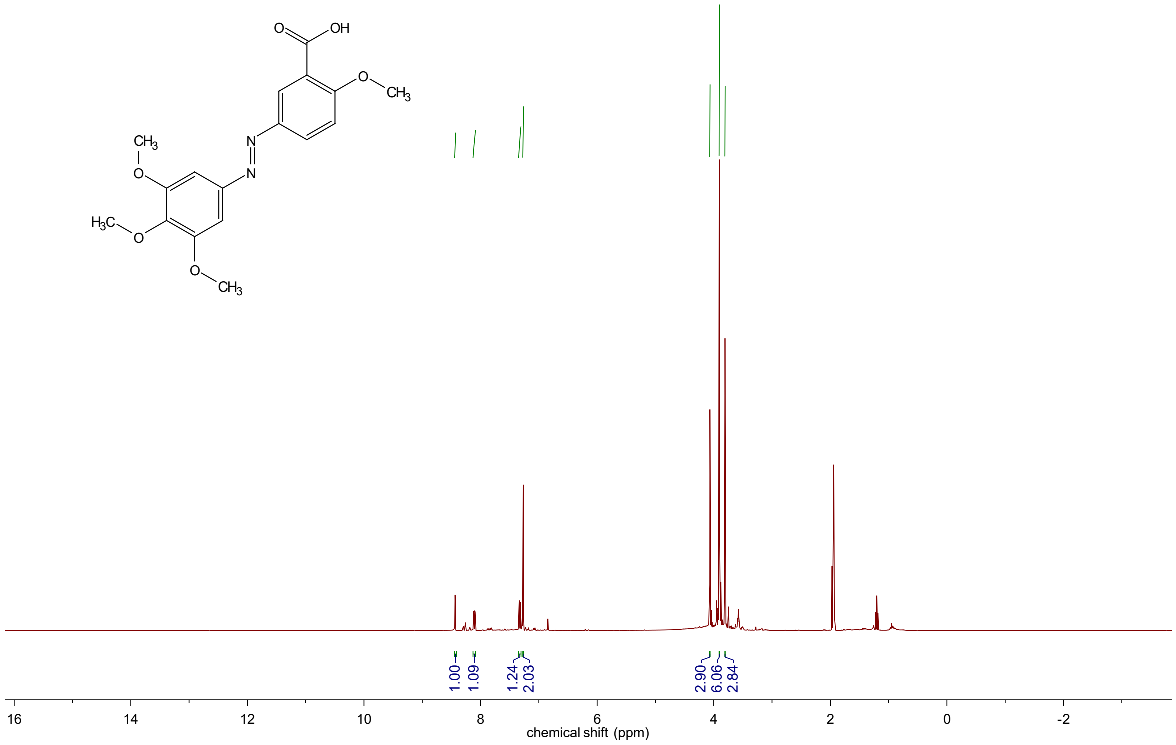

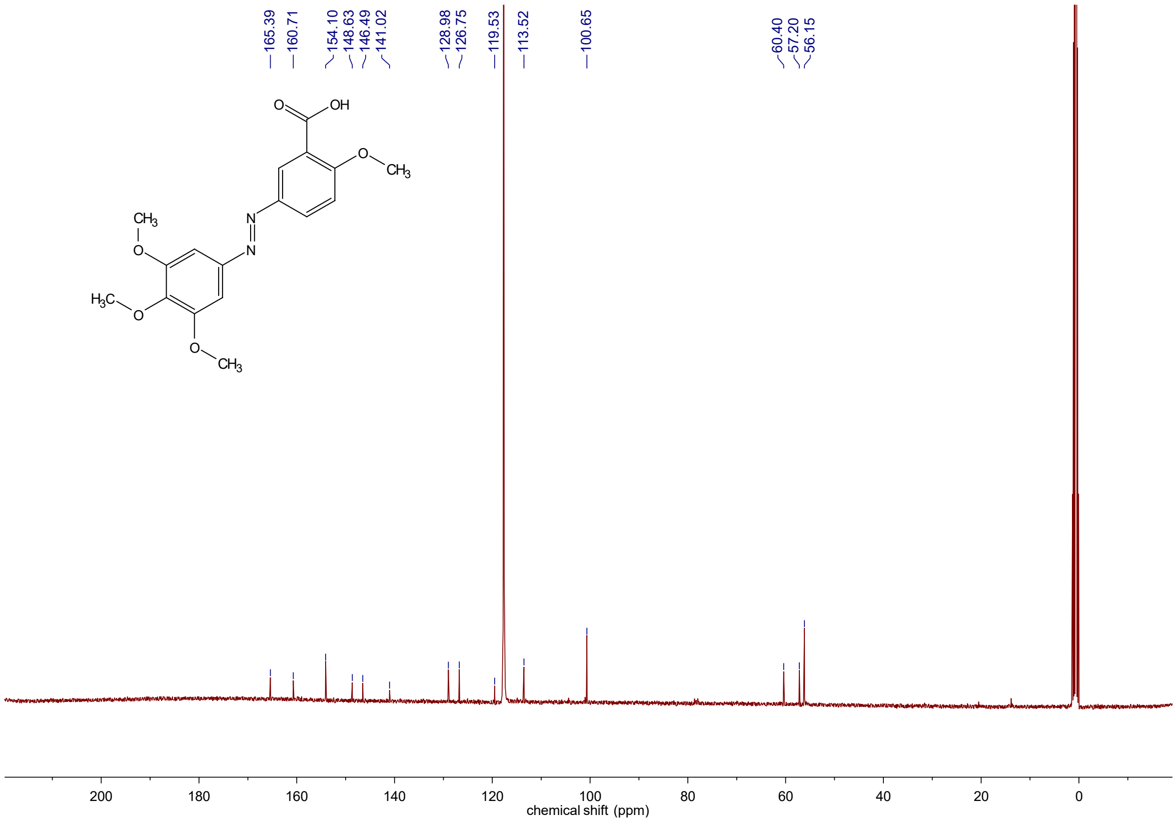

1-(4-(methylthio)phenyl)-2-(3,4,5-trimethoxyphenyl)diazene (**PST-13**)

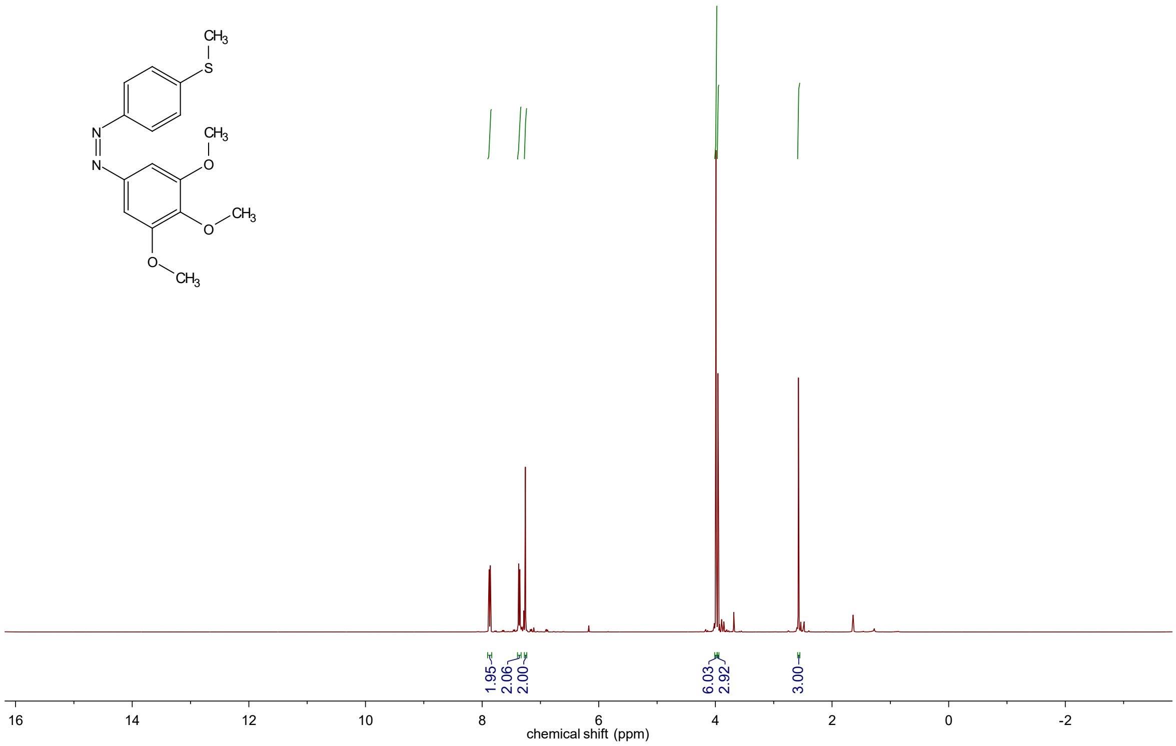

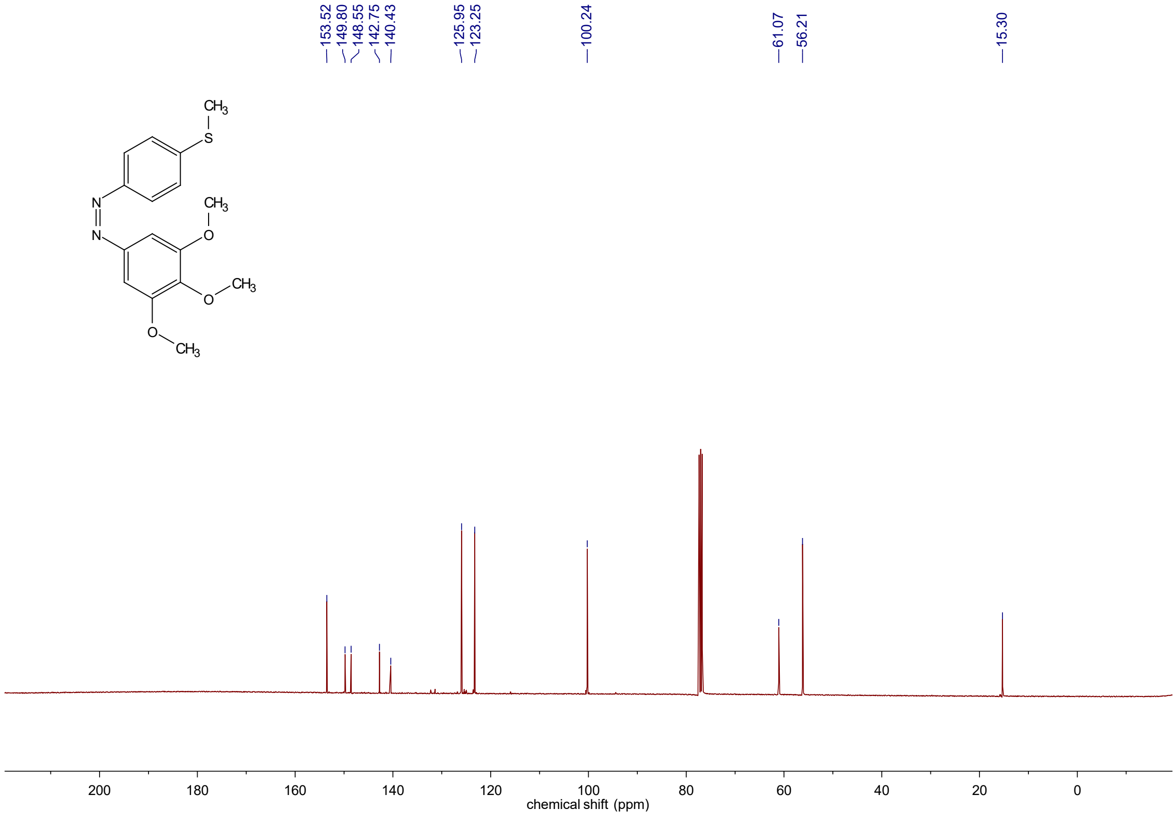

1-(4-(methylsulfinyl)phenyl)-2-(3,4,5-trimethoxyphenyl)diazene (**PST-14**)

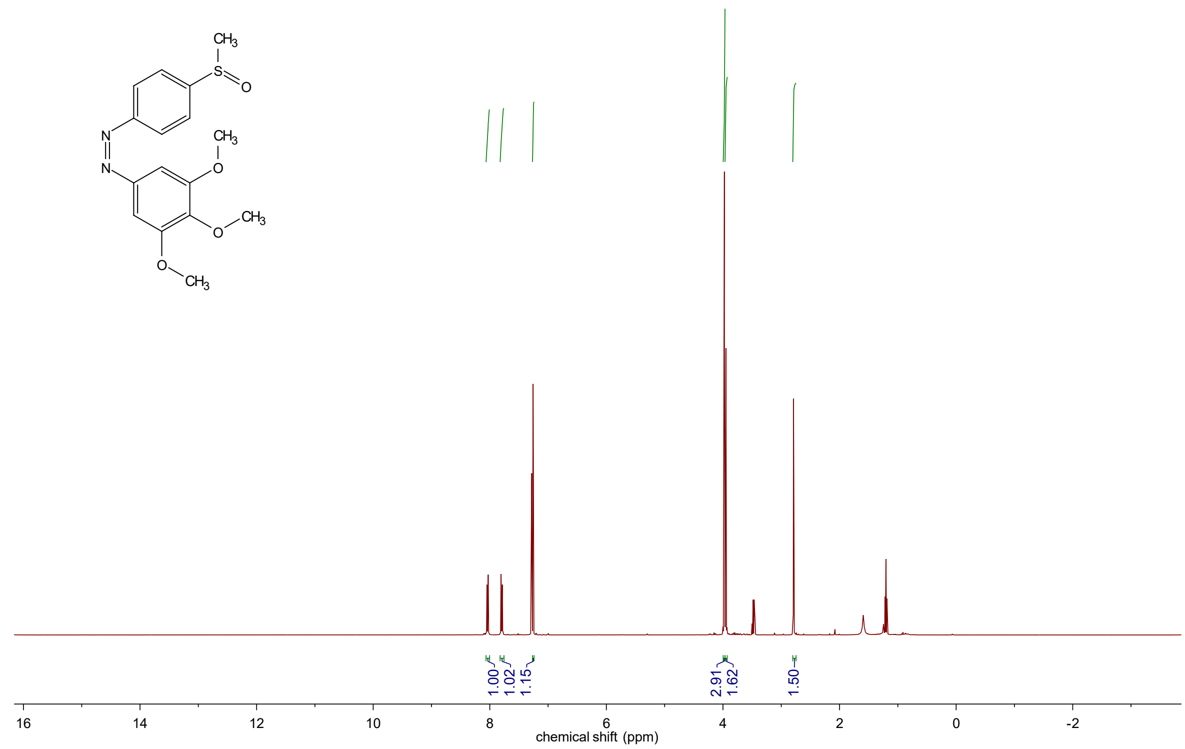

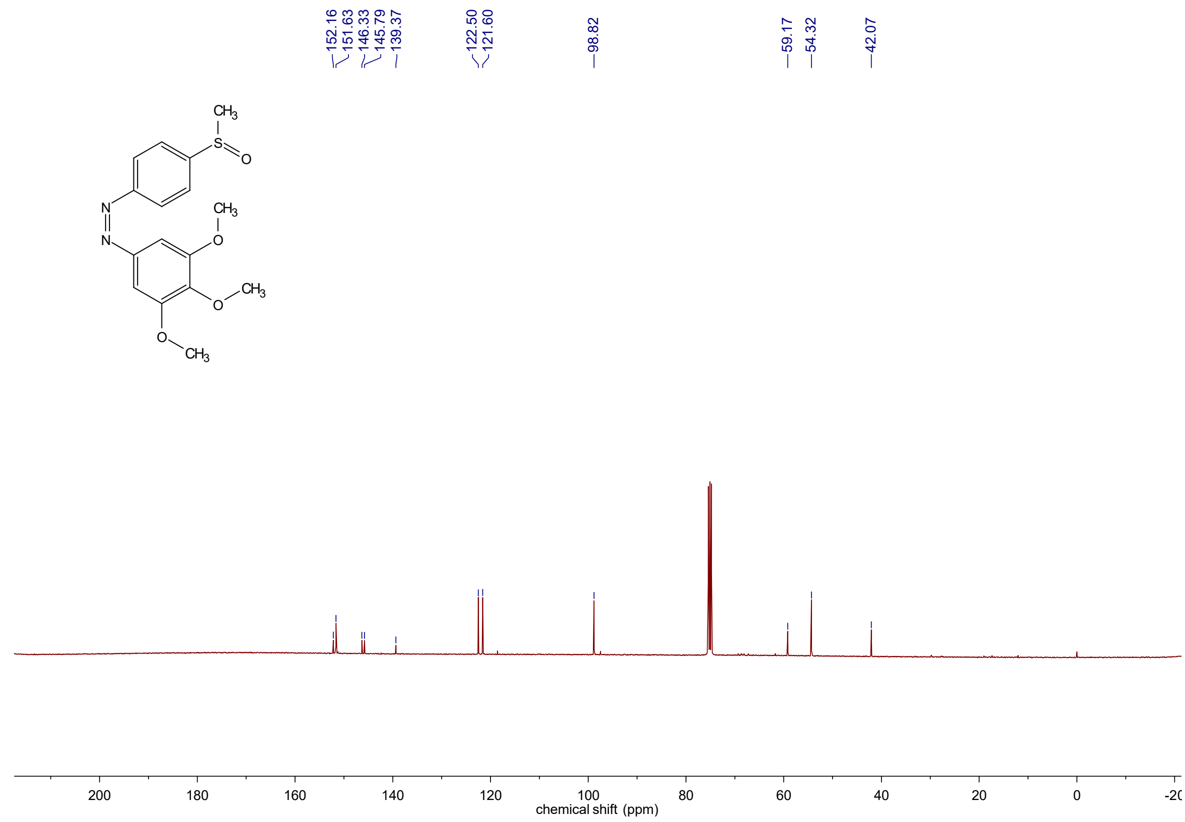

1-(4-(methylsulfonyl)phenyl)-2-(3,4,5-trimethoxyphenyl)diazene (**PST-15**)

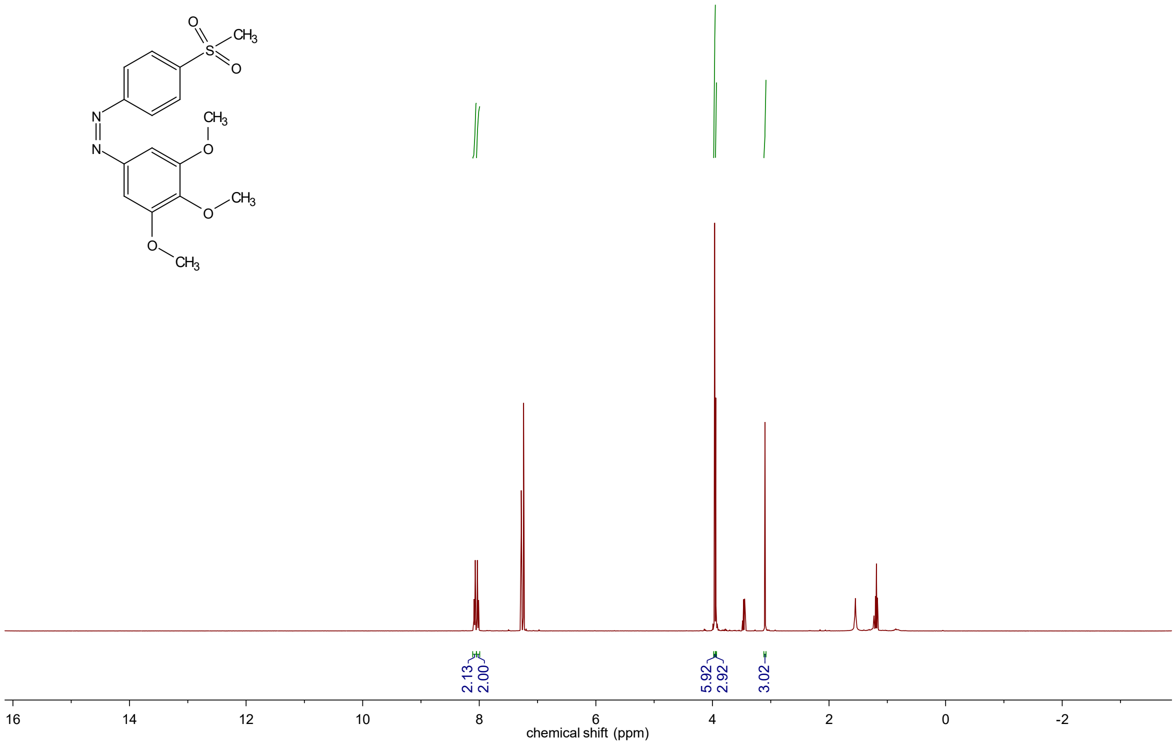

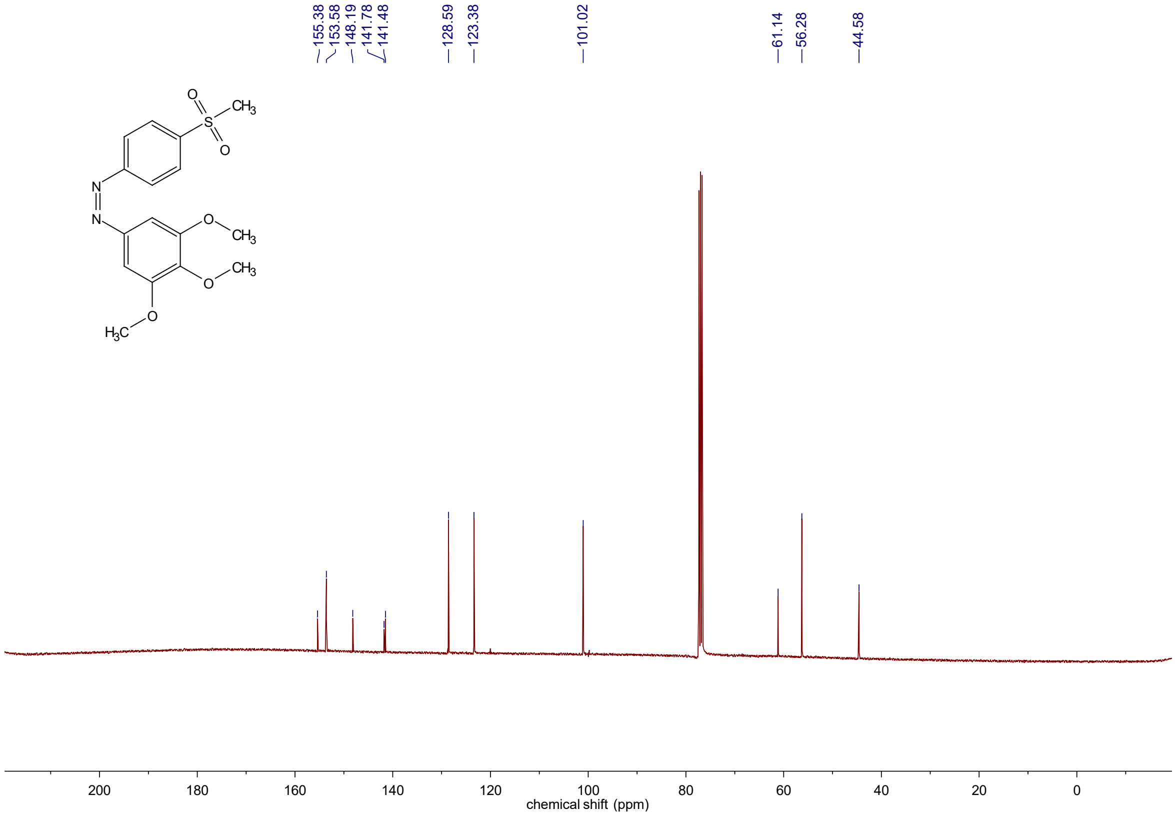

1-(2,3-difluoro-4-methoxyphenyl)-2-(3,4,5-trimethoxyphenyl)diazene (**PST-16**)

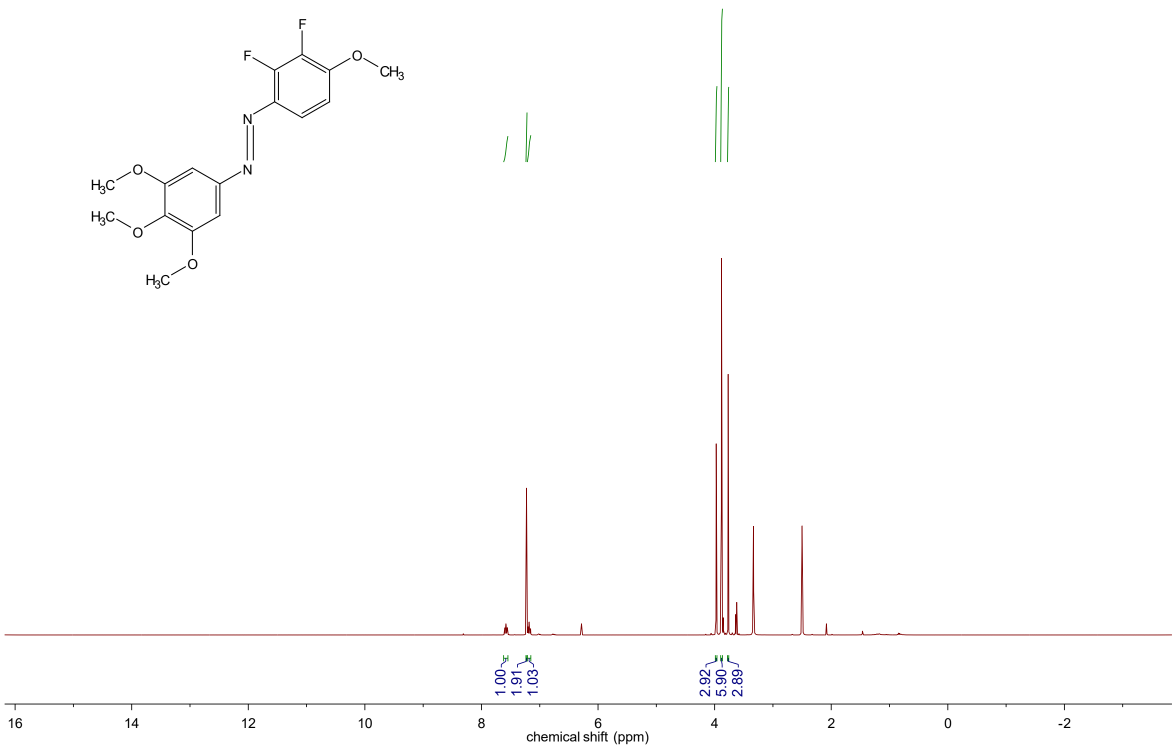

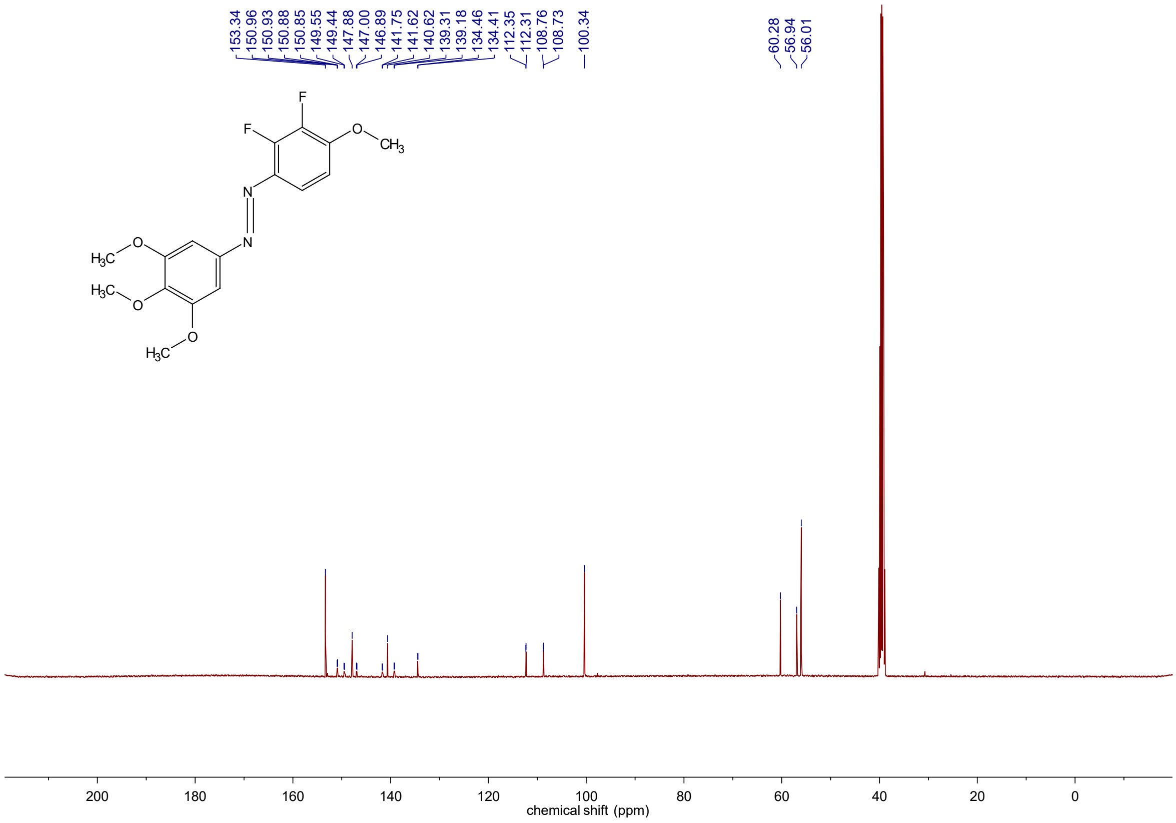

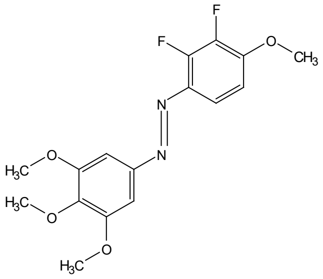

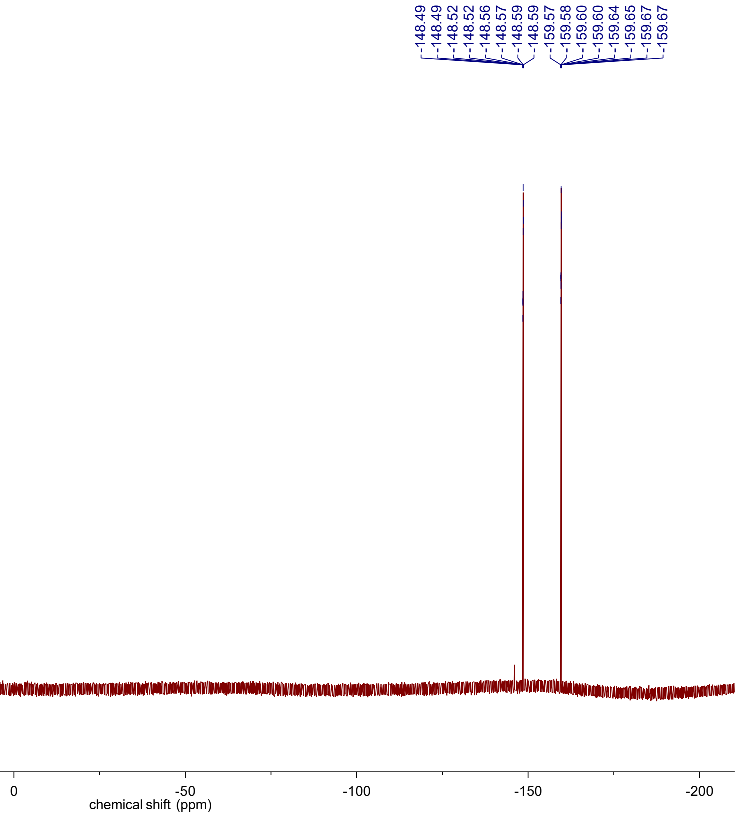
-148.49
-148.49
-148.52
-148.52
-148.56
-148.57
-148.59
-148.59
-159.57
-159.58
-159.60
-159.60
-159.64
-159.65
-159.67
-159.67
100
50
0
-50
-100
-150
-200
chemical shift (ppm)

1-(2-fluoro-4-methoxy-3-nitrophenyl)-2-(3,4,5-trimethoxyphenyl)diazene (**PST-17**)

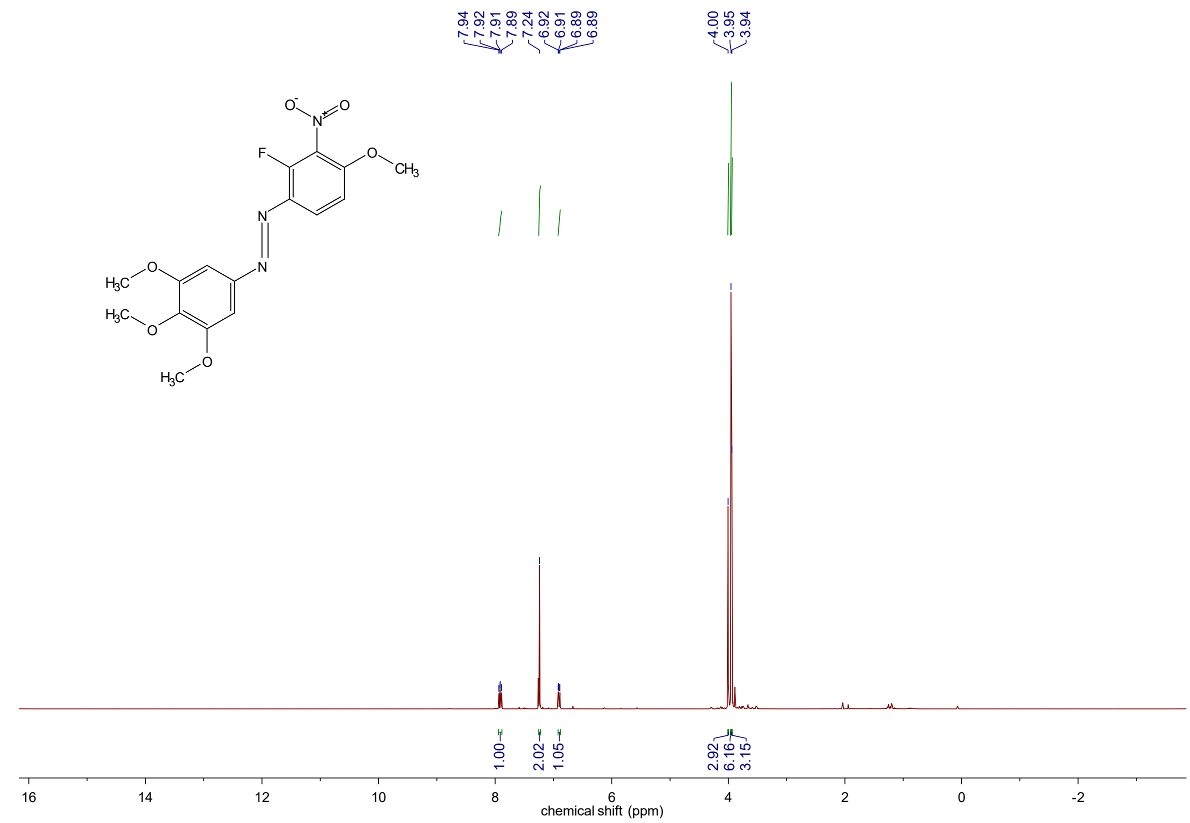

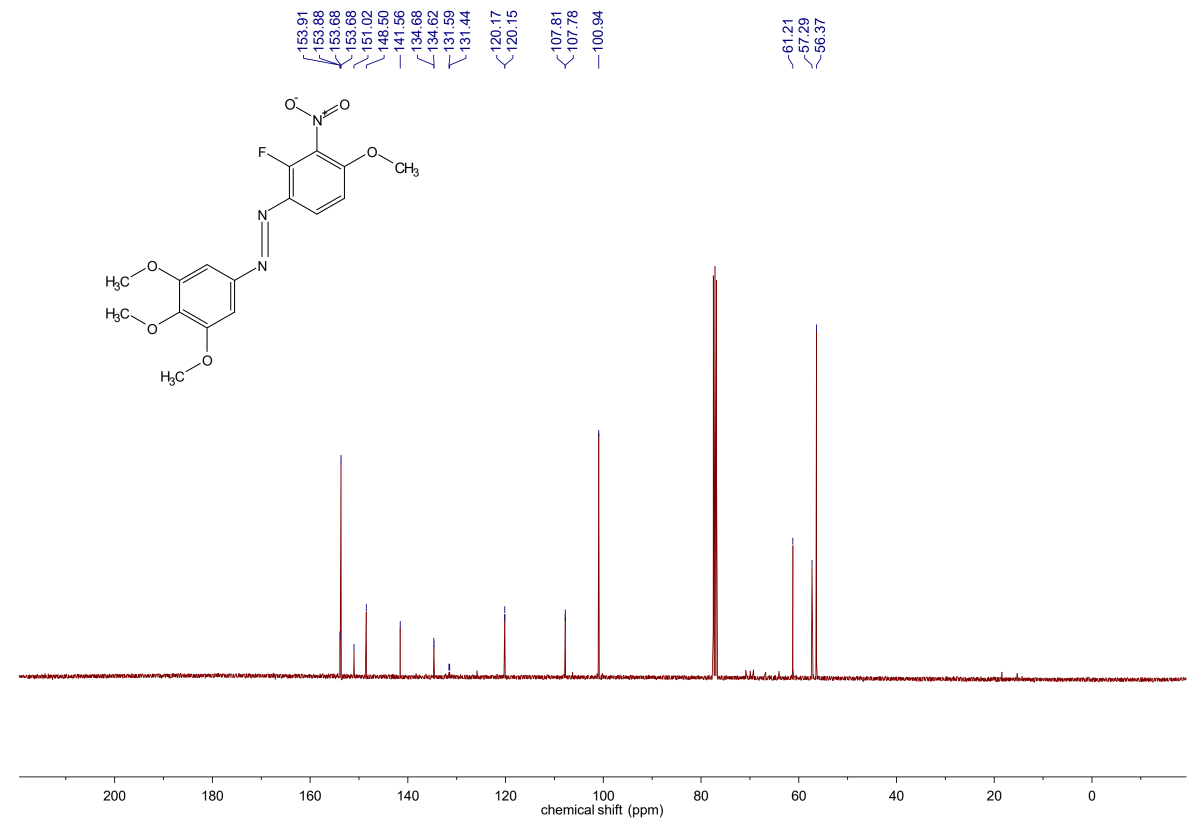

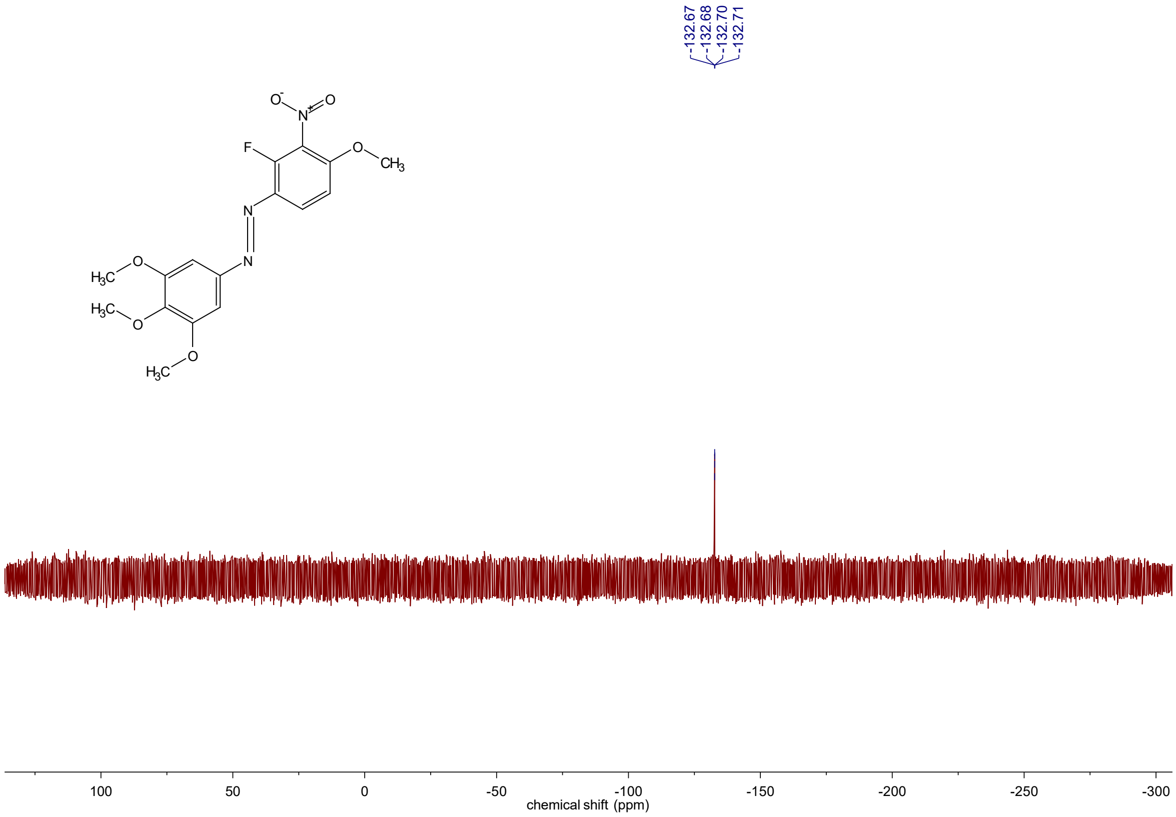

-132.67
-132.68
-132.70
-132.71
O-
N+
O
F
O
CH3
N
N
H3C
O
H3C
O
O
H3C
100
50
0
-50
-100
-150
-200
-250
-300
chemical shift (ppm)

2-methoxy-5-((3,4,5-trimethoxyphenyl)diazenyl)cyclohepta-2,4,6-trien-1-one (**PST-18**)

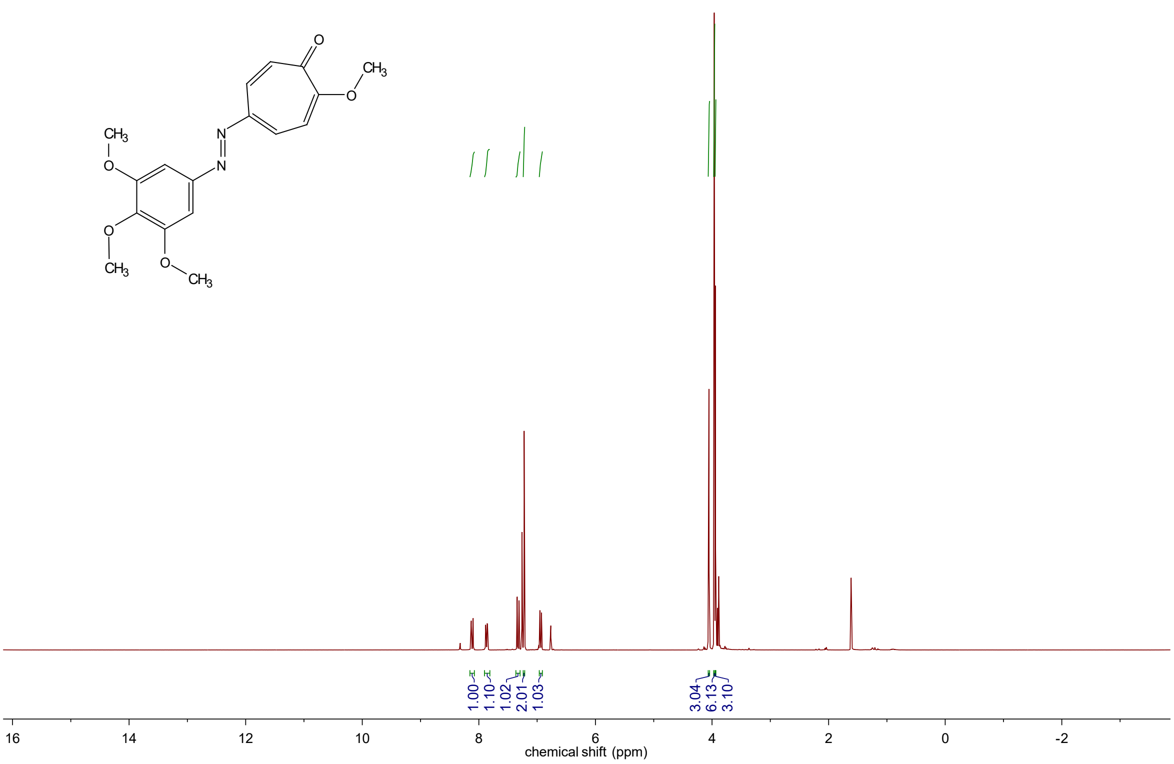

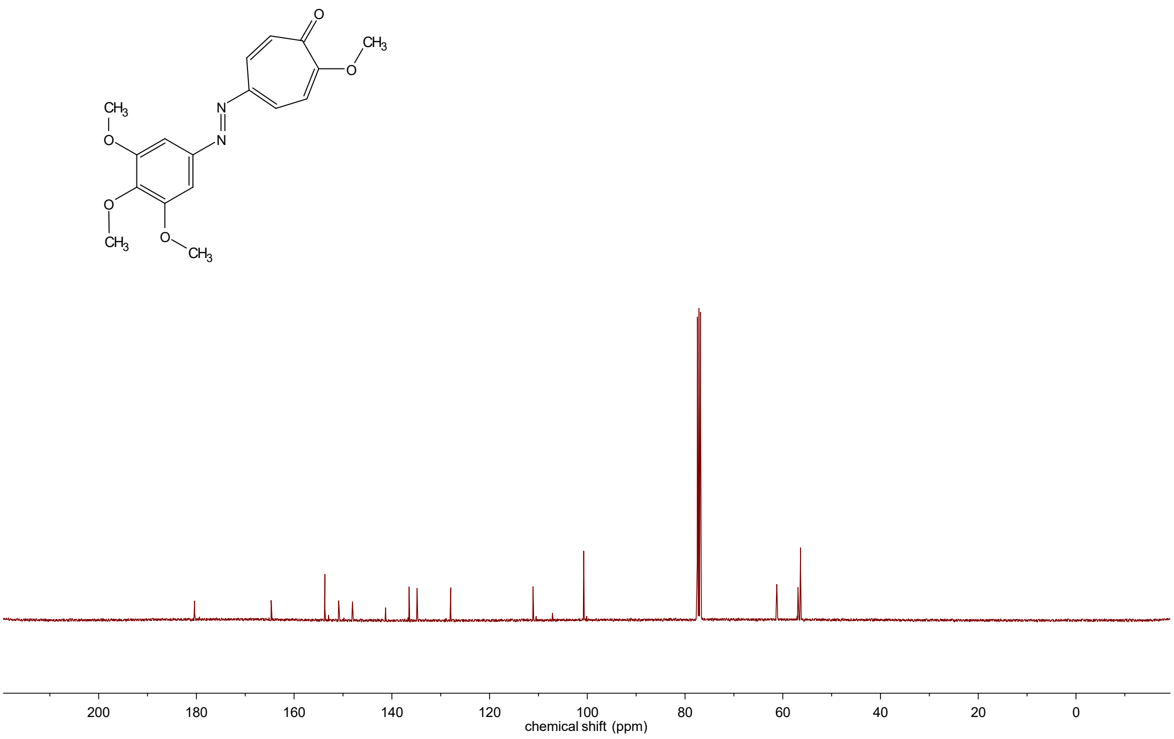

8-methoxy-5-((3,4,5-trimethoxyphenyl)diazenyl)quinoline (**PST-20**)

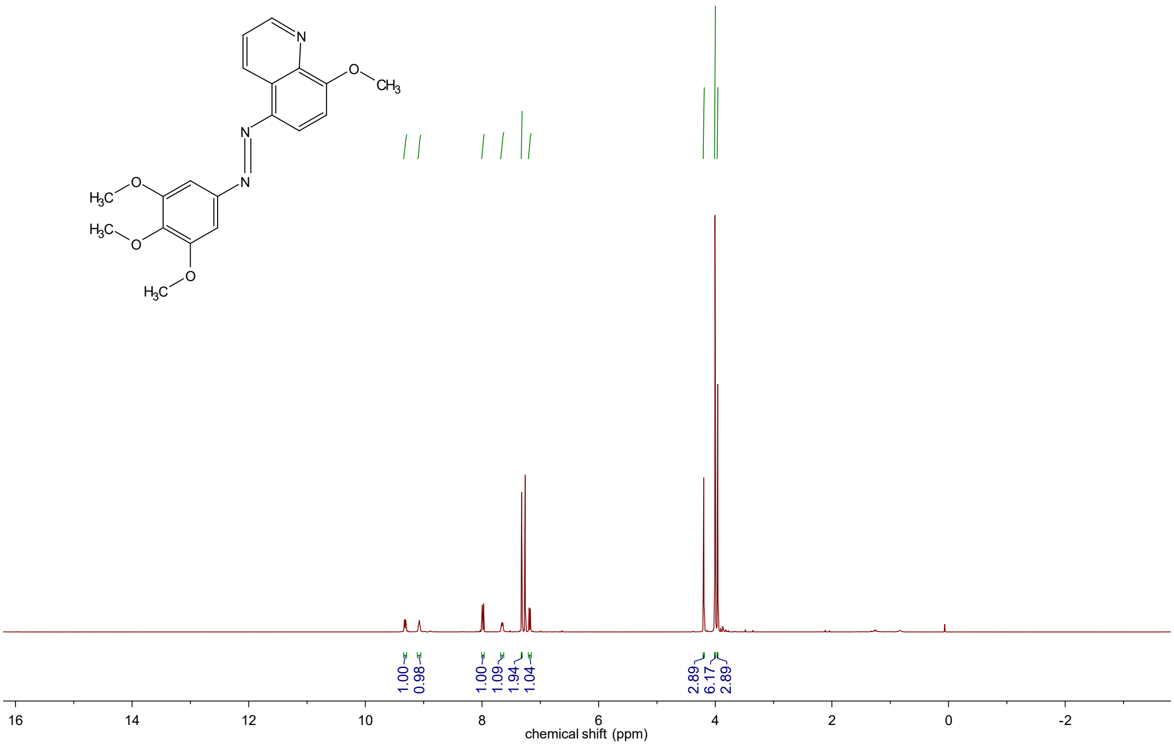

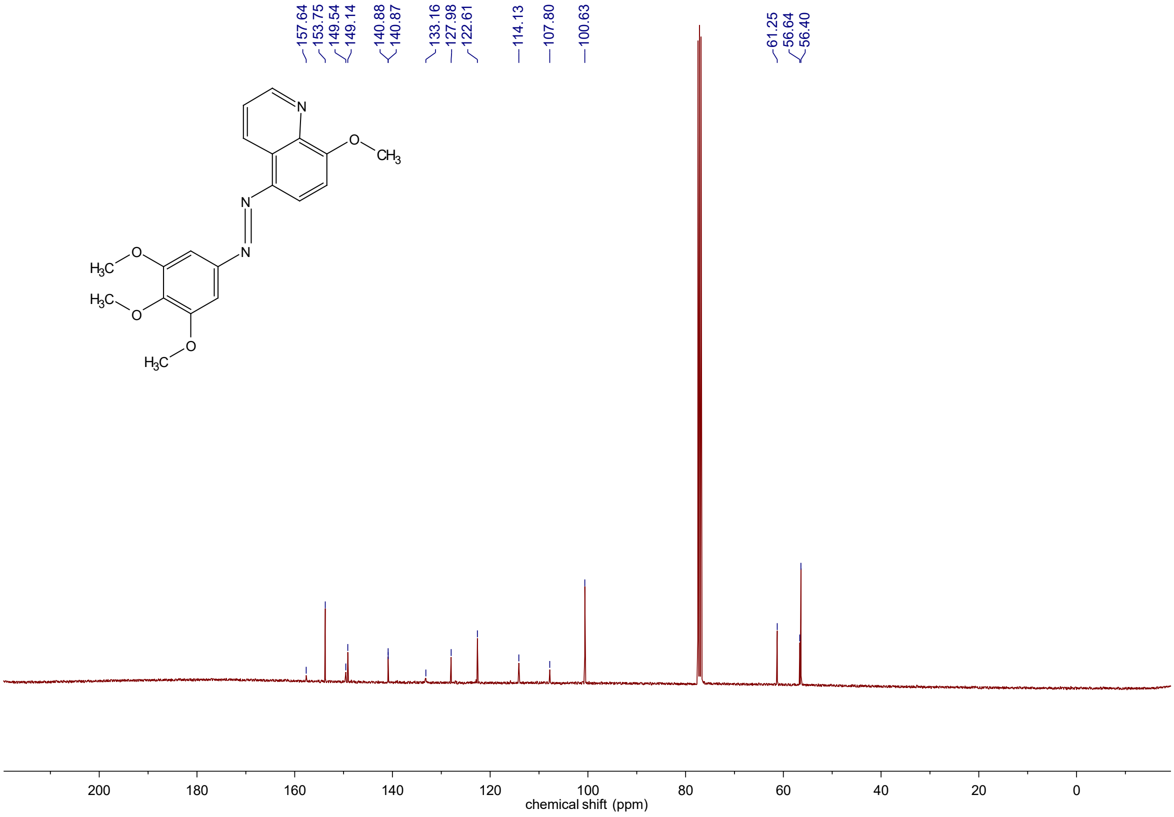

methyl 8-methoxy-5-((3,4,5-trimethoxyphenyl)diazenyl)quinoline-2-carboxylate (**PST-21**)

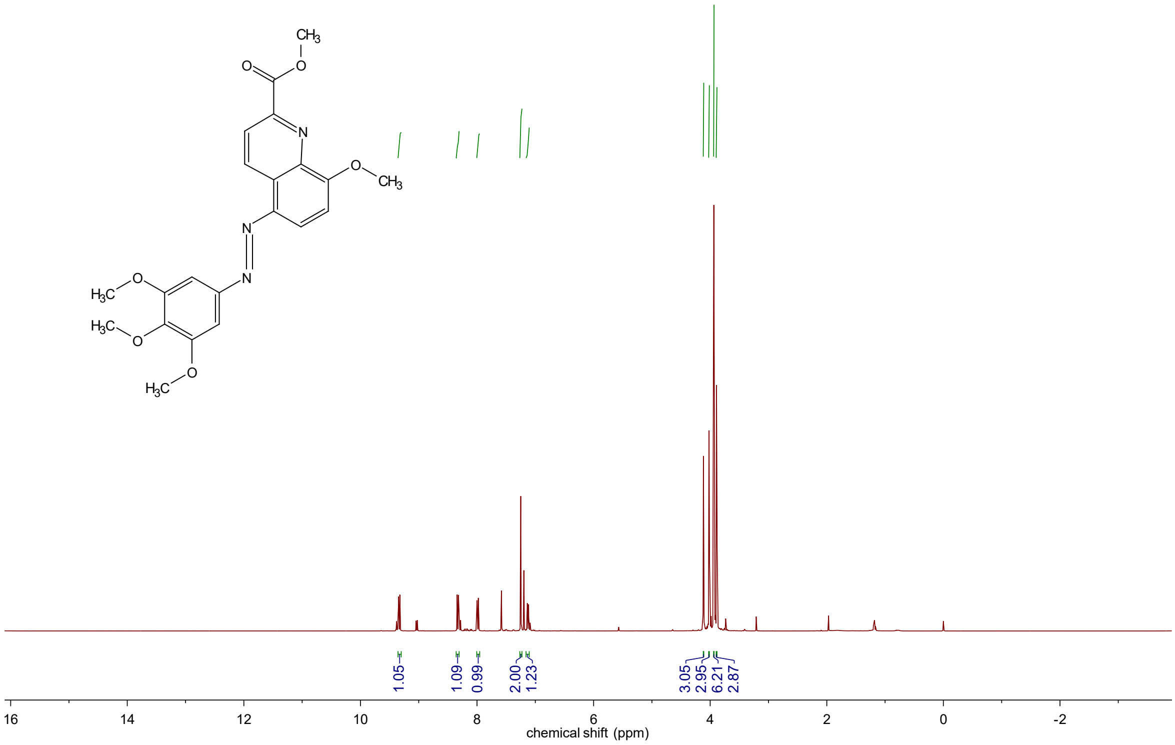

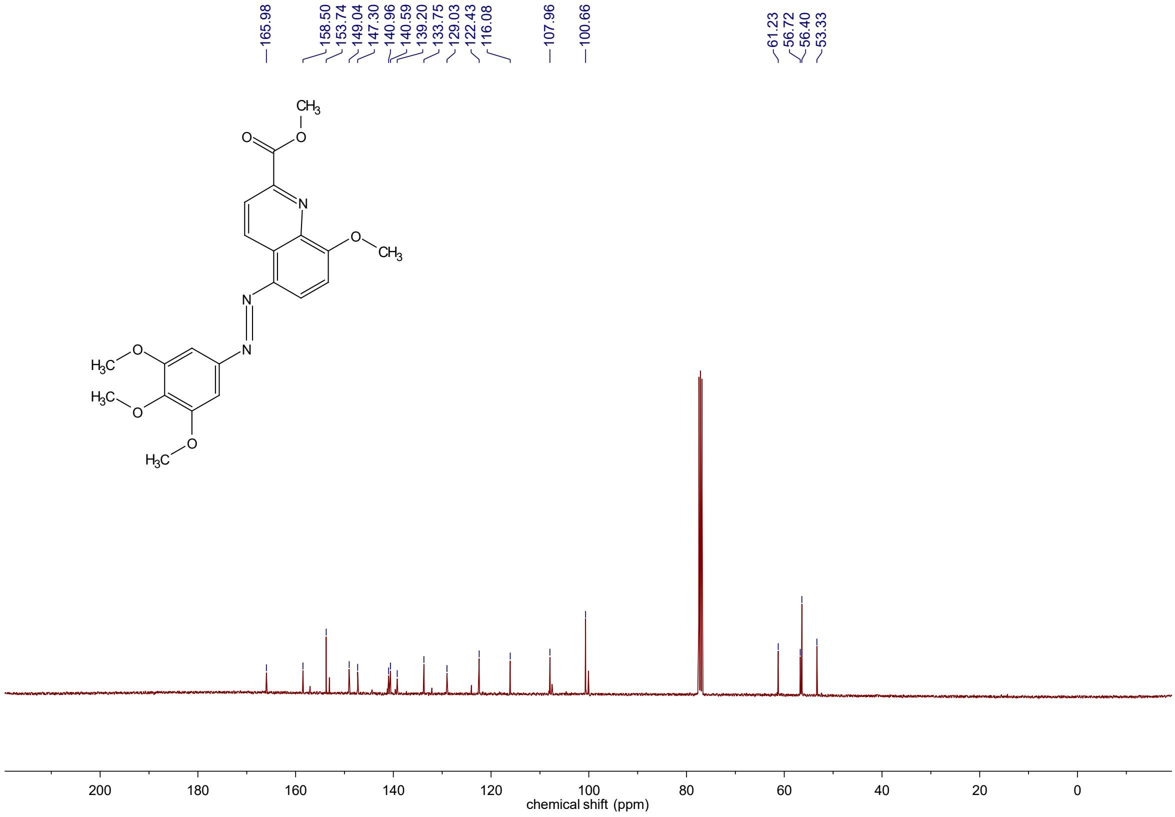

1-(2-bromo-3,4,5-trimethoxyphenyl)-2-(4-methoxyphenyl)diazene (**PST-22**)

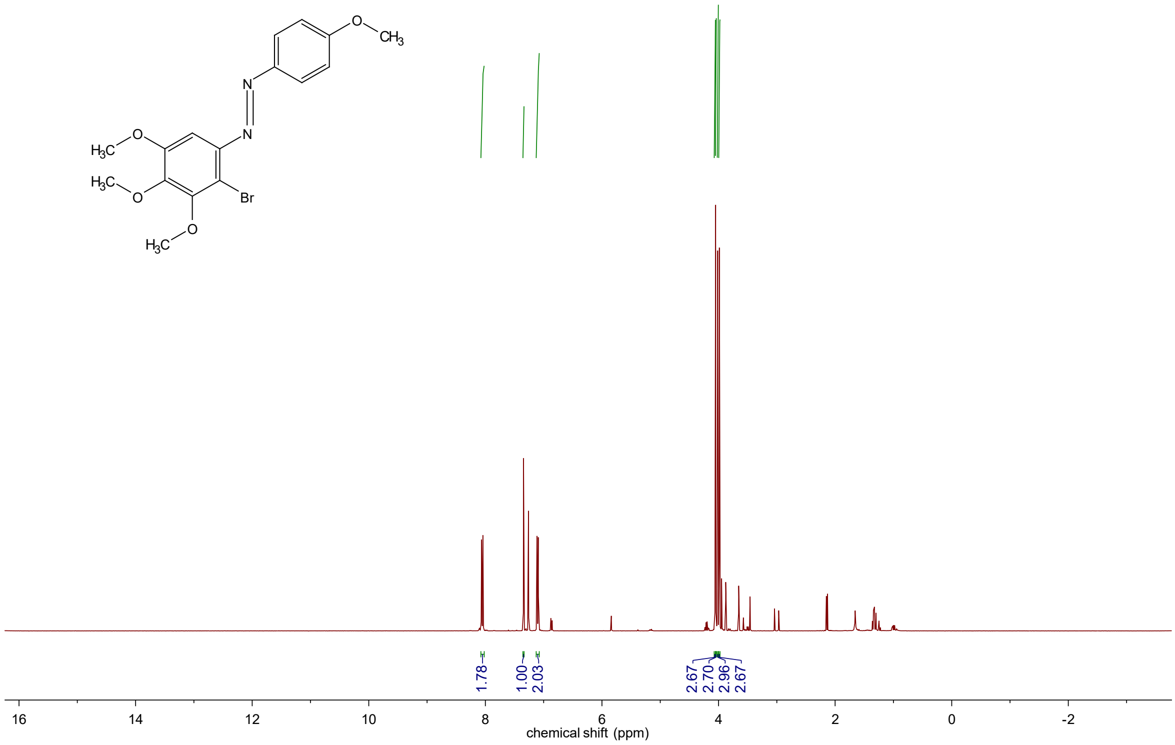

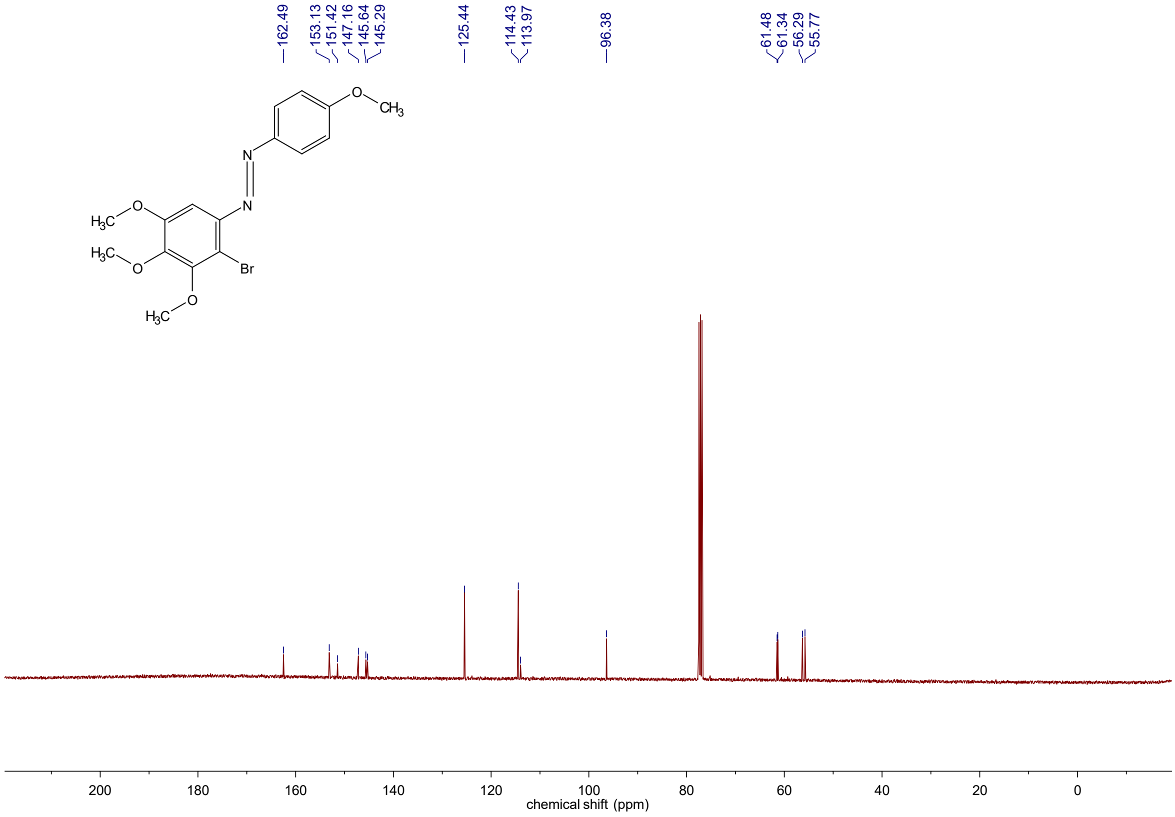

1-(4-methoxyphenyl)-2-(2,3,4,5-tetramethoxyphenyl)diazene (**PST-23)**

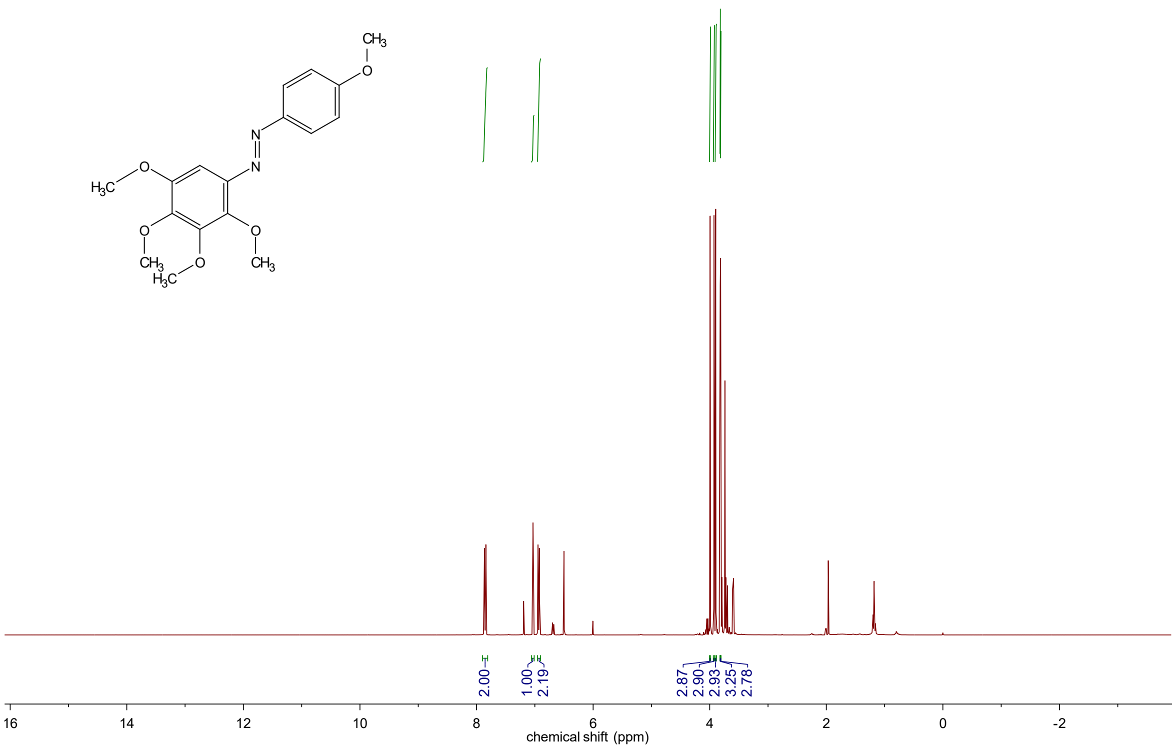

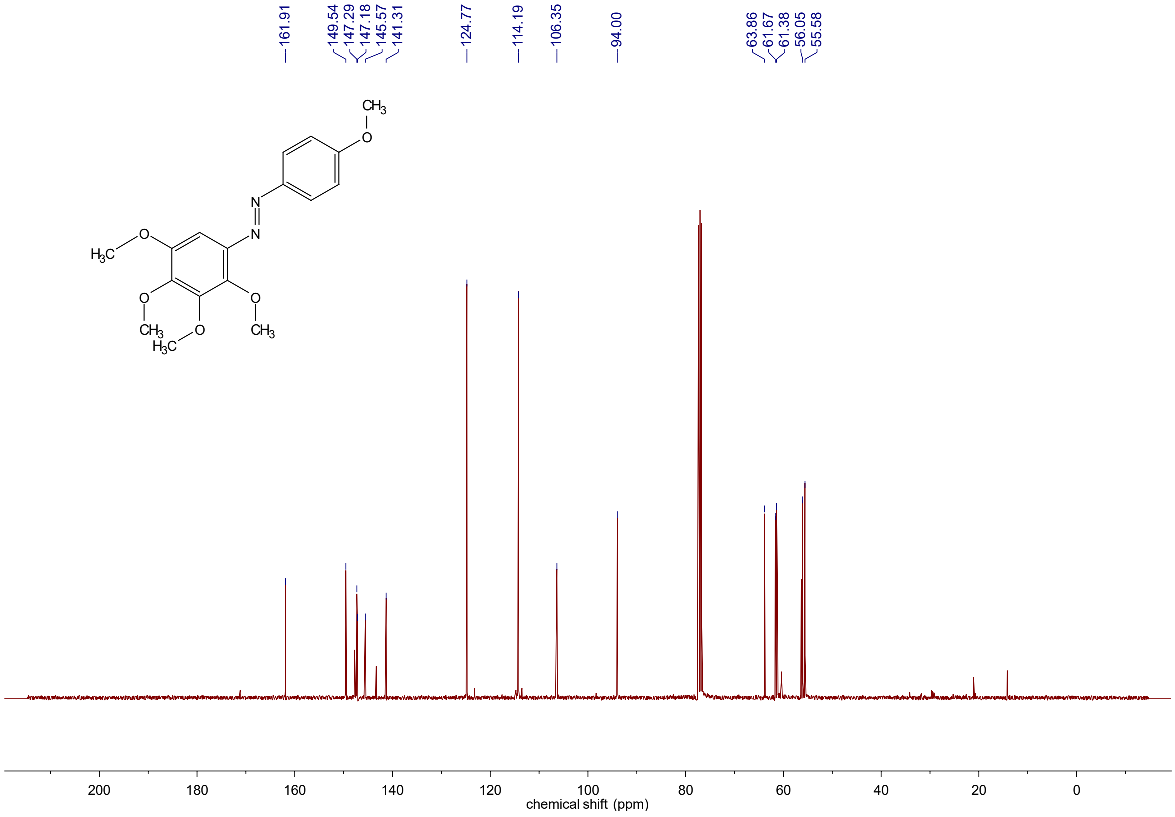

(2,3-dimethoxy-5-((4-methoxyphenyl)diazenyl)phenyl)methanol (**PST-24**)

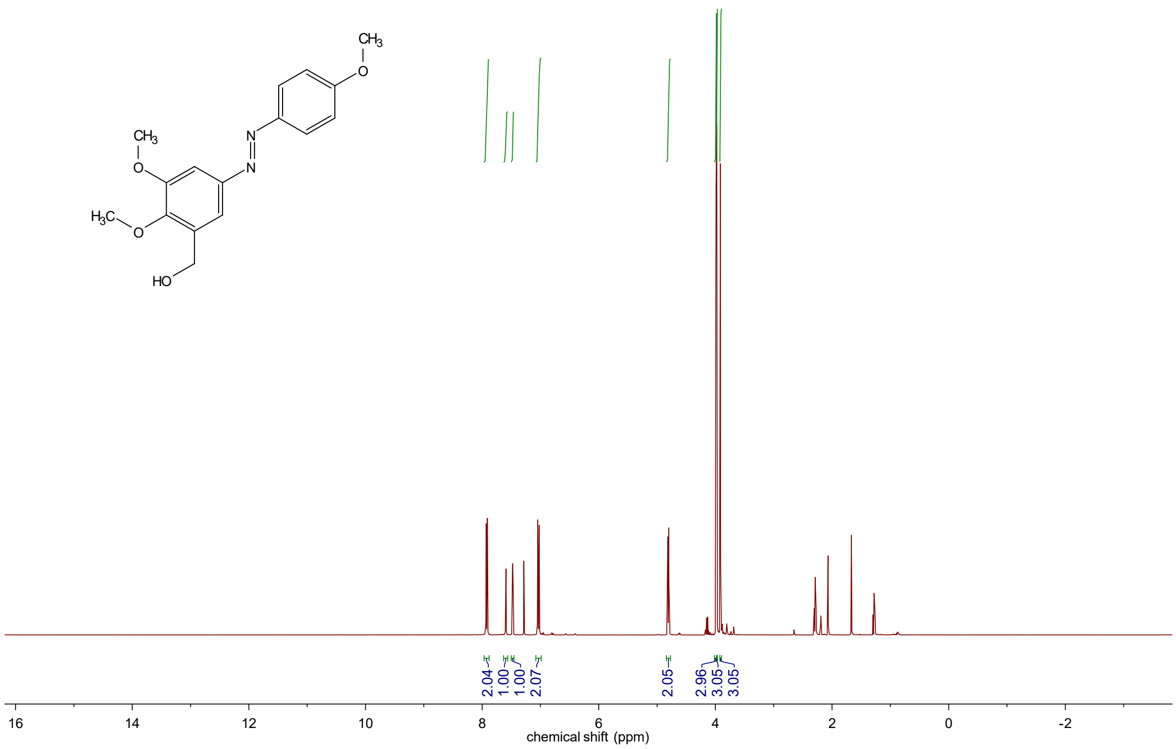

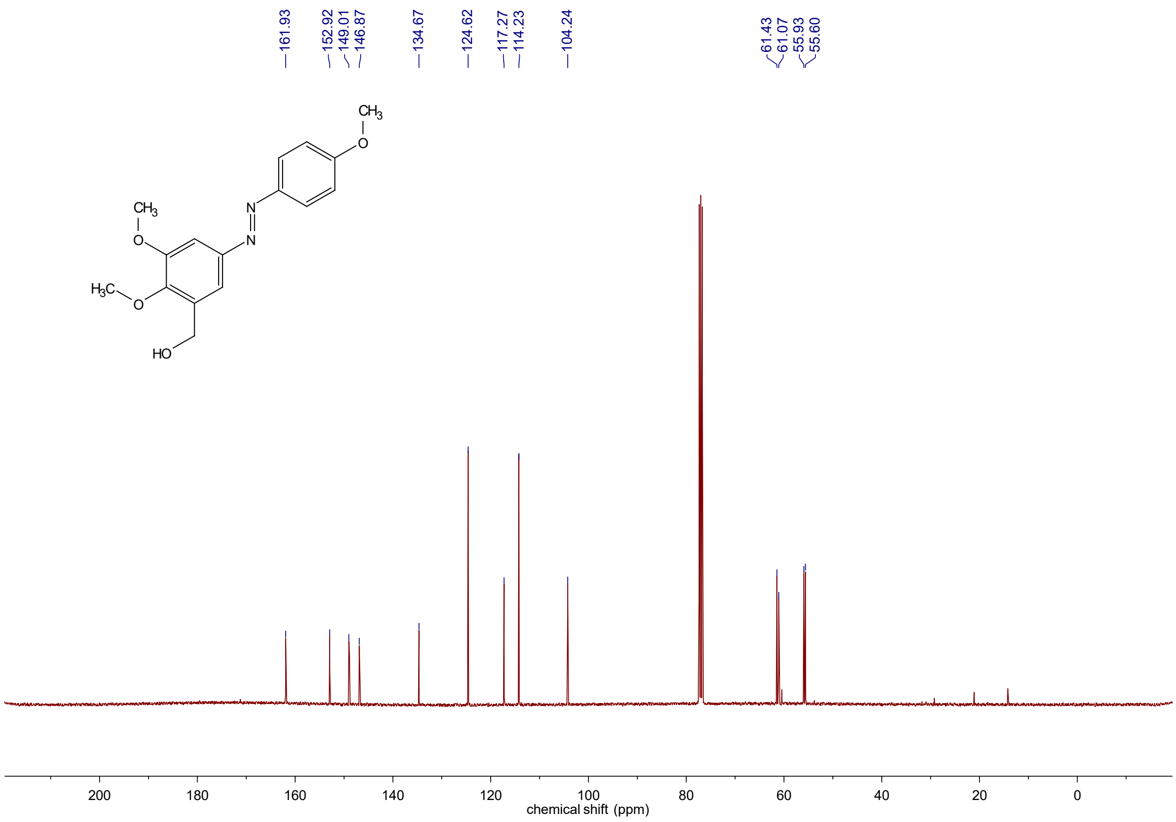

1-(2,3-dimethoxy-5-((4-methoxyphenyl)diazenyl)phenyl)-N,N-dimethylmethanammonium 2,2,2-trifluoroacetate (**PST-25**)

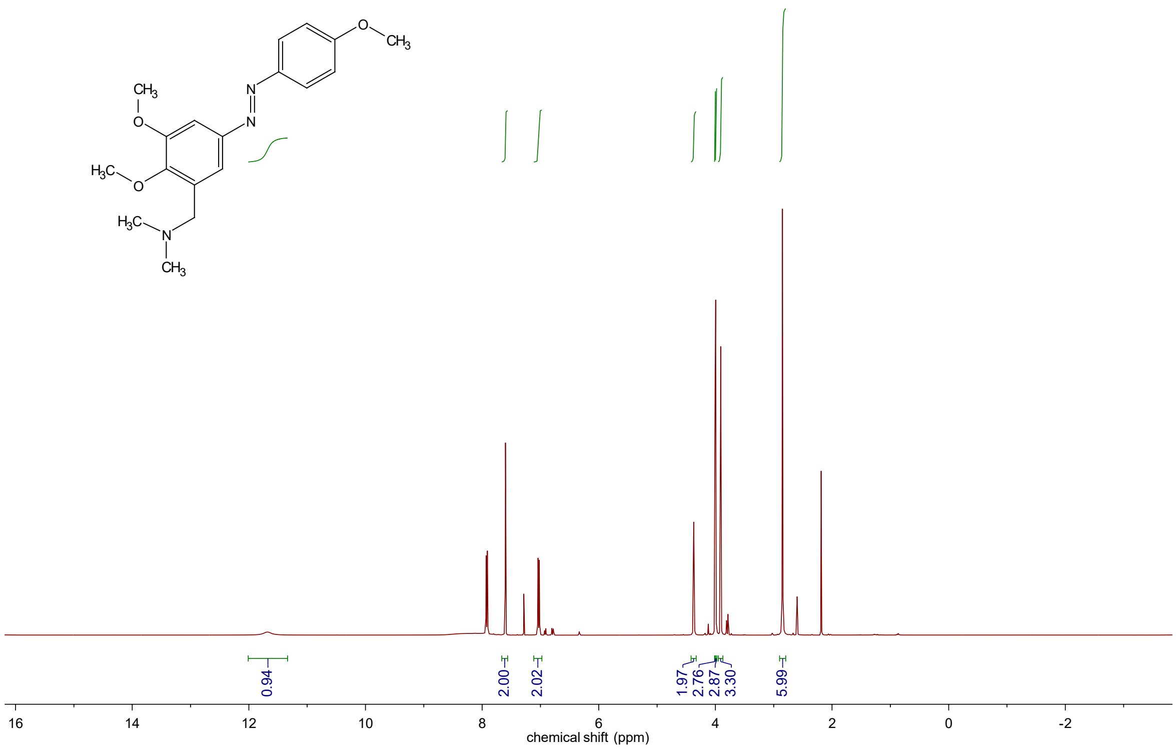

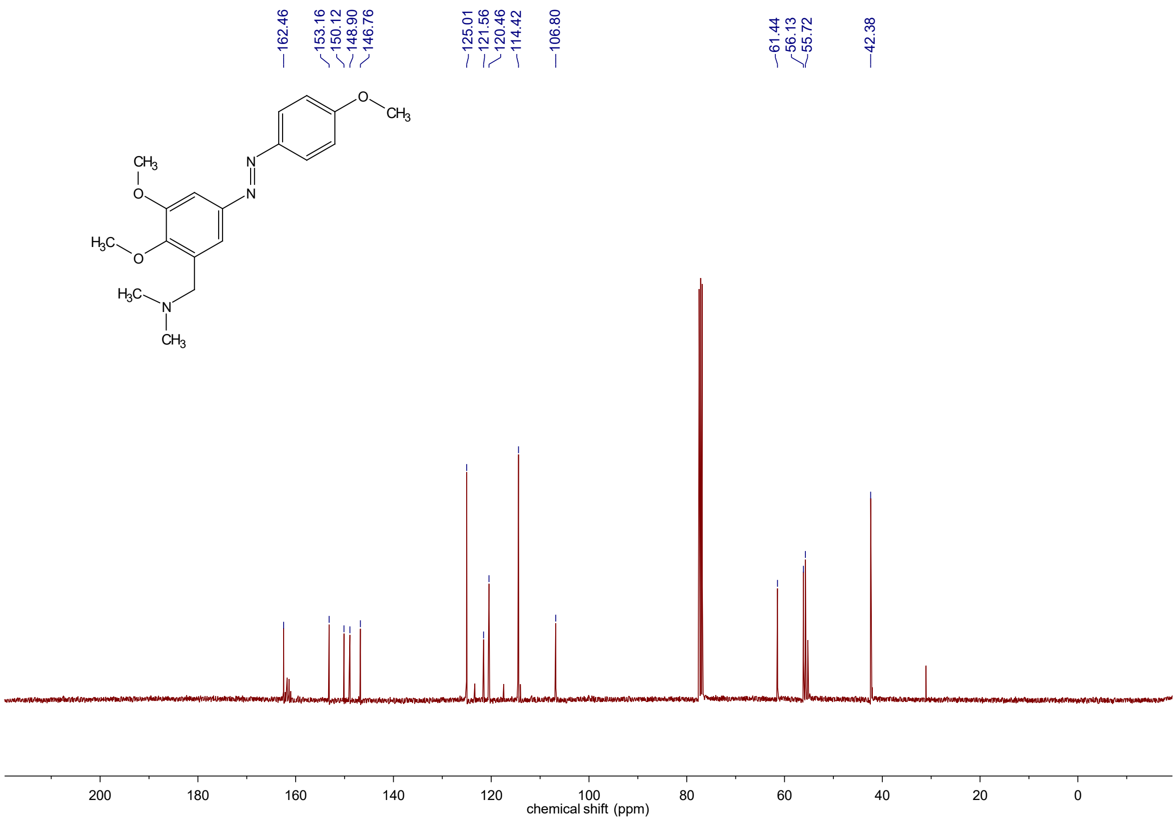

2,3-dimethoxy-5-((4-methoxyphenyl)diazenyl)phenol (**PST-26**)

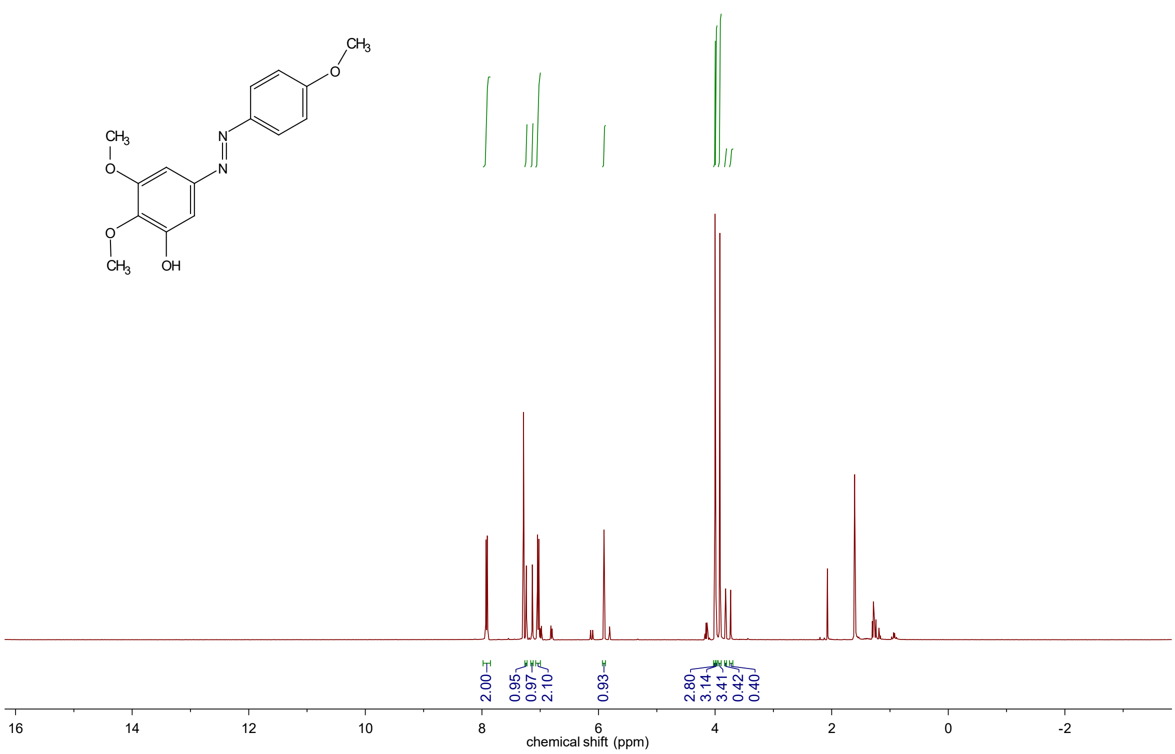

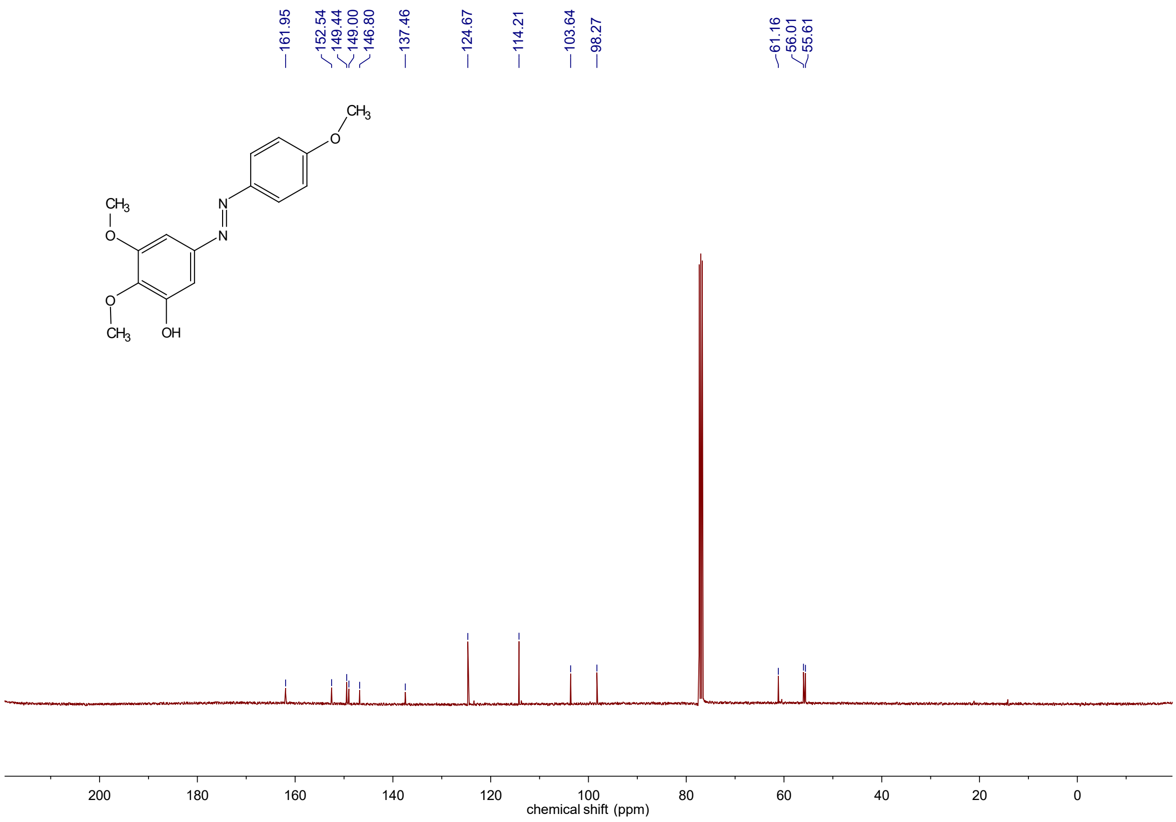

5-((3,5-diethyl-4-methoxyphenyl)diazenyl)-2-methylphenol (**PST-27**)

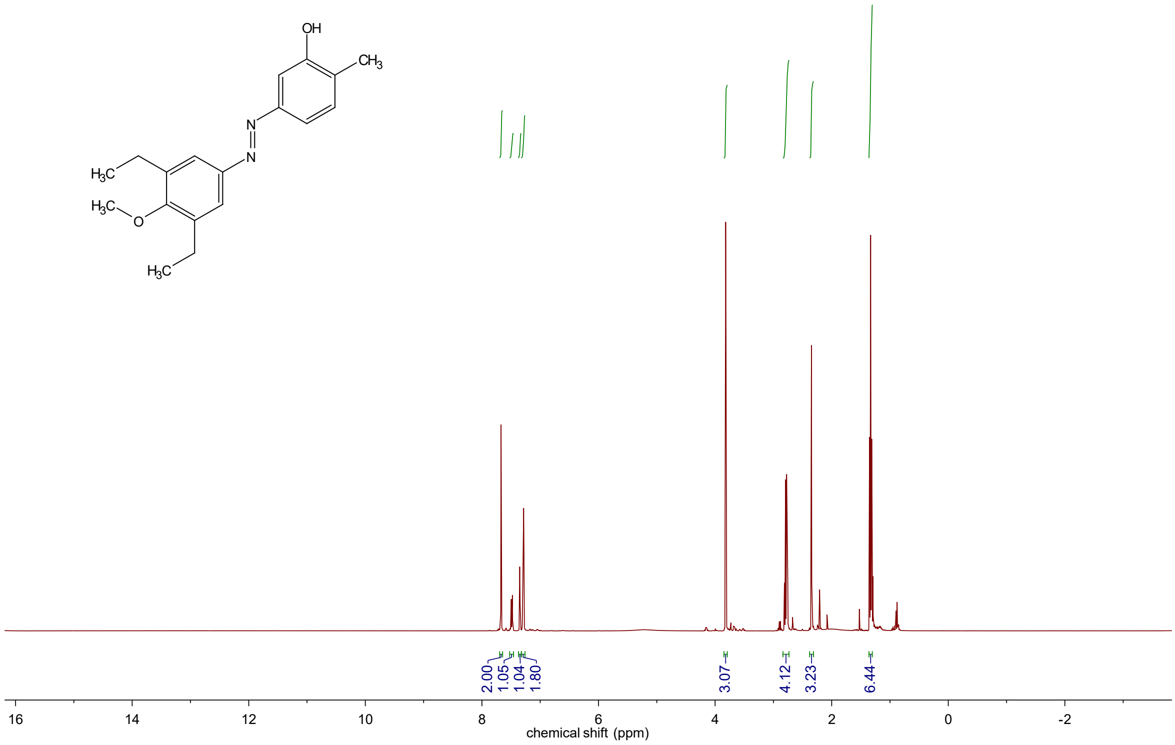

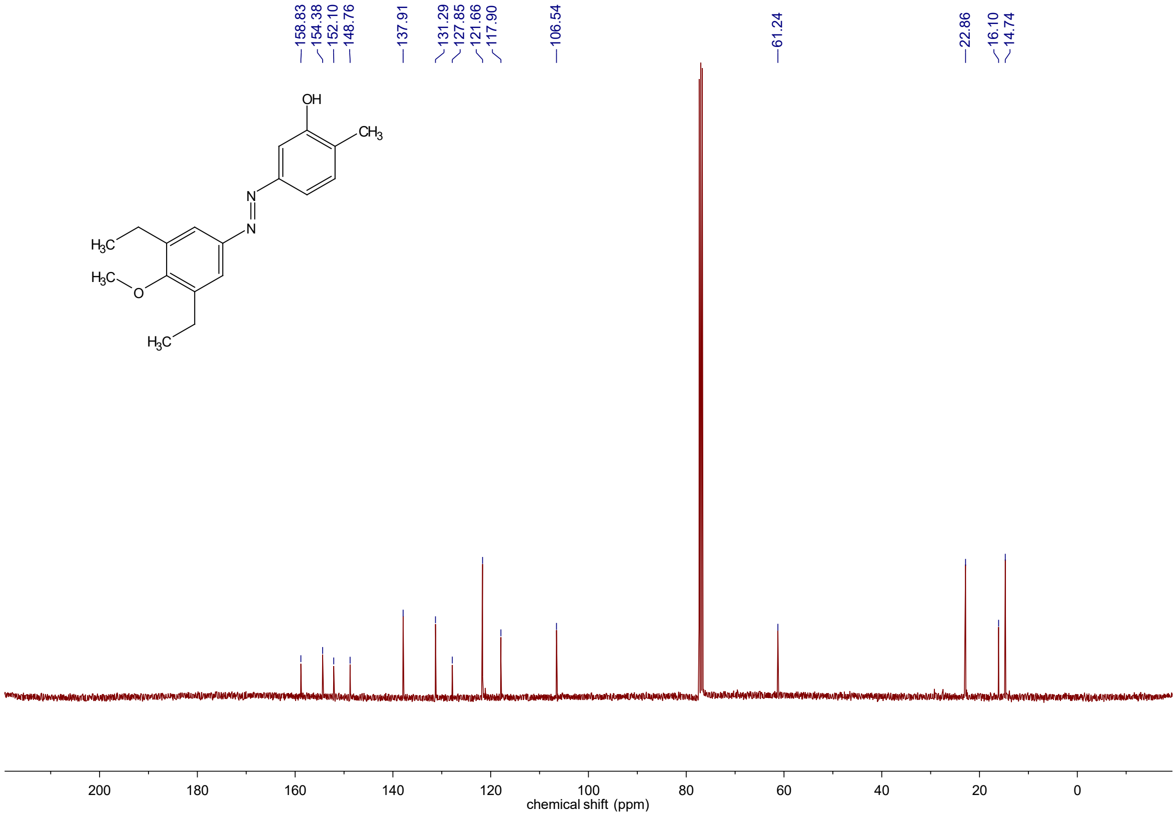

5-((3,5-diethyl-4-methoxyphenyl)diazenyl)-2-methylphenyl phosphate (**PST-27P**)

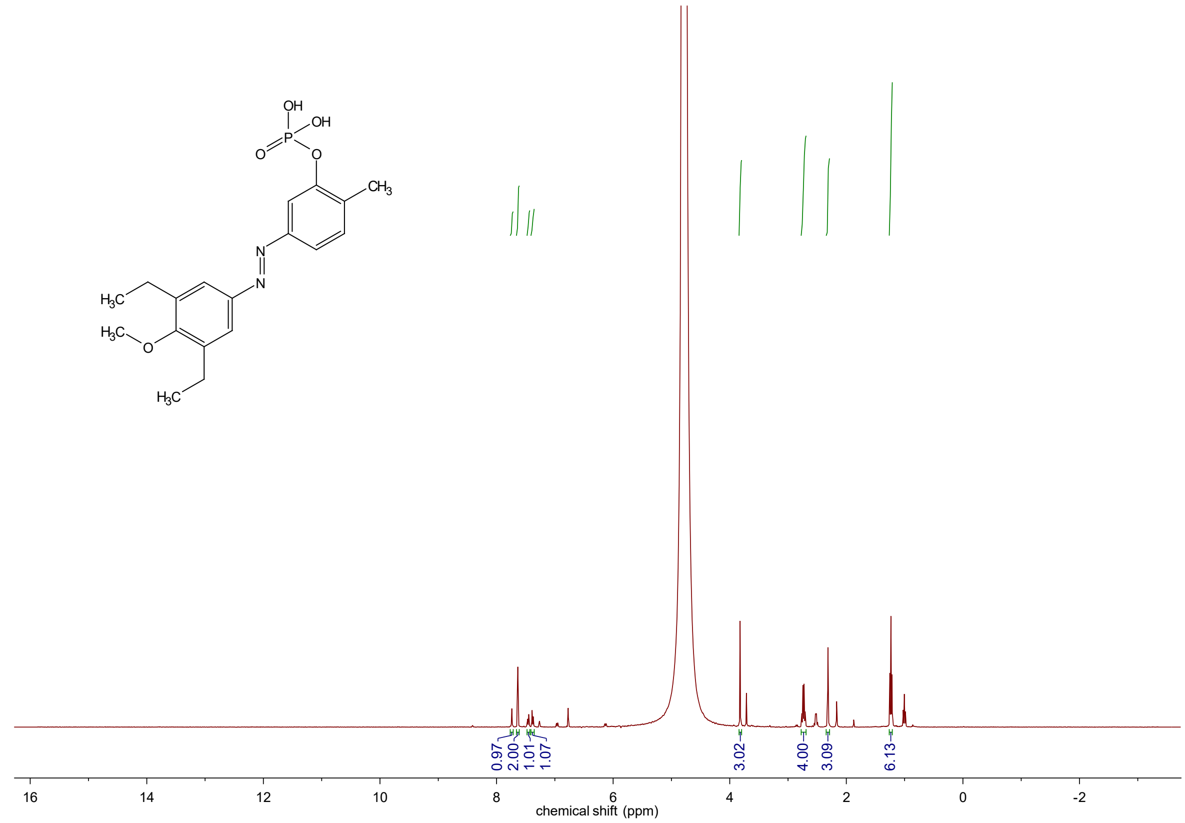

1-(3,4-dimethoxyphenyl)-2-(2,3,4,5-tetramethoxyphenyl)diazene (**PST-28**)

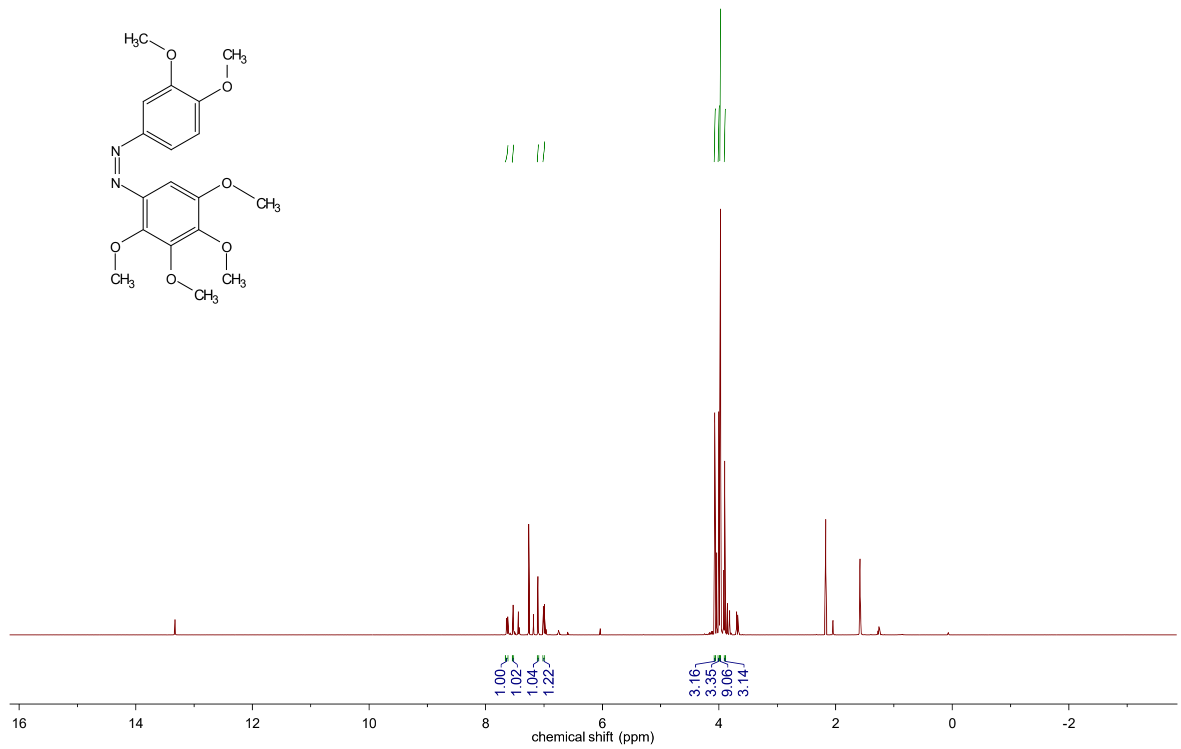

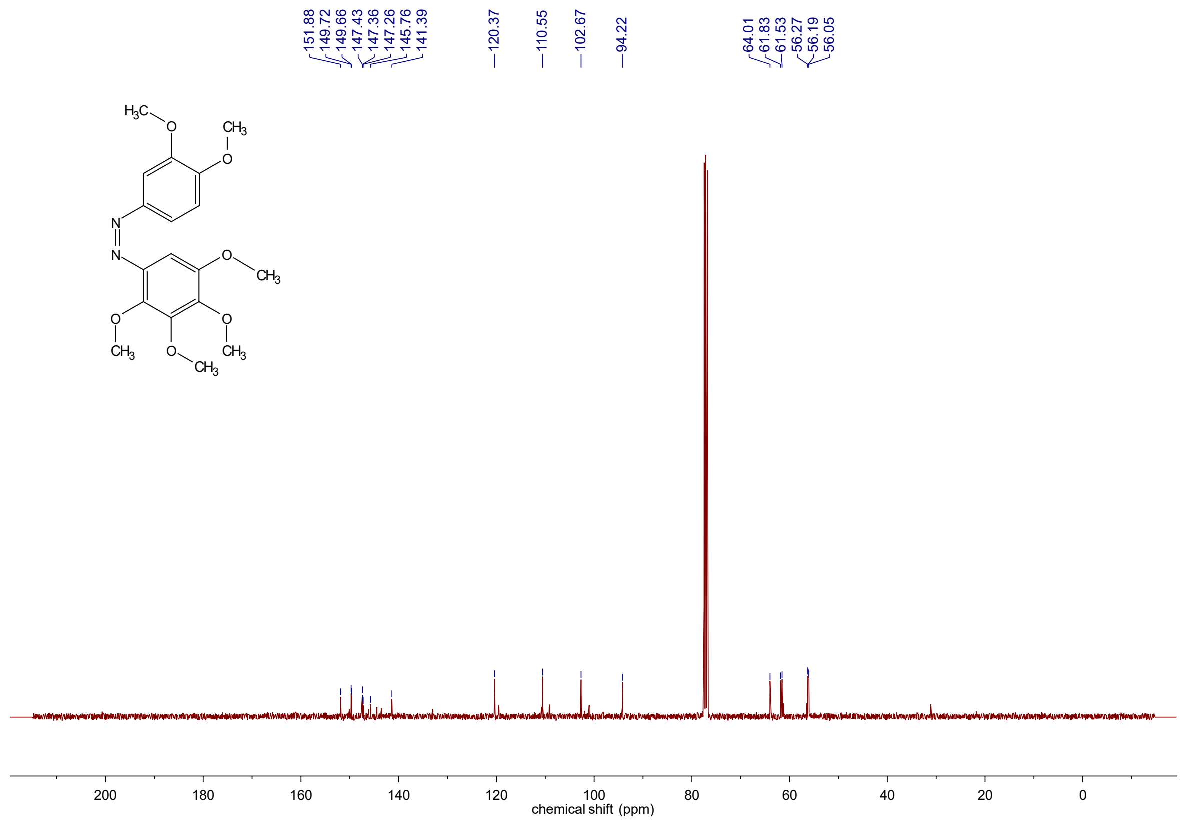

5-((5-methoxy-2-((3,4,5-trimethoxyphenyl)diazenyl)phenyl)amino)-5-oxopentanoic acid (**PST-29**)

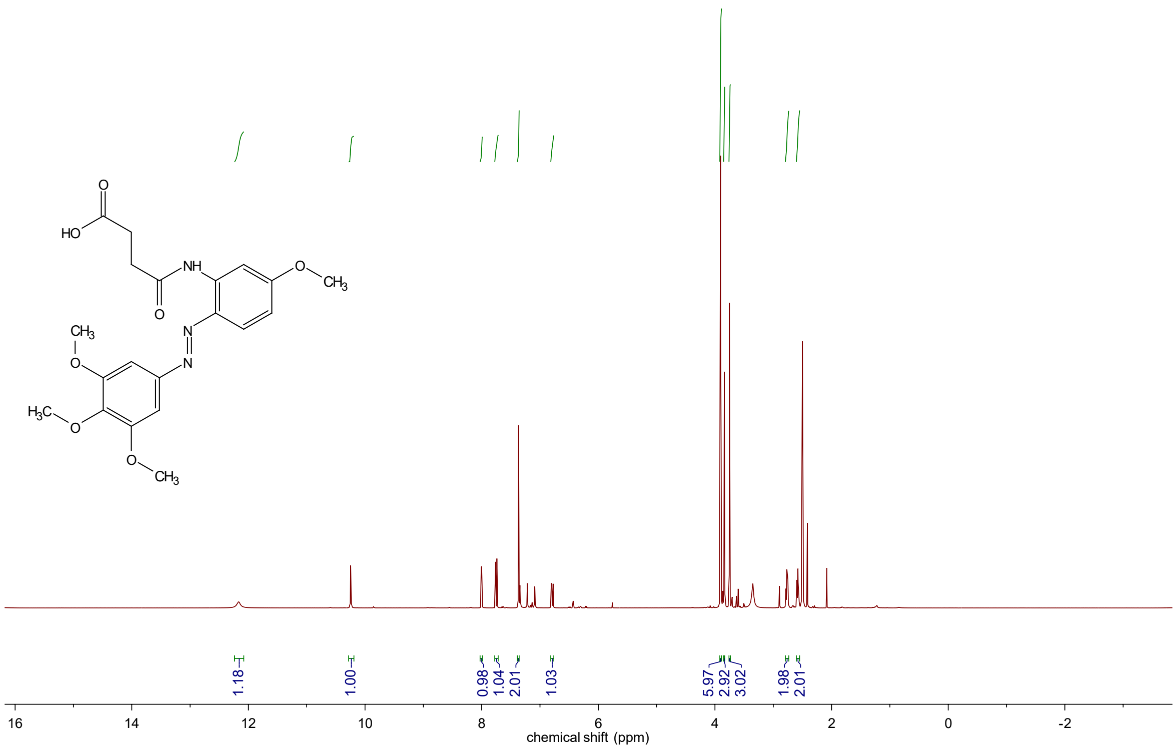

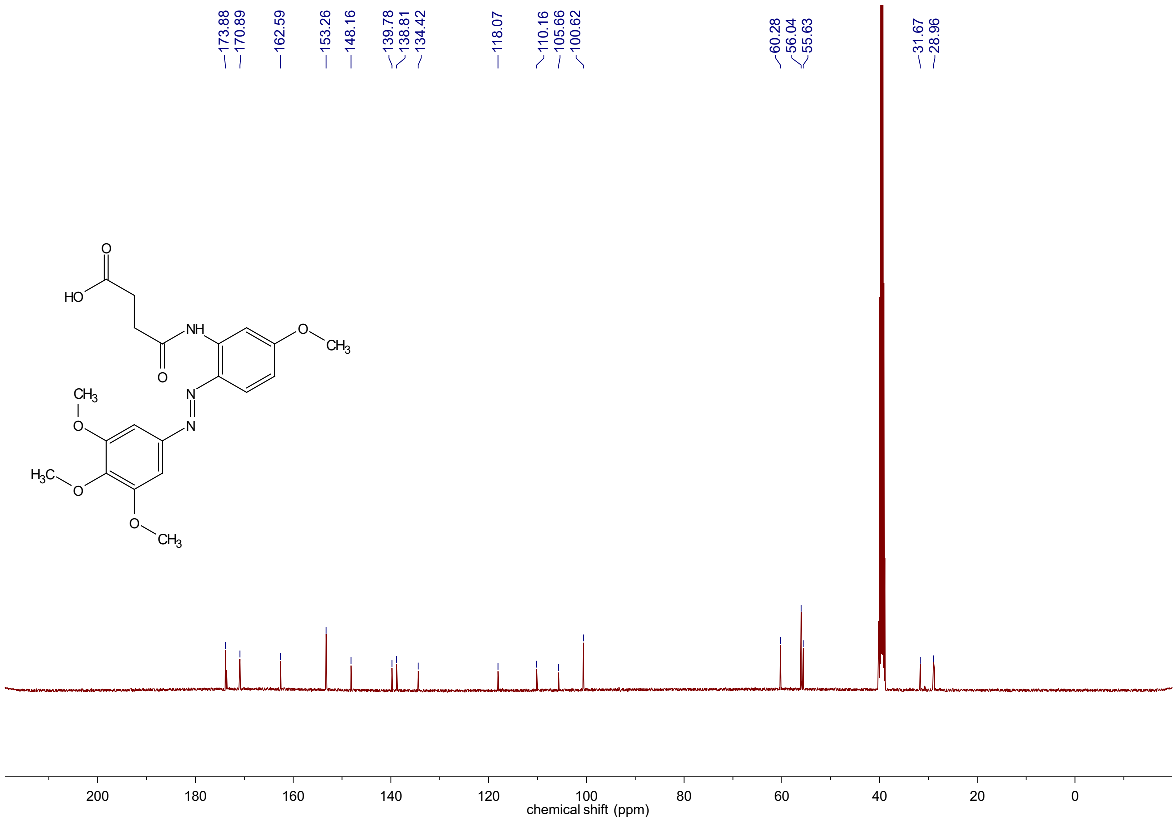

5-methoxy-2-((3,4,5-trimethoxyphenyl)diazenyl)aniline (**S29**)

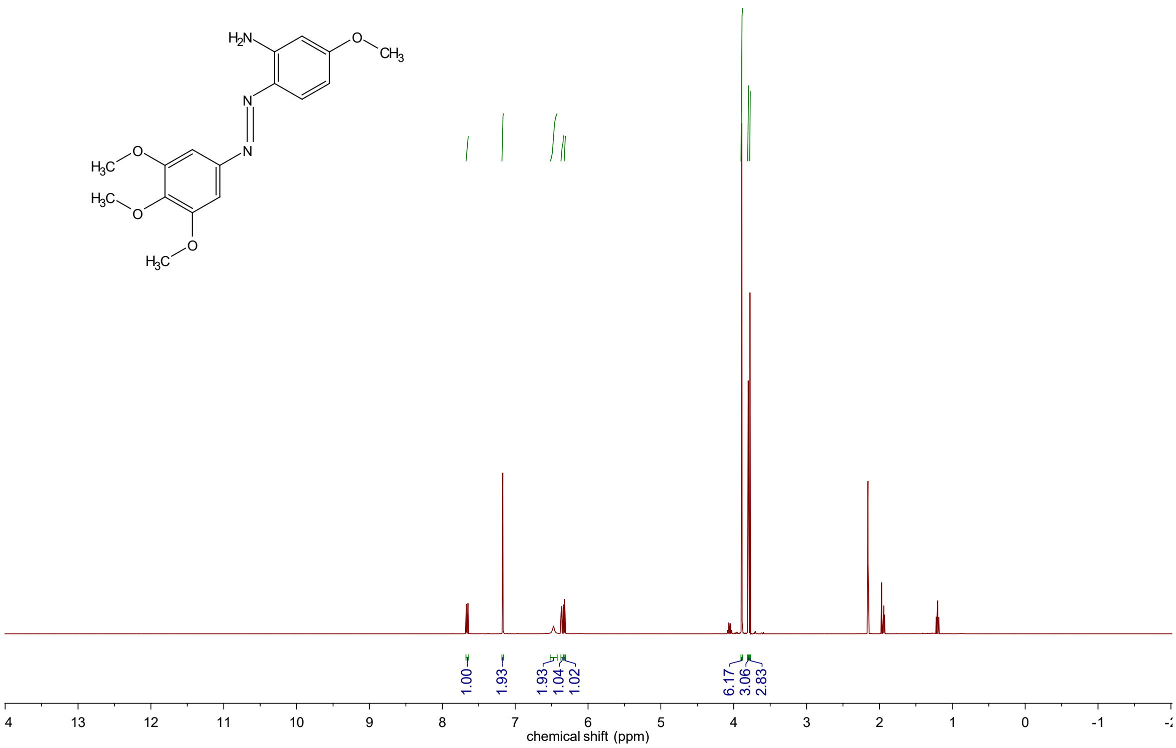

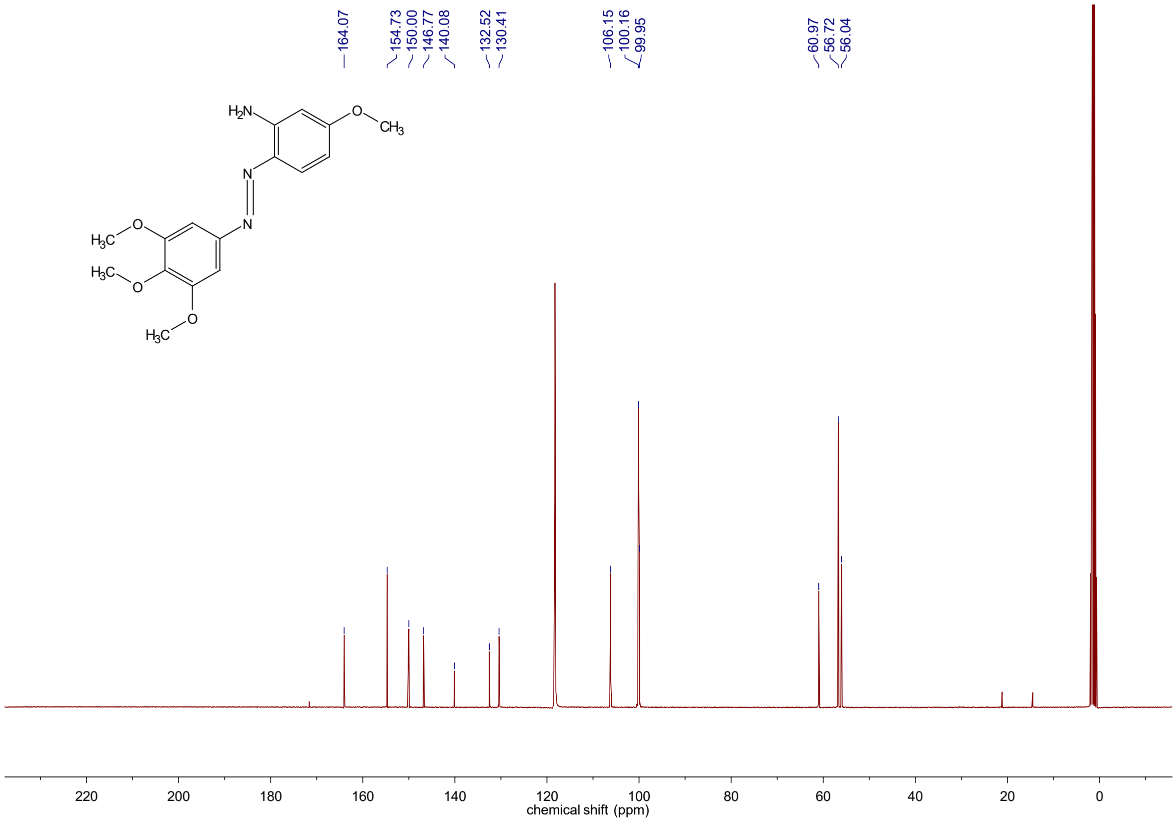

3-methoxy-2-methyl-6-((3,4,5-trimethoxyphenyl)diazenyl)phenol (**S30**)

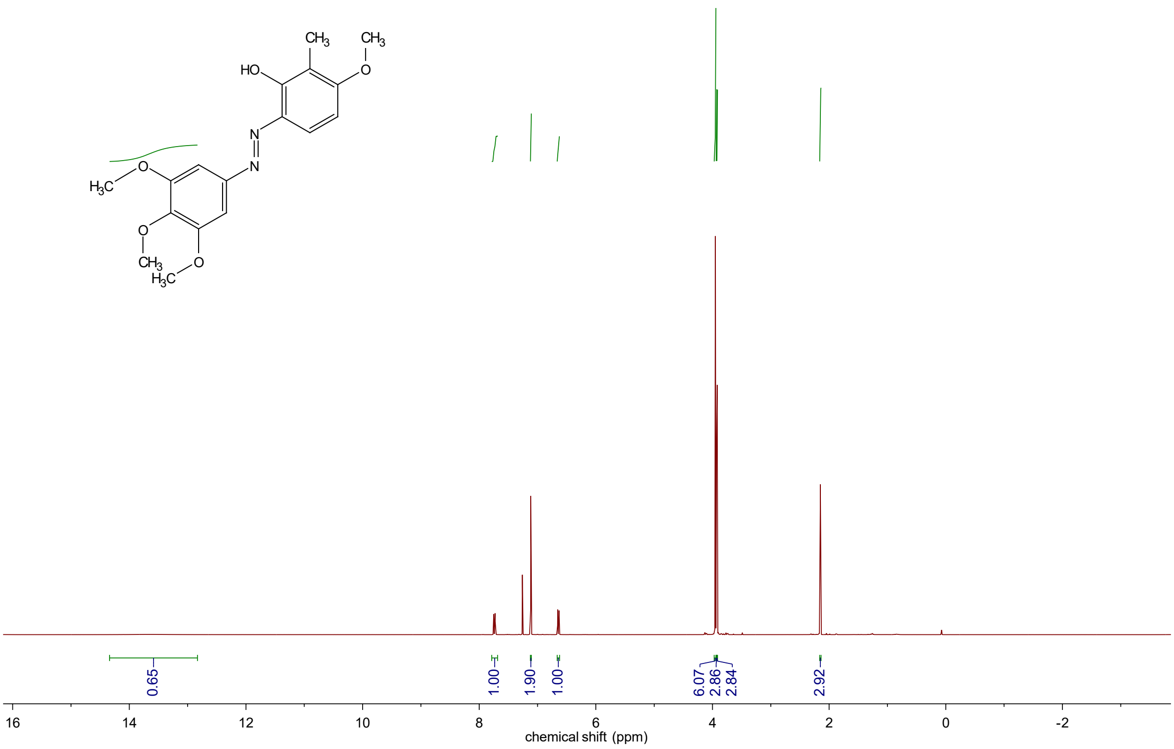

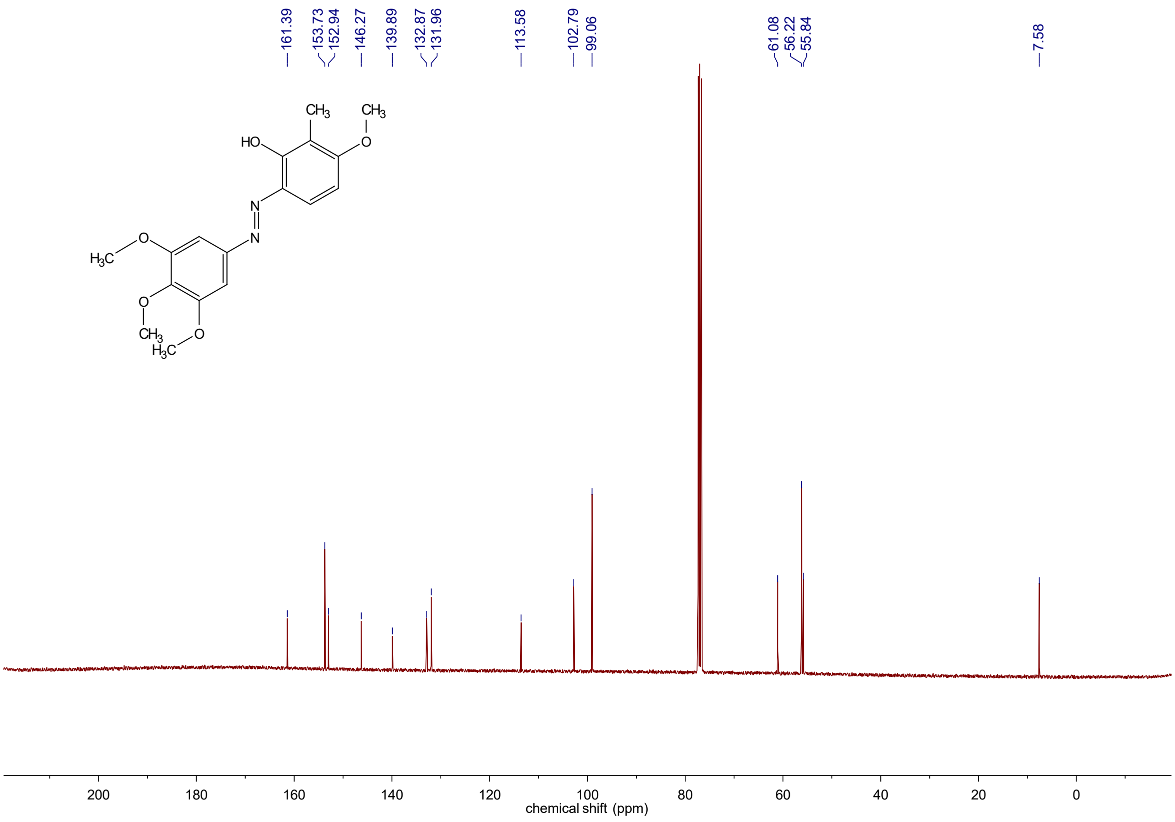

3-(3-methoxy-2-methyl-6-((3,4,5-trimethoxyphenyl)diazenyl)phenoxy)propan-1-ol (**PST-30**)

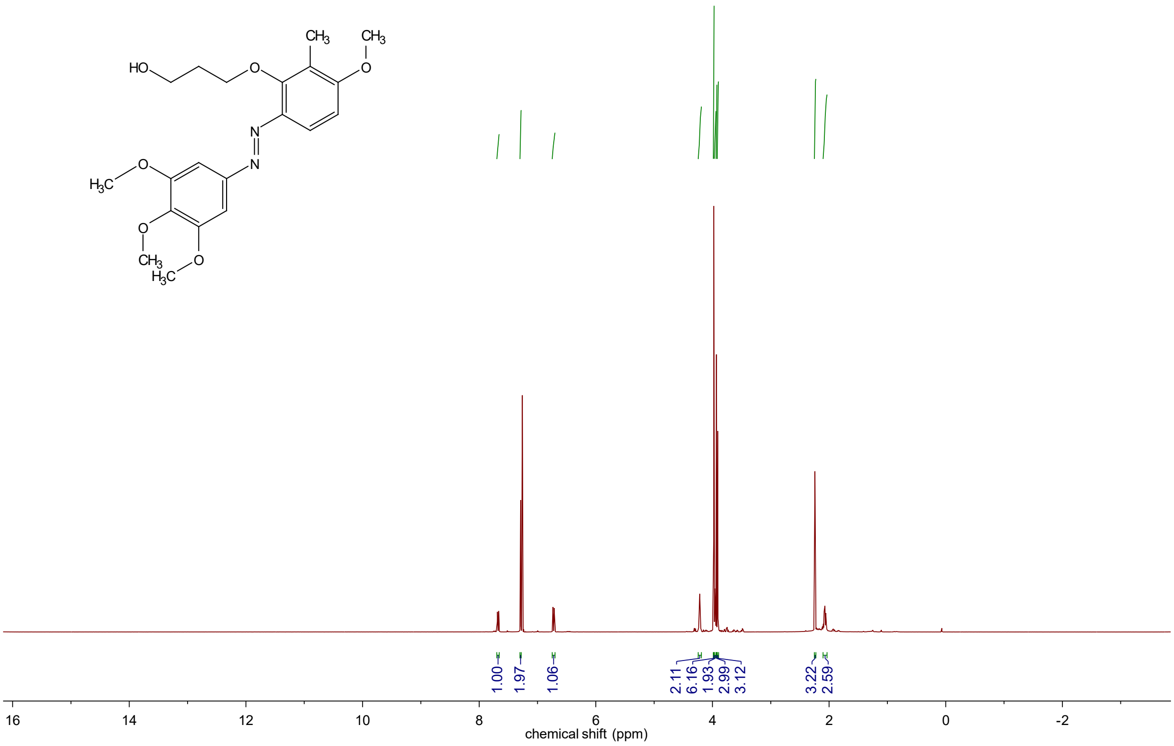

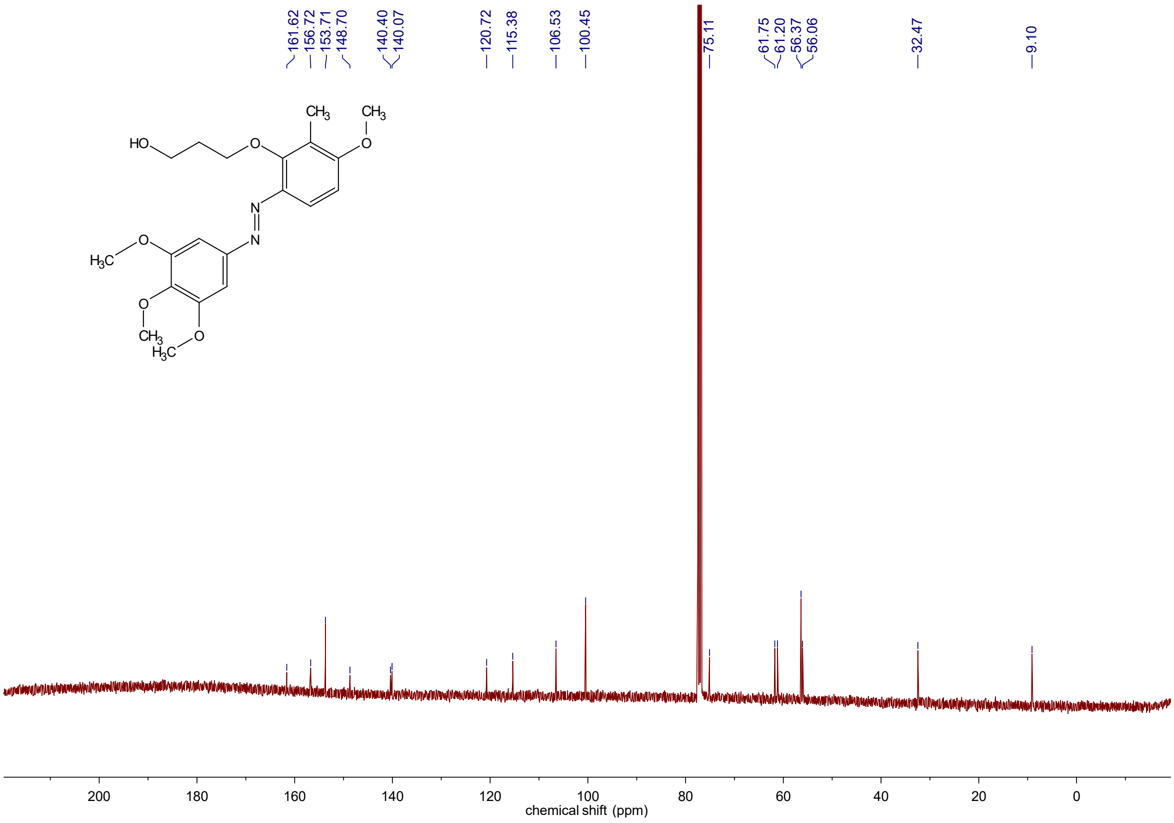

5-methoxy-2-((3,4,5-trimethoxyphenyl)diazenyl)phenol (**S28**)

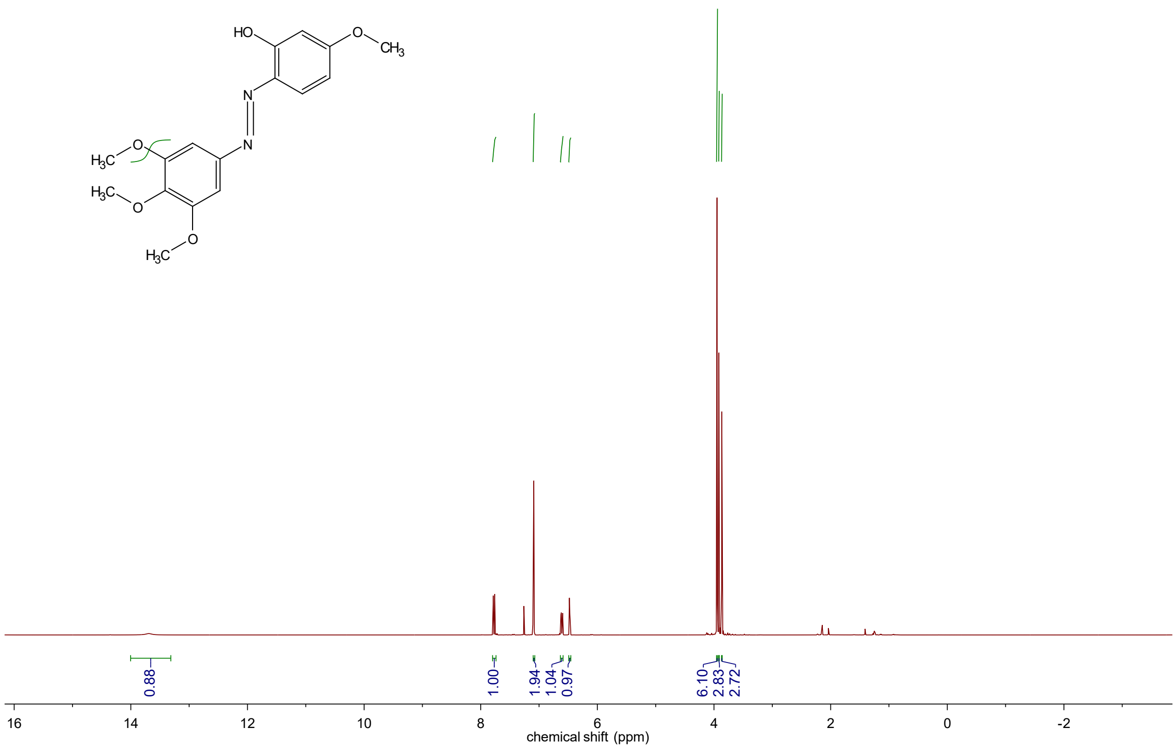

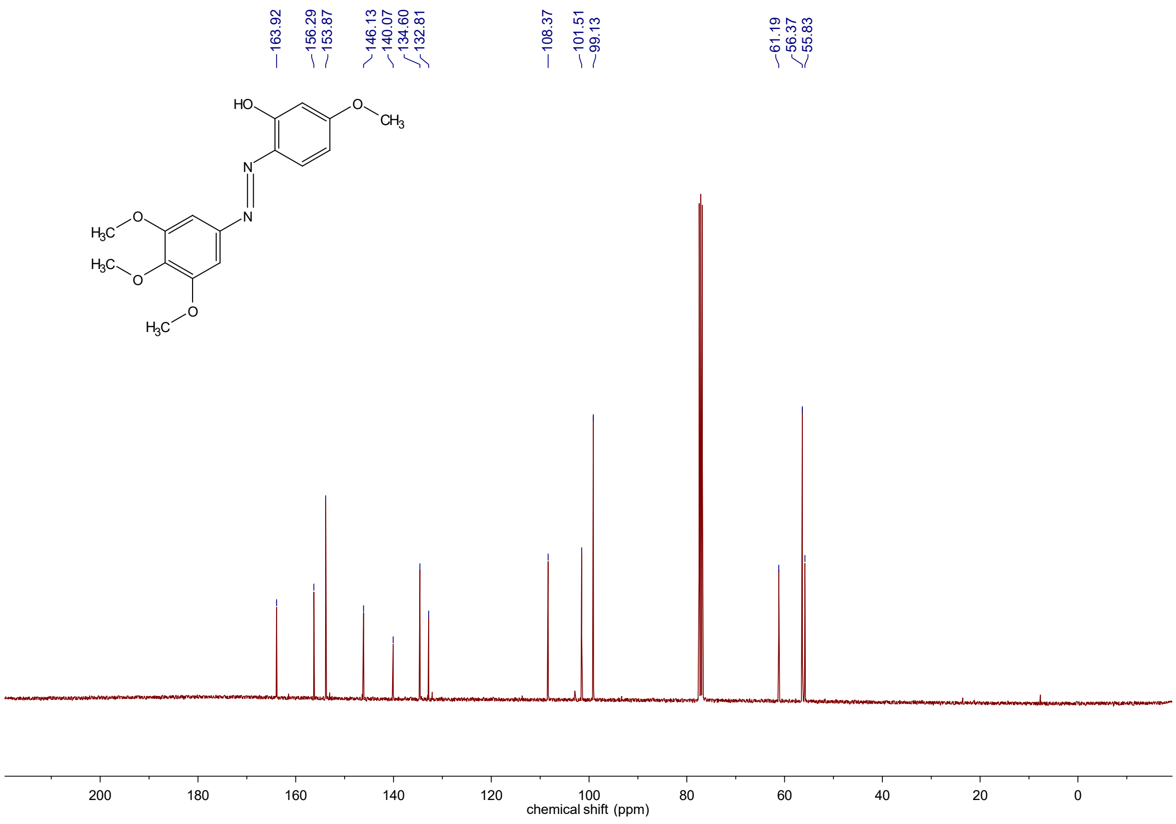

N-(6-(diethylamino)-9-(2-(4-(3-(3-methoxy-2-methyl-6-((3,4,5-trimethoxyphenyl)diazenyl)phenoxy)propyl)piperazine-1-carbonyl)phenyl)-3H-xanthen-3-ylidene)-*N*-ethylethanaminium bis(formate) salt (**MR69**)

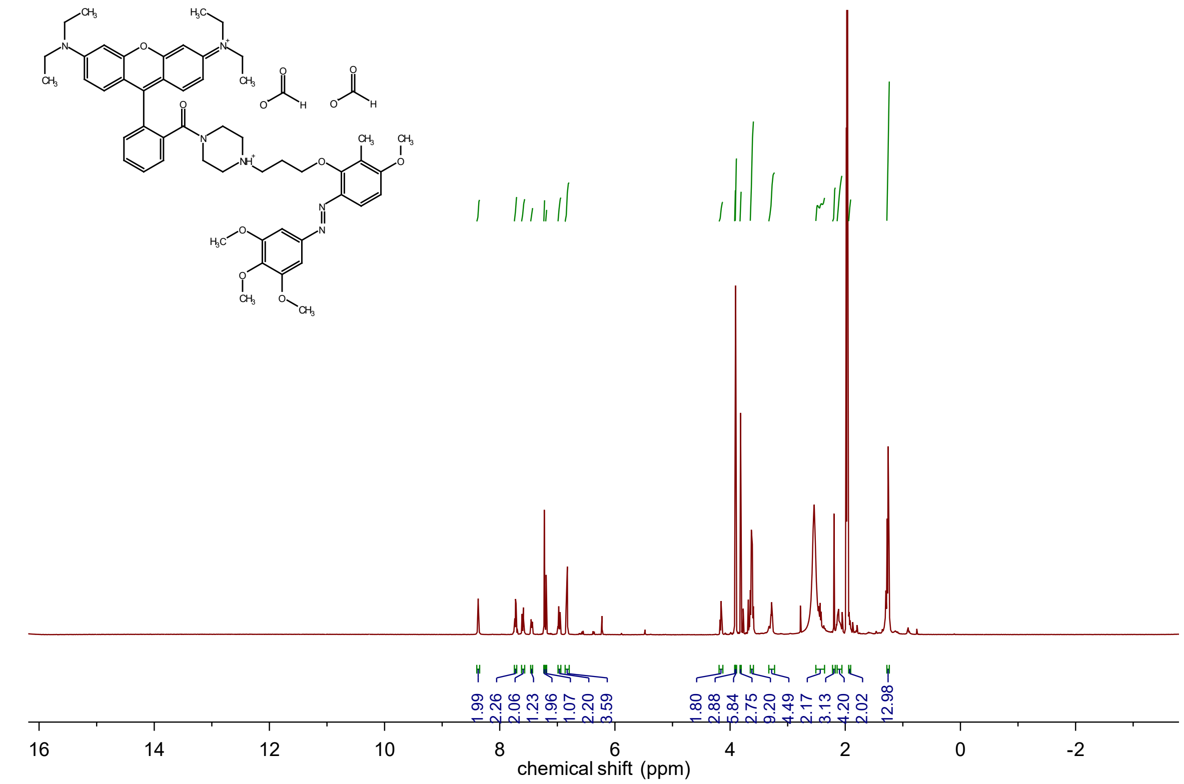

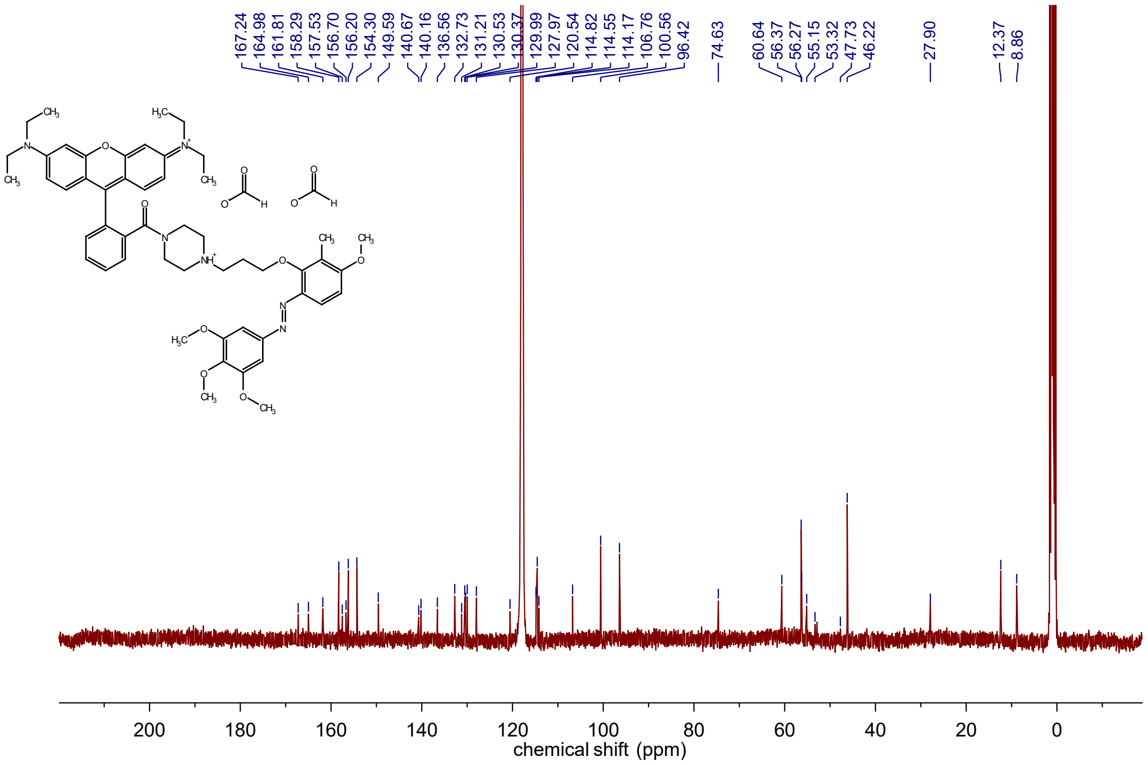

N-(5-methoxy-2-((3,4,5-trimethoxyphenyl)diazenyl)phenyl)-4-(4-(7-nitrobenzo[c][1,2,5]oxadiazol-4-yl)piperazin-1-yl)-4-oxobutanamide (**MR110**)

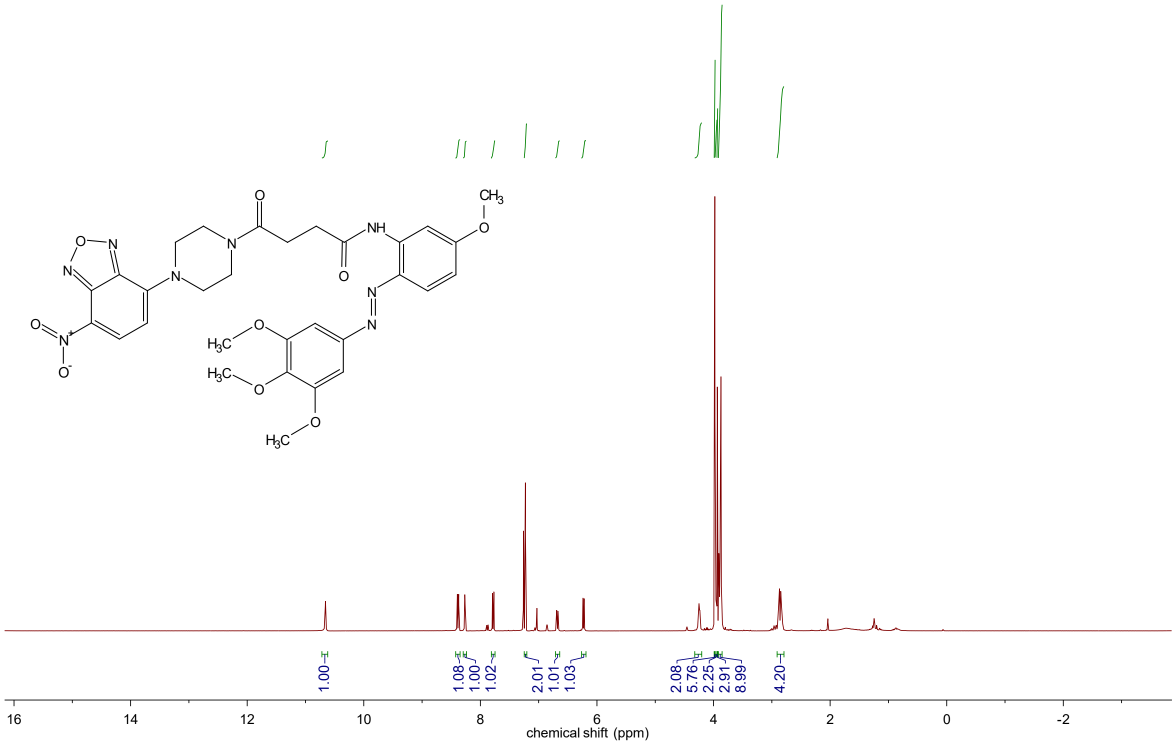

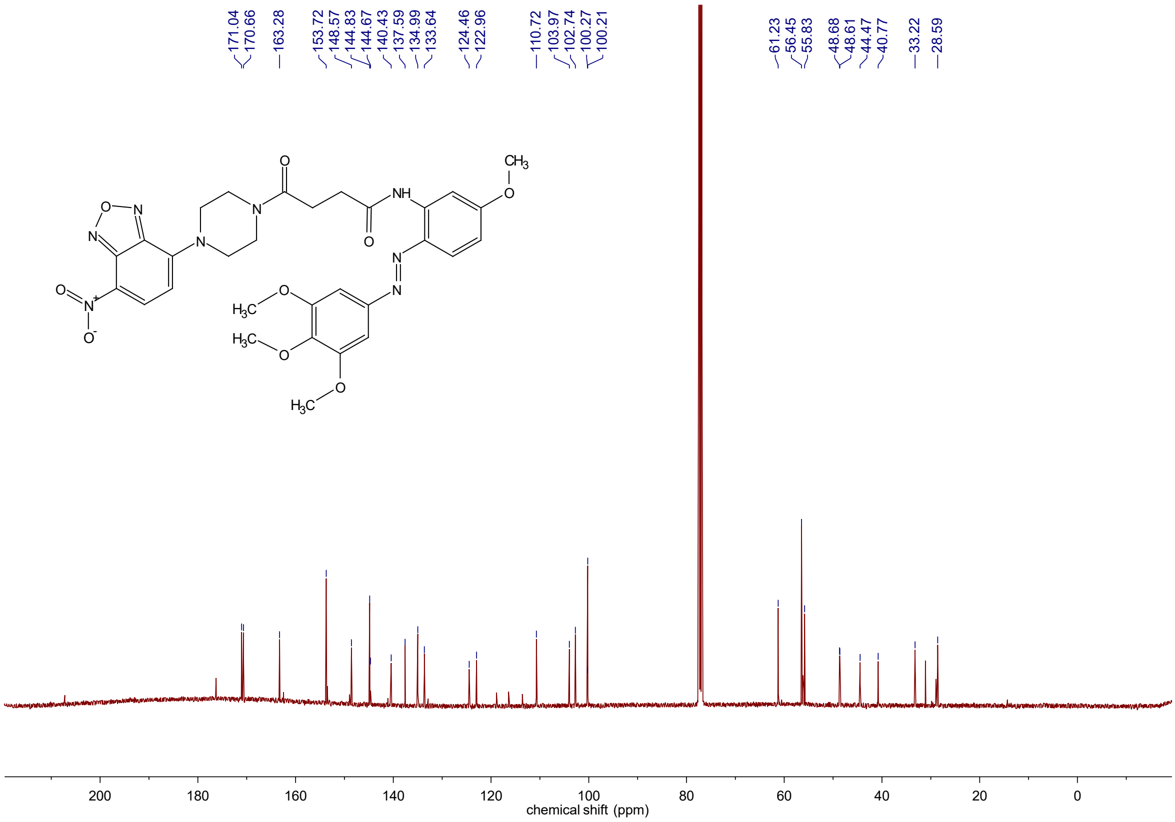

5-((3,5-diethyl-4-fluorophenyl)diazenyl)-2-methylphenyl dihydrogen phosphate (**PST-31**)

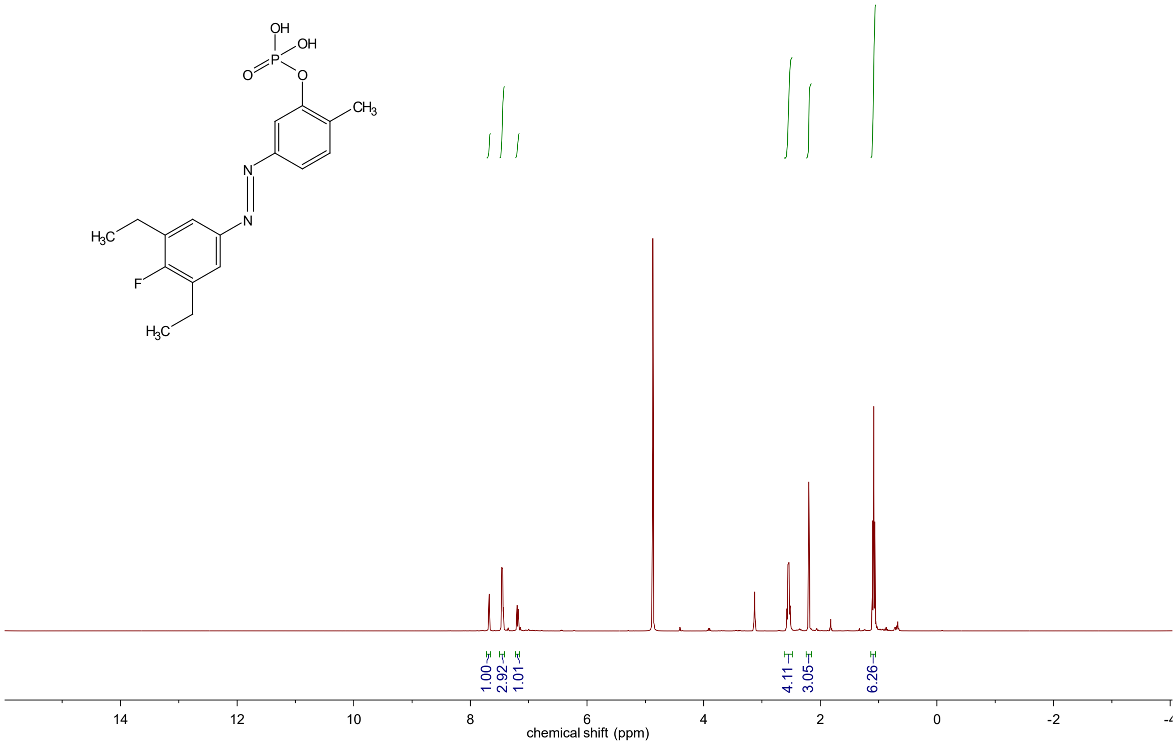

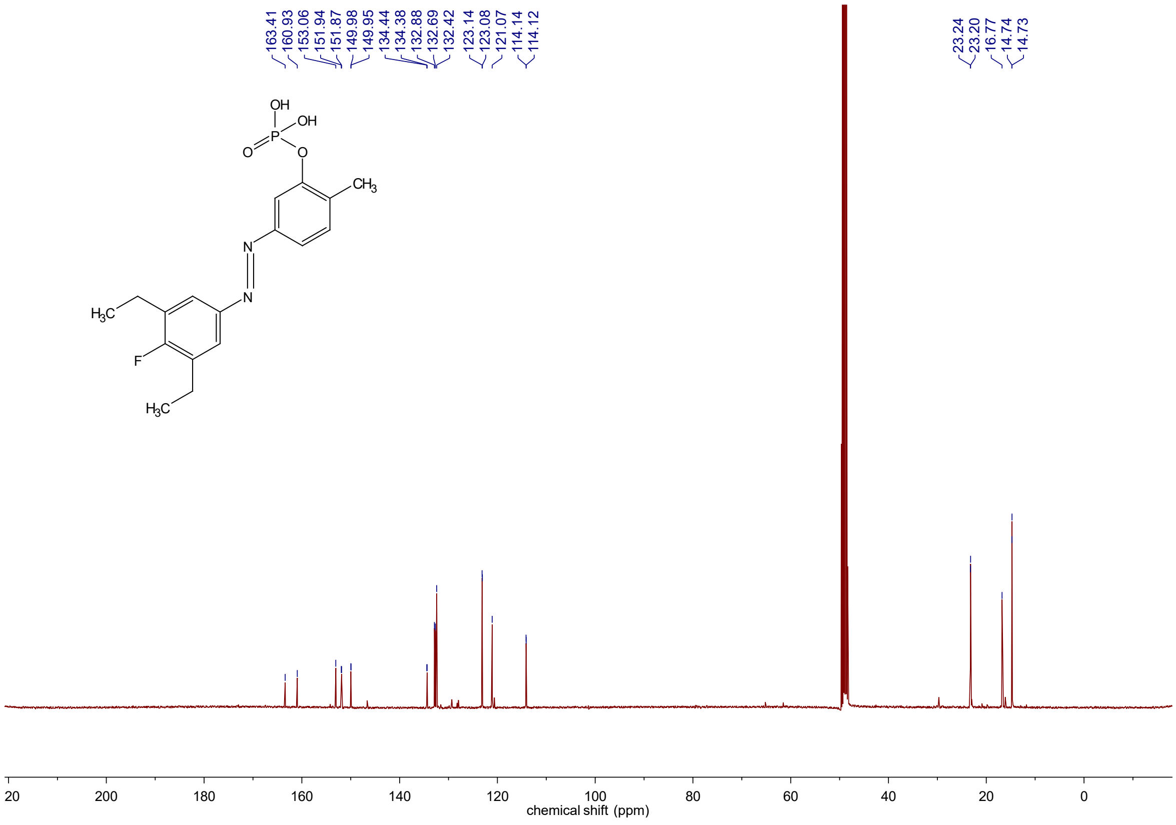

2-methoxy-5-((2,3,6,7-tetrahydro-1H,5H-pyrido[3,2,1-ij]quinolin-9-yl)diazenyl)phenyl dihydrogen phosphate (**PST-32**)

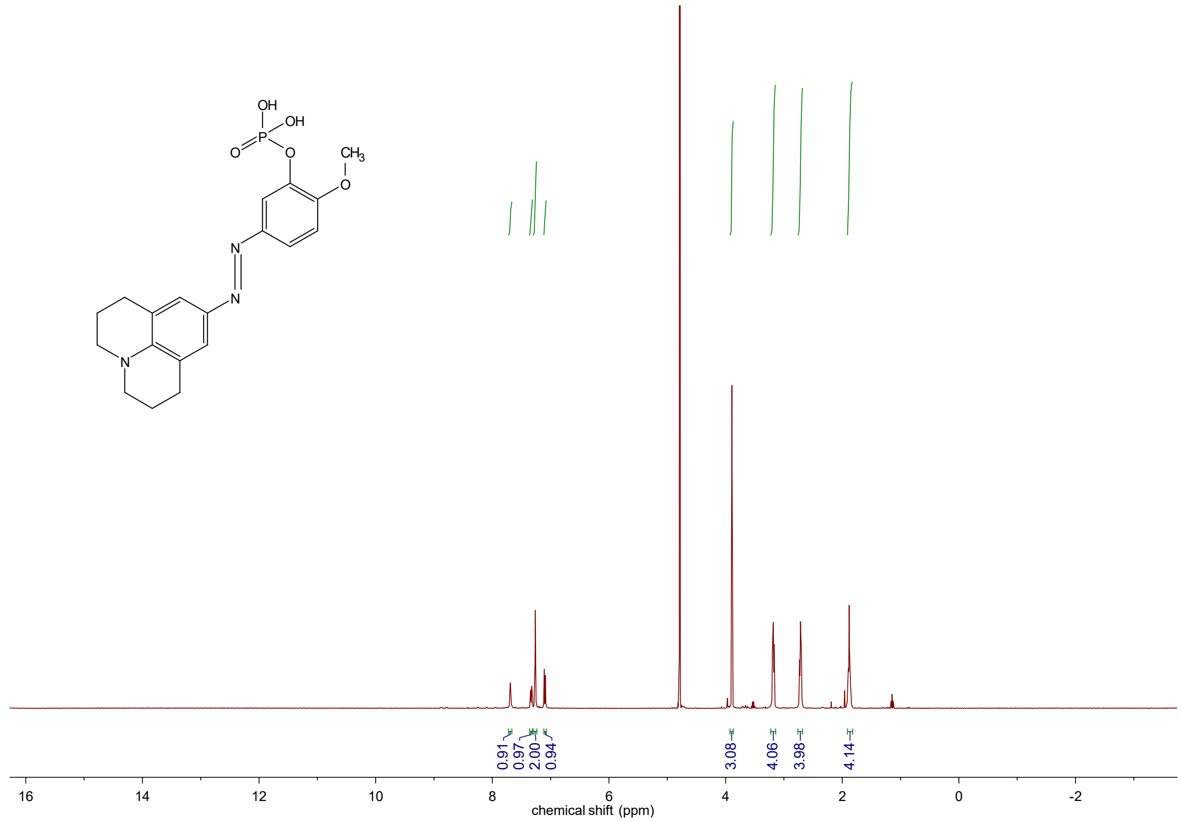

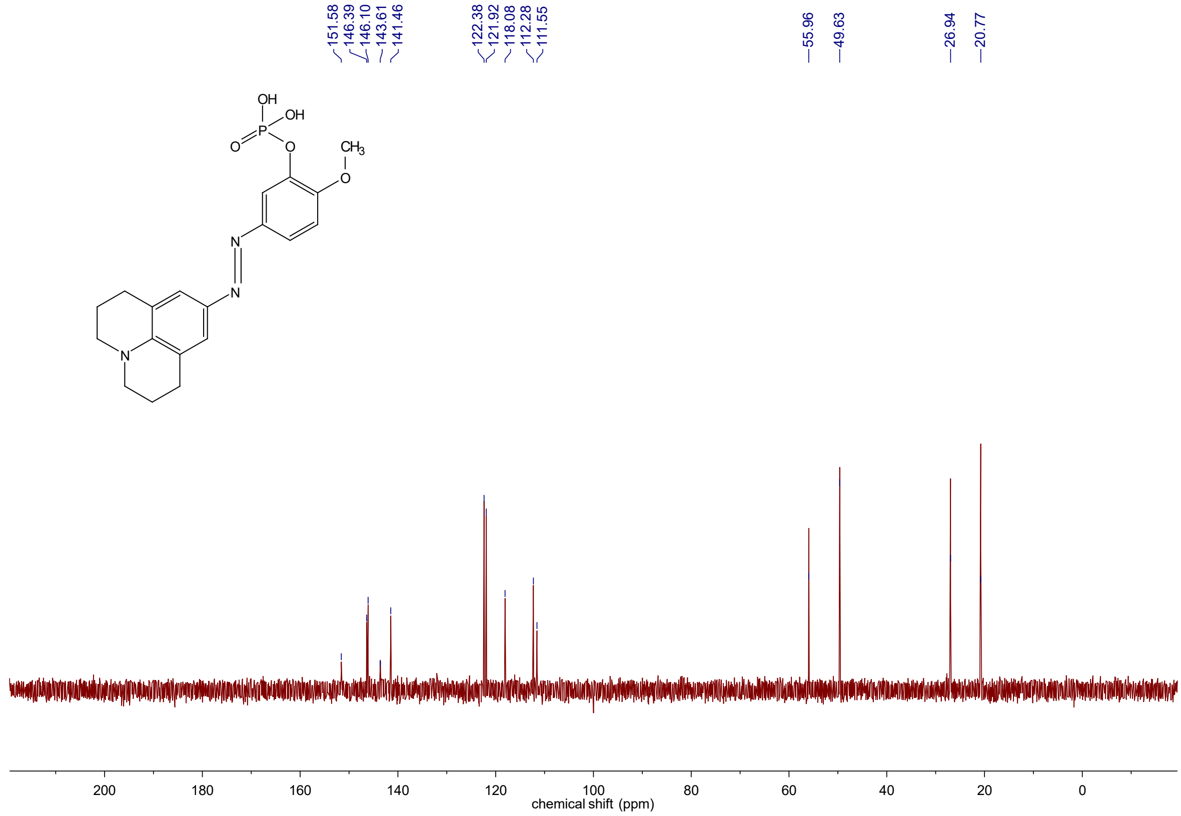

Supplemental References

[1] C. Dumontet, M. A. Jordan, *Nat Rev Drug Discov* **2010**, *9*, 790–803.
[2] Y.-L. Lian, Y.-C. Lin, *Current Opinion in Cell Biology* **2024**, *88*, 102360.
[3] T. Wittmann, A. Dema, J. van Haren, *Current Opinion in Cell Biology* **2020**, *66*, 1–10.
[4] J. C. M. Meiring, I. Grigoriev, W. Nijenhuis, L. C. Kapitein, A. Akhmanova, *Current Biology* **2022**, *32*, 4660-4674.e6.
[5] J. R. Peterson, T. J. Mitchison, *Chemistry & Biology* **2002**, *9*, 1275–1285.
[6] A. Jordan, J. A. Hadfield, N. J. Lawrence, A. T. McGown, *Medicinal Research Reviews* **1998**, *18*, 259–296.
[7] T. J. Mitchison, *MBoC* **2012**, *23*, 1–6.
[8] M.-J. Pérez-Pérez, E.-M. Priego, O. Bueno, M. S. Martins, M.-D. Canela, S. Liekens, *J. Med. Chem.* **2016**, *59*, 8685–8711.
[9] O. Thorn-Seshold, in *Molecular Photoswitches*, **2022**, pp. 873–919.
[10] W. A. Velema, W. Szymanski, B. L. Feringa, *J. Am. Chem. Soc.* **2014**, *136*, 2178–2191.
[11] O. Thorn-Seshold, J. C. M. Meiring, in *Microtubules: Methods and Protocols* (Ed.: H. Inaba), Springer US, New York, NY, **2022**, pp. 403–430.
[12] M. S. Hamaguchi, Y. Hiramoto, *Development, Growth & Differentiation* **1986**, *28*, 143–156.
[13] M. Wühr, E. S. Tan, S. K. Parker, H. W. Detrich, T. J. Mitchison, *Current Biology* **2010**, *20*, 2040–2045.
[14] J. A. Hadfield, A. T. Mcgown, S. P. Mayalarp, E. J. Land, I. Hamblett, K. Gaukroger, N. J. Lawrence, L. A. Hepworth, J. Butler, *Substituted Stilbenes, Their Reactions and Anticancer Activity*, **2002**, WO2002050007A2.
[15] R. H. Bisby, S. W. Botchway, J. A. Hadfield, A. T. Mcgown, K. M. Scherer, *Multi-Photon Isomerisation of Combretastatins and Their Use in Therapy*, **2013**, WO2013021208A2.
[16] L. Gao, J. C. M. Meiring, Y. Kraus, M. Wranik, T. Weinert, S. D. Pritzl, R. Bingham, E. Ntouliou, K. I. Jansen, N. Olieric, J. Standfuss, L. C. Kapitein, T. Lohmüller, J. Ahlfeld, A. Akhmanova, M. O. Steinmetz, O. Thorn-Seshold, *Cell Chemical Biology* **2021**, *28*, 228-241.e6.
[17] L. Gao, J. C. M. Meiring, A. Varady, I. E. Ruider, C. Heise, M. Wranik, C. D. Velasco, J. A. Taylor, B. Terni, T. Weinert, J. Standfuss, C. C. Cabernard, A. Llobet, M. O. Steinmetz, A. R. Bausch, M. Distel, J. Thorn-Seshold, A. Akhmanova, O. Thorn-Seshold, *J. Am. Chem. Soc.* **2022**, *144*, 5614–5628.
[18] M. Medarde, A. B. S. Maya, C. Pérez-Melero, *Journal of Enzyme Inhibition and Medicinal Chemistry* **2004**, *19*, 521–540.
[19] C. Álvarez, R. Álvarez, P. Corchete, C. Pérez-Melero, R. Peláez, M. Medarde, *European Journal of Medicinal Chemistry* **2010**, *45*, 588–597.
[20] S. Jiang, C. Crogan-Grundy, J. Drewe, B. Tseng, S. X. Cai, *Bioorganic & Medicinal Chemistry Letters* **2008**, *18*, 5725–5728.
[21] G. R. Reddy, C.-C. Kuo, U.-K. Tan, M. S. Coumar, C.-Y. Chang, Y.-K. Chiang, M.-J. Lai, J.-Y. Yeh, S.-Y. Wu, J.-Y. Chang, J.-P. Liou, H.-P. Hsieh, *J. Med. Chem.* **2008**, *51*, 8163–8167.
[22] O. M. Friedman, R. M. Gofstein, A. M. Seligman, *J. Am. Chem. Soc.* **1949**, *71*, 3010–3013.
[23] S. K. Rastogi, Z. Zhao, S. L. Barrett, S. D. Shelton, M. Zafferani, H. E. Anderson, M. O. Blumenthal, L. R. Jones, L. Wang, X. Li, C. N. Streu, L. Du, W. J. Brittain, *European Journal of Medicinal Chemistry* **2018**, *143*, 1–7.
[24] G. C. Tron, T. Pirali, G. Sorba, F. Pagliai, S. Busacca, A. A. Genazzani, *J. Med. Chem.* **2006**, *49*, 3033–3044.
[25] P. Ball, C. H. Nicholls, *Dyes and Pigments* **1982**, *3*, 5–26.
[26] M. Borowiak, W. Nahaboo, M. Reynders, K. Nekolla, P. Jalinot, J. Hasserodt, M. Rehberg, M. Delattre, S. Zahler, A. Vollmar, D. Trauner, O. Thorn-Seshold, *Cell* **2015**, *162*, 403–411.

[27] J. Zenker, M. D. White, R. M. Templin, R. G. Parton, O. Thorn-Seshold, S. Bissiere, N. Plachta, *Science* **2017**, *357*, 925–928.
[28] K. Eguchi, Z. Taoufiq, O. Thorn-Seshold, D. Trauner, M. Hasegawa, T. Takahashi, *J. Neurosci.* **2017**, *37*, 6043–6052.
[29] M. Sawada, N. Ohno, M. Kawaguchi, S. Huang, T. Hikita, Y. Sakurai, H. Bang Nguyen, T. Quynh Thai, Y. Ishido, Y. Yoshida, H. Nakagawa, A. Uemura, K. Sawamoto, *The EMBO Journal* **2018**, *37*, e97404.
[30] J. Moreno, L. Grubert, J. Schwarz, D. Bléger, S. Hecht, *Chemistry – A European Journal* **2017**, *23*, 14090–14095.
[31] Z. Liu, P. J. Sadler, *Acc. Chem. Res.* **2014**, *47*, 1174–1185.
[32] K. K.-W. Lo, K. Y. Zhang, *RSC Adv.* **2012**, *2*, 12069–12083.
[33] A. A. Beharry, O. Sadovski, G. A. Woolley, *J. Am. Chem. Soc.* **2011**, *133*, 19684–19687.
[34] D. Bléger, J. Schwarz, A. M. Brouwer, S. Hecht, *J. Am. Chem. Soc.* **2012**, *134*, 20597–20600.
[35] S. Samanta, A. Babalhavaeji, M. Dong, G. A. Woolley, *Angewandte Chemie International Edition* **2013**, *52*, 14127–14130.
[36] R. Siewertsen, H. Neumann, B. Buchheim-Stehn, R. Herges, C. Näther, F. Renth, F. Temps, *J. Am. Chem. Soc.* **2009**, *131*, 15594–15595.
[37] S. Samanta, A. A. Beharry, O. Sadovski, T. M. McCormick, A. Babalhavaeji, V. Tropepe, G. A. Woolley, *J. Am. Chem. Soc.* **2013**, *135*, 9777–9784.
[38] F. M. Raymo, M. Tomasulo, *Chem. Soc. Rev.* **2005**, *34*, 327–336.
[39] A. Chevalier, P.-Y. Renard, A. Romieu, *Tetrahedron Letters* **2014**, *55*, 6759–6763.
[40] T. B. Norsten, N. R. Branda, *J. Am. Chem. Soc.* **2001**, *123*, 1784–1785.
[41] A. J. Myles, N. R. Branda, *Advanced Functional Materials* **2002**, *12*, 167–173.
[42] M. Izquierdo-Serra, M. Gascón-Moya, J. J. Hirtz, S. Pittolo, K. E. Poskanzer, È. Ferrer, R. Alibés, F. Busqué, R. Yuste, J. Hernando, P. Gorostiza, *J. Am. Chem. Soc.* **2014**, *136*, 8693–8701.
[43] J. Moreno, M. Gerecke, L. Grubert, S. A. Kovalenko, S. Hecht, *Angewandte Chemie International Edition* **2016**, *55*, 1544–1547.
[44] D. Bléger, S. Hecht, *Angewandte Chemie International Edition* **2015**, *54*, 11338–11349.
[45] B. Baumgartner, V. Glembockyte, R. Mayer, A. Gonzalez-Hernandez, R. Kindler, A. Valavalkar, A. Wiegand, A. Müller-Deku, L. Grubert, F. Steiner, C. Gross, M. Reynders, V. Grenier, J. Broichhagen, S. Hecht, P. Tinnefeld, A. Ofial, B. Dietzek-Ivansic, J. Levitz, O. Thorn-Seshold, *ChemRxiv* **2023**, DOI 10.26434/chemrxiv-2023-37sv4.
[46] B. Baumgartner, V. Glembockyte, A. Gonzalez-Hernandez, A. Valavalkar, R. Mayer, L. Fillbrook, A. Müller-Deku, J. Zhang, F. Steiner, A. Wiegand, C. Gross, M. Reynders, H. Munguba, A. Arefin, A. Ofial, J. Beves, T. Lohmüller, B. Dietzek-Ivansic, J. Broichhagen, P. Tinnefeld, J. Levitz, O. Thorn-Seshold, *ChemRxiv* **2024**, DOI 10.26434/chemrxiv-2024-vm4n3.
[47] O. Thorn-Seshold, M. Borowiak, D. Trauner, J. Hasserodt, *Azoaryls as Reversibly Modulatable Tubulin Inhibitors*, **2014**, WO2015166295.
[48] A. Goulet-Hanssens, M. Utecht, D. Mutruc, E. Titov, J. Schwarz, L. Grubert, D. Bléger, P. Saalfrank, S. Hecht, *J. Am. Chem. Soc.* **2017**, *139*, 335–341.
[49] S. Lee, Y. You, K. Ohkubo, S. Fukuzumi, W. Nam, *Chem. Sci.* **2014**, *5*, 1463–1474.
[50] R. Gaspari, A. E. Prota, K. Bargsten, A. Cavalli, M. O. Steinmetz, *Chem* **2017**, *2*, 102–113.
[51] Y. Kraus, C. Glas, B. Melzer, L. Gao, C. Heise, M. Preuße, J. Ahlfeld, F. Bracher, O. Thorn-Seshold, *European Journal of Medicinal Chemistry* **2020**, *186*, 111865.
[52] T. Nguyen, M. B. Francis, *Org. Lett.* **2003**, *5*, 3245–3248.
[53] T. Fehrentz, M. Schönberger, D. Trauner, *Angewandte Chemie International Edition* **2011**, *50*, 12156–12182.
[54] H. E. Gottlieb, V. Kotlyar, A. Nudelman, *J. Org. Chem.* **1997**, *62*, 7512–7515.
[55] S. Dixit, Q. T. Siddiqui, M. Muneer, N. Agarwal, *Tetrahedron Letters* **2016**, *57*, 4228–4231.
[56] G. S. Hartley, *Nature* **1937**, *140*, 281–281.

[57] R. H. Bisby, S. W. Botchway, J. A. Hadfield, A. T. McGown, A. W. Parker, K. M. Scherer, *European Journal of Cancer* **2012**, *48*, 1896–1903.
[58] M. Matsumoto, K. Kobayashi, Y. Hotta, *J. Org. Chem.* **1984**, *49*, 4740–4741.
[59] Y. L. Choi, H. S. Lim, H. J. Lim, J.-N. Heo, *Org. Lett.* **2012**, *14*, 5102–5105.
[60] M. A. Brimble, S. J. Phythian, H. Prabaharan, *J. Chem. Soc., Perkin Trans. 1* **1995**, 2855–2860.
[61] L. Wang, Y.-Y. Zhang, L. Wang, F. Liu, L.-L. Cao, J. Yang, C. Qiao, Y. Ye, *European Journal of Medicinal Chemistry* **2014**, *80*, 535–542.
[62] M. Cushman, D. Nagarathnam, D. Gopal, A. K. Chakraborti, C. M. Lin, E. Hamel, *J. Med. Chem.* **1991**, *34*, 2579–2588.
[63] S. Bolte, F. P. Cordelières, *Journal of Microscopy* **2006**, *224*, 213–232.
[64] M. Rempfler, V. Stierle, K. Ditzel, S. Kumar, P. Paulitschke, B. Andres, B. H. Menze, *Medical Image Analysis* **2018**, *48*, 147–161.
[65] L. Pecqueur, C. Duellberg, B. Dreier, Q. Jiang, C. Wang, A. Plückthun, T. Surrey, B. Gigant, M. Knossow, *Proceedings of the National Academy of Sciences* **2012**, *109*, 12011–12016.
[66] G. La Sala, N. Olieric, A. Sharma, F. Viti, F. de Asis Balaguer Perez, L. Huang, J. R. Tonra, G. K. Lloyd, S. Decherchi, J. F. Díaz, M. O. Steinmetz, A. Cavalli, *Chem* **2019**, *5*, 2969–2986.
[67] T. Mühlethaler, N. Olieric, V. A. Ehrhard, M. Wranik, J. Standfuss, A. Sharma, A. E. Prota, M. O. Steinmetz, in *Microtubules: Methods and Protocols* (Ed.: H. Inaba), Springer US, New York, NY, **2022**, pp. 349–374.
[68] W. Kabsch, *Acta Cryst D* **2010**, *66*, 125–132.
[69] P. Evans, *Acta Cryst D* **2006**, *62*, 72–82.
[70] M. D. Winn, C. C. Ballard, K. D. Cowtan, E. J. Dodson, P. Emsley, P. R. Evans, R. M. Keegan, E. B. Krissinel, A. G. W. Leslie, A. McCoy, S. J. McNicholas, G. N. Murshudov, N. S. Pannu, E. A. Potterton, H. R. Powell, R. J. Read, A. Vagin, K. S. Wilson, *Acta Cryst D* **2011**, *67*, 235–242.
[71] I. J. Tickle, C. Flensburg, P. Keller, W. Paciorek, A. Sharff, C. Vonrhein, G. Bricogne, "STARANISO (http://staraniso.globalphasing.org/cgi-bin/staraniso.cgi)," can be found under http://staraniso.globalphasing.org/cgi-bin/staraniso.cgi, **2018**.
[72] T. Weinert, N. Olieric, R. Cheng, S. Brünle, D. James, D. Ozerov, D. Gashi, L. Vera, M. Marsh, K. Jaeger, F. Dworkowski, E. Panepucci, S. Basu, P. Skopintsev, A. S. Doré, T. Geng, R. M. Cooke, M. Liang, A. E. Prota, V. Panneels, P. Nogly, U. Ermler, G. Schertler, M. Hennig, M. O. Steinmetz, M. Wang, J. Standfuss, *Nat Commun* **2017**, *8*, 542.
[73] O. S. Smart, T. O. Womack, C. Flensburg, P. Keller, W. Paciorek, A. Sharff, C. Vonrhein, G. Bricogne, *Acta Cryst D* **2012**, *68*, 368–380.
[74] P. D. Adams, P. V. Afonine, G. Bunkóczi, V. B. Chen, I. W. Davis, N. Echols, J. J. Headd, L.-W. Hung, G. J. Kapral, R. W. Grosse-Kunstleve, A. J. McCoy, N. W. Moriarty, R. Oeffner, R. J. Read, D. C. Richardson, J. S. Richardson, T. C. Terwilliger, P. H. Zwart, *Acta Cryst D* **2010**, *66*, 213–221.
[75] P. Emsley, K. Cowtan, *Acta Cryst D* **2004**, *60*, 2126–2132.
